# Supplementary figures and images for: The dinucleotide composition of the Zika virus genome is shaped by conflicting evolutionary pressures in mammalian hosts and mosquito vectors
Source: PLoS Biol. 2021 Apr 19;19(4):e3001201. doi: 10.1371/journal.pbio.3001201 (PMC8084339; doi:10.1371/journal.pbio.3001201)

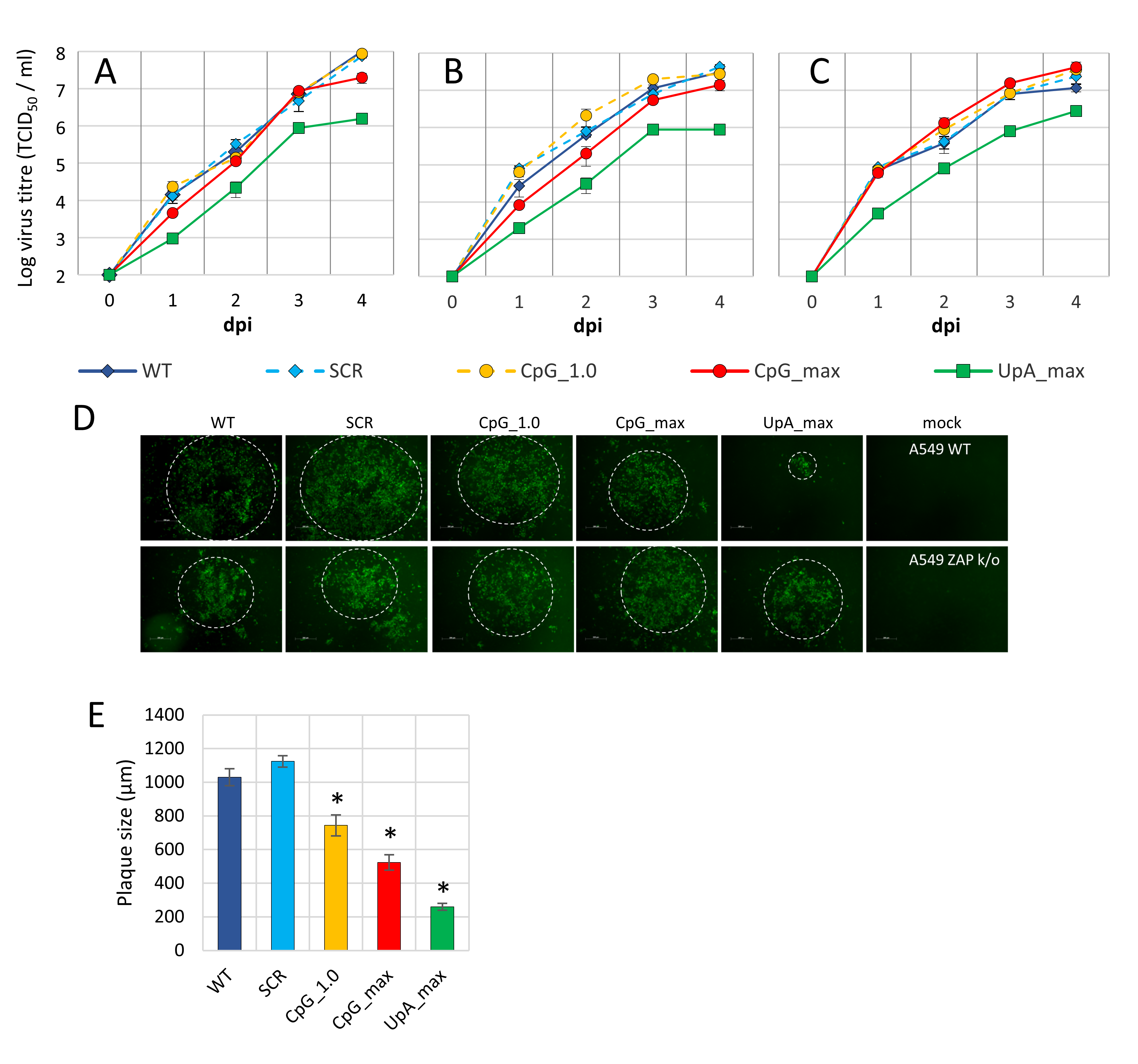

Supplement: S1 Fig — Growth curves on RNaseL knockout (A), OAS1 knockout (B), and OAS3 knockout (C) A549 cells. Cells were infected with mutant ZIKV viruses at 1 RNA/cell. At the indicated dpi, the 50% tissue culture infectious dose was determined by end-point dilution assays. Data points represent the average of 2 biological experiments and the error bars indicate 1 standard error of the mean. (D) Representative images of ZIKV induced immunoplaques in A549 and A549 ZAP knockout cells. Cells were fixed and stained for ZIKV E protein with primary antibody 4G2 and secondary Alexa Fluor 488 (green). Results of an immunoplaque assay on Vero E6 cells (E). Data points represent the average plaque size, error bars indicate 1 standard error of the mean, and asterisks highlight significant differences from WT (p < 0.05, one-way ANOVA with Dunnett post hoc test). Please refer to S1 Data for the numerical values underlying panels A–C and E. dpi, days postinfection; OAS1, 2′-5′-oligoadenylate synthetase 1; OAS3, 2′-5′-oligoadenylate synthetase 3; RNaseL, ribonuclease L; SCR, scrambled control virus; WT, wild-type; ZIKV, Zika virus. (TIF) [file pbio.3001201.s001.tif]

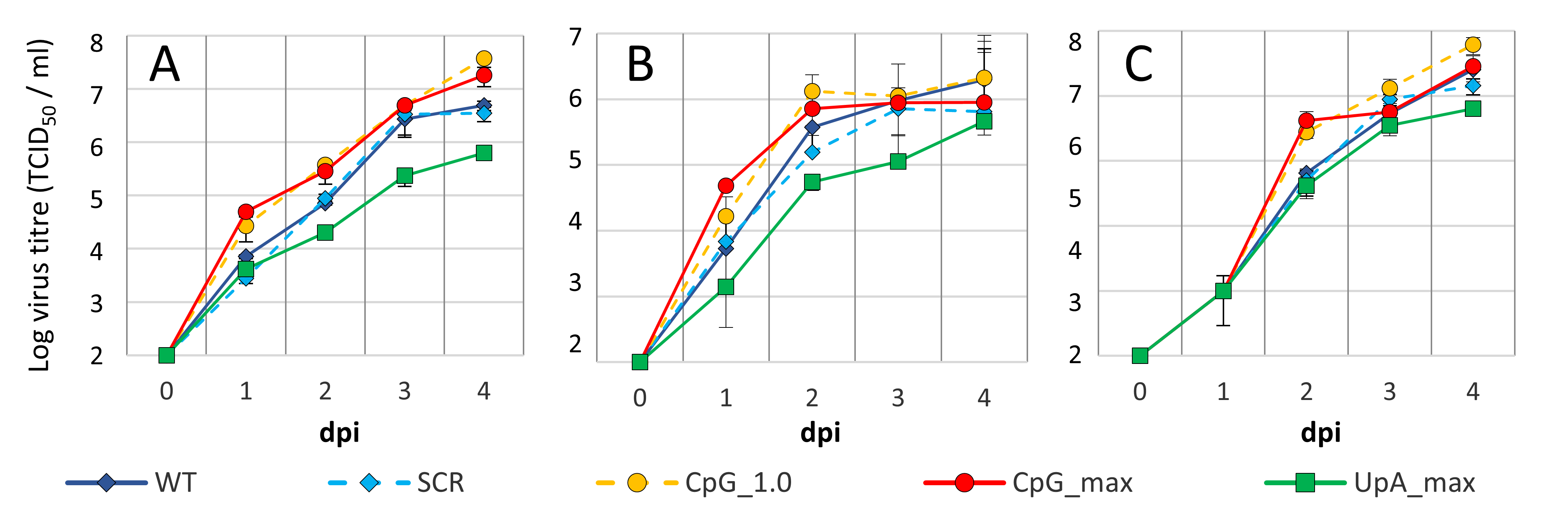

Supplement: S2 Fig — Ae. albopictus mosquito cell lines: C6/36 (A), U4.4 (B), and RML-12 (C) were infected with the mutant ZIKV viruses at 10 RNA/cell. At the indicated dpi, the 50% tissue culture infectious dose was determined by end-point dilution assays. Data points represent the average of 2 independent biological experiments and the error bars indicate 1 standard error of the mean. Please refer to S1 Data for the numerical values underlying this figure. dpi, days postinfection; SCR, scrambled control virus; ZIKV, Zika virus. (TIF) [file pbio.3001201.s002.tif]

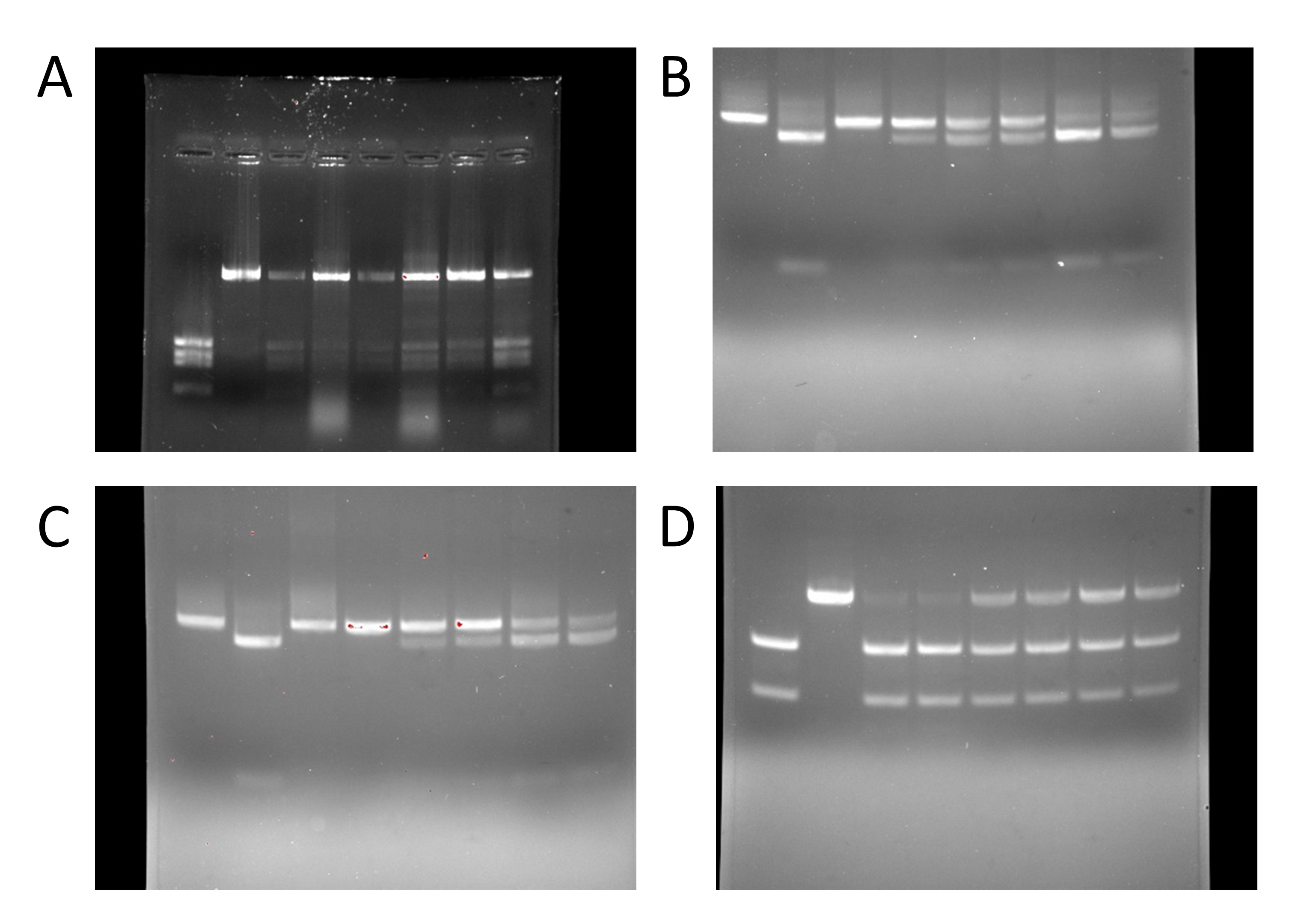

Supplement: S3 Fig — Complete and unmodified gel images corresponding to Fig 4. WT ZIKV mixed with either the SCR (A), CpG-high viruses (CpG_1.0 and CpG_max) (B, C), or UpA_max (D). SCR, scrambled control virus; WT, wild-type; ZIKV, Zika virus. (TIF) [file pbio.3001201.s003.tif]

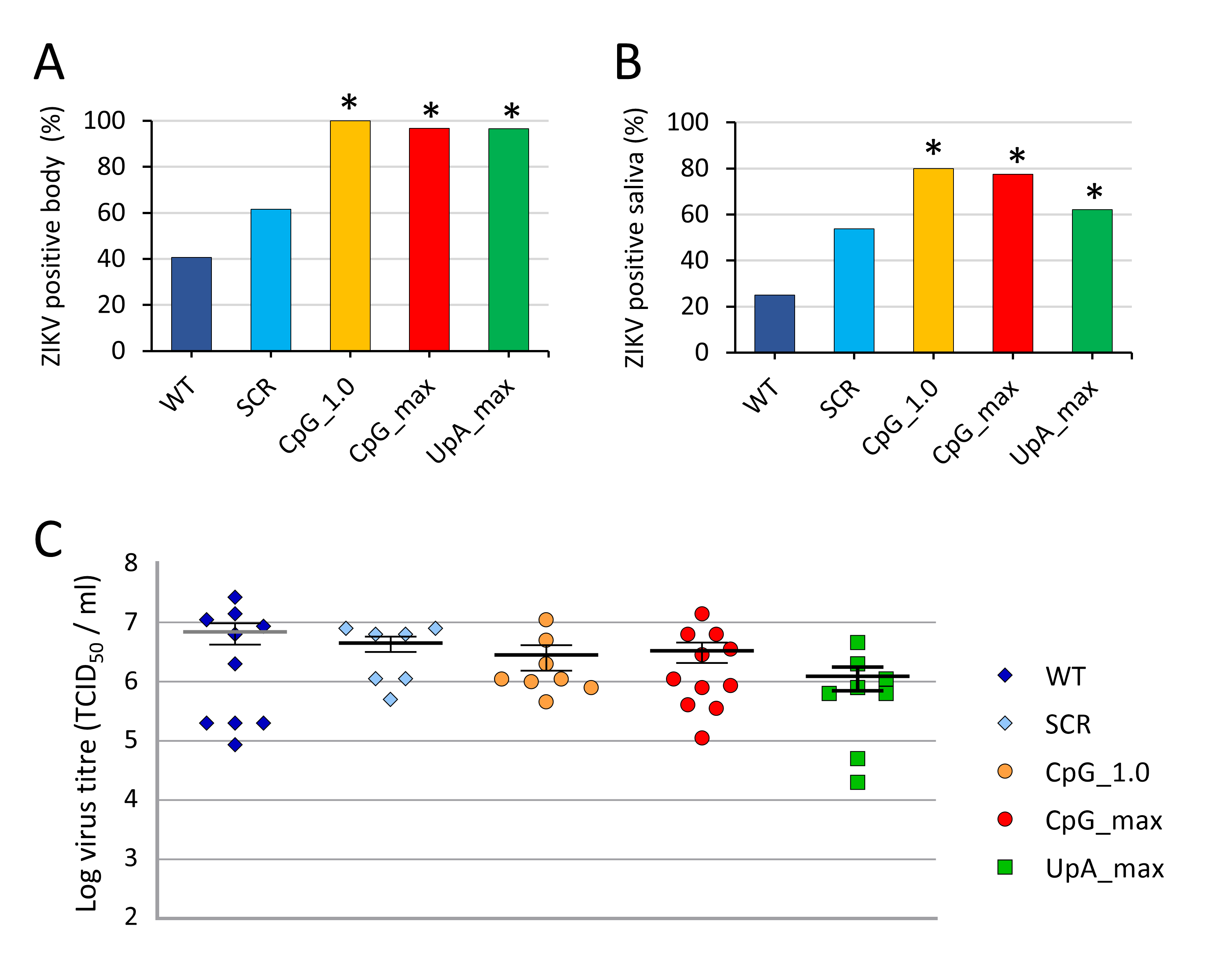

Supplement: S4 Fig — Aedes mosquitoes were offered an infectious blood meal containing equal infectious virus titers of each of the Zika viruses. Mosquito body homogenates (A) and mosquito saliva (B) were inoculated on Vero E6 cells to detect the presence of infectious virus. Bars represent the percentage of ZIKV-positive samples with n varied between 13 and 32 engorged mosquitoes per experimental group. Asterisks represent significant difference from WT (Fisher exact test, p < 0.01). The infectious virus titer inside ZIKV-positive mosquito bodies was determined by end-point dilution assay (C). Data points indicate individual mosquito samples and the mean and SEM for each experimental group are displayed. Please refer to S1 Data for the numerical values underlying this figure. (TIF) [file pbio.3001201.s004.tif]
