# Supplementary material for: The dinucleotide composition of the Zika virus genome is shaped by conflicting evolutionary pressures in mammalian hosts and mosquito vectors
Source: PLoS Biol. 2021 Apr 19;19(4):e3001201. doi: 10.1371/journal.pbio.3001201 (PMC8084339; doi:10.1371/journal.pbio.3001201)
Supplement: S1 Table — CpG and UpA dinucleotide composition of the mutated region in ZIKV and the codon adaptation index compared to both Homo sapiens and Drosophila melanogaster. Additional numerical values that underlie the values presented in this table are available in S1 Data. (PDF) [file pbio.3001201.s005.pdf]

Supplement table 1 – Composition of mutated region

	Total nt	*Freq G + C*	*Total CpG*	*Ratio O/E CpG*	*Total UpA*	*Ratio O/E UpA*	*CAI[a] Hs*	*CAI[b] Dm*
Wildtype	10727	0.51	308	0.45	342	0.54	0.71	0.60
Wildtype	1654	0.53	55	0.49	57	0.64	0.77	0.64
SCR	1654	0.53	55	0.49	49	0.55	0.76	0.65
CpG_1.0	1654	0.46	114	1.01	57	0.64	0.70	0.63
CpG_max	1654	0.61	245	1.62	57	0.93	0.67	0.71
UpA_max	1654	0.53	55	0.68	207	1.71	0.63	0.51

[a] Codon adaptation index (CAI) calculated against the codon usage table of Homo sapiens (Hs)

[b] CAI calculated against the codon usage table of Drosophila melanogaster (Dm)
