# Supplementary material for: The dinucleotide composition of the Zika virus genome is shaped by conflicting evolutionary pressures in mammalian hosts and mosquito vectors
Source: PLoS Biol. 2021 Apr 19;19(4):e3001201. doi: 10.1371/journal.pbio.3001201 (PMC8084339; doi:10.1371/journal.pbio.3001201)
Supplement: S1 Text — Accession numbers that refer to RNA sequences from Homo sapiens, Aedes aegypti, and the flavivirus genomes that were used to generate Fig 1A and 1C. (PDF) [file pbio.3001201.s006.pdf]

H. Sapiens	*Ae. Aegypti*	flaviviruses	ZIKV isolates
NM_000043.6	XM_021837896.1	DQ235145	KU365778
NM_000061.3	XM_021838519.1	AY323490	KU497555.1
NM_000068.4	XM_021838576.1	AF331718	KU955595.1
NM_000101.4	XM_021856355.1	AF253419	KU955593.1
NM_000116.5	XM_021837651.1	NC_001809	KX247646.1
NM_000117.3	XR_002502975.1	DQ235152	KU744693.1
NM_000148.4	XM_021848511.1	DQ235151	MK241417.1
NM_000151.4	XR_002502976.1	DQ235153	MN100039.1
NM_000184.3	XR_002501108.1	AY323489	KX117076.1
NM_000193.4	XR_002500046.1	PWARPT	MH513600.1
NM_000195.5	XR_002499437.1	DQ235149	KY785466.1
NM_000221.3	XR_002500378.1	TEU27495	KY014320.2
NM_000233.4	XM_021839360.1	KU761576	MK028857.1
NM_000244.3	XM_021855092.1	L40361	KU922960.1
NM_000285.4	XR_002499655.1	DQ235144	KY014319.2
NM_000289.6	XM_001660351.2	DQ235150	KY014300.2
NM_000325.6	XM_001653904.2	DQ235148	KY325476.1
NM_000334.4	XR_002499247.1	DQ235146	KX830930.1
NM_000355.4	XM_021843798.1	KF917535	KU527068
NM_000360.4	XM_021841335.1	AY632536	
NM_000380.4	XM_021839798.1	AY632538	
NM_000420.3	XR_002500965.1	KF917538	
NM_000442.5	XM_001649659.2	DVU88536	
NM_000455.5	XM_001653108.2	DENRCG	
NM_000463.3	XR_002498938.1	DENCME	
NM_000486.6	XM_001651609.2	AF326573	
NM_000487.6	XM_001652798.2	LN849009	
NM_000514.4	XM_001649658.2	JEVCG	
NM_000531.6	XR_002500826.1	EU082200	
NM_000603.5	XR_002500705.1	AY898809	
NM_000661.5	XR_002500569.1	AF161266	
NM_000666.3	XR_002501273.1	DQ525916	
NM_000679.4	XR_002500752.1	AY453411	
NM_000700.3	XR_002499843.1	KUNCG	
NM_000705.4	XM_021853272.1	WNFCG	
NM_000717.5	XM_021847017.1	EU082199	
NM_000723.5	XM_021839613.1	NC_009029	
NM_000742.4	XM_021846941.1	MF380434	
NM_000748.3	XR_002500629.1	NC_009028	
NM_000784.4	XM_001661645.2	MF461639	
NM_000794.5	XR_002501303.1	KC734552	
NM_000808.4	XM_021857092.1	NC_018705	
NM_000809.4	XM_001650132.2	KF192951	
NM_000842.5	XR_002499034.1	DQ859059	
NM_000845.3	XR_002500400.1	DQ859063	
NM_000848.4	XM_001659562.2	DQ859058	
NM_000862.3	XM_021844096.1	X03700	
NM_000869.6	XM_021854409.1	NC_008718	
NM_000882.4	XM_021839364.1	NC_026624	

NM_000896.3	XM_021857090.1	NC_005039
NM_000911.4	XM_021847667.1	AF160193
NM_000932.5	XM_021856647.1	NC_026620
NM_000936.4	XM_021839537.1	AJ242984
NM_000952.5	XM_021850851.1	AJ299445
NM_000954.6	XM_001656983.2	KJ469370
NM_000961.4	XM_021856340.1	NC_034007
NM_001001.5	XM_021837967.1	AF144692
NM_001001349.2	XM_021856059.1	NC_005064
NM_001001437.4	XR_002499502.1	JQ268258
NM_001001479.4	XR_002499293.1	NC_024299
NM_001001547.3	XM_021850832.1	NC_027817
NM_001001563.5	XR_002502609.1	NC_001564
NM_001001580.2	XM_021856422.1	GQ165809
NM_001001694.3	XR_002499302.1	NC_012932
NM_001001715.4	XM_021850829.1	KC505248
NM_001001722.2	XR_002499606.1	NC_012671
NM_001001913.2	XM_021842531.1	HE574574
NM_001001918.1	XR_002502037.1	NC_008604
NM_001001952.1	XR_002499177.1	KX669689
NM_001001957.2	XM_021839538.1	DQ400858
NM_001001958.1	XR_002503231.1	NC_033694
NM_001001965.1	XM_001649656.2	NC_024017
NM_001001991.3	XM_021856243.1	MF139576
NM_001002006.3	XR_002500221.1	NC_017086
NM_001002249.2	XR_002500915.1	NC_016997
NM_001002251.3	XM_021849176.1	KC692068
NM_001002261.4	XM_001652840.2	KC496020
NM_001002262.4	XM_021842437.1	MF139575
NM_001002294.3	XR_002500263.1	NC_024805
NM_001002814.3	XM_001653251.2	EU159426
NM_001002879.1	XR_002502942.1	MG587038
NM_001002914.3	XR_002499522.1	MH158418
NM_001003443.3	XM_021838320.1	MH158417
NM_001003745.2	XR_002502671.1	MH158416
NM_001003760.5	XM_021842955.1	MH158415
NM_001003789.3	XR_002500597.1	
NM_001003845.3	XM_001655319.2	
NM_001003897.2	XR_002498878.1	
NM_001003937.3	XR_002501094.1	
NM_001003941.3	XM_001649647.2	
NM_001004.4	XR_002500515.1	
NM_001004056.2	XR_002502072.1	
NM_001004135.2	XM_021843923.1	
NM_001004298.4	XR_002500009.1	
NM_001004416.3	XM_021848799.1	
NM_001004463.2	XR_002500395.1	
NM_001004705.2	XR_002499348.1	
NM_001004723.3	XR_002500819.1	
NM_001004732.2	XR_002500043.1	

NM_001004738.2	XM_021854178.1
NM_001004750.1	XR_002502353.1
NM_001004752.2	XR_002502492.1
NM_001004757.2	XM_021839628.1
NM_001005160.3	XR_002500971.1
NM_001005163.2	XM_021843332.1
NM_001005165.2	XM_001649672.2
NM_001005166.5	XM_021844475.1
NM_001005172.2	XM_001653220.2
NM_001005198.2	XM_001656328.2
NM_001005241.4	XM_021842532.1
NM_001005279.3	XR_002503024.1
NM_001005284.1	XM_021842460.1
NM_001005289.5	XR_002499049.1
NM_001005369.1	XM_021841437.1
NM_001005404.4	XM_021844264.1
NM_001005416.2	XM_021851628.1
NM_001005466.2	XR_002500997.1
NM_001005473.3	XM_021842795.1
NM_001005489.2	XR_002499861.1
NM_001005527.3	XR_002502233.1
NM_001005781.2	XM_021852829.1
NM_001005853.1	XM_001661458.2
NM_001006109.1	XM_021855606.1
NM_001006946.2	XM_001656229.2
NM_001007025.2	XM_021850900.1
NM_001007089.4	XM_021844310.1
NM_001007226.1	XR_002499834.1
NM_001007234.3	XR_002498801.1
NM_001007464.3	XR_002500163.1
NM_001007530.3	XR_002502932.1
NM_001007537.2	XR_002502345.1
NM_001007559.3	XM_001660308.2
NM_001008215.3	XM_001658818.2
NM_001008273.2	XM_001649665.2
NM_001008387.3	XM_021852850.1
NM_001008740.4	XM_021843268.1
NM_001008781.3	XM_001662067.2
NM_001009566.3	XR_002498673.1
NM_001009584.2	XM_021846071.1
NM_001009614.3	XM_001647908.2
NM_001009992.1	XM_001652936.2
NM_001010000.3	XR_002501908.1
NM_001010844.4	XM_021844314.1
NM_001010866.4	XM_001661544.2
NM_001010877.5	XM_021838751.1
NM_001011.4	XM_021850874.1
NM_001011658.4	XM_001659574.2
NM_001011709.3	XM_021857311.1
NM_001012455.2	XM_021848696.1

NM_001012727.2	XM_021851496.1
NM_001012763.2	XM_021844347.1
NM_001013253.2	XR_002498816.1
NM_001013438.3	XR_002502089.1
NM_001013630.2	XM_011494961.2
NM_001013690.5	XM_021857312.1
NM_001013699.3	XM_001659603.2
NM_001013837.2	XM_021842468.1
NM_001014283.2	XM_021853495.1
NM_001014336.2	XM_001658477.2
NM_001014342.3	XM_021852378.1
NM_001014435.2	XR_002501980.1
NM_001014447.3	XM_021837982.1
NM_001014999.3	XM_021850754.1
NM_001015.5	XM_021849319.1
NM_001015049.3	XR_002500809.1
NM_001015050.3	XM_021848995.1
NM_001015072.4	XM_021856296.1
NM_001017395.5	XM_001651099.2
NM_001017416.2	XM_021844340.1
NM_001017926.3	XM_021844346.1
NM_001017970.3	XR_002500570.1
NM_001018100.5	XM_021847572.1
NM_001018136.3	XR_002501642.1
NM_001018160.2	XM_021842896.1
NM_001018161.2	XM_021853485.1
NM_001019.5	XM_021850768.1
NM_001020821.2	XM_021844306.1
NM_001024074.3	XM_001651095.2
NM_001024381.2	XM_021855457.1
NM_001024383.2	XR_002499044.1
NM_001024593.2	XM_021842264.1
NM_001024601.3	XM_021841334.1
NM_001024628.3	XM_021844313.1
NM_001024662.3	XR_002503129.1
NM_001024855.3	XR_002503136.1
NM_001024924.2	XM_021844246.1
NM_001025076.2	XR_002500336.1
NM_001025081.2	XM_021853642.1
NM_001025107.3	XM_021839614.1
NM_001025160.3	XM_021850164.1
NM_001025238.2	XR_002502762.1
NM_001029875.3	XM_001659747.3
NM_001029891.3	XR_002499399.1
NM_001029955.4	XM_021839505.1
NM_001031698.3	XM_021841432.1
NM_001031713.3	XR_002501504.1
NM_001031735.3	XM_021849007.1
NM_001031835.3	XM_021857462.1
NM_001032.5	XM_001657977.2

NM_001033117.3	XM_001660597.2
NM_001033602.4	XM_001654891.2
NM_001033855.3	XR_002502700.1
NM_001034172.4	XM_021837394.1
NM_001034957.3	XM_001651695.2
NM_001035003.2	XR_002498611.1
NM_001037132.4	XM_021846196.1
NM_001037165.2	XR_002499975.1
NM_001037232.4	XR_002500384.1
NM_001037330.3	XM_021842231.1
NM_001037331.3	XM_021844343.1
NM_001037442.4	XR_002500401.1
NM_001037499.2	XR_002499402.1
NM_001037553.2	XM_021844365.1
NM_001037582.3	XR_002498598.1
NM_001037671.4	XM_001654389.2
NM_001038707.2	XM_021847927.1
NM_001039130.2	XR_002501977.1
NM_001039211.3	XM_021839673.1
NM_001039213.4	XR_002500499.1
NM_001039348.3	XM_021854226.1
NM_001039374.5	XR_002500473.1
NM_001039656.1	XM_021855298.1
NM_001039664.2	XR_002499666.1
NM_001039780.4	XM_001662134.2
NM_001040025.3	XR_002501421.1
NM_001040105.2	XR_002500397.1
NM_001040107.2	XR_002499170.1
NM_001040110.2	XM_001659590.2
NM_001040126.2	XM_021848608.1
NM_001040272.6	XR_002498935.1
NM_001040284.3	XR_002502283.1
NM_001040443.3	XM_021837869.1
NM_001040647.2	XM_021839369.1
NM_001042351.3	XM_021844624.1
NM_001042361.5	XM_001649652.2
NM_001042370.2	XR_002501342.1
NM_001042422.3	XR_002499384.1
NM_001042423.3	XM_001651978.2
NM_001042428.2	XM_021856855.1
NM_001042442.3	XM_001661646.2
NM_001042450.4	XR_002499112.1
NM_001042459.3	XR_002499931.1
NM_001042469.3	XM_021844219.1
NM_001042574.3	XR_002501535.1
NM_001042616.3	XR_002498794.1
NM_001042705.3	XM_021844363.1
NM_001043353.2	XM_021848994.1
NM_001045477.4	XR_002498918.1
NM_001063.4	XM_001660307.2

NM_001076683.2	XM_021843025.1
NM_001077.4	XM_021848999.1
NM_001077188.2	XM_021851716.1
NM_001077194.2	XR_002498936.1
NM_001077471.3	XM_021839357.1
NM_001077477.3	XR_002502028.1
NM_001077480.3	XM_001655174.2
NM_001077526.3	XM_021844469.1
NM_001077619.2	XM_021844368.1
NM_001077653.2	XM_001652515.2
NM_001077664.3	XM_021837887.1
NM_001078170.2	XR_002501641.1
NM_001079817.3	XM_001648290.2
NM_001079882.2	XM_001652439.1
NM_001080121.3	XR_002501234.1
NM_001080446.3	XM_021854213.1
NM_001080482.4	XR_002499813.1
NM_001080503.3	XM_001650651.2
NM_001080534.3	XR_002502882.1
NM_001080744.2	XM_001650627.2
NM_001080998.2	XM_001658763.2
NM_001081492.2	XM_021848027.1
NM_001081573.3	XM_001660306.2
NM_001081675.3	XM_021844356.1
NM_001083592.2	XM_021851489.1
NM_001083600.3	XM_021856403.1
NM_001083956.2	XM_021844736.1
NM_001085426.3	XM_021850730.1
NM_001093729.2	XM_021853349.1
NM_001097605.2	XR_002500998.1
NM_001098212.2	XR_002501864.1
NM_001098405.2	XM_021837888.1
NM_001098412.2	XM_021839564.1
NM_001098418.2	XM_021844309.1
NM_001098504.2	XM_021843047.1
NM_001098519.2	XM_021853610.1
NM_001098525.3	XR_002501503.1
NM_001098535.1	XR_002502990.1
NM_001098670.2	XR_002501749.1
NM_001098818.4	XR_002501620.1
NM_001099403.2	XR_002501587.1
NM_001099650.2	XM_001660303.2
NM_001099669.2	XM_001655670.2
NM_001099670.3	XM_021856701.1
NM_001099681.2	XR_002498829.1
NM_001099733.2	XR_002499945.1
NM_001099735.2	XM_001654902.2
NM_001099746.2	XM_011494663.2
NM_001099783.2	XM_021853577.1
NM_001099857.5	XM_021837745.1

NM_001100407.3	XM_001659051.2
NM_001100411.3	XM_021844233.1
NM_001100620.3	XM_001649537.2
NM_001100621.3	XR_002501422.1
NM_001100625.3	XM_021844241.1
NM_001101311.2	XM_001661485.2
NM_001101362.3	XM_021838675.1
NM_001102470.2	XM_001660722.2
NM_001102559.2	XM_001654006.2
NM_001102562.3	XM_021851408.1
NM_001105.5	XR_002500135.1
NM_001105207.3	XR_002499366.1
NM_001105513.3	XM_021847919.1
NM_001105518.2	XM_021844852.1
NM_001105558.1	XR_002500772.1
NM_001105563.2	XM_001654652.2
NM_001105569.3	XM_001655668.2
NM_001105573.2	XR_002501293.1
NM_001109754.4	XM_021845365.1
NM_001110354.2	XM_021855447.1
NM_001111035.3	XR_002498847.1
NM_001111307.2	XM_001661984.2
NM_001111309.1	XM_001648207.2
NM_001113324.3	XM_021845366.1
NM_001113348.2	XR_002502445.1
NM_001113511.2	XM_021851409.1
NM_001113525.2	XM_021855190.1
NM_001113535.2	XM_011494756.2
NM_001113546.2	XM_021837396.1
NM_001114086.2	XM_021839356.1
NM_001114092.2	XR_002499894.1
NM_001114182.3	XM_021839954.1
NM_001114377.2	XM_021849169.1
NM_001114759.3	XM_021853335.1
NM_001117.5	XM_001660301.2
NM_001122630.2	XR_002501618.1
NM_001122679.2	XM_021841219.1
NM_001122825.2	XR_002502799.1
NM_001122847.3	XR_002500899.1
NM_001122853.3	XM_021856183.1
NM_001122898.3	XM_021849333.1
NM_001123366.2	XM_021849097.1
NM_001123377.2	XM_021851594.1
NM_001126129.2	XM_021855455.1
NM_001126339.3	XM_001654900.2
NM_001127223.1	XM_001662456.2
NM_001127255.1	XR_002499500.1
NM_001127361.3	XM_021855286.1
NM_001127362.2	XM_021857197.1
NM_001127392.3	XM_001660623.2

NM_001127493.3	XR_002501976.1
NM_001127585.2	XM_001663928.2
NM_001127644.2	XM_001656568.2
NM_001127670.4	XM_021844167.1
NM_001127698.2	XM_001651203.2
NM_001127700.2	XM_001657679.2
NM_001127895.2	XM_021856850.1
NM_001128310.3	XM_021851424.1
NM_001128612.2	XM_001652656.2
NM_001128631.3	XM_001649339.2
NM_001128633.2	XR_002503107.1
NM_001128635.1	XM_021844753.1
NM_001128847.4	XM_021856122.1
NM_001128852.2	XR_002502691.1
NM_001128932.2	XM_021849171.1
NM_001129729.3	XM_021850780.1
NM_001129830.3	XR_002501746.1
NM_001129834.2	XR_002502713.1
NM_001129837.2	XR_002500879.1
NM_001130066.2	XM_001663845.2
NM_001130087.2	XM_021843046.1
NM_001130091.2	XM_001654004.2
NM_001130410.2	XM_021837649.1
NM_001130455.2	XM_021855580.1
NM_001130514.3	XM_001650449.2
NM_001130831.2	XM_021850537.1
NM_001130861.1	XM_021838725.1
NM_001130864.2	XM_021843888.1
NM_001130957.2	XM_021847795.1
NM_001130964.2	XM_021847666.1
NM_001131009.2	XM_011494845.2
NM_001134296.2	XR_002502881.1
NM_001134373.3	XR_002502471.1
NM_001134432.2	XR_002499370.1
NM_001134451.2	XR_002499583.1
NM_001134649.3	XR_002501238.1
NM_001134672.2	XR_002499730.1
NM_001134673.4	XM_021840542.1
NM_001134759.2	XM_021839373.1
NM_001134771.2	XR_002502651.1
NM_001134774.2	XM_021851738.1
NM_001135054.2	XM_021855291.1
NM_001135191.2	XM_001655010.2
NM_001135599.4	XM_021844133.1
NM_001135664.2	XM_001650203.2
NM_001135669.2	XM_021842667.1
NM_001135671.3	XM_021852016.1
NM_001135675.2	XM_021839525.1
NM_001135697.3	XM_001653180.2
NM_001135701.2	XM_021851066.1

NM_001135707.3	XM_021843082.1
NM_001135955.3	XR_002500073.1
NM_001135998.3	XM_021848386.1
NM_001135999.1	XM_021842130.1
NM_001136020.3	XM_021854203.1
NM_001136109.3	XM_001650025.2
NM_001136130.3	XM_021855773.1
NM_001136214.3	XM_001659702.2
NM_001136263.2	XM_001655178.2
NM_001136493.3	XR_002500273.1
NM_001136497.3	XM_021844159.1
NM_001136534.3	XM_021845707.1
NM_001136558.2	XM_021840544.1
NM_001136575.2	XM_021853620.1
NM_001137675.4	XM_021837676.1
NM_001139459.2	XM_021852244.1
NM_001139518.1	XR_002500600.1
NM_001142310.2	XR_002498881.1
NM_001142370.2	XM_021842137.1
NM_001142434.2	XM_001651303.2
NM_001142463.3	XM_001660714.2
NM_001142473.2	XM_021851011.1
NM_001142536.2	XM_021840603.1
NM_001142540.2	XM_001660213.2
NM_001142545.2	XM_011494649.2
NM_001142550.2	XM_021856473.1
NM_001142576.2	XR_002499485.1
NM_001142625.2	XM_021855770.1
NM_001142643.3	XM_021847664.1
NM_001142673.3	XM_021856987.1
NM_001142678.2	XM_021853575.1
NM_001142799.3	XM_021851607.1
NM_001142800.2	XM_021845066.1
NM_001142935.2	XM_021840541.1
NM_001142961.1	XM_001654459.3
NM_001143773.1	XR_002500728.1
NM_001143788.2	XR_002498927.1
NM_001143812.2	XR_002501772.1
NM_001143823.3	XM_021855836.1
NM_001143942.2	XM_001655657.2
NM_001144012.3	XR_002502915.1
NM_001144060.2	XR_002500851.1
NM_001144831.2	XM_001660202.2
NM_001144875.2	XR_002498767.1
NM_001144879.2	XM_001660604.2
NM_001144903.3	XM_021852964.1
NM_001144916.2	XM_021838257.1
NM_001144920.3	XM_001655303.2
NM_001144924.2	XM_021848578.1
NM_001144984.3	XR_002499555.1

NM_001145059.2	XR_002499734.1
NM_001145102.2	XM_021839876.1
NM_001145104.2	XM_021844095.1
NM_001145118.1	XR_002501686.1
NM_001145124.1	XM_021854208.1
NM_001145144.2	XM_021842582.1
NM_001145146.2	XM_021839543.1
NM_001145345.1	XM_001663374.2
NM_001145445.1	XM_021841635.1
NM_001145475.3	XM_001651495.2
NM_001145548.2	XM_001663015.2
NM_001145640.2	XR_002501458.1
NM_001145650.2	XM_001658827.2
NM_001145659.1	XR_002501262.1
NM_001145668.2	XM_001657903.2
NM_001145713.2	XM_001660414.2
NM_001145717.1	XR_002499433.1
NM_001145734.2	XM_021855626.1
NM_001145829.2	XM_021846359.1
NM_001145928.2	XR_002500618.1
NM_001146055.2	XM_001656485.3
NM_001146158.2	XR_002501440.1
NM_001146171.2	XM_021847573.1
NM_001146191.2	XR_002502754.1
NM_001146333.3	XM_021848953.1
NM_001146339.2	XM_021849088.1
NM_001146683.2	XR_002500632.1
NM_001146686.3	XR_002503020.1
NM_001148.6	XM_021845608.1
NM_001159286.2	XR_002502650.1
NM_001159322.2	XM_021855628.1
NM_001159379.2	XM_021839670.1
NM_001159554.1	XM_021850915.1
NM_001159601.2	XM_021847038.1
NM_001159740.2	XM_001660730.2
NM_001159773.2	XR_002503181.1
NM_001160002.2	XM_011495066.2
NM_001160116.2	XM_021849087.1
NM_001160125.2	XM_001657764.2
NM_001160166.2	XR_002499642.1
NM_001160176.4	XM_021852576.1
NM_001160224.2	XM_001659180.1
NM_001160244.2	XR_002502949.1
NM_001160300.2	XM_021846536.1
NM_001160302.1	XM_021849405.1
NM_001161330.2	XM_021854866.1
NM_001161368.3	XM_021852995.1
NM_001161454.1	XM_001652365.2
NM_001161527.2	XM_021849170.1
NM_001161562.3	XM_001649750.2

NM_001161584.2	XM_021845159.1
NM_001161662.2	XM_021856425.1
NM_001162407.1	XM_021853208.1
NM_001162862.2	XM_001652313.2
NM_001162936.2	XR_002498855.1
NM_001163335.2	XM_021841531.1
NM_001163484.2	XM_001661348.3
NM_001163560.3	XM_021845131.1
NM_001163735.2	XM_021856683.1
NM_001164161.2	XM_021844171.1
NM_001164283.3	XM_001661007.2
NM_001164319.2	XM_021857597.1
NM_001164381.2	XM_021848580.1
NM_001164399.2	XM_021843277.1
NM_001164442.2	XM_001661784.2
NM_001164538.2	XR_002501499.1
NM_001164542.2	XR_002503273.1
NM_001164619.2	XR_002503071.1
NM_001164638.3	XM_021843979.1
NM_001164690.2	XM_021837968.1
NM_001164694.2	XR_002502498.1
NM_001164738.1	XM_021848396.1
NM_001164752.2	XR_002499278.1
NM_001164778.2	XR_002498804.1
NM_001164781.2	XM_021849025.1
NM_001164831.3	XM_021844116.1
NM_001164835.2	XM_021852697.1
NM_001165255.2	XM_021856590.1
NM_001165412.2	XM_021837995.1
NM_001165885.2	XM_021847325.1
NM_001165928.3	XM_001663026.2
NM_001165945.2	XM_001649671.2
NM_001166208.2	XM_011495224.2
NM_001166280.2	XM_021843763.1
NM_001166287.2	XM_001648972.2
NM_001166387.4	XM_021853336.1
NM_001166393.2	XM_021850949.1
NM_001166533.2	XM_021839541.1
NM_001166703.2	XM_001661166.2
NM_001166704.2	XM_021850960.1
NM_001167605.2	XR_002502270.1
NM_001167608.3	XM_001663498.2
NM_001167890.2	XM_021841326.1
NM_001167915.3	XR_002500373.1
NM_001168323.2	XM_001652071.2
NM_001168390.2	XM_021850955.1
NM_001170330.1	XR_002503092.1
NM_001170423.2	XM_001659755.2
NM_001170670.2	XM_021844190.1
NM_001170687.4	XM_021843079.1

NM_001170741.3	XM_021844324.1
NM_001170760.2	XM_001648225.2
NM_001170881.2	XM_021857502.1
NM_001171025.2	XM_001650968.2
NM_001171653.2	XR_002501970.1
NM_001171743.3	XR_002502948.1
NM_001171934.1	XR_002501813.1
NM_001172568.2	XM_021850749.1
NM_001172640.2	XM_021851897.1
NM_001172671.2	XM_021850115.1
NM_001172771.2	XM_021845376.1
NM_001172772.2	XM_021855629.1
NM_001172815.3	XM_001651216.2
NM_001172818.1	XM_001658820.2
NM_001173129.2	XM_021847574.1
NM_001174070.3	XM_021848671.1
NM_001174085.2	XM_021854864.1
NM_001174104.2	XM_001661747.2
NM_001174105.2	XR_002502454.1
NM_001174127.2	XM_021842675.1
NM_001174166.2	XR_002500097.1
NM_001177310.2	XM_021844712.1
NM_001177704.3	XM_001664182.2
NM_001177800.2	XR_002500196.1
NM_001177801.2	XR_002503135.1
NM_001177998.2	XM_001656650.2
NM_001178030.2	XM_021857081.1
NM_001178094.2	XM_021849163.1
NM_001178106.1	XR_002503255.1
NM_001178140.2	XR_002500520.1
NM_001184693.2	XM_001655691.2
NM_001184696.1	XR_002502340.1
NM_001184721.2	XM_001655646.2
NM_001184751.2	XM_001661338.2
NM_001184760.2	XR_002500561.1
NM_001184792.2	XR_002501955.1
NM_001184866.2	XM_021853605.1
NM_001184872.2	XM_021843602.1
NM_001184903.1	XM_001662189.3
NM_001184970.3	XM_001660967.2
NM_001184974.2	XM_021857066.1
NM_001184993.2	XM_001658441.2
NM_001185054.2	XM_021850172.1
NM_001185119.1	XM_021844521.1
NM_001185149.1	XR_002500902.1
NM_001190158.1	XR_002500194.1
NM_001190447.2	XM_021839739.1
NM_001190462.2	XM_021846542.1
NM_001190482.2	XM_021848401.1
NM_001190789.2	XM_001663679.2

NM_001190791.2	XM_021855299.1
NM_001190801.2	XM_021843729.1
NM_001190811.2	XM_021856205.1
NM_001190848.2	XM_021857546.1
NM_001190882.3	XM_001651206.2
NM_001190983.2	XM_021839639.1
NM_001191029.2	XR_002503257.1
NM_001191037.2	XM_001657417.2
NM_001191059.4	XR_002501586.1
NM_001193272.2	XR_002503021.1
NM_001193360.2	XM_021840501.1
NM_001193461.2	XM_021856559.1
NM_001193487.3	XM_021845705.1
NM_001193503.2	XM_021840487.1
NM_001193557.2	XM_001656559.2
NM_001194958.2	XR_002501801.1
NM_001195035.2	XM_021846137.1
NM_001195076.2	XR_002501597.1
NM_001195104.3	XM_021856937.1
NM_001195203.4	XM_021854860.1
NM_001195249.2	XM_021844090.1
NM_001195296.2	XM_021847596.1
NM_001195303.3	XR_002501581.1
NM_001195307.2	XM_021852226.1
NM_001195578.2	XR_002498810.1
NM_001195803.2	XM_021838741.1
NM_001198541.3	XM_021845943.1
NM_001198621.4	XR_002501889.1
NM_001198689.2	XM_021842372.1
NM_001198756.1	XM_001654990.2
NM_001198855.1	XM_001661988.2
NM_001198898.2	XM_021843294.1
NM_001198909.2	XM_001648723.2
NM_001199041.2	XM_021837694.1
NM_001199042.2	XM_001662155.2
NM_001199081.3	XM_001660041.2
NM_001199135.3	XM_021837678.1
NM_001199148.1	XM_021840612.1
NM_001199255.3	XM_001660277.2
NM_001199262.3	XR_002500072.1
NM_001199379.2	XM_021845792.1
NM_001199388.3	XM_001657239.3
NM_001199427.2	XR_002503133.1
NM_001199463.2	XM_021850453.1
NM_001199570.2	XM_011495347.2
NM_001199636.2	XM_021852727.1
NM_001199637.2	XM_021843291.1
NM_001199642.1	XM_021845023.1
NM_001199661.1	XR_002500803.1
NM_001199708.2	XM_001660760.2

NM_001199752.3	XR_002498798.1
NM_001199771.3	XM_021852381.1
NM_001199805.1	XM_021846144.1
NM_001199837.3	XM_021847389.1
NM_001199851.1	XM_001653669.2
NM_001199859.3	XM_021843293.1
NM_001199876.1	XM_001657906.2
NM_001199987.2	XM_001655635.2
NM_001200051.2	XR_002501799.1
NM_001201365.2	XM_021848395.1
NM_001201473.2	XR_002501064.1
NM_001201480.2	XM_001663652.2
NM_001202409.2	XM_021856348.1
NM_001202410.2	XM_021857637.1
NM_001202498.2	XM_021845008.1
NM_001202555.2	XM_001657908.2
NM_001203261.2	XM_021843286.1
NM_001204066.2	XM_001658890.2
NM_001204078.2	XM_021853959.1
NM_001204145.3	XR_002498591.1
NM_001204148.3	XM_021845693.1
NM_001204175.2	XM_021848676.1
NM_001204190.2	XM_001650008.2
NM_001204212.2	XM_021840237.1
NM_001204221.2	XM_021844087.1
NM_001204290.2	XM_021847088.1
NM_001204293.2	XM_021847395.1
NM_001204408.2	XM_001654903.2
NM_001204505.3	XM_021847672.1
NM_001204514.2	XM_001661499.2
NM_001204698.2	XM_021840503.1
NM_001204803.2	XM_021843987.1
NM_001204887.2	XM_021844500.1
NM_001205301.2	XM_001652627.2
NM_001206482.2	XR_002501432.1
NM_001206488.3	XM_001650755.2
NM_001206627.2	XR_002500560.1
NM_001206671.4	XM_021853006.1
NM_001206924.2	XR_002500807.1
NM_001206994.2	XM_001651466.2
NM_001207037.2	XR_002499852.1
NM_001207042.3	XM_021849174.1
NM_001207051.2	XM_001657418.2
NM_001207053.2	XM_001652760.2
NM_001214907.1	XM_001658243.3
NM_001226.4	XM_021848786.1
NM_001228.4	XM_021857248.1
NM_001235.5	XM_021843728.1
NM_001242375.1	XM_021856224.1
NM_001242376.3	XM_021845689.1

NM_001242399.2	XM_021853873.1
NM_001242560.2	XR_002498978.1
NM_001242589.3	XM_001647950.2
NM_001242597.2	XM_021851961.1
NM_001242598.2	XM_021847323.1
NM_001242630.2	XM_001653229.2
NM_001242639.2	XM_021855068.1
NM_001242692.2	XM_021856681.1
NM_001242763.2	XM_001652384.2
NM_001242798.2	XM_021851567.1
NM_001242818.2	XM_021845075.1
NM_001242820.2	XM_021845789.1
NM_001242847.2	XM_021841454.1
NM_001242848.2	XM_001649759.2
NM_001242851.1	XR_002502780.1
NM_001242858.3	XR_002500034.1
NM_001242868.2	XR_002498946.1
NM_001242875.3	XM_021849999.1
NM_001243042.1	XM_021843324.1
NM_001243108.2	XR_002500096.1
NM_001243116.2	XM_001655235.2
NM_001243137.2	XM_021851745.1
NM_001243177.4	XM_021848500.1
NM_001243237.2	XM_001660369.2
NM_001243256.2	XM_001648309.2
NM_001243271.2	XR_002502328.1
NM_001243423.2	XM_021840502.1
NM_001243597.2	XM_021845448.1
NM_001243754.2	XM_021855103.1
NM_001243782.2	XM_021853044.1
NM_001243784.2	XM_021842664.1
NM_001243798.2	XM_021843415.1
NM_001243799.1	XM_021847262.1
NM_001243925.2	XM_021853718.1
NM_001243942.1	XM_001657893.2
NM_001243998.2	XM_001662691.2
NM_001244.4	XM_021844497.1
NM_001244015.2	XM_021853865.1
NM_001244192.2	XM_021850783.1
NM_001245005.2	XM_021853908.1
NM_001247997.2	XM_001648004.2
NM_001248002.2	XM_021856439.1
NM_001251962.2	XR_002500550.1
NM_001251963.2	XR_002499567.1
NM_001251973.2	XM_021857598.1
NM_001251974.2	XM_021855986.1
NM_001252007.1	XM_021838555.1
NM_001252120.2	XR_002501085.1
NM_001252226.2	XM_021844151.1
NM_001252269.2	XM_001662058.2

NM_001253723.2	XM_001656300.2
NM_001253750.1	XM_021842665.1
NM_001253793.2	XM_021851943.1
NM_001253826.2	XM_021839693.1
NM_001253846.2	XR_002499498.1
NM_001253853.3	XM_021851611.1
NM_001253891.2	XM_011495241.2
NM_001254.4	XR_002502586.1
NM_001256047.2	XR_002500610.1
NM_001256054.3	XM_021856685.1
NM_001256092.2	XM_001651573.2
NM_001256155.2	XM_001654825.3
NM_001256165.1	XM_001662721.2
NM_001256293.2	XM_001654683.2
NM_001256304.3	XM_001658422.3
NM_001256328.2	XM_021850491.1
NM_001256337.3	XM_021856443.1
NM_001256348.2	XR_002501117.1
NM_001256371.2	XM_021847995.1
NM_001256412.2	XM_021843833.1
NM_001256423.2	XR_002502269.1
NM_001256468.2	XR_002502606.1
NM_001256494.2	XM_001660777.2
NM_001256496.2	XM_001654663.2
NM_001256503.2	XM_001649169.2
NM_001256549.2	XM_021839552.1
NM_001256601.1	XM_021857554.1
NM_001256605.2	XM_021856624.1
NM_001256619.2	XM_001652103.2
NM_001256630.1	XM_001658226.2
NM_001256643.1	XR_002499161.1
NM_001256677.1	XR_002502195.1
NM_001256688.2	XR_002502099.1
NM_001256701.1	XR_002502041.1
NM_001256725.2	XR_002503095.1
NM_001256727.2	XM_001661421.2
NM_001256753.2	XM_021844756.1
NM_001256758.1	XM_001664031.2
NM_001256860.1	XM_021846816.1
NM_001256864.2	XM_001653428.2
NM_001256873.1	XM_021854109.1
NM_001256887.3	XM_021841327.1
NM_001256917.2	XR_002502159.1
NM_001257.5	XM_001663229.2
NM_001257230.2	XM_021849076.1
NM_001257282.2	XM_021844201.1
NM_001257414.2	XM_021856440.1
NM_001258028.2	XM_001651455.2
NM_001258208.2	XM_011495262.2
NM_001258216.1	XR_002502801.1

NM_001258252.1	XM_001654081.2
NM_001258264.1	XM_021839740.1
NM_001258269.1	XM_001648040.2
NM_001258298.2	XM_001655170.2
NM_001258324.1	XM_021852551.1
NM_001258325.1	XR_002502458.1
NM_001258331.2	XR_002500591.1
NM_001258355.2	XM_021845678.1
NM_001258373.2	XM_001651222.2
NM_001258379.2	XM_021848879.1
NM_001258395.2	XM_021843891.1
NM_001258399.2	XM_021854123.1
NM_001258416.2	XM_021838603.1
NM_001258418.2	XR_002503274.1
NM_001258447.1	XM_021843892.1
NM_001258450.2	XM_021842659.1
NM_001258453.2	XM_001650241.2
NM_001260510.2	XR_002502091.1
NM_001261409.1	XR_002499822.1
NM_001261818.2	XR_002501784.1
NM_001261837.2	XM_021851052.1
NM_001261838.2	XM_021850427.1
NM_001265578.2	XM_021843507.1
NM_001265608.2	XM_001663168.2
NM_001267040.1	XM_021854001.1
NM_001267064.2	XM_021842663.1
NM_001267571.2	XM_021856216.1
NM_001267574.2	XM_021843658.1
NM_001269048.3	XM_021841218.1
NM_001270375.2	XM_021846729.1
NM_001270400.2	XM_021843894.1
NM_001270411.2	XR_002503277.1
NM_001270420.2	XM_011494840.2
NM_001270437.2	XM_021852553.1
NM_001270453.2	XM_021854043.1
NM_001270492.2	XR_002502790.1
NM_001270501.2	XM_021844458.1
NM_001270530.2	XM_021852416.1
NM_001270532.2	XM_001651293.2
NM_001270601.2	XM_001660399.2
NM_001270623.2	XM_021846498.1
NM_001270695.1	XM_021846767.1
NM_001270709.2	XM_021846440.1
NM_001270733.2	XM_021843509.1
NM_001270766.2	XM_001662208.2
NM_001270841.2	XM_021848488.1
NM_001270895.2	XM_011495374.2
NM_001270941.2	XM_021842660.1
NM_001271012.2	XM_021852552.1
NM_001271023.2	XM_021856720.1

NM_001271213.2	XR_002501739.1
NM_001271286.2	XM_021842516.1
NM_001271427.2	XM_021843510.1
NM_001271442.1	XM_021848487.1
NM_001271519.2	XM_001659290.2
NM_001271560.2	XM_021853071.1
NM_001271573.2	XM_021843837.1
NM_001271696.3	XM_021844839.1
NM_001271719.2	XM_001654236.2
NM_001271734.2	XM_021843033.1
NM_001271780.2	XM_021846924.1
NM_001271823.2	XR_002499785.1
NM_001271825.2	XM_021844160.1
NM_001271830.2	XM_021857013.1
NM_001271834.2	XM_021856711.1
NM_001271872.3	XR_002501742.1
NM_001271900.2	XR_002501514.1
NM_001271918.2	XR_002502374.1
NM_001271926.3	XM_001661608.2
NM_001271970.2	XM_021853604.1
NM_001272042.2	XR_002500530.1
NM_001272053.2	XM_021840918.1
NM_001272077.2	XR_002499263.1
NM_001272104.2	XM_021851123.1
NM_001273.5	XM_021848162.1
NM_001276252.2	XM_001662946.2
NM_001276271.2	XR_002502637.1
NM_001276375.2	XM_001657456.2
NM_001276395.2	XM_021856714.1
NM_001276437.2	XM_021845103.1
NM_001276453.2	XR_002499367.1
NM_001276501.2	XM_021850823.1
NM_001276723.2	XR_002499280.1
NM_001276726.2	XM_021842508.1
NM_001277075.3	XM_021843670.1
NM_001277081.1	XR_002501804.1
NM_001277083.2	XM_021848640.1
NM_001277227.2	XR_002500478.1
NM_001277403.2	XM_021844185.1
NM_001277971.2	XM_021843834.1
NM_001278067.1	XR_002503244.1
NM_001278162.2	XM_021846791.1
NM_001278210.2	XM_001654001.2
NM_001278214.2	XM_001658199.2
NM_001278215.2	XM_001652124.2
NM_001278267.1	XM_001662720.2
NM_001278296.2	XM_001659087.2
NM_001278300.2	XM_021848501.1
NM_001278309.2	XM_021845690.1
NM_001278398.2	XM_001658579.2

NM_001278428.3	XM_021845355.1
NM_001278432.1	XM_021838747.1
NM_001278464.2	XM_021844156.1
NM_001278471.2	XR_002498691.1
NM_001278499.2	XM_021843681.1
NM_001278500.2	XR_002500391.1
NM_001278503.2	XM_021857106.1
NM_001278512.2	XM_021844836.1
NM_001278524.2	XM_001661870.2
NM_001278546.2	XM_021843656.1
NM_001278548.1	XR_002499824.1
NM_001278549.2	XM_021845287.1
NM_001278559.2	XM_001660321.2
NM_001278592.2	XR_002500057.1
NM_001278599.2	XM_001653625.3
NM_001278603.2	XM_021848638.1
NM_001278606.2	XM_001659405.2
NM_001278610.2	XR_002498945.1
NM_001278618.2	XM_021856315.1
NM_001278629.2	XM_021838855.1
NM_001278639.2	XM_021838860.1
NM_001278641.2	XM_001652025.2
NM_001278645.2	XM_021843718.1
NM_001278678.2	XM_001658777.2
NM_001278715.2	XM_001652492.2
NM_001278794.2	XM_001658036.3
NM_001278928.2	XM_021844400.1
NM_001279.4	XM_021838854.1
NM_001279349.2	XM_001656551.2
NM_001280550.2	XR_002499342.1
NM_001280795.2	XM_021847660.1
NM_001280799.2	XM_021843716.1
NM_001281518.3	XR_002499823.1
NM_001281520.2	XR_002502953.1
NM_001282068.2	XM_001662289.2
NM_001282113.2	XM_021842507.1
NM_001282117.2	XR_002498731.1
NM_001282190.2	XM_021838091.1
NM_001282195.2	XM_021845679.1
NM_001282215.2	XM_021838862.1
NM_001282237.2	XM_021840238.1
NM_001282305.1	XM_021856773.1
NM_001282310.2	XM_001659866.2
NM_001282356.2	XM_021856164.1
NM_001282398.2	XM_021845076.1
NM_001282418.2	XM_021851047.1
NM_001282419.3	XM_001654676.2
NM_001282456.4	XM_021854029.1
NM_001282466.2	XM_021844261.1
NM_001282597.3	XM_021846764.1

NM_001282624.2	XM_021848026.1
NM_001282706.2	XM_021849061.1
NM_001282715.2	XM_021851069.1
NM_001282719.2	XM_021848351.1
NM_001282753.2	XM_021856289.1
NM_001282755.2	XR_002501131.1
NM_001282790.2	XM_021854033.1
NM_001282792.2	XR_002499707.1
NM_001282796.2	XM_001658039.2
NM_001282800.2	XM_021854055.1
NM_001282803.2	XM_021838861.1
NM_001282806.2	XM_021847333.1
NM_001282851.2	XM_021850916.1
NM_001282854.2	XM_021843822.1
NM_001282885.2	XM_001653951.2
NM_001282914.2	XM_021844197.1
NM_001282920.1	XM_021846549.1
NM_001282921.2	XR_002501849.1
NM_001282933.2	XM_001652622.2
NM_001282959.2	XM_021844381.1
NM_001283004.3	XM_021839480.1
NM_001283023.2	XM_021841341.1
NM_001283027.1	XM_021854014.1
NM_001283060.2	XM_021851048.1
NM_001283235.3	XM_021849826.1
NM_001284210.2	XM_021854981.1
NM_001284502.2	XM_021846770.1
NM_001284511.2	XR_002500281.1
NM_001284528.2	XM_021850430.1
NM_001285.4	XM_021851125.1
NM_001285526.2	XM_021853547.1
NM_001286.5	XM_001654897.2
NM_001286035.2	XM_021846562.1
NM_001286051.2	XM_001660659.2
NM_001286094.2	XM_021844163.1
NM_001286107.2	XM_001663935.2
NM_001286139.2	XR_002498993.1
NM_001286197.2	XR_002503138.1
NM_001286206.2	XM_021845548.1
NM_001286210.2	XM_021845414.1
NM_001286354.1	XM_021842384.1
NM_001286399.2	XM_001650789.2
NM_001286427.1	XM_021840096.1
NM_001286536.2	XM_021844333.1
NM_001286537.2	XM_001663454.2
NM_001286550.2	XM_021857047.1
NM_001286563.3	XM_021838761.1
NM_001286583.2	XM_021838863.1
NM_001286613.2	XM_021848233.1
NM_001286639.2	XM_021857485.1

NM_001286655.1	XM_021854145.1
NM_001286688.2	XM_021840811.1
NM_001286695.1	XM_021844378.1
NM_001286700.2	XM_021846759.1
NM_001286702.2	XM_021852609.1
NM_001286749.2	XM_021844382.1
NM_001286751.2	XM_001661138.2
NM_001286756.2	XR_002501424.1
NM_001286760.1	XM_021838234.1
NM_001286810.2	XM_021857254.1
NM_001286828.2	XR_002500694.1
NM_001286830.2	XM_021843992.1
NM_001286957.2	XR_002499118.1
NM_001287005.2	XM_001660027.2
NM_001287060.2	XM_021856407.1
NM_001287136.1	XM_021846773.1
NM_001287190.2	XM_021844383.1
NM_001287262.2	XR_002500486.1
NM_001287390.3	XM_001659691.2
NM_001287394.2	XM_021844003.1
NM_001287422.2	XM_021851196.1
NM_001287430.1	XM_021849663.1
NM_001287432.2	XM_021850694.1
NM_001287509.2	XM_021844663.1
NM_001287513.2	XR_002501488.1
NM_001287525.2	XM_001651785.2
NM_001287601.1	XM_021847361.1
NM_001288566.2	XM_001657123.2
NM_001288568.2	XM_021847338.1
NM_001288582.2	XM_001650767.2
NM_001288643.2	XR_002498889.1
NM_001288653.2	XM_021851600.1
NM_001288662.1	XM_021850690.1
NM_001288705.3	XM_021851886.1
NM_001288713.1	XR_002499069.1
NM_001288732.2	XR_002500252.1
NM_001288746.2	XR_002499987.1
NM_001288752.2	XR_002499817.1
NM_001288836.1	XM_021846782.1
NM_001288951.2	XM_021839661.1
NM_001288967.2	XM_021843197.1
NM_001288975.2	XM_021847104.1
NM_001288980.2	XM_021852814.1
NM_001288989.2	XM_021853980.1
NM_001289035.2	XM_021856847.1
NM_001289037.2	XM_021843159.1
NM_001289057.2	XR_002499786.1
NM_001289129.2	XM_021857238.1
NM_001289152.2	XM_021856678.1
NM_001289173.2	XR_002500469.1

NM_001289180.2	XM_021838251.1
NM_001289184.2	XR_002500222.1
NM_001289189.2	XM_021849703.1
NM_001289396.1	XM_021846804.1
NM_001289399.1	XM_011494934.2
NM_001289911.2	XM_021843703.1
NM_001289975.1	XR_002501820.1
NM_001290030.2	XM_021839624.1
NM_001290059.2	XM_001653429.2
NM_001290184.2	XR_002498937.1
NM_001290194.2	XM_021838571.1
NM_001290233.2	XM_021853989.1
NM_001290258.2	XR_002501491.1
NM_001290360.3	XM_001660770.2
NM_001291281.3	XM_021854010.1
NM_001291308.3	XM_021852890.1
NM_001291328.2	XM_021856408.1
NM_001291454.2	XR_002501306.1
NM_001291491.2	XM_001650901.3
NM_001291527.2	XM_001657703.2
NM_001291588.2	XM_021851568.1
NM_001291700.1	XM_021852363.1
NM_001291716.2	XR_002501474.1
NM_001291719.2	XM_001650661.2
NM_001291732.2	XM_021842364.1
NM_001291738.1	XM_001647871.2
NM_001291813.2	XM_021842410.1
NM_001291826.2	XM_021842587.1
NM_001291837.2	XM_021841546.1
NM_001291860.2	XR_002503030.1
NM_001291873.3	XM_001650333.2
NM_001291879.2	XM_021854930.1
NM_001291896.2	XM_001647936.2
NM_001291918.2	XM_021841407.1
NM_001291931.2	XM_021842381.1
NM_001291956.3	XM_021851140.1
NM_001291979.2	XM_021843126.1
NM_001292004.2	XM_001652207.2
NM_001292018.2	XM_021853839.1
NM_001292048.2	XM_001659191.3
NM_001293213.2	XM_001652668.2
NM_001293628.2	XR_002499419.1
NM_001293810.1	XM_001649463.2
NM_001294332.2	XM_021853112.1
NM_001294335.2	XR_002502296.1
NM_001294338.2	XM_001657698.2
NM_001297424.2	XM_021852558.1
NM_001297435.1	XM_021839577.1
NM_001297440.2	XM_021845523.1
NM_001297569.2	XM_001650453.2

NM_001297592.2	XM_021856310.1
NM_001297610.2	XR_002501445.1
NM_001297643.2	XR_002501588.1
NM_001297647.2	XM_021847809.1
NM_001297648.2	XM_001654094.2
NM_001297654.2	XM_021846288.1
NM_001297664.1	XM_021854105.1
NM_001297669.3	XM_021851650.1
NM_001297710.2	XM_021853413.1
NM_001297723.2	XR_002502372.1
NM_001297724.2	XM_001663951.2
NM_001297759.2	XM_021841448.1
NM_001297774.2	XM_001654289.2
NM_001297776.2	XM_021852923.1
NM_001300756.2	XM_021847541.1
NM_001300786.2	XR_002502740.1
NM_001300787.2	XM_021846438.1
NM_001300856.2	XM_021840924.1
NM_001300859.2	XM_021846478.1
NM_001300867.2	XM_021848859.1
NM_001300885.2	XR_002500027.1
NM_001300887.2	XM_001661874.2
NM_001300898.2	XM_021857483.1
NM_001300911.2	XR_002502197.1
NM_001300919.1	XM_021850685.1
NM_001300926.2	XR_002503190.1
NM_001300938.2	XR_002502999.1
NM_001300946.3	XM_021857110.1
NM_001300960.2	XM_021846854.1
NM_001300982.2	XM_021837317.1
NM_001300990.2	XM_021839485.1
NM_001300992.2	XM_001657621.2
NM_001301021.2	XM_011494795.2
NM_001301028.3	XM_021849961.1
NM_001301031.1	XM_001659105.2
NM_001301097.1	XM_021855772.1
NM_001301134.2	XR_002500219.1
NM_001301244.2	XM_001659476.2
NM_001301253.2	XM_001658646.2
NM_001301409.2	XM_001652481.2
NM_001301645.2	XM_021839738.1
NM_001301648.2	XM_021837951.1
NM_001301775.2	XM_021853956.1
NM_001301828.2	XM_021844966.1
NM_001301830.2	XM_021842951.1
NM_001302509.2	XM_021843803.1
NM_001302554.2	XM_021850134.1
NM_001302622.2	XM_021843526.1
NM_001302688.2	XR_002500729.1
NM_001302695.2	XM_021846883.1

NM_001302817.3	XM_021843001.1
NM_001302836.2	XM_001656173.2
NM_001303000.2	XM_001653370.2
NM_001303030.2	XR_002499134.1
NM_001303037.2	XM_021841596.1
NM_001303047.2	XM_011495175.2
NM_001303235.2	XM_021857168.1
NM_001303255.3	XM_001655409.2
NM_001303276.2	XM_021854172.1
NM_001303459.3	XM_001649686.2
NM_001303475.1	XM_021848054.1
NM_001303477.2	XM_021844447.1
NM_001303487.3	XM_001655924.2
NM_001303498.3	XM_021844461.1
NM_001303615.2	XM_021842377.1
NM_001303619.2	XM_001654548.2
NM_001304331.2	XM_001656508.2
NM_001304345.2	XM_001649188.2
NM_001304360.2	XM_021849300.1
NM_001304488.1	XM_021851138.1
NM_001304489.1	XM_021856894.1
NM_001304500.2	XM_021853405.1
NM_001304507.1	XM_021848615.1
NM_001304513.2	XR_002502564.1
NM_001304549.2	XM_021845107.1
NM_001304569.2	XR_002500382.1
NM_001304763.2	XM_021852372.1
NM_001304808.3	XM_021847718.1
NM_001304814.2	XM_021851750.1
NM_001304819.2	XM_021853279.1
NM_001304834.2	XM_021837528.1
NM_001304942.2	XM_021856154.1
NM_001304947.3	XR_002500493.1
NM_001304957.2	XM_001656281.2
NM_001304993.2	XM_021854506.1
NM_001305080.2	XR_002502366.1
NM_001305108.1	XR_002500983.1
NM_001305208.2	XM_021850975.1
NM_001305457.2	XM_021854332.1
NM_001305545.1	XM_021850808.1
NM_001305625.2	XM_021841547.1
NM_001306070.2	XM_021842990.1
NM_001306092.2	XM_021838740.1
NM_001306151.2	XR_002502097.1
NM_001306160.3	XM_011494945.2
NM_001306172.2	XM_021851061.1
NM_001306197.2	XM_021838684.1
NM_001306200.2	XM_021841902.1
NM_001306214.2	XM_021844071.1
NM_001307976.2	XM_021842484.1

NM_001308023.2	XR_002502014.1
NM_001308028.2	XM_021853447.1
NM_001308075.2	XM_021848645.1
NM_001308080.2	XM_021852913.1
NM_001308097.2	XM_021850182.1
NM_001308105.1	XM_021838341.1
NM_001308119.2	XM_021856589.1
NM_001308161.1	XM_021842916.1
NM_001308169.2	XM_001660482.2
NM_001308185.2	XM_021837693.1
NM_001308203.2	XM_021843896.1
NM_001308237.2	XM_001657229.2
NM_001308242.2	XR_002501766.1
NM_001308264.2	XM_021855639.1
NM_001308342.2	XM_021843865.1
NM_001308430.2	XM_001661471.2
NM_001308469.3	XR_002498875.1
NM_001308954.2	XM_021840545.1
NM_001309193.2	XM_021851136.1
NM_001309427.2	XM_021853484.1
NM_001309450.2	XM_021855888.1
NM_001310137.3	XM_021843788.1
NM_001310160.2	XR_002499812.1
NM_001311197.2	XM_021842376.1
NM_001311311.2	XM_021852910.1
NM_001312921.2	XM_011494754.2
NM_001313741.1	XM_021844074.1
NM_001313943.2	XR_002502603.1
NM_001313953.3	XM_021851139.1
NM_001314052.2	XM_021850657.1
NM_001315.3	XM_001660731.2
NM_001316314.3	XM_021850835.1
NM_001316317.2	XR_002502679.1
NM_001316348.2	XM_021851137.1
NM_001316358.2	XM_021841339.1
NM_001316374.2	XR_002500901.1
NM_001316943.2	XM_021845506.1
NM_001316965.2	XM_001656167.2
NM_001317019.1	XM_021851824.1
NM_001317034.1	XR_002499590.1
NM_001317062.2	XM_021842563.1
NM_001317103.2	XR_002500827.1
NM_001317120.2	XM_001662114.2
NM_001317161.2	XM_021844504.1
NM_001317184.2	XR_002498975.1
NM_001317406.3	XM_021841474.1
NM_001317721.2	XM_001660709.2
NM_001317740.2	XM_001657255.3
NM_001317774.1	XR_002499599.1
NM_001317781.2	XM_021849557.1

NM_001317788.2	XM_021841123.1
NM_001317789.1	XR_002503215.1
NM_001317804.2	XM_021857050.1
NM_001317891.1	XM_021845196.1
NM_001317895.2	XM_001648213.2
NM_001317919.2	XM_021842472.1
NM_001317935.2	XM_021843007.1
NM_001317943.2	XM_001662940.2
NM_001317992.2	XM_021851965.1
NM_001318021.1	XM_001663913.2
NM_001318022.2	XM_021842475.1
NM_001318036.2	XM_001659128.2
NM_001318042.2	XM_021839379.1
NM_001318068.1	XM_001654523.2
NM_001318082.2	XM_001648138.2
NM_001318091.2	XM_001657015.2
NM_001318154.2	XM_001652433.2
NM_001318199.3	XM_021853134.1
NM_001318226.2	XM_001647622.2
NM_001318238.2	XM_021848336.1
NM_001318239.2	XR_002502621.1
NM_001318500.2	XM_021855469.1
NM_001318710.2	XM_021857141.1
NM_001318715.2	XM_001663442.2
NM_001318726.2	XM_021849829.1
NM_001318761.2	XR_002503012.1
NM_001318781.2	XM_021847329.1
NM_001318787.2	XM_011495397.2
NM_001318800.2	XM_001658372.2
NM_001318815.2	XR_002498748.1
NM_001318830.2	XM_001659903.2
NM_001318841.2	XR_002499356.1
NM_001318855.1	XM_021842564.1
NM_001318870.2	XM_001657774.2
NM_001318873.2	XM_021856900.1
NM_001318909.2	XM_021841084.1
NM_001318928.2	XM_021857108.1
NM_001318942.1	XR_002502872.1
NM_001318945.2	XM_021847086.1
NM_001319039.2	XM_021850042.1
NM_001319062.2	XM_021855047.1
NM_001319092.1	XM_001653496.2
NM_001319110.2	XM_021847802.1
NM_001319198.2	XM_021845932.1
NM_001319217.2	XM_021857071.1
NM_001319220.2	XM_021842782.1
NM_001319228.2	XM_021851967.1
NM_001319229.2	XM_021856951.1
NM_001319242.1	XM_021850097.1
NM_001319295.2	XM_021854944.1

NM_001319297.2	XM_021856715.1
NM_001319658.2	XR_002500678.1
NM_001319664.2	XM_021848764.1
NM_001319952.2	XM_021852470.1
NM_001319960.2	XM_021855633.1
NM_001319988.2	XM_001654710.2
NM_001320000.2	XM_001659028.2
NM_001320050.2	XM_001663670.2
NM_001320134.1	XM_021848707.1
NM_001320210.2	XM_011495042.2
NM_001320219.2	XM_021847031.1
NM_001320321.2	XM_021847428.1
NM_001320351.2	XM_021849998.1
NM_001320365.2	XM_001657243.3
NM_001320458.2	XM_021854057.1
NM_001320488.2	XM_021840643.1
NM_001320511.2	XM_001654262.2
NM_001320546.3	XM_021853690.1
NM_001320555.2	XM_021855041.1
NM_001320557.2	XM_021846765.1
NM_001320585.1	XR_002498800.1
NM_001320592.2	XM_011494887.2
NM_001320597.2	XM_021842405.1
NM_001320608.2	XM_021848885.1
NM_001320624.1	XM_021851287.1
NM_001320714.2	XM_021845029.1
NM_001320715.2	XM_021838512.1
NM_001320722.3	XM_001656686.2
NM_001320746.3	XM_021850775.1
NM_001320750.3	XM_021851073.1
NM_001320830.2	XM_021838010.1
NM_001320840.2	XM_021841085.1
NM_001320849.2	XM_021856541.1
NM_001320851.2	XR_002500421.1
NM_001320865.2	XM_001653939.2
NM_001320870.2	XM_001659150.2
NM_001320872.2	XM_021856892.1
NM_001320883.2	XM_021851477.1
NM_001320933.2	XM_021844287.1
NM_001320948.2	XM_021852024.1
NM_001320953.2	XM_001661599.2
NM_001320971.2	XM_001654839.2
NM_001321007.2	XM_001648211.2
NM_001321015.2	XM_001652006.2
NM_001321018.2	XR_002499649.1
NM_001321072.1	XM_021856460.1
NM_001321107.2	XM_021853424.1
NM_001321144.2	XM_021838340.1
NM_001321224.2	XM_001651325.2
NM_001321234.2	XR_002500909.1

NM_001321266.2	XM_021840091.1
NM_001321285.2	XM_001659467.2
NM_001321356.2	XM_021848040.1
NM_001321363.2	XM_021846481.1
NM_001321365.2	XM_021852505.1
NM_001321369.2	XM_021854512.1
NM_001321381.3	XR_002502935.1
NM_001321458.2	XM_001656432.2
NM_001321470.1	XM_021850938.1
NM_001321501.2	XR_002498704.1
NM_001321525.1	XM_001659473.2
NM_001321592.2	XM_021850720.1
NM_001321612.2	XR_002500160.1
NM_001321617.2	XM_021843198.1
NM_001321677.2	XR_002503261.1
NM_001321747.2	XM_001650139.2
NM_001321808.2	XR_002500018.1
NM_001321868.2	XM_021857564.1
NM_001321881.2	XM_021856331.1
NM_001321907.3	XM_001662070.2
NM_001321908.3	XM_021856975.1
NM_001321923.2	XM_021846585.1
NM_001321927.1	XM_021846993.1
NM_001321929.1	XM_021852621.1
NM_001321935.1	XM_001658594.3
NM_001321953.2	XM_021854513.1
NM_001321972.2	XR_002500077.1
NM_001321983.2	XM_001664214.2
NM_001321993.3	XM_001660375.2
NM_001322012.2	XR_002502951.1
NM_001322028.2	XR_002501124.1
NM_001322029.2	XM_021845873.1
NM_001322085.2	XM_021857088.1
NM_001322147.2	XM_021838562.1
NM_001322164.2	XM_001658061.2
NM_001322180.2	XM_021842480.1
NM_001322215.2	XM_021845190.1
NM_001322221.2	XM_021844471.1
NM_001322252.2	XM_021852397.1
NM_001322256.2	XR_002502005.1
NM_001322296.2	XM_021854818.1
NM_001322367.1	XM_021848380.1
NM_001322391.2	XM_021837265.1
NM_001322402.3	XM_021845088.1
NM_001322494.1	XM_021852447.1
NM_001322840.2	XM_021851702.1
NM_001322861.2	XM_001662825.2
NM_001322889.2	XM_021855088.1
NM_001322902.2	XM_021854300.1
NM_001322920.1	XM_001653224.2

NM_001322929.2	XM_001647730.2
NM_001322930.2	XM_021854819.1
NM_001322963.2	XM_021848231.1
NM_001322971.2	XM_021839797.1
NM_001323022.1	XR_002500313.1
NM_001323067.2	XM_021841462.1
NM_001323073.1	XM_021842938.1
NM_001323255.2	XM_021842382.1
NM_001323304.2	XM_021851536.1
NM_001323329.2	XM_021843002.1
NM_001323342.2	XM_001654503.2
NM_001323360.2	XM_001648559.2
NM_001323367.2	XM_021855119.1
NM_001323377.2	XM_021845198.1
NM_001323382.2	XM_021840727.1
NM_001323389.2	XM_021849889.1
NM_001323395.2	XM_021852028.1
NM_001323543.2	XM_001663586.2
NM_001323563.2	XM_021838195.1
NM_001323575.2	XM_021851537.1
NM_001323578.2	XR_002499262.1
NM_001323644.2	XM_021856069.1
NM_001323657.2	XM_001654882.2
NM_001323674.2	XM_001659207.2
NM_001323681.2	XM_021837858.1
NM_001323906.2	XM_001648230.3
NM_001323907.2	XM_021849187.1
NM_001323911.2	XM_021844141.1
NM_001323930.2	XR_002503226.1
NM_001323948.2	XM_021839729.1
NM_001323964.2	XM_021852067.1
NM_001323972.2	XM_021845703.1
NM_001323973.2	XM_021852546.1
NM_001324002.1	XM_001657709.2
NM_001324003.1	XM_021842311.1
NM_001324025.2	XM_021854104.1
NM_001324029.3	XM_021843255.1
NM_001324067.2	XR_002503097.1
NM_001324092.2	XM_021843501.1
NM_001324093.2	XR_002500417.1
NM_001324094.2	XM_001654213.2
NM_001324109.2	XM_021839567.1
NM_001324116.3	XM_021857437.1
NM_001324119.2	XM_001659086.2
NM_001324138.2	XM_001655261.2
NM_001324169.2	XM_021844172.1
NM_001324171.2	XR_002503134.1
NM_001324178.2	XM_001652473.2
NM_001324186.2	XM_021853067.1
NM_001324187.1	XR_002502537.1

NM_001324198.2	XM_021848762.1
NM_001324201.2	XM_021839043.1
NM_001324202.2	XM_001658100.2
NM_001324220.2	XM_021849726.1
NM_001324230.2	XM_021842388.1
NM_001324242.2	XM_021843708.1
NM_001324283.2	XM_021854553.1
NM_001324294.2	XM_021853233.1
NM_001324296.2	XM_001650039.2
NM_001324303.2	XM_001662227.2
NM_001324304.2	XM_021845034.1
NM_001324343.2	XM_021852674.1
NM_001324353.2	XM_001663607.2
NM_001324354.2	XR_002502859.1
NM_001324357.2	XM_021849648.1
NM_001324387.2	XM_021843721.1
NM_001324390.1	XM_021848536.1
NM_001324415.2	XM_021857073.1
NM_001324446.2	XM_021842596.1
NM_001326314.2	XM_021845197.1
NM_001326332.2	XR_002502965.1
NM_001328620.2	XM_021850038.1
NM_001328627.1	XM_001654943.2
NM_001328646.3	XM_021839037.1
NM_001328654.2	XM_021851900.1
NM_001328674.2	XR_002499032.1
NM_001329137.2	XM_001659994.3
NM_001329144.2	XM_021849145.1
NM_001329201.2	XR_002502238.1
NM_001329442.2	XM_001650048.2
NM_001329586.2	XM_001657486.2
NM_001329615.2	XR_002501018.1
NM_001329633.2	XR_002500411.1
NM_001329634.1	XM_021853795.1
NM_001329718.2	XM_021856887.1
NM_001329723.2	XM_021856208.1
NM_001329763.2	XM_021842085.1
NM_001329775.2	XM_001656698.2
NM_001329803.2	XM_021837992.1
NM_001329911.2	XM_001654504.2
NM_001329919.2	XM_001663991.2
NM_001329920.2	XM_021844370.1
NM_001329974.2	XM_021843502.1
NM_001330000.2	XM_001655288.2
NM_001330059.2	XM_021845822.1
NM_001330062.2	XM_021851241.1
NM_001330094.2	XM_021848400.1
NM_001330113.2	XM_001655801.2
NM_001330115.2	XM_001656755.2
NM_001330125.1	XM_021850727.1

NM_001330128.2	XM_021843649.1
NM_001330130.2	XR_002502355.1
NM_001330132.2	XM_021840371.1
NM_001330148.2	XM_021837315.1
NM_001330157.2	XM_001653262.2
NM_001330170.2	XM_001660969.2
NM_001330183.2	XM_001650004.2
NM_001330187.2	XM_021850811.1
NM_001330214.1	XM_021845007.1
NM_001330227.3	XR_002501470.1
NM_001330250.1	XM_021850773.1
NM_001330252.2	XM_021837307.1
NM_001330285.2	XM_001661936.2
NM_001330303.2	XM_021837320.1
NM_001330310.2	XM_021841675.1
NM_001330316.2	XR_002499059.1
NM_001330326.2	XM_021852711.1
NM_001330354.2	XM_021842302.1
NM_001330373.2	XM_021842308.1
NM_001330380.2	XM_021855513.1
NM_001330388.2	XM_021842303.1
NM_001330404.2	XM_021854650.1
NM_001330406.2	XM_021856721.1
NM_001330420.2	XM_021841356.1
NM_001330442.2	XM_021846281.1
NM_001330447.2	XM_001657901.2
NM_001330475.2	XM_021845560.1
NM_001330514.2	XM_021857246.1
NM_001330521.2	XR_002498699.1
NM_001330537.2	XM_021856324.1
NM_001330570.3	XM_021845933.1
NM_001330613.2	XM_021851419.1
NM_001330645.2	XM_001657197.2
NM_001330713.2	XM_021854464.1
NM_001330721.2	XR_002499859.1
NM_001330723.2	XM_021843375.1
NM_001330728.1	XM_001657341.2
NM_001330729.2	XM_021856415.1
NM_001330750.2	XM_001649107.2
NM_001330759.2	XM_021841674.1
NM_001330996.3	XM_021842470.1
NM_001331066.2	XM_021837860.1
NM_001331071.3	XM_021843024.1
NM_001331158.2	XM_001658754.2
NM_001331211.2	XM_021842228.1
NM_001344.4	XM_021853747.1
NM_001345881.2	XR_002499098.1
NM_001345882.2	XR_002503002.1
NM_001345888.2	XR_002500582.1
NM_001345909.2	XM_021844106.1

NM_001345949.3	XM_021844351.1
NM_001345977.1	XR_002499612.1
NM_001346004.2	XM_021842511.1
NM_001346070.2	XR_002501380.1
NM_001346103.2	XM_001650120.2
NM_001346116.2	XM_021846390.1
NM_001346139.1	XM_021849423.1
NM_001346223.2	XR_002498846.1
NM_001346227.2	XM_021842895.1
NM_001346229.2	XM_021850572.1
NM_001346253.2	XM_001648666.2
NM_001346254.2	XM_021852619.1
NM_001346268.2	XM_021844403.1
NM_001346311.2	XM_021843252.1
NM_001346341.2	XM_021847805.1
NM_001346342.2	XM_021840604.1
NM_001346353.2	XM_001658075.2
NM_001346382.2	XM_021851428.1
NM_001346404.1	XM_021854339.1
NM_001346413.2	XM_021851812.1
NM_001346450.2	XM_021845875.1
NM_001346457.2	XR_002498602.1
NM_001346474.2	XM_001657900.2
NM_001346477.2	XM_021852029.1
NM_001346482.1	XM_021843254.1
NM_001346543.2	XR_002502356.1
NM_001346548.2	XM_021840011.1
NM_001346549.2	XM_001649597.2
NM_001346560.3	XM_021843210.1
NM_001346603.2	XM_021852579.1
NM_001346719.2	XM_001652301.2
NM_001346818.1	XM_021844986.1
NM_001346862.2	XM_011495227.2
NM_001346932.2	XM_021844769.1
NM_001346934.2	XM_021852309.1
NM_001346946.2	XR_002499982.1
NM_001346955.2	XM_001650094.2
NM_001346972.2	XM_021845701.1
NM_001347015.2	XM_021851214.1
NM_001347018.2	XM_021841599.1
NM_001347031.2	XM_021844671.1
NM_001347096.2	XM_001653925.2
NM_001347219.2	XM_001655139.2
NM_001347664.1	XM_021857247.1
NM_001347703.2	XM_021840823.1
NM_001347738.2	XM_001651818.2
NM_001347828.2	XR_002502343.1
NM_001347849.2	XM_021843650.1
NM_001347864.2	XM_001650929.3
NM_001347893.2	XM_021852031.1

NM_001347895.2	XM_021843646.1
NM_001347898.2	XR_002501948.1
NM_001347946.2	XM_021839581.1
NM_001347947.2	XM_001656413.2
NM_001347967.2	XM_001652696.2
NM_001347997.2	XR_002499715.1
NM_001348013.2	XM_001656592.2
NM_001348129.2	XM_021847359.1
NM_001348136.2	XM_021843161.1
NM_001348144.2	XM_021856693.1
NM_001348189.2	XM_021844291.1
NM_001348203.1	XM_021842943.1
NM_001348233.2	XM_021857032.1
NM_001348239.2	XR_002499757.1
NM_001348300.1	XM_021848063.1
NM_001348304.1	XM_001653085.2
NM_001348322.1	XM_021839829.1
NM_001348323.3	XM_001651837.2
NM_001348372.2	XR_002502833.1
NM_001348397.2	XR_002499499.1
NM_001348424.1	XM_021846862.1
NM_001348460.2	XM_021856757.1
NM_001348466.2	XM_001656961.2
NM_001348509.2	XM_021839728.1
NM_001348542.2	XM_021839727.1
NM_001348547.2	XM_021857165.1
NM_001348702.2	XM_001653703.2
NM_001348735.2	XM_021842616.1
NM_001348756.2	XR_002499417.1
NM_001348764.2	XM_021846792.1
NM_001348768.2	XM_021848486.1
NM_001348781.2	XM_021843342.1
NM_001348986.1	XM_021850742.1
NM_001348988.1	XM_001654139.2
NM_001349012.1	XR_002502715.1
NM_001349041.2	XM_021853864.1
NM_001349044.2	XM_021842723.1
NM_001349099.2	XM_001648710.2
NM_001349104.2	XM_001659763.2
NM_001349112.3	XM_001648209.2
NM_001349149.2	XM_021854061.1
NM_001349157.2	XM_001649791.2
NM_001349158.2	XM_021843315.1
NM_001349168.2	XM_021842724.1
NM_001349191.2	XM_021844149.1
NM_001349193.2	XM_021854559.1
NM_001349222.2	XM_001660997.2
NM_001349230.2	XM_001653828.2
NM_001349242.2	XM_021844278.1
NM_001349258.2	XM_001651312.2

NM_001349261.2	XM_021853810.1
NM_001349263.2	XM_021839546.1
NM_001349297.2	XM_021856614.1
NM_001349312.2	XM_021846109.1
NM_001349320.2	XM_021842324.1
NM_001349350.2	XM_021851746.1
NM_001349359.2	XM_021842725.1
NM_001349362.2	XM_021840889.1
NM_001349378.2	XM_001648797.2
NM_001349379.2	XM_001651301.2
NM_001349383.1	XM_021847999.1
NM_001349385.1	XM_021838774.1
NM_001349390.2	XM_001662630.2
NM_001349394.2	XM_021855278.1
NM_001349395.2	XM_021848072.1
NM_001349402.2	XM_021853615.1
NM_001349413.1	XM_021849164.1
NM_001349450.1	XM_021842727.1
NM_001349457.2	XM_001662758.2
NM_001349460.2	XM_001655850.2
NM_001349464.2	XM_021844275.1
NM_001349476.1	XR_002502130.1
NM_001349479.1	XM_021847370.1
NM_001349498.1	XM_021844050.1
NM_001349551.2	XM_021847679.1
NM_001349623.1	XM_021843112.1
NM_001349635.1	XM_021844640.1
NM_001349661.2	XM_021851429.1
NM_001349711.2	XM_021838057.1
NM_001349712.2	XR_002502017.1
NM_001349735.2	XR_002501375.1
NM_001349743.2	XR_002499925.1
NM_001349776.2	XM_021837308.1
NM_001349803.3	XM_021842290.1
NM_001349853.2	XR_002498709.1
NM_001349866.2	XM_021852382.1
NM_001349921.2	XM_021842528.1
NM_001349935.2	XM_001659672.2
NM_001349969.2	XM_021839630.1
NM_001349990.2	XM_021850740.1
NM_001349991.2	XM_021837353.1
NM_001350017.2	XM_021849531.1
NM_001350022.2	XM_001658963.2
NM_001350043.2	XM_021850802.1
NM_001350044.2	XM_021839719.1
NM_001350115.2	XM_021855951.1
NM_001350123.2	XM_001650904.2
NM_001350234.2	XM_021850353.1
NM_001350251.2	XM_021838545.1
NM_001350262.2	XM_021844876.1

NM_001350303.2	XM_001663217.2
NM_001350310.2	XM_021851529.1
NM_001350327.2	XM_021839430.1
NM_001350331.2	XM_011495002.2
NM_001350394.2	XM_021848907.1
NM_001350398.2	XM_021844996.1
NM_001350399.2	XM_021839717.1
NM_001350402.1	XM_021844659.1
NM_001350442.2	XM_001657927.2
NM_001350446.2	XM_021852355.1
NM_001350456.2	XM_021848053.1
NM_001350506.2	XM_001650110.3
NM_001350514.2	XM_001651706.3
NM_001350565.2	XM_001654953.2
NM_001350570.2	XM_001650995.2
NM_001350581.2	XM_001648775.2
NM_001350602.2	XM_021852904.1
NM_001350614.2	XM_001660506.2
NM_001350629.2	XM_021856308.1
NM_001350635.3	XM_021848700.1
NM_001350645.2	XM_001655624.2
NM_001350681.1	XR_002500903.1
NM_001350714.2	XM_021851427.1
NM_001350745.2	XM_021843220.1
NM_001350769.2	XM_021843031.1
NM_001350781.1	XM_001655171.2
NM_001350829.2	XM_001650404.2
NM_001350894.1	XM_021844617.1
NM_001350898.2	XM_021854336.1
NM_001350913.2	XM_021853926.1
NM_001350922.2	XM_021846189.1
NM_001350936.2	XM_021838972.1
NM_001350955.2	XR_002501675.1
NM_001351023.2	XM_001660792.2
NM_001351044.2	XM_001658809.2
NM_001351059.2	XM_001654174.2
NM_001351068.2	XM_021842500.1
NM_001351076.2	XM_001660501.2
NM_001351087.2	XM_021842086.1
NM_001351100.2	XR_002502162.1
NM_001351114.2	XM_001660509.2
NM_001351123.2	XM_001653591.2
NM_001351127.2	XM_021842707.1
NM_001351136.2	XM_021845146.1
NM_001351140.2	XM_001654018.2
NM_001351149.2	XM_021842469.1
NM_001351163.2	XM_021841422.1
NM_001351222.2	XR_002501932.1
NM_001351242.2	XM_021850906.1
NM_001351243.2	XM_021843011.1

NM_001351263.2	XM_021845065.1
NM_001351266.2	XM_021842986.1
NM_001351271.2	XM_021839792.1
NM_001351293.2	XR_002501991.1
NM_001351302.3	XM_001654417.2
NM_001351336.2	XR_002502530.1
NM_001351357.2	XR_002500622.1
NM_001351367.2	XM_001651675.2
NM_001351392.2	XM_001663531.2
NM_001351408.2	XM_021840373.1
NM_001351418.2	XM_021845649.1
NM_001351462.2	XR_002503160.1
NM_001351488.2	XM_021837733.1
NM_001351509.2	XM_021837340.1
NM_001351521.2	XM_021841100.1
NM_001351531.2	XM_021845564.1
NM_001351532.2	XM_021846197.1
NM_001351568.3	XM_021856145.1
NM_001351587.2	XM_021839722.1
NM_001351589.2	XM_021844492.1
NM_001351609.2	XM_021854130.1
NM_001351615.2	XR_002502867.1
NM_001351616.1	XM_021838203.1
NM_001351624.2	XM_021838756.1
NM_001351644.2	XM_021842087.1
NM_001351835.2	XR_002502511.1
NM_001351942.2	XM_021850020.1
NM_001351959.2	XR_002500813.1
NM_001351970.2	XM_021845856.1
NM_001352027.3	XM_021838614.1
NM_001352053.2	XM_021847406.1
NM_001352057.2	XR_002500090.1
NM_001352081.2	XM_021854505.1
NM_001352098.2	XR_002500900.1
NM_001352102.2	XM_021848796.1
NM_001352106.2	XM_021838895.1
NM_001352108.2	XM_021844406.1
NM_001352139.2	XM_011495019.2
NM_001352161.2	XM_001652122.2
NM_001352162.2	XM_001655974.2
NM_001352173.2	XM_001656494.2
NM_001352210.2	XM_021837953.1
NM_001352242.2	XM_011495049.2
NM_001352308.2	XM_001653694.2
NM_001352411.2	XM_021843247.1
NM_001352442.2	XM_001653285.2
NM_001352498.2	XM_001653189.2
NM_001352516.2	XM_021847497.1
NM_001352517.1	XM_021849736.1
NM_001352529.2	XM_021842936.1

NM_001352539.2	XM_021851333.1
NM_001352542.2	XR_002499379.1
NM_001352615.2	XM_021842855.1
NM_001352617.2	XM_001657204.2
NM_001352618.2	XM_021845321.1
NM_001352670.2	XM_001662842.2
NM_001352671.2	XM_021844444.1
NM_001352684.2	XR_002501420.1
NM_001352695.2	XM_001654505.2
NM_001352718.2	XR_002500176.1
NM_001352750.2	XR_002501413.1
NM_001352764.2	XM_021843119.1
NM_001352825.2	XM_021856178.1
NM_001352826.2	XM_001648139.3
NM_001352849.2	XM_021843846.1
NM_001352881.1	XM_021848305.1
NM_001352910.2	XR_002502364.1
NM_001352912.1	XM_021839040.1
NM_001352934.1	XM_021857729.1
NM_001352949.1	XR_002502761.1
NM_001352961.1	XM_021841598.1
NM_001352988.2	XM_021851071.1
NM_001352995.2	XM_001654153.2
NM_001353006.2	XM_021851467.1
NM_001353011.2	XM_001648229.2
NM_001353018.2	XM_021843335.1
NM_001353023.2	XM_021852335.1
NM_001353064.2	XM_001647784.2
NM_001353075.1	XM_021853568.1
NM_001353098.2	XM_001663447.2
NM_001353124.2	XM_021853046.1
NM_001353154.1	XM_001656742.2
NM_001353207.1	XM_001655673.2
NM_001353222.2	XR_002500817.1
NM_001353229.2	XM_021842848.1
NM_001353232.1	XM_021847117.1
NM_001353237.1	XM_021839944.1
NM_001353248.2	XM_001651300.2
NM_001353301.2	XM_021852310.1
NM_001353302.1	XR_002500770.1
NM_001353313.2	XM_001656603.2
NM_001353320.2	XM_021856241.1
NM_001353340.1	XM_021855736.1
NM_001353486.2	XM_001663333.2
NM_001353533.2	XR_002498915.1
NM_001353551.1	XM_021847768.1
NM_001353602.2	XR_002498898.1
NM_001353658.2	XM_021843123.1
NM_001353666.2	XM_001649999.2
NM_001353676.2	XM_021850796.1

NM_001353695.2	XM_001656215.2
NM_001353715.2	XM_021847649.1
NM_001353717.2	XR_002502288.1
NM_001353734.2	XM_001657501.3
NM_001353743.2	XM_021851470.1
NM_001353776.2	XM_021848767.1
NM_001353780.2	XM_001649372.2
NM_001353824.2	XM_021856618.1
NM_001353827.2	XM_021843454.1
NM_001353829.2	XM_021839025.1
NM_001353837.2	XM_001657842.2
NM_001353881.2	XM_021855269.1
NM_001353904.1	XM_001656094.2
NM_001353924.2	XM_021850515.1
NM_001353951.2	XM_021844301.1
NM_001353985.2	XM_021844256.1
NM_001353994.1	XM_021848456.1
NM_001354001.3	XM_001652868.2
NM_001354011.2	XM_021843569.1
NM_001354024.2	XM_021851622.1
NM_001354047.1	XM_021846486.1
NM_001354096.2	XM_021841646.1
NM_001354173.2	XR_002500681.1
NM_001354260.2	XM_001660941.2
NM_001354300.2	XM_021854252.1
NM_001354315.2	XM_001653648.2
NM_001354346.2	XM_021851990.1
NM_001354392.2	XM_021854761.1
NM_001354397.2	XM_021852005.1
NM_001354398.2	XM_021843595.1
NM_001354423.2	XM_021840022.1
NM_001354424.2	XM_021838209.1
NM_001354427.2	XM_021843319.1
NM_001354460.2	XM_001650621.2
NM_001354473.2	XR_002500853.1
NM_001354476.2	XM_021843051.1
NM_001354567.2	XM_021843524.1
NM_001354579.2	XM_021840602.1
NM_001354608.2	XR_002499630.1
NM_001354619.2	XM_021842728.1
NM_001354640.2	XM_021843862.1
NM_001354690.3	XM_021838760.1
NM_001354693.3	XR_002498926.1
NM_001354698.2	XM_021846296.1
NM_001354712.2	XM_021843857.1
NM_001354727.2	XM_001652893.2
NM_001354751.2	XM_021839540.1
NM_001354779.2	XM_001663017.2
NM_001354817.1	XM_001656310.2
NM_001354932.2	XR_002500946.1

NM_001354934.2	XM_021849153.1
NM_001354974.2	XM_001656130.2
NM_001355001.2	XR_002502329.1
NM_001355006.2	XM_001658603.2
NM_001355276.2	XM_021851324.1
NM_001355278.1	XM_021846515.1
NM_001355465.2	XM_021844428.1
NM_001356372.2	XM_021843353.1
NM_001357943.2	XR_002499142.1
NM_001358263.1	XM_021851188.1
NM_001358345.2	XM_021852478.1
NM_001359651.2	XM_021849659.1
NM_001361.5	XM_021851466.1
NM_001362481.2	XM_021853750.1
NM_001362777.2	XM_001651903.2
NM_001362787.2	XM_021853047.1
NM_001362848.1	XM_021839668.1
NM_001362872.2	XM_021851233.1
NM_001362873.2	XM_001661089.2
NM_001362926.2	XM_001652894.2
NM_001362965.2	XM_001650765.3
NM_001362988.2	XM_021843053.1
NM_001363041.2	XM_021852605.1
NM_001363046.2	XR_002499338.1
NM_001363083.1	XM_021856965.1
NM_001363104.2	XM_021852623.1
NM_001363138.1	XM_021852628.1
NM_001363343.2	XM_021846024.1
NM_001363345.2	XM_021850774.1
NM_001363366.1	XR_002502946.1
NM_001363413.2	XM_021839502.1
NM_001363447.2	XM_021853797.1
NM_001363460.1	XM_021844277.1
NM_001363514.2	XM_001658576.2
NM_001363516.2	XM_001650095.2
NM_001363518.2	XM_021849715.1
NM_001363522.2	XM_001654272.2
NM_001363539.2	XM_021844412.1
NM_001363543.2	XM_021844187.1
NM_001363578.1	XM_021855734.1
NM_001363651.3	XM_021838001.1
NM_001363655.2	XM_021841703.1
NM_001363657.3	XM_001661492.2
NM_001363699.2	XM_001656055.2
NM_001363707.2	XM_001659438.2
NM_001363737.2	XM_021853786.1
NM_001363766.1	XM_021843571.1
NM_001363794.2	XM_021848107.1
NM_001363802.1	XR_002502668.1
NM_001363816.2	XR_002498912.1

NM_001363875.2	XR_002501626.1
NM_001363900.1	XM_021843452.1
NM_001363914.1	XM_001651230.2
NM_001363916.1	XM_021846293.1
NM_001363964.1	XM_021846105.1
NM_001363988.1	XM_001653686.2
NM_001364021.1	XM_001648644.2
NM_001364084.2	XM_001654240.2
NM_001364159.2	XR_002498828.1
NM_001364228.2	XM_021857688.1
NM_001364245.2	XM_001657215.2
NM_001364255.2	XM_021846492.1
NM_001364393.2	XM_001662049.3
NM_001364486.2	XM_021850339.1
NM_001364520.2	XM_021844533.1
NM_001364573.2	XM_021852185.1
NM_001364681.2	XR_002501627.1
NM_001364682.1	XM_021846493.1
NM_001364818.2	XM_021844616.1
NM_001365125.2	XM_001650949.2
NM_001365156.1	XM_021847383.1
NM_001365202.1	XM_021844485.1
NM_001365203.1	XM_021845596.1
NM_001365204.1	XR_002500056.1
NM_001365208.1	XM_021844833.1
NM_001365250.1	XM_021850039.1
NM_001365252.1	XM_021838151.1
NM_001365264.1	XM_021839656.1
NM_001365373.1	XM_021845473.1
NM_001365408.1	XM_001656781.2
NM_001365496.2	XR_002499005.1
NM_001365520.2	XM_021847364.1
NM_001365598.3	XM_021853553.1
NM_001365603.3	XM_021855479.1
NM_001365634.1	XM_021854471.1
NM_001365713.1	XM_021842759.1
NM_001365717.1	XM_001656935.2
NM_001365743.2	XM_021856639.1
NM_001365768.2	XM_001650920.2
NM_001365774.2	XM_021846760.1
NM_001365779.1	XM_021846556.1
NM_001365805.1	XM_021856484.1
NM_001365835.2	XM_021842632.1
NM_001365837.1	XR_002499775.1
NM_001365855.1	XM_021850923.1
NM_001365891.2	XM_021842761.1
NM_001365902.3	XM_001650881.2
NM_001365951.3	XM_021856491.1
NM_001366086.1	XR_002500464.1
NM_001366088.2	XR_002499596.1

NM_001366213.1	XR_002501022.1
NM_001366253.2	XM_021849692.1
NM_001366283.2	XM_001655351.2
NM_001366361.1	XM_001657307.2
NM_001366371.2	XM_021837711.1
NM_001366473.2	XM_021856227.1
NM_001366479.2	XM_001648306.2
NM_001366501.2	XM_021849766.1
NM_001366532.1	XM_021845073.1
NM_001366545.2	XM_001661314.2
NM_001366556.2	XM_001664108.2
NM_001366570.1	XM_021850539.1
NM_001366583.1	XR_002500249.1
NM_001366586.1	XM_001662998.2
NM_001366618.1	XM_021843256.1
NM_001366682.2	XR_002501143.1
NM_001366686.3	XM_011494703.2
NM_001366779.1	XM_021839027.1
NM_001366803.2	XM_021849481.1
NM_001366993.1	XR_002502386.1
NM_001367064.1	XM_021852087.1
NM_001367360.2	XM_021854102.1
NM_001367404.1	XM_021850677.1
NM_001367424.1	XM_021840853.1
NM_001367460.1	XM_021841072.1
NM_001367461.1	XM_021843908.1
NM_001367462.1	XR_002500084.1
NM_001367510.1	XM_001648408.2
NM_001367517.1	XM_001654349.2
NM_001367561.1	XM_001653701.2
NM_001367569.1	XM_021850748.1
NM_001367570.1	XM_001663625.2
NM_001367600.2	XM_001661587.2
NM_001367645.1	XM_001659844.2
NM_001367651.1	XM_021844369.1
NM_001367691.1	XM_021851347.1
NM_001367737.1	XM_001652340.2
NM_001367747.1	XR_002499400.1
NM_001367753.1	XR_002500236.1
NM_001367791.1	XM_021848037.1
NM_001367797.1	XM_021850734.1
NM_001367810.1	XM_001648803.2
NM_001367842.1	XR_002502653.1
NM_001367850.1	XM_021843253.1
NM_001367851.1	XM_021842915.1
NM_001367856.1	XM_021854321.1
NM_001367860.1	XM_001662654.2
NM_001367872.1	XM_021855622.1
NM_001367873.1	XM_021843677.1
NM_001367895.1	XM_021850701.1

NM_001367897.1	XR_002502415.1
NM_001367904.1	XM_021837318.1
NM_001367915.1	XM_001651037.2
NM_001367968.1	XM_001653891.2
NM_001367975.1	XM_001663743.2
NM_001368066.1	XM_021846871.1
NM_001368275.1	XM_021843695.1
NM_001368324.2	XM_021852433.1
NM_001368888.2	XR_002500643.1
NM_001368900.2	XR_002503178.1
NM_001368906.2	XM_021838793.1
NM_001368921.2	XM_021845858.1
NM_001369044.1	XM_021853769.1
NM_001369110.1	XR_002501817.1
NM_001369256.1	XM_021856662.1
NM_001369401.1	XM_021838013.1
NM_001369406.2	XM_001648419.2
NM_001369468.1	XM_021852386.1
NM_001369486.1	XM_021850063.1
NM_001369495.1	XM_021839609.1
NM_001369512.1	XM_001662350.2
NM_001369523.1	XM_021841425.1
NM_001369542.1	XR_002501512.1
NM_001369551.1	XM_021850209.1
NM_001369578.1	XM_021846436.1
NM_001369579.1	XM_001655075.3
NM_001369580.1	XM_021845860.1
NM_001369583.1	XM_021850362.1
NM_001369600.1	XM_001648795.2
NM_001369639.1	XM_001661594.3
NM_001369644.1	XR_002501983.1
NM_001369672.1	XM_021842880.1
NM_001369703.1	XM_021839802.1
NM_001369788.1	XM_001657539.2
NM_001369799.1	XM_021843387.1
NM_001369821.1	XM_001650334.2
NM_001369864.1	XM_001660596.2
NM_001369917.1	XM_001658114.2
NM_001369920.1	XM_021844102.1
NM_001369930.1	XM_021849237.1
NM_001370076.1	XM_021846020.1
NM_001370085.1	XM_001655165.2
NM_001370104.2	XM_021848197.1
NM_001370113.2	XM_021839563.1
NM_001370126.1	XM_021848102.1
NM_001370136.1	XM_021838446.1
NM_001370153.1	XM_021844449.1
NM_001370167.1	XR_002498845.1
NM_001370202.1	XM_021852647.1
NM_001370221.1	XM_021845637.1

NM_001370237.1	XM_021854579.1
NM_001370260.1	XM_001654324.2
NM_001370261.1	XM_001654170.2
NM_001370271.1	XM_021849228.1
NM_001370317.1	XM_021847650.1
NM_001370348.2	XM_021853273.1
NM_001370367.1	XM_021845643.1
NM_001370403.1	XM_001651395.2
NM_001370426.1	XM_001650877.2
NM_001370432.1	XM_001658309.2
NM_001370461.1	XM_001654871.2
NM_001370467.1	XR_002500145.1
NM_001370501.1	XM_021852648.1
NM_001370532.1	XM_001652650.2
NM_001370533.1	XM_021839795.1
NM_001370554.1	XM_001662670.2
NM_001370567.1	XM_021845138.1
NM_001370588.1	XM_021847268.1
NM_001370608.1	XM_021843172.1
NM_001370639.1	XR_002499865.1
NM_001370649.1	XM_021837331.1
NM_001370652.1	XM_021850056.1
NM_001370687.1	XM_021839188.1
NM_001371009.1	XM_021845647.1
NM_001371075.1	XR_002502440.1
NM_001371128.1	XM_001654718.2
NM_001371137.1	XM_021849229.1
NM_001371138.1	XM_021852105.1
NM_001371168.1	XM_001656037.2
NM_001371206.1	XM_021839584.1
NM_001371210.1	XM_021842062.1
NM_001371215.1	XR_002502634.1
NM_001371216.1	XM_021857428.1
NM_001371217.1	XR_002501222.1
NM_001371237.1	XM_001662175.2
NM_001371251.1	XM_001655598.2
NM_001371328.1	XR_002500067.1
NM_001371332.1	XM_021849473.1
NM_001371333.1	XM_001653265.2
NM_001371335.1	XM_021848848.1
NM_001371350.1	XM_021847029.1
NM_001371355.1	XM_021844936.1
NM_001371362.1	XM_001651704.2
NM_001371389.2	XM_021857206.1
NM_001371410.1	XM_001663709.2
NM_001371427.1	XM_021850629.1
NM_001371445.1	XM_001655806.2
NM_001371452.1	XM_021843386.1
NM_001371589.1	XM_021849243.1
NM_001371592.1	XM_021845570.1

NM_001371740.1	XR_002501674.1
NM_001371774.1	XR_002499206.1
NM_001371858.3	XM_021852203.1
NM_001371923.1	XM_001650831.2
NM_001371936.1	XM_021854438.1
NM_001372024.1	XM_021853567.1
NM_001372025.1	XM_001648677.3
NM_001372070.1	XM_021854519.1
NM_001372080.1	XM_001663110.2
NM_001372167.1	XM_021838447.1
NM_001372189.1	XM_001660365.2
NM_001374256.1	XM_021837943.1
NM_001374264.2	XM_001649766.2
NM_001374269.1	XM_021846715.1
NM_001374357.1	XM_001652321.2
NM_001374523.1	XM_021851570.1
NM_001374596.1	XR_002502950.1
NM_001374781.1	XM_021845661.1
NM_001374824.1	XM_021854627.1
NM_001374847.1	XM_021843465.1
NM_001374868.1	XR_002499086.1
NM_001375286.1	XM_021845620.1
NM_001375291.1	XM_021850389.1
NM_001375296.1	XM_021857204.1
NM_001375299.1	XM_001661561.2
NM_001375321.1	XM_021843986.1
NM_001375330.1	XR_002498802.1
NM_001375334.1	XM_021840130.1
NM_001375353.1	XR_002502827.1
NM_001375360.1	XM_021843373.1
NM_001375411.1	XR_002499265.1
NM_001375444.1	XM_001654388.2
NM_001375463.1	XR_002498914.1
NM_001375467.1	XM_001648892.3
NM_001375468.1	XM_021848560.1
NM_001375477.1	XM_021848031.1
NM_001375491.1	XM_001655100.3
NM_001375492.1	XM_021850891.1
NM_001375554.1	XM_021843618.1
NM_001375575.1	XM_001658031.2
NM_001375583.1	XM_021844279.1
NM_001375585.1	XM_021852679.1
NM_001375589.2	XM_001660720.2
NM_001375618.1	XM_001659751.2
NM_001375630.1	XM_001648227.2
NM_001375651.1	XM_021856517.1
NM_001375655.1	XM_021846590.1
NM_001375673.1	XM_001663136.2
NM_001375697.1	XM_021850442.1
NM_001375705.1	XR_002500702.1

NM_001375720.1	XM_021851346.1
NM_001375725.1	XM_001649342.2
NM_001375741.1	XM_021849762.1
NM_001375756.1	XM_001651334.2
NM_001375780.1	XR_002500555.1
NM_001375845.1	XM_001648782.2
NM_001375897.1	XM_001654325.2
NM_001375908.1	XM_021853373.1
NM_001375939.1	XM_021843336.1
NM_001375968.1	XM_001659499.2
NM_001376068.1	XM_021847145.1
NM_001376085.1	XR_002500039.1
NM_001376112.1	XM_021847010.1
NM_001376119.1	XM_011495451.2
NM_001376131.1	XM_001655795.2
NM_001376132.1	XM_021843453.1
NM_001376150.1	XM_021856870.1
NM_001376153.1	XM_001652759.2
NM_001376157.1	XM_021856852.1
NM_001376161.1	XM_021843693.1
NM_001376248.1	XM_021845124.1
NM_001376251.1	XM_021848744.1
NM_001376253.1	XM_021840576.1
NM_001376323.2	XM_021848252.1
NM_001376342.1	XM_021838107.1
NM_001376351.1	XM_001660504.2
NM_001376354.1	XM_021844704.1
NM_001376364.1	XR_002501247.1
NM_001376379.1	XR_002501660.1
NM_001376443.1	XR_002500388.1
NM_001376446.1	XM_021848610.1
NM_001376450.1	XM_021849257.1
NM_001376483.1	XM_021850383.1
NM_001376523.1	XR_002501014.1
NM_001376529.1	XM_001649558.2
NM_001376543.1	XR_002503139.1
NM_001376580.1	XM_021848704.1
NM_001376591.1	XM_021837390.1
NM_001376616.1	XM_021843756.1
NM_001376619.1	XM_021855577.1
NM_001376625.1	XR_002499947.1
NM_001376639.1	XM_021839197.1
NM_001376654.1	XR_002502770.1
NM_001376682.1	XM_021844427.1
NM_001376690.1	XM_021852256.1
NM_001376696.1	XM_021839593.1
NM_001376725.1	XM_021843839.1
NM_001376726.1	XM_021840825.1
NM_001376728.1	XM_001648038.3
NM_001376783.1	XM_021850635.1

NM_001376793.1	XM_001652352.2
NM_001376817.1	XM_021848030.1
NM_001376821.1	XM_021856853.1
NM_001376844.1	XM_021847998.1
NM_001376887.1	XR_002500317.1
NM_001376932.1	XM_021843326.1
NM_001377062.1	XR_002499339.1
NM_001377129.1	XM_021842066.1
NM_001377130.1	XM_001662711.2
NM_001377173.1	XM_001652497.2
NM_001377174.1	XM_001653655.2
NM_001377178.1	XM_021846386.1
NM_001377198.1	XM_021841419.1
NM_001377203.1	XM_001662420.2
NM_001377204.1	XM_001658588.2
NM_001377208.1	XM_001659727.2
NM_001377212.1	XM_001649902.2
NM_001377228.1	XM_021848253.1
NM_001377238.1	XM_001648586.2
NM_001377291.1	XM_001652734.2
NM_001377317.2	XM_021837654.1
NM_001377358.1	XM_001659414.2
NM_001377362.1	XM_001652002.2
NM_001377377.1	XR_002500921.1
NM_001377379.1	XR_002503191.1
NM_001377394.1	XM_021847972.1
NM_001377405.1	XM_021844177.1
NM_001377408.1	XM_001651067.2
NM_001377411.1	XM_001651278.2
NM_001377418.1	XM_021844350.1
NM_001377427.1	XM_001659705.2
NM_001377470.1	XM_001659534.2
NM_001377491.1	XM_021849191.1
NM_001377492.1	XM_021849226.1
NM_001377496.1	XR_002498596.1
NM_001377931.1	XR_002503221.1
NM_001377961.1	XM_021839381.1
NM_001377970.1	XM_001659407.2
NM_001377974.1	XM_001659597.2
NM_001378029.1	XM_021851874.1
NM_001378034.1	XM_001659623.2
NM_001378074.1	XM_021842607.1
NM_001378077.1	XM_021844432.1
NM_001378092.1	XM_021846584.1
NM_001378094.1	XM_021838159.1
NM_001378102.1	XM_021839947.1
NM_001378107.1	XM_021846615.1
NM_001378132.1	XM_021844818.1
NM_001378144.1	XM_021854439.1
NM_001378152.1	XM_021840035.1

NM_001378157.1	XM_001659263.2
NM_001378178.1	XM_021851787.1
NM_001378181.1	XM_021848237.1
NM_001378254.1	XM_021851747.1
NM_001378257.1	XR_002502338.1
NM_001378270.1	XM_021847213.1
NM_001378408.1	XM_021855655.1
NM_001378456.1	XM_021840906.1
NM_001378485.1	XR_002498653.1
NM_001378486.1	XR_002499682.1
NM_001378533.1	XM_021848562.1
NM_001378538.1	XM_021854763.1
NM_001378546.1	XM_021838015.1
NM_001378851.1	XM_001651595.2
NM_001379104.1	XR_002500928.1
NM_001379331.1	XM_021849474.1
NM_001379461.1	XM_021850567.1
NM_001379515.1	XM_001649607.2
NM_001379551.1	XM_001651347.2
NM_001379566.1	XR_002499839.1
NM_001379610.1	XM_021853753.1
NM_001379614.1	XM_021850839.1
NM_001381865.2	XM_021845566.1
NM_001381886.1	XR_002498655.1
NM_001381939.1	XM_021844174.1
NM_001381941.1	XM_021854798.1
NM_001381949.1	XR_002498677.1
NM_001382242.1	XR_002502961.1
NM_001382287.1	XM_021843352.1
NM_001382288.1	XM_021854799.1
NM_001382310.1	XM_001651499.2
NM_001382339.1	XM_021844803.1
NM_001382396.1	XM_001654171.2
NM_001382408.1	XM_021840590.1
NM_001382424.1	XM_021850233.1
NM_001382429.1	XM_001656688.2
NM_001382467.1	XM_021850680.1
NM_001382470.1	XR_002503204.1
NM_001382527.1	XM_001663502.2
NM_001382528.1	XM_021854444.1
NM_001382554.1	XM_021855572.1
NM_001382573.1	XR_002503177.1
NM_001382611.1	XM_001653206.2
NM_001382616.1	XM_021857284.1
NM_001382619.1	XM_021855728.1
NM_001382628.1	XM_021852373.1
NM_001382634.1	XM_021855018.1
NM_001382639.1	XM_021841708.1
NM_001382645.1	XM_021839504.1
NM_001382652.1	XM_021849392.1

NM_001382674.1	XM_001654441.2
NM_001382696.1	XM_021850385.1
NM_001382707.1	XM_021839246.1
NM_001382722.1	XM_021843173.1
NM_001382730.1	XR_002499063.1
NM_001382740.1	XM_001653663.2
NM_001382767.1	XM_001660717.2
NM_001382787.1	XM_021842604.1
NM_001384133.1	XM_021844614.1
NM_001384268.1	XM_021852516.1
NM_001384339.1	XM_021838841.1
NM_001384350.1	XM_021843356.1
NM_001384356.1	XM_021847118.1
NM_001384369.1	XM_001654856.2
NM_001384387.1	XM_021842051.1
NM_001384390.1	XM_001656810.2
NM_001384401.1	XM_021843234.1
NM_001384404.1	XM_001653623.3
NM_001384414.1	XR_002501132.1
NM_001384420.1	XM_021851901.1
NM_001384445.1	XR_002501148.1
NM_001384459.1	XM_001653684.2
NM_001384464.1	XR_002502133.1
NM_001384488.1	XM_021851810.1
NM_001384508.1	XR_002499449.1
NM_001384513.1	XM_001655688.2
NM_001384526.1	XM_021842760.1
NM_001384534.1	XM_021843694.1
NM_001384537.1	XM_021842021.1
NM_001384538.1	XR_002503163.1
NM_001384554.1	XM_001660818.2
NM_001384572.1	XM_001651802.3
NM_001384576.1	XM_021851876.1
NM_001384582.1	XM_021849161.1
NM_001384682.1	XM_001660729.2
NM_001384704.1	XM_021849343.1
NM_001384707.1	XR_002501137.1
NM_001384708.1	XM_021845005.1
NM_001384713.1	XM_001655480.2
NM_001384734.1	XR_002502748.1
NM_001384745.1	XM_001660649.2
NM_001384778.1	XM_021850395.1
NM_001384809.1	XR_002500984.1
NM_001384851.1	XM_001658593.3
NM_001384866.1	XM_001657780.2
NM_001384867.1	XM_021847435.1
NM_001384882.1	XM_021840391.1
NM_001384892.1	XM_021845253.1
NM_001384898.1	XM_021853833.1
NM_001384900.1	XR_002501215.1

NM_001384936.1	XM_021852649.1
NM_001384988.1	XM_021848032.1
NM_001384993.1	XM_021850426.1
NM_001384995.1	XM_001660837.2
NM_001385010.1	XM_021843475.1
NM_001385017.1	XR_002499787.1
NM_001385028.1	XM_021837825.1
NM_001385029.1	XM_001648655.2
NM_001385072.1	XM_021842100.1
NM_001385084.1	XR_002502470.1
NM_001385086.1	XM_021840732.1
NM_001385093.1	XM_021842902.1
NM_001385110.1	XM_001661245.2
NM_001385111.1	XM_021855180.1
NM_001385116.1	XR_002503216.1
NM_001385146.1	XM_021852476.1
NM_001385177.1	XM_021857155.1
NM_001385195.1	XM_021856159.1
NM_001385217.1	XM_001650214.2
NM_001385218.1	XM_021853681.1
NM_001385245.1	XM_021844103.1
NM_001385251.1	XM_001656041.2
NM_001385258.1	XM_021839589.1
NM_001385259.1	XM_021844411.1
NM_001385274.1	XM_001658056.2
NM_001385292.1	XM_001657034.2
NM_001385321.1	XR_002501773.1
NM_001385341.1	XR_002501794.1
NM_001385348.1	XM_021856523.1
NM_001385351.1	XM_021840915.1
NM_001385354.1	XM_021853148.1
NM_001385361.1	XM_001654123.2
NM_001385368.1	XR_002500172.1
NM_001385374.1	XM_021853678.1
NM_001385426.1	XM_021849975.1
NM_001385429.1	XM_021855692.1
NM_001385444.1	XM_021848429.1
NM_001385463.1	XM_001660758.2
NM_001385478.1	XM_021848225.1
NM_001385491.1	XM_001652606.2
NM_001385509.1	XM_021846156.1
NM_001385531.1	XM_021851186.1
NM_001385551.1	XM_021848743.1
NM_001385558.1	XM_011495301.2
NM_001385618.1	XM_021854227.1
NM_001385621.1	XM_021851399.1
NM_001385673.1	XM_001661400.2
NM_001385676.1	XR_002499119.1
NM_001385689.1	XM_001663611.2
NM_001385693.1	XM_021847437.1

NM_001385703.1	XM_021847520.1
NM_001385705.1	XM_001663921.2
NM_001385730.1	XM_001653240.2
NM_001385735.1	XM_021838211.1
NM_001385748.1	XM_021840261.1
NM_001385757.1	XR_002501515.1
NM_001385769.1	XM_001651775.2
NM_001385778.1	XM_021852847.1
NM_001385784.1	XM_021850994.1
NM_001385792.1	XM_001656277.2
NM_001385809.1	XM_021850441.1
NM_001385812.1	XM_021848688.1
NM_001385821.1	XM_021848322.1
NM_001385883.1	XM_001653985.2
NM_001385905.1	XM_021854498.1
NM_001385913.1	XM_021850354.1
NM_001385947.1	XM_021841963.1
NM_001385967.1	XR_002500955.1
NM_001385997.1	XM_021842236.1
NM_001386004.1	XM_021843753.1
NM_001386015.1	XM_021852670.1
NM_001386019.1	XM_021844992.1
NM_001386027.1	XM_001653727.2
NM_001386032.1	XM_021854142.1
NM_001386084.1	XM_001648445.2
NM_001386095.1	XR_002500627.1
NM_001386139.1	XM_021854370.1
NM_001386141.1	XM_021844178.1
NM_001386146.1	XM_021846184.1
NM_001386191.1	XM_021850175.1
NM_001386193.1	XM_001652150.2
NM_001386198.1	XM_001659354.2
NM_001386208.1	XR_002502887.1
NM_001386290.1	XM_021844813.1
NM_001386425.1	XM_021846895.1
NM_001386430.1	XM_001652293.2
NM_001386441.1	XM_021855978.1
NM_001386462.1	XR_002498737.1
NM_001386585.1	XM_021851144.1
NM_001386608.1	XM_001663038.2
NM_001386609.1	XM_001655895.2
NM_001386677.1	XM_021839662.1
NM_001386682.1	XM_001661866.2
NM_001386793.1	XM_021854022.1
NM_001386901.1	XM_001652130.2
NM_001386929.1	XM_021845599.1
NM_001386934.1	XM_021841637.1
NM_001386968.1	XM_021857440.1
NM_001386978.1	XR_002498738.1
NM_001387002.1	XM_021842336.1

NM_001387009.1	XM_021846142.1
NM_001387053.1	XM_021844990.1
NM_001387063.1	XM_021844988.1
NM_001387067.1	XR_002502188.1
NM_001387096.1	XM_021838669.1
NM_001387097.1	XM_001653912.2
NM_001387102.1	XM_001664122.2
NM_001387112.1	XM_021844179.1
NM_001387119.1	XR_002500539.1
NM_001387138.1	XM_021848751.1
NM_001387195.1	XR_002503263.1
NM_001387206.1	XM_021854358.1
NM_001387230.1	XM_021851148.1
NM_001387260.1	XM_001649850.2
NM_001387272.1	XM_021841961.1
NM_001387302.1	XM_021852846.1
NM_001387303.1	XM_021847018.1
NM_001387308.1	XM_001651473.2
NM_001387317.1	XM_021850806.1
NM_001387318.1	XR_002499340.1
NM_001387320.1	XM_001657890.2
NM_001387347.1	XM_021844334.1
NM_001387379.1	XM_001655370.2
NM_001387401.1	XM_021843707.1
NM_001387412.1	XM_001654136.2
NM_001387418.1	XM_021852068.1
NM_001387446.1	XM_021848111.1
NM_001387481.1	XM_001654210.2
NM_001387490.1	XM_001652234.2
NM_001387501.1	XM_021845022.1
NM_001387505.1	XM_001651929.2
NM_001387526.1	XM_001648519.2
NM_001387562.1	XM_001661204.2
NM_001387569.1	XM_021844046.1
NM_001387598.1	XM_021855139.1
NM_001387623.1	XM_021854125.1
NM_001387650.1	XM_021837914.1
NM_001387652.1	XR_002502150.1
NM_001387663.1	XM_021850328.1
NM_001387697.1	XM_021853606.1
NM_001387762.1	XM_021849693.1
NM_001387777.1	XM_021853455.1
NM_001387780.1	XM_021842342.1
NM_001387794.1	XR_002500740.1
NM_001387813.1	XR_002501271.1
NM_001387821.1	XR_002499261.1
NM_001387856.1	XM_001659225.2
NM_001387862.1	XM_021837921.1
NM_001387919.1	XM_021842739.1
NM_001387940.1	XM_021857104.1

NM_001387960.1	XM_001649090.2
NM_001387968.1	XM_021852093.1
NM_001387971.1	XM_021857459.1
NM_001387976.1	XM_021838767.1
NM_001387984.1	XM_021854357.1
NM_001388001.1	XM_021844101.1
NM_001388009.1	XR_002500098.1
NM_001388013.1	XM_011495296.2
NM_001388042.1	XM_021840327.1
NM_001388043.1	XM_001657189.2
NM_001388083.1	XM_021857315.1
NM_001388091.1	XM_021857501.1
NM_001388109.1	XM_021854027.1
NM_001388111.1	XM_021849297.1
NM_001388118.1	XM_001654951.2
NM_001388127.1	XM_001655001.2
NM_001388133.1	XM_021846235.1
NM_001388137.1	XM_021849851.1
NM_001388151.1	XM_021855689.1
NM_001388165.1	XM_021838213.1
NM_001388167.1	XR_002501070.1
NM_001388171.1	XM_021847517.1
NM_001388188.1	XM_001655954.2
NM_001388191.1	XM_021857280.1
NM_001388200.1	XM_021854554.1
NM_001388215.1	XM_001651906.2
NM_001388223.1	XM_021855694.1
NM_001388235.1	XM_021848314.1
NM_001388256.1	XM_021845477.1
NM_001388265.1	XM_021841116.1
NM_001388269.1	XM_021840416.1
NM_001388285.1	XM_001650260.2
NM_001388288.1	XR_002502722.1
NM_001388300.1	XM_001660397.2
NM_001388306.1	XR_002500575.1
NM_001388358.1	XM_021847556.1
NM_001388380.1	XM_001654726.2
NM_001388391.1	XM_001657155.3
NM_001388401.1	XM_021845006.1
NM_001388410.1	XM_021840098.1
NM_001388427.1	XM_021841319.1
NM_001388496.1	XM_021843527.1
NM_001396.5	XM_021849152.1
NM_001399.5	XR_002502744.1
NM_001405.4	XM_001653709.2
NM_001458.5	XM_021856679.1
NM_001464.5	XR_002501427.1
NM_001487.3	XM_021846537.1
NM_001521.4	XM_021856274.1
NM_001535.5	XM_021854631.1

NM_001545.3	XM_001663633.2
NM_001546.4	XM_001659878.2
NM_001556.3	XM_021857643.1
NM_001605.3	XM_001661756.2
NM_001618.4	XM_001656424.2
NM_001619.5	XR_002500970.1
NM_001640.4	XM_021850903.1
NM_001669.4	XR_002501098.1
NM_001674.4	XM_021839941.1
NM_001718.6	XM_021844978.1
NM_001729.4	XM_001648346.2
NM_001731.3	XM_001661212.2
NM_001741.3	XM_001658813.2
NM_001745.4	XM_021845038.1
NM_001834.5	XM_001660393.2
NM_001858.6	XM_021837698.1
NM_001876.4	XM_001651070.2
NM_001895.4	XM_021847655.1
NM_001939.3	XM_021842284.1
NM_001951.4	XM_001649124.2
NM_001966.4	XM_001648252.2
NM_001988.4	XM_021850926.1
NM_001992.5	XM_021844020.1
NM_001997.5	XM_001659901.2
NM_002040.4	XM_001662483.2
NM_002086.5	XM_021842560.1
NM_002092.4	XM_021857105.1
NM_002122.5	XM_021840100.1
NM_002127.6	XM_021849661.1
NM_002188.3	XM_021849980.1
NM_002235.4	XM_021849483.1
NM_002241.5	XM_021844941.1
NM_002258.3	XM_021848315.1
NM_002262.5	XM_021854835.1
NM_002264.4	XM_021848822.1
NM_002279.5	XM_001648840.2
NM_002317.7	XM_001651584.3
NM_002346.3	XM_001647885.2
NM_002348.4	XM_021849274.1
NM_002360.4	XM_021844335.1
NM_002362.4	XM_001658173.2
NM_002376.7	XM_021846037.1
NM_002383.4	XM_021850461.1
NM_002390.6	XM_011494854.2
NM_002409.5	XM_001655197.2
NM_002420.6	XM_021852786.1
NM_002441.5	XM_021848049.1
NM_002446.4	XM_021845929.1
NM_002449.5	XM_021848547.1
NM_002470.4	XM_021844974.1

NM_002477.1	XR_002502584.1
NM_002478.5	XR_002499986.1
NM_002483.7	XM_021843089.1
NM_002514.4	XM_021840211.1
NM_002522.4	XR_002500712.1
NM_002525.3	XM_021839940.1
NM_002537.3	XM_021854258.1
NM_002597.5	XM_001647887.2
NM_002599.5	XM_021848050.1
NM_002612.4	XM_021839752.1
NM_002622.5	XR_002501953.1
NM_002638.4	XM_021849001.1
NM_002653.5	XM_001648946.2
NM_002666.5	XM_021848036.1
NM_002674.4	XM_021838507.1
NM_002691.4	XM_021852291.1
NM_002715.4	XM_021855122.1
NM_002783.3	XM_021843166.1
NM_002795.4	XM_021839789.1
NM_002803.4	XM_001655221.2
NM_002813.7	XR_002498905.1
NM_002841.4	XM_021855690.1
NM_002853.4	XM_021840213.1
NM_002885.4	XM_001663117.2
NM_002951.5	XM_001658160.2
NM_002956.3	XM_011495461.2
NM_003035.2	XM_021843174.1
NM_003053.4	XM_021856963.1
NM_003096.4	XM_021848526.1
NM_003124.5	XR_002501917.1
NM_003141.4	XM_021838160.1
NM_003149.3	XM_001657791.2
NM_003155.3	XM_021840234.1
NM_003171.5	XM_021856061.1
NM_003203.5	XM_001662026.2
NM_003238.6	XM_021845255.1
NM_003250.6	XM_021853464.1
NM_003254.3	XM_021855511.1
NM_003265.3	XM_011494866.2
NM_003274.5	XM_001663294.2
NM_003278.3	XM_001655092.2
NM_003313.4	XM_021850166.1
NM_003319.4	XM_021840380.1
NM_003324.5	XM_021850927.1
NM_003331.5	XM_021842777.1
NM_003347.4	XM_021845688.1
NM_003377.5	XM_001663929.2
NM_003392.7	XM_021837287.1
NM_003407.5	XM_021837257.1
NM_003409.5	XM_021846026.1

NM_003445.4	XM_021845090.1
NM_003446.4	XM_001648386.2
NM_003490.4	XM_001648254.2
NM_003503.4	XR_002502127.1
NM_003504.5	XM_001658214.2
NM_003510.3	XM_001654682.3
NM_003514.2	XM_021844520.1
NM_003569.3	XM_001648152.2
NM_003610.4	XM_021852929.1
NM_003617.4	XM_021849976.1
NM_003633.4	XM_001656237.2
NM_003639.4	XM_021843938.1
NM_003645.4	XM_021842793.1
NM_003654.6	XR_002502045.1
NM_003670.3	XM_021854445.1
NM_003702.4	XM_021854748.1
NM_003710.4	XM_021857290.1
NM_003735.3	XM_021847937.1
NM_003799.3	XM_021846351.1
NM_003812.4	XM_021847944.1
NM_003854.4	XM_021848375.1
NM_003884.5	XM_021852876.1
NM_003907.3	XR_002499941.1
NM_003948.5	XM_001648247.2
NM_003959.3	XM_021844104.1
NM_003963.3	XM_021857521.1
NM_003974.4	XM_021853352.1
NM_003981.4	XR_002502802.1
NM_003989.5	XM_021848388.1
NM_003992.5	XM_001655130.2
NM_004000.3	XM_001649911.2
NM_004010.3	XM_001662529.2
NM_004039.3	XM_021851683.1
NM_004041.5	XM_021851941.1
NM_004052.3	XM_021856774.1
NM_004061.5	XR_002500429.1
NM_004091.4	XM_021854834.1
NM_004094.5	XM_021857647.1
NM_004111.6	XM_021847938.1
NM_004112.4	XM_021855252.1
NM_004137.4	XR_002499494.1
NM_004145.4	XM_001662763.2
NM_004196.7	XM_021854247.1
NM_004197.2	XM_021854790.1
NM_004206.4	XM_001652376.2
NM_004208.4	XM_021850402.1
NM_004213.5	XM_021845847.1
NM_004214.5	XM_021839510.1
NM_004236.4	XM_021844626.1
NM_004246.3	XM_011495283.2

NM_004254.4	XM_021845230.1
NM_004257.6	XM_001655560.2
NM_004279.3	XM_021848446.1
NM_004316.4	XM_021845577.1
NM_004326.4	XM_021841197.1
NM_004354.3	XM_021847003.1
NM_004359.2	XM_021848447.1
NM_004362.3	XM_001663682.2
NM_004403.3	XM_021851371.1
NM_004418.4	XR_002502992.1
NM_004448.4	XM_021853648.1
NM_004452.4	XM_021840099.1
NM_004459.7	XM_021855695.1
NM_004492.3	XM_021842026.1
NM_004507.4	XM_021843761.1
NM_004508.4	XM_001653075.2
NM_004524.3	XM_021854789.1
NM_004532.6	XM_021842931.1
NM_004535.3	XR_002500139.1
NM_004544.4	XM_001656305.2
NM_004606.5	XM_021848935.1
NM_004660.5	XR_002499027.1
NM_004663.5	XR_002501645.1
NM_004688.3	XM_021848567.1
NM_004690.4	XM_001654020.2
NM_004720.7	XM_001652913.2
NM_004764.5	XM_021857441.1
NM_004769.4	XM_021853587.1
NM_004821.3	XM_021843522.1
NM_004840.3	XM_021844250.1
NM_004854.5	XM_021850697.1
NM_004861.3	XM_021855578.1
NM_004863.4	XM_001652970.2
NM_004896.5	XM_021856581.1
NM_004901.5	XM_021855646.1
NM_004902.4	XM_021840293.1
NM_004904.4	XM_021847635.1
NM_004942.4	XM_001653335.2
NM_004945.4	XM_021855870.1
NM_004953.5	XM_021844293.1
NM_004959.5	XR_002499163.1
NM_004972.4	XR_002498866.1
NM_004981.2	XM_021855618.1
NM_004994.3	XM_001654003.2
NM_005014.3	XM_021837341.1
NM_005020.5	XM_001656408.2
NM_005028.5	XM_021849534.1
NM_005032.7	XM_001654543.2
NM_005045.4	XM_001659155.2
NM_005052.3	XR_002502414.1

NM_005061.3	XM_021852058.1
NM_005103.5	XM_021855516.1
NM_005131.3	XM_021839629.1
NM_005156.7	XM_021845224.1
NM_005175.3	XR_002501624.1
NM_005186.4	XM_001654160.2
NM_005189.3	XM_021853143.1
NM_005191.4	XM_021841737.1
NM_005230.4	XM_021843191.1
NM_005235.3	XM_001662404.2
NM_005258.3	XM_021845467.1
NM_005262.3	XM_021848319.1
NM_005278.5	XM_021857463.1
NM_005294.3	XM_021849075.1
NM_005313.5	XM_021855687.1
NM_005319.4	XM_021849500.1
NM_005327.7	XM_001650932.2
NM_005360.5	XM_021846295.1
NM_005365.5	XM_021842794.1
NM_005372.1	XM_001653158.2
NM_005374.5	XR_002499323.1
NM_005387.7	XR_002498692.1
NM_005398.7	XM_001658094.2
NM_005426.3	XM_001650248.2
NM_005460.4	XM_021857074.1
NM_005469.4	XM_001659913.2
NM_005486.3	XM_001658884.2
NM_005496.3	XM_001655116.2
NM_005530.3	XM_021856973.1
NM_005534.4	XM_021846930.1
NM_005537.5	XM_001650071.2
NM_005554.4	XM_021838685.1
NM_005568.5	XM_021848621.1
NM_005600.3	XM_021850947.1
NM_005640.3	XM_021852842.1
NM_005658.5	XR_002500962.1
NM_005670.4	XM_021840629.1
NM_005691.4	XM_021856013.1
NM_005720.4	XM_021841927.1
NM_005729.4	XM_021843458.1
NM_005741.5	XM_021845383.1
NM_005744.5	XM_001660975.2
NM_005745.8	XM_021844874.1
NM_005802.5	XM_021847814.1
NM_005803.4	XM_021855338.1
NM_005817.5	XM_021843878.1
NM_005819.6	XM_021847943.1
NM_005824.3	XM_021840369.1
NM_005838.4	XR_002502382.1
NM_005894.3	XR_002502163.1

NM_005902.4	XM_021847054.1
NM_005914.4	XM_021854333.1
NM_005920.4	XM_001658008.2
NM_005928.4	XM_021844627.1
NM_005968.5	XM_001653812.2
NM_005987.4	XM_021843270.1
NM_006003.3	XM_021851129.1
NM_006009.4	XM_021849912.1
NM_006016.6	XM_021838966.1
NM_006035.4	XR_002501370.1
NM_006080.3	XM_021839703.1
NM_006092.4	XM_021839601.1
NM_006134.7	XM_021849190.1
NM_006140.6	XM_021852849.1
NM_006156.3	XM_021846176.1
NM_006177.5	XM_021854800.1
NM_006192.5	XR_002499060.1
NM_006237.4	XM_021854475.1
NM_006242.4	XM_021854381.1
NM_006246.5	XM_021843194.1
NM_006301.4	XM_001660080.2
NM_006315.6	XM_001655013.2
NM_006331.8	XM_021854555.1
NM_006341.4	XM_021856117.1
NM_006345.4	XM_011495344.2
NM_006350.5	XM_021839228.1
NM_006391.3	XM_021853635.1
NM_006408.4	XM_001648071.2
NM_006424.3	XM_021848105.1
NM_006456.3	XM_021857466.1
NM_006460.3	XM_021856515.1
NM_006462.6	XM_021846075.1
NM_006467.3	XM_021847567.1
NM_006480.5	XM_021857640.1
NM_006495.4	XR_002500665.1
NM_006536.7	XM_021847175.1
NM_006560.4	XM_021854386.1
NM_006567.5	XM_021850980.1
NM_006573.5	XM_001657334.2
NM_006585.4	XM_021845479.1
NM_006615.3	XM_021854264.1
NM_006653.5	XM_021849502.1
NM_006657.3	XM_001664068.2
NM_006668.2	XM_021856640.1
NM_006700.3	XR_002500053.1
NM_006721.4	XM_021856641.1
NM_006734.4	XM_021838648.1
NM_006755.2	XR_002500010.1
NM_006790.3	XM_021842252.1
NM_006808.3	XM_021848626.1

NM_006818.4	XM_021843035.1
NM_006828.4	XM_021856532.1
NM_006829.3	XM_021852431.1
NM_006833.5	XM_021842762.1
NM_006868.4	XM_021845373.1
NM_006875.4	XM_021842545.1
NM_006888.6	XM_021844184.1
NM_006928.5	XM_001663342.2
NM_006932.5	XM_001658735.2
NM_006980.5	XM_001654145.2
NM_006999.6	XR_002501443.1
NM_007006.3	XM_021846932.1
NM_007007.3	XM_021842589.1
NM_007010.5	XM_021845697.1
NM_007108.4	XM_021842366.1
NM_007112.5	XM_001656937.2
NM_007118.4	XM_001650437.2
NM_007134.1	XM_021848316.1
NM_007192.4	XM_021854174.1
NM_007194.4	XM_001661402.2
NM_007196.4	XM_001653671.3
NM_007210.4	XM_021854259.1
NM_007211.5	XM_021857336.1
NM_007219.5	XM_021843455.1
NM_007223.3	XM_001661474.2
NM_007267.7	XM_001648244.2
NM_007276.5	XM_021851280.1
NM_007278.2	XM_021844082.1
NM_007294.4	XR_002502844.1
NM_007333.2	XR_002502721.1
NM_009589.5	XM_021853768.1
NM_012088.3	XM_021846177.1
NM_012116.4	XM_021847940.1
NM_012130.4	XM_021841960.1
NM_012133.6	XM_001659167.2
NM_012138.4	XM_021855515.1
NM_012154.5	XM_021840480.1
NM_012164.4	XM_021851338.1
NM_012182.3	XM_021857642.1
NM_012207.3	XM_021846790.1
NM_012257.4	XM_021856737.1
NM_012265.3	XM_001653004.2
NM_012282.4	XM_021852404.1
NM_012294.5	XM_001661916.2
NM_012402.5	XM_021849231.1
NM_012413.4	XM_021845100.1
NM_012423.4	XM_021848622.1
NM_012427.5	XM_001661687.2
NM_012461.3	XM_021841749.1
NM_013239.5	XM_021851614.1

NM_013246.3	XM_021849604.1
NM_013249.4	XM_001657750.2
NM_013252.3	XM_021856015.1
NM_013264.5	XM_021847578.1
NM_013306.5	XM_001654327.2
NM_013308.4	XM_021842419.1
NM_013363.4	XM_001663344.2
NM_013365.5	XM_021850449.1
NM_013410.4	XR_002501987.1
NM_013444.4	XM_021847997.1
NM_013449.4	XM_021842259.1
NM_013450.4	XM_001660021.2
NM_013975.4	XM_021839793.1
NM_014015.4	XM_021842645.1
NM_014017.4	XM_021848605.1
NM_014045.5	XM_021843927.1
NM_014053.4	XM_021839936.1
NM_014058.4	XM_021850526.1
NM_014060.3	XM_021855166.1
NM_014064.4	XM_021850082.1
NM_014071.5	XM_021853141.1
NM_014203.3	XM_021845758.1
NM_014205.4	XM_021855914.1
NM_014264.5	XM_021856214.1
NM_014276.4	XM_001653563.2
NM_014284.3	XM_021840976.1
NM_014287.4	XM_021857550.1
NM_014339.7	XM_001654331.2
NM_014343.3	XM_001660376.2
NM_014349.3	XR_002501678.1
NM_014480.4	XM_021843653.1
NM_014482.3	XM_001660208.2
NM_014488.5	XM_021841588.1
NM_014563.6	XM_021838207.1
NM_014596.6	XR_002499194.1
NM_014598.4	XM_001652416.2
NM_014602.3	XM_021857579.1
NM_014633.5	XR_002502317.1
NM_014639.4	XM_021849852.1
NM_014640.5	XM_021848418.1
NM_014709.4	XM_021838711.1
NM_014757.5	XM_021847188.1
NM_014812.3	XM_021852699.1
NM_014813.3	XM_021850463.1
NM_014822.4	XM_001663572.2
NM_014848.7	XM_021852810.1
NM_014861.4	XM_021845384.1
NM_014872.3	XM_021850046.1
NM_014882.3	XM_001655952.2
NM_014886.6	XM_021842135.1

NM_014903.6	XR_002502676.1
NM_014912.5	XM_021855418.1
NM_014917.4	XM_021847636.1
NM_014944.4	XM_021844970.1
NM_014961.5	XM_001661027.2
NM_014977.3	XM_021845013.1
NM_015000.4	XR_002500800.1
NM_015025.4	XM_021853767.1
NM_015082.2	XM_021843064.1
NM_015114.3	XM_021847714.1
NM_015147.3	XM_021849292.1
NM_015151.4	XM_001650570.2
NM_015189.3	XM_021847733.1
NM_015196.4	XM_001657619.2
NM_015205.3	XM_021855676.1
NM_015232.2	XM_021851898.1
NM_015241.3	XR_002502203.1
NM_015245.3	XM_021854749.1
NM_015253.2	XM_021856334.1
NM_015264.2	XM_021857291.1
NM_015266.3	XM_021848217.1
NM_015270.5	XM_001651977.2
NM_015284.4	XR_002499372.1
NM_015319.2	XM_021839201.1
NM_015323.5	XM_021844968.1
NM_015331.3	XM_021838716.1
NM_015367.4	XM_021837653.1
NM_015395.3	XM_021851476.1
NM_015425.6	XR_002502860.1
NM_015434.4	XM_021837786.1
NM_015436.4	XM_021850178.1
NM_015471.4	XM_021840726.1
NM_015513.6	XM_021845481.1
NM_015566.3	XM_001662749.2
NM_015567.2	XR_002501845.1
NM_015575.4	XM_021857148.1
NM_015596.3	XM_021854875.1
NM_015633.3	XM_021849240.1
NM_015646.6	XM_021843827.1
NM_015657.4	XM_021855517.1
NM_015692.5	XM_021838805.1
NM_015693.4	XM_021854883.1
NM_015716.5	XM_011495463.2
NM_015719.4	XM_001656380.2
NM_015722.4	XM_021842862.1
NM_015723.5	XM_021850017.1
NM_015853.5	XM_021856171.1
NM_015967.7	XM_021838524.1
NM_015986.4	XM_021843883.1
NM_016003.4	XM_021855419.1

NM_016021.3	XR_002500079.1
NM_016026.4	XM_001661783.2
NM_016033.3	XM_021850465.1
NM_016083.6	XM_021839230.1
NM_016085.5	XM_021851400.1
NM_016111.4	XM_021844415.1
NM_016148.5	XM_001649704.2
NM_016161.3	XM_001650744.2
NM_016170.5	XM_021856453.1
NM_016178.2	XM_021853789.1
NM_016184.4	XM_021857332.1
NM_016235.3	XM_021851757.1
NM_016275.5	XM_021840812.1
NM_016284.5	XM_021848158.1
NM_016287.5	XM_001659479.2
NM_016315.4	XM_021838390.1
NM_016321.3	XM_021838503.1
NM_016322.4	XM_021843517.1
NM_016323.4	XM_021848060.1
NM_016360.4	XM_021844285.1
NM_016379.4	XM_021840850.1
NM_016464.5	XM_021841323.1
NM_016541.3	XR_002502449.1
NM_016553.5	XM_021847872.1
NM_016562.4	XM_021856176.1
NM_016568.3	XR_002501877.1
NM_016605.3	XM_001660101.3
NM_016617.4	XM_021843945.1
NM_016628.5	XM_021854050.1
NM_016643.4	XM_021843243.1
NM_016652.6	XM_021845247.1
NM_016734.3	XM_021838391.1
NM_016829.3	XM_021851004.1
NM_017514.5	XM_021854524.1
NM_017516.3	XM_021848635.1
NM_017522.5	XM_021847553.1
NM_017527.4	XM_021844300.1
NM_017533.2	XM_021844522.1
NM_017548.5	XM_021839663.1
NM_017576.4	XM_021848002.1
NM_017620.3	XM_021841114.1
NM_017659.4	XM_021851630.1
NM_017661.4	XM_021857478.1
NM_017676.2	XM_021855673.1
NM_017677.4	XM_021854585.1
NM_017686.4	XM_021844708.1
NM_017705.4	XM_021848455.1
NM_017714.3	XM_021851758.1
NM_017719.5	XM_021850630.1
NM_017789.5	XM_021857193.1

NM_017816.3	XM_021854266.1
NM_017831.4	XR_002501574.1
NM_017832.4	XM_021851413.1
NM_017867.3	XR_002499304.1
NM_017872.5	XM_021854250.1
NM_017891.5	XM_001651387.2
NM_017899.4	XR_002502253.1
NM_017905.6	XM_001653355.2
NM_017954.11	XM_021854385.1
NM_017956.4	XM_021840914.1
NM_018003.4	XR_002501500.1
NM_018047.3	XM_021843976.1
NM_018059.5	XR_002503045.1
NM_018062.4	XM_021855840.1
NM_018095.6	XR_002500504.1
NM_018099.5	XM_021846788.1
NM_018120.6	XM_001660197.2
NM_018166.3	XM_021844749.1
NM_018188.5	XM_021846737.1
NM_018223.2	XM_021855142.1
NM_018242.3	XM_021853295.1
NM_018270.6	XM_001658851.2
NM_018313.5	XM_001658130.2
NM_018314.6	XM_021852851.1
NM_018318.5	XM_021844750.1
NM_018324.3	XM_021853253.1
NM_018330.7	XM_001658604.2
NM_018340.3	XM_021845341.1
NM_018342.5	XM_021845714.1
NM_018374.4	XM_021840674.1
NM_018383.5	XM_021839626.1
NM_018400.4	XM_001658442.2
NM_018404.3	XM_001660361.2
NM_018451.5	XM_001660346.2
NM_018455.6	XM_001650902.2
NM_018480.7	XM_021844620.1
NM_018487.3	XM_001654332.2
NM_018561.5	XM_021852989.1
NM_018648.4	XM_001663218.2
NM_018651.4	XM_021850267.1
NM_018669.6	XM_021838110.1
NM_018724.4	XM_001658613.2
NM_018837.4	XM_001657980.3
NM_018838.5	XM_021844862.1
NM_018842.5	XM_021856771.1
NM_018898.5	XM_001651960.2
NM_018946.4	XM_001660751.2
NM_018952.5	XM_021849634.1
NM_018956.5	XM_021849444.1
NM_018963.5	XM_021838977.1

NM_018970.7	XM_021846735.1
NM_018981.4	XM_021842396.1
NM_018990.4	XM_021837620.1
NM_018999.4	XM_021853616.1
NM_019046.3	XM_021842617.1
NM_019112.4	XM_021840611.1
NM_019116.3	XR_002502491.1
NM_019120.5	XM_021853601.1
NM_019601.4	XM_021843820.1
NM_019844.4	XM_021847451.1
NM_019849.3	XM_021848661.1
NM_020133.3	XM_001654035.2
NM_020154.3	XM_001659808.2
NM_020164.5	XM_021844115.1
NM_020166.5	XR_002500787.1
NM_020171.2	XM_021838061.1
NM_020203.5	XM_021839226.1
NM_020207.7	XM_021845394.1
NM_020228.3	XR_002499903.1
NM_020244.3	XM_021855165.1
NM_020248.3	XM_021850702.1
NM_020337.3	XM_021845136.1
NM_020376.4	XR_002500838.1
NM_020377.5	XM_021842398.1
NM_020409.3	XM_021844006.1
NM_020422.6	XM_021856691.1
NM_020425.6	XM_001661052.2
NM_020469.3	XM_021838960.1
NM_020485.6	XM_021856749.1
NM_020528.3	XM_021855584.1
NM_020677.6	XM_021850043.1
NM_020692.3	XM_001660707.2
NM_020741.3	XM_001655106.2
NM_020760.4	XM_021847486.1
NM_020765.3	XM_021855867.1
NM_020781.4	XR_002502926.1
NM_020784.3	XM_021838342.1
NM_020824.4	XM_021857266.1
NM_020827.3	XM_021847455.1
NM_020830.5	XM_011495178.2
NM_020839.4	XM_021844113.1
NM_020853.2	XM_021848937.1
NM_020886.4	XM_001659822.2
NM_020911.2	XM_021848122.1
NM_020920.4	XM_021837546.1
NM_020959.3	XM_021857019.1
NM_020962.3	XM_001657957.3
NM_020984.4	XM_001654924.2
NM_020989.4	XM_021853360.1
NM_021033.7	XR_002502777.1

NM_021072.4	XM_021845515.1
NM_021104.2	XM_021853014.1
NM_021110.4	XR_002499151.1
NM_021134.4	XM_021840325.1
NM_021158.5	XM_001648222.2
NM_021168.5	XM_021856111.1
NM_021186.5	XM_021850047.1
NM_021192.3	XM_021849253.1
NM_021200.3	XM_021852402.1
NM_021202.3	XM_021846896.1
NM_021225.5	XM_021853391.1
NM_021245.4	XR_002499979.1
NM_021252.5	XM_021852889.1
NM_021574.3	XM_021846846.1
NM_021603.4	XM_021840851.1
NM_021623.2	XM_021838672.1
NM_021634.4	XM_001651377.2
NM_021643.4	XM_021843975.1
NM_021806.4	XM_001650372.2
NM_021983.4	XM_021848770.1
NM_022053.4	XM_001653662.2
NM_022058.4	XM_021846446.1
NM_022064.5	XR_002503146.1
NM_022098.4	XR_002501635.1
NM_022119.4	XM_021841985.1
NM_022135.4	XM_021850049.1
NM_022142.5	XM_021845770.1
NM_022170.2	XM_021840907.1
NM_022355.4	XM_021841754.1
NM_022437.3	XM_001647598.2
NM_022464.5	XM_021843467.1
NM_022468.5	XM_021852239.1
NM_022568.4	XM_021855854.1
NM_022645.2	XR_002501197.1
NM_022727.6	XM_021845395.1
NM_022757.5	XM_021851795.1
NM_022764.3	XM_021844507.1
NM_022765.4	XR_002499855.1
NM_022774.3	XM_021840059.1
NM_022784.3	XM_021847833.1
NM_022817.3	XM_001657987.2
NM_022818.5	XM_001649803.2
NM_022823.3	XM_021849442.1
NM_022902.5	XM_021846867.1
NM_023000.3	XR_002503086.1
NM_023915.4	XR_002500641.1
NM_023918.3	XM_001663680.2
NM_023937.4	XM_021848147.1
NM_023940.3	XM_021846327.1
NM_023946.5	XM_021839977.1

NM_024011.4	XM_021844131.1
NM_024059.3	XM_021840111.1
NM_024061.4	XM_021852698.1
NM_024067.4	XM_021853252.1
NM_024120.5	XM_001652178.2
NM_024121.3	XM_021852245.1
NM_024299.4	XM_021840049.1
NM_024324.5	XM_001648798.2
NM_024504.4	XM_021853901.1
NM_024512.5	XM_021849630.1
NM_024525.5	XM_021842037.1
NM_024528.4	XM_001648314.2
NM_024541.3	XM_021839171.1
NM_024556.4	XM_001656578.2
NM_024568.4	XM_021842374.1
NM_024577.4	XM_021843973.1
NM_024594.4	XM_021841308.1
NM_024598.4	XM_001656954.3
NM_024613.4	XM_021850371.1
NM_024617.4	XM_021848146.1
NM_024644.5	XM_021838825.1
NM_024687.4	XM_021837983.1
NM_024729.4	XM_021848713.1
NM_024754.5	XM_001656969.2
NM_024770.5	XM_021845615.1
NM_024783.4	XM_001656664.2
NM_024792.3	XM_021848139.1
NM_024798.3	XR_002500274.1
NM_024806.4	XM_021850374.1
NM_024830.5	XM_021841699.1
NM_024869.3	XM_021845638.1
NM_024872.4	XM_021845727.1
NM_024877.4	XM_021854419.1
NM_024894.4	XM_021854797.1
NM_024895.5	XM_021855856.1
NM_024899.4	XM_021844586.1
NM_024913.5	XM_001663357.2
NM_024939.3	XM_001655462.2
NM_024940.8	XM_021850887.1
NM_024955.6	XM_021850521.1
NM_024956.4	XM_021848144.1
NM_025052.5	XM_021845612.1
NM_025058.5	XM_001657178.2
NM_025142.1	XM_021855113.1
NM_025149.6	XM_001650856.2
NM_025160.7	XM_021841797.1
NM_025205.5	XM_021850434.1
NM_025233.7	XM_021851872.1
NM_025241.3	XM_001650649.2
NM_025258.3	XM_021841028.1

NM_025261.3	XM_021853176.1
NM_025264.5	XM_021855032.1
NM_030592.3	XM_021843236.1
NM_030631.4	XR_002503270.1
NM_030671.3	XM_021843974.1
NM_030763.3	XR_002500148.1
NM_030772.5	XR_002500829.1
NM_030779.4	XM_021852780.1
NM_030798.5	XR_002501172.1
NM_030820.4	XM_021842399.1
NM_030904.1	XM_021850276.1
NM_030912.3	XM_021843828.1
NM_030920.5	XM_021851441.1
NM_030927.4	XM_021851101.1
NM_030941.3	XM_021845046.1
NM_030943.4	XM_021850368.1
NM_030956.4	XM_001653621.3
NM_030961.3	XM_001652357.2
NM_030969.5	XM_021842283.1
NM_031231.4	XM_021852092.1
NM_031243.3	XM_021845488.1
NM_031268.6	XM_021845838.1
NM_031280.4	XM_021842403.1
NM_031294.4	XM_021846348.1
NM_031371.4	XM_001654644.2
NM_031444.4	XR_002500510.1
NM_031455.4	XM_021855764.1
NM_031495.2	XM_021856949.1
NM_031500.3	XM_021842524.1
NM_031852.2	XM_021850924.1
NM_031860.3	XM_001648903.2
NM_031922.5	XM_021851368.1
NM_031961.3	XM_001659147.2
NM_031965.2	XM_021853299.1
NM_032044.4	XM_021851326.1
NM_032049.4	XM_021847735.1
NM_032109.3	XM_001656631.2
NM_032111.4	XM_021839066.1
NM_032118.4	XM_021854722.1
NM_032125.3	XM_021842346.1
NM_032127.4	XR_002498687.1
NM_032145.5	XM_021850290.1
NM_032149.3	XM_001659309.2
NM_032164.4	XM_001653664.2
NM_032181.3	XM_001647738.2
NM_032268.5	XM_021838387.1
NM_032271.3	XM_021842957.1
NM_032360.4	XM_021844495.1
NM_032373.5	XM_001654255.2
NM_032375.5	XM_021842157.1

NM_032408.4	XM_001655976.2
NM_032414.3	XM_001662958.2
NM_032477.3	XR_002498923.1
NM_032523.4	XM_011494635.2
NM_032524.2	XM_021852216.1
NM_032536.4	XM_021847173.1
NM_032539.5	XM_001659698.2
NM_032550.4	XM_021841640.1
NM_032554.4	XM_001656410.2
NM_032630.3	XM_021853265.1
NM_032681.4	XM_001657060.2
NM_032796.4	XM_021847918.1
NM_032816.5	XM_021849592.1
NM_032831.4	XM_021852328.1
NM_032843.5	XM_021839530.1
NM_032866.5	XM_021839301.1
NM_032869.4	XM_021843446.1
NM_032881.3	XM_001655217.2
NM_032980.4	XM_001661051.2
NM_033011.4	XM_021839263.1
NM_033056.4	XM_001663673.2
NM_033070.3	XM_001651810.2
NM_033092.4	XR_002501058.1
NM_033110.3	XM_001664039.2
NM_033113.3	XM_021838839.1
NM_033123.4	XR_002498651.1
NM_033169.2	XM_021845031.1
NM_033172.3	XR_002498644.1
NM_033180.5	XR_002499503.1
NM_033181.4	XM_021854312.1
NM_033191.3	XM_001655475.2
NM_033253.4	XM_021846579.1
NM_033289.2	XM_001649708.2
NM_033293.4	XM_021851681.1
NM_033295.4	XM_001662718.2
NM_033313.3	XM_021844621.1
NM_033342.4	XM_021840656.1
NM_033394.3	XM_021847719.1
NM_033413.4	XM_001661603.2
NM_033423.5	XM_021843644.1
NM_033503.4	XM_021854877.1
NM_033670.4	XM_021840725.1
NM_052868.6	XM_021837624.1
NM_052870.4	XM_021841752.1
NM_052879.5	XR_002499564.1
NM_052902.4	XM_021839178.1
NM_052904.4	XM_021857180.1
NM_052926.3	XM_021839857.1
NM_052934.4	XM_021840492.1
NM_052951.3	XM_021845188.1

NM_052955.3	XM_021849311.1
NM_052959.3	XM_001658229.2
NM_052961.4	XM_001654719.2
NM_053016.6	XM_001654979.2
NM_053023.5	XM_021854114.1
NM_053051.5	XM_021847815.1
NM_054021.2	XM_021846519.1
NM_057090.3	XM_021837755.1
NM_057164.5	XM_001652683.2
NM_057179.3	XM_001658137.2
NM_058163.3	XM_021845901.1
NM_058169.6	XM_001664064.2
NM_058176.2	XM_001658134.2
NM_058187.5	XM_021840296.1
NM_058197.5	XM_021853463.1
NM_078481.4	XM_021852791.1
NM_078485.4	XM_021839861.1
NM_078626.3	XM_011495173.2
NM_078628.2	XM_001660666.2
NM_080282.4	XM_021851948.1
NM_080423.3	XM_021854327.1
NM_080590.4	XR_002500631.1
NM_080659.3	XR_002501973.1
NM_080672.5	XR_002501944.1
NM_080679.3	XM_021851062.1
NM_080737.3	XM_021840860.1
NM_080833.3	XM_021852965.1
NM_080836.4	XM_021849716.1
NM_080838.3	XM_001655358.2
NM_080841.3	XM_021845259.1
NM_080862.3	XM_021842917.1
NM_080868.3	XM_001650797.2
NM_080872.4	XM_021844730.1
NM_080879.3	XM_001649364.2
NM_080913.3	XM_021854616.1
NM_130759.4	XM_021843213.1
NM_130762.3	XM_021852220.1
NM_130787.3	XM_001656421.2
NM_130852.3	XM_001663220.2
NM_133277.4	XM_021853472.1
NM_133344.3	XM_021844337.1
NM_133373.5	XM_021841644.1
NM_133506.3	XM_021842496.1
NM_133635.6	XM_021837639.1
NM_134425.4	XM_021849960.1
NM_134440.3	XM_001662875.2
NM_134442.5	XM_021856817.1
NM_138286.3	XM_001655960.2
NM_138324.3	XM_021850612.1
NM_138348.6	XM_021844773.1

NM_138355.4	XM_021840788.1
NM_138373.5	XM_021845480.1
NM_138391.6	XR_002501709.1
NM_138413.4	XM_001657749.2
NM_138431.3	XM_021844720.1
NM_138461.4	XR_002501527.1
NM_138476.4	XM_021852214.1
NM_138499.4	XM_001655434.2
NM_138700.4	XM_021847545.1
NM_138702.1	XM_021845632.1
NM_138705.4	XM_001663174.2
NM_138711.6	XM_021853467.1
NM_138714.4	XM_021837507.1
NM_138736.3	XM_021842859.1
NM_138771.4	XM_021842413.1
NM_138782.3	XM_021840791.1
NM_138788.5	XM_001650091.2
NM_138804.5	XM_021840191.1
NM_138810.4	XM_021839732.1
NM_138927.4	XM_001655012.2
NM_138940.3	XM_021838180.1
NM_138973.4	XR_002501286.1
NM_139052.3	XM_021841102.1
NM_139074.4	XM_021852707.1
NM_139075.4	XR_002502883.1
NM_139136.4	XM_021844569.1
NM_139137.4	XM_021855004.1
NM_139209.3	XM_001656632.2
NM_139246.5	XM_001656511.3
NM_139314.3	XM_021838157.1
NM_144569.6	XM_001649542.2
NM_144608.2	XM_021855590.1
NM_144617.3	XM_021841152.1
NM_144632.5	XM_021857043.1
NM_144666.3	XM_021853139.1
NM_144672.4	XM_001651364.2
NM_144691.4	XM_021840481.1
NM_144707.4	XM_001652727.2
NM_144720.4	XM_021849915.1
NM_144725.4	XM_021838784.1
NM_144777.3	XM_001648546.2
NM_144778.4	XM_001649235.2
NM_144976.4	XM_021841307.1
NM_144989.3	XM_001654512.2
NM_145071.4	XM_021852198.1
NM_145166.4	XR_002501843.1
NM_145177.3	XM_021853156.1
NM_145178.4	XM_021844729.1
NM_145186.3	XM_021846372.1
NM_145241.5	XM_021838179.1

NM_145248.5	XM_021857591.1
NM_145257.5	XM_021849514.1
NM_145278.5	XM_021850804.1
NM_145333.3	XM_021850805.1
NM_145658.4	XM_021855441.1
NM_145808.4	XM_021837756.1
NM_145888.3	XM_021851856.1
NM_147127.5	XM_001663647.2
NM_147185.3	XM_021855851.1
NM_147686.4	XM_001663698.2
NM_148963.4	XM_021843054.1
NM_152244.2	XM_021840790.1
NM_152246.3	XM_021840493.1
NM_152274.5	XM_021844575.1
NM_152280.5	XM_001651378.2
NM_152282.5	XM_021841383.1
NM_152317.4	XM_021847836.1
NM_152318.3	XM_001660068.2
NM_152331.4	XM_021850275.1
NM_152344.4	XM_021847075.1
NM_152365.3	XM_021851372.1
NM_152369.5	XM_001658043.2
NM_152373.4	XM_021839903.1
NM_152374.2	XM_021847721.1
NM_152417.3	XM_021852436.1
NM_152432.4	XM_021841905.1
NM_152467.5	XM_021852843.1
NM_152496.3	XR_002501695.1
NM_152498.3	XM_001649226.2
NM_152512.4	XR_002499022.1
NM_152613.3	XM_021841479.1
NM_152614.3	XM_021847989.1
NM_152628.4	XM_001648272.2
NM_152641.4	XM_021845047.1
NM_152654.3	XM_001654410.2
NM_152682.4	XM_021843520.1
NM_152694.3	XM_021839320.1
NM_152724.3	XM_021850772.1
NM_152731.3	XM_001651858.2
NM_152753.4	XR_002500917.1
NM_152779.4	XM_001657533.2
NM_152792.4	XM_021841901.1
NM_152795.4	XM_021851313.1
NM_152830.3	XM_021841746.1
NM_152841.2	XM_001662376.2
NM_152869.4	XM_001651874.2
NM_152926.3	XM_021856096.1
NM_152990.4	XR_002500447.1
NM_153006.3	XM_021856525.1
NM_153047.4	XM_021842717.1

NM_153211.4	XM_021842202.1
NM_153273.4	XM_001648690.2
NM_153332.4	XM_021849305.1
NM_153437.3	XM_021839231.1
NM_153439.1	XM_021839001.1
NM_153456.4	XM_021840450.1
NM_153478.3	XM_021853483.1
NM_153480.2	XM_021840322.1
NM_153608.4	XM_001659703.2
NM_153694.5	XM_001657875.2
NM_153695.4	XM_001648980.3
NM_153712.5	XM_021843442.1
NM_153713.3	XM_021853716.1
NM_170750.3	XM_021845051.1
NM_171829.3	XM_001661135.2
NM_172107.4	XM_001658859.2
NM_172108.5	XR_002500189.1
NM_172173.3	XR_002500002.1
NM_172225.2	XM_001651537.2
NM_172316.3	XR_002502928.1
NM_172341.4	XR_002502009.1
NM_173081.5	XM_021854738.1
NM_173156.3	XM_021848445.1
NM_173163.3	XM_021854515.1
NM_173208.3	XM_021844734.1
NM_173216.2	XR_002501122.1
NM_173491.4	XM_021850951.1
NM_173497.4	XM_021841909.1
NM_173512.2	XM_021856435.1
NM_173515.4	XM_021837515.1
NM_173591.7	XR_002500329.1
NM_173595.4	XR_002502914.1
NM_173619.4	XM_001660857.2
NM_173626.4	XM_021838568.1
NM_173637.4	XM_001662997.2
NM_173653.4	XM_021839760.1
NM_173678.3	XM_021840662.1
NM_173689.7	XM_001657506.2
NM_173701.2	XM_021849734.1
NM_173803.4	XM_021844655.1
NM_173808.3	XR_002501329.1
NM_173826.4	XM_021852407.1
NM_173831.4	XM_021856660.1
NM_174882.3	XM_021856197.1
NM_174894.3	XM_021856180.1
NM_174931.4	XM_021847555.1
NM_174934.4	XR_002502681.1
NM_174969.4	XM_021850156.1
NM_175039.4	XM_021850413.1
NM_175066.4	XR_002500265.1

NM_175569.3	XM_001649085.2
NM_175739.4	XM_021850053.1
NM_175861.3	XM_001656542.2
NM_176787.5	XM_021845582.1
NM_177452.4	XM_001661566.2
NM_177536.4	XM_021843723.1
NM_177937.3	XM_021851130.1
NM_177972.3	XM_001660445.2
NM_177990.4	XR_002498656.1
NM_178151.3	XM_021837691.1
NM_178174.3	XM_021839109.1
NM_178177.5	XM_021841108.1
NM_178232.4	XM_021856743.1
NM_178332.2	XM_021855194.1
NM_178353.2	XR_002499310.1
NM_178354.3	XM_021848828.1
NM_178356.3	XM_001660561.2
NM_178452.6	XM_021839637.1
NM_178517.5	XM_021846190.1
NM_178543.5	XR_002499496.1
NM_178569.4	XM_001661479.2
NM_178587.3	XM_021852993.1
NM_178812.4	XM_021842867.1
NM_178813.6	XR_002499347.1
NM_178862.3	XR_002499147.1
NM_180982.3	XM_021843554.1
NM_181078.3	XM_021851435.1
NM_181359.3	XM_021852059.1
NM_181523.3	XR_002501290.1
NM_181538.3	XM_021857490.1
NM_181644.5	XM_001663205.2
NM_181673.3	XM_001652914.2
NM_181676.3	XM_021855456.1
NM_181715.3	XM_021855216.1
NM_181790.1	XM_021853481.1
NM_181892.4	XM_001659792.2
NM_182489.2	XM_001653554.2
NM_182491.4	XM_021848788.1
NM_182497.4	XM_001661558.2
NM_182498.4	XM_021855208.1
NM_182513.3	XM_001662835.2
NM_182581.4	XM_021855390.1
NM_182616.4	XM_001652280.2
NM_182628.3	XM_021837294.1
NM_182691.3	XM_001655751.2
NM_182719.2	XM_001661243.2
NM_182724.2	XM_021852395.1
NM_182767.6	XM_001658318.2
NM_183059.3	XM_021840572.1
NM_183060.3	XM_001648695.2

NM_183239.2	XM_021845360.1
NM_183323.3	XM_021844758.1
NM_183352.3	XM_021841822.1
NM_183361.3	XM_021854662.1
NM_183372.9	XM_001655821.2
NM_183419.4	XR_002500081.1
NM_187841.3	XM_021854053.1
NM_194272.3	XM_021853264.1
NM_194278.3	XM_001654448.2
NM_194294.2	XM_021838365.1
NM_194332.3	XM_001663041.2
NM_194447.3	XM_001661650.2
NM_197954.3	XM_021856192.1
NM_197966.3	XM_021843722.1
NM_197970.3	XM_021852763.1
NM_198057.3	XM_021856110.1
NM_198098.4	XM_021854858.1
NM_198186.3	XM_021853050.1
NM_198205.2	XM_021845532.1
NM_198269.3	XM_021855761.1
NM_198273.2	XM_021851693.1
NM_198316.2	XM_021845787.1
NM_198383.3	XM_001648703.2
NM_198404.3	XM_021852787.1
NM_198476.5	XM_021848453.1
NM_198493.3	XM_001650093.2
NM_198498.3	XM_021853077.1
NM_198530.4	XR_002502772.1
NM_198531.5	XM_001663703.2
NM_198686.3	XM_021854299.1
NM_198687.2	XM_021846191.1
NM_198699.1	XM_021843304.1
NM_198977.2	XM_021843698.1
NM_198996.4	XM_021841784.1
NM_198999.3	XM_001660340.2
NM_199123.2	XM_021846548.1
NM_199127.2	XM_001648185.2
NM_199162.3	XM_021850139.1
NM_199177.4	XR_002500886.1
NM_199184.2	XM_021842204.1
NM_199231.2	XM_021842504.1
NM_199245.3	XM_021841305.1
NM_199263.3	XM_021838332.1
NM_199321.3	XM_001658373.2
NM_199334.5	XM_021841781.1
NM_199344.3	XM_021855855.1
NM_199350.3	XM_021845816.1
NM_199363.3	XM_021848127.1
NM_199478.3	XM_021852330.1
NM_199487.3	XM_001647941.2

NM_201262.2	XM_021852437.1
NM_201380.4	XR_002499828.1
NM_201402.3	XM_021840445.1
NM_201414.3	XM_001651231.2
NM_201440.2	XR_002499858.1
NM_201441.3	XM_021850624.1
NM_201525.4	XM_001650195.2
NM_201557.4	XM_021854661.1
NM_201575.4	XM_021851521.1
NM_201632.5	XM_021839265.1
NM_203295.2	XM_021848831.1
NM_203344.3	XM_021841482.1
NM_203374.2	XM_001656365.2
NM_203453.5	XM_021839851.1
NM_203459.3	XR_002499350.1
NM_205768.3	XM_001658649.2
NM_205862.2	XM_021848220.1
NM_206825.2	XM_001648022.2
NM_206890.3	XM_021848825.1
NM_206909.3	XM_021853570.1
NM_206920.3	XM_021840065.1
NM_207128.3	XM_001657185.2
NM_207334.3	XM_021851950.1
NM_207335.4	XM_001652445.2
NM_207352.4	XM_021854096.1
NM_207364.2	XM_021840523.1
NM_207372.2	XM_021838377.1
NM_207417.3	XM_021847564.1
NM_207426.3	XM_021839557.1
NM_207519.2	XM_021855410.1
NM_212472.2	XM_001660839.2
NM_212478.3	XM_021844203.1
NM_212543.2	XR_002500143.1
NM_213603.3	XM_001653037.2
NM_213674.1	XM_021848295.1
NR_001574.2	XR_002499002.1
NR_002190.1	XM_021855579.1
NR_002311.1	XM_001657874.2
NR_002717.2	XR_002499927.1
NR_002720.2	XM_001662534.2
NR_002724.2	XM_021845223.1
NR_002807.4	XM_021844731.1
NR_002832.2	XR_002500243.1
NR_002836.2	XM_001648188.2
NR_002923.2	XM_021852329.1
NR_003040.2	XM_021842363.1
NR_003090.3	XM_021842249.1
NR_003187.3	XM_021847816.1
NR_003189.2	XM_001653596.2
NR_003228.1	XM_021839519.1

NR_003260.1	XM_021843725.1
NR_015358.2	XM_021845617.1
NR_015419.2	XM_001664115.2
NR_015436.2	XM_021844810.1
NR_015440.1	XM_021840447.1
NR_015448.1	XM_021849940.1
NR_016021.2	XM_021851989.1
NR_023349.3	XM_021842165.1
NR_023385.1	XM_021845283.1
NR_024027.2	XM_021840660.1
NR_024035.2	XM_001655373.2
NR_024056.2	XM_021843870.1
NR_024122.2	XM_001659683.2
NR_024159.1	XR_002502268.1
NR_024162.1	XM_021852320.1
NR_024188.3	XM_021841971.1
NR_024237.2	XM_001656411.2
NR_024248.1	XM_001661062.2
NR_024256.1	XR_002500489.1
NR_024269.1	XM_021848914.1
NR_024274.1	XM_001655435.2
NR_024345.1	XM_001651868.2
NR_024397.1	XM_021854733.1
NR_024435.2	XM_021855552.1
NR_024448.2	XM_021845216.1
NR_024452.1	XM_021842166.1
NR_024455.1	XM_001662902.2
NR_024457.2	XM_001660023.2
NR_024511.2	XR_002499099.1
NR_024515.2	XM_021841981.1
NR_024531.1	XM_001652000.2
NR_024533.1	XM_001662372.2
NR_024542.1	XM_001663156.2
NR_024603.1	XM_021840665.1
NR_024606.2	XM_021847205.1
NR_026542.1	XM_021852675.1
NR_026564.1	XR_002503047.1
NR_026597.2	XM_021857142.1
NR_026656.1	XM_021852086.1
NR_026708.2	XM_021845839.1
NR_026803.2	XM_001649793.2
NR_026818.1	XM_021843764.1
NR_026825.2	XM_001651811.2
NR_026857.1	XM_021855504.1
NR_026873.1	XM_021851652.1
NR_026878.1	XM_021857264.1
NR_026929.1	XM_001653873.2
NR_026933.2	XM_001654852.2
NR_026943.1	XR_002502630.1
NR_026963.1	XM_021848827.1

NR_026971.1	XM_021841223.1
NR_026988.1	XM_021848350.1
NR_027055.1	XM_001662273.2
NR_027072.2	XM_021848273.1
NR_027073.1	XM_021845289.1
NR_027078.1	XM_001656905.2
NR_027097.2	XR_002502782.1
NR_027111.1	XR_002499143.1
NR_027148.1	XR_002500337.1
NR_027163.1	XM_021845115.1
NR_027176.1	XM_021838643.1
NR_027232.1	XM_021847509.1
NR_027242.1	XM_021845483.1
NR_027277.2	XM_021841608.1
NR_027279.1	XR_002500231.1
NR_027354.2	XM_021856307.1
NR_027355.2	XM_021857583.1
NR_027365.3	XM_021837288.1
NR_027368.2	XM_021847508.1
NR_027379.1	XM_021851228.1
NR_027413.2	XM_001654515.2
NR_027418.1	XM_021841639.1
NR_027451.1	XM_021838101.1
NR_027454.2	XM_001648518.2
NR_027514.3	XM_021857265.1
NR_027632.1	XM_021853783.1
NR_027645.1	XM_021846371.1
NR_027684.2	XM_001655596.2
NR_027711.1	XM_021852992.1
NR_027749.3	XM_021856722.1
NR_027754.2	XM_021849579.1
NR_027763.2	XM_021854618.1
NR_027855.2	XM_001654176.2
NR_027946.3	XM_021846955.1
NR_027948.3	XR_002499157.1
NR_028025.3	XM_021843933.1
NR_028035.4	XM_001654966.2
NR_028272.1	XM_001652239.2
NR_028415.1	XM_021849794.1
NR_028436.3	XM_021848513.1
NR_028477.2	XM_021845960.1
NR_029392.1	XM_021846623.1
NR_029403.2	XM_021848880.1
NR_029408.1	XM_021840378.1
NR_030697.1	XM_021855076.1
NR_030770.2	XM_001661095.2
NR_033186.1	XM_021857250.1
NR_033267.1	XM_021842420.1
NR_033359.1	XM_021849936.1
NR_033658.1	XM_021852974.1

NR_033660.2	XM_001654095.2
NR_033702.2	XM_021855077.1
NR_033757.3	XM_021840505.1
NR_033807.3	XM_021844882.1
NR_033818.2	XM_021848936.1
NR_033835.1	XM_021846720.1
NR_033838.1	XM_021850815.1
NR_033839.1	XM_021846936.1
NR_033857.1	XM_021854018.1
NR_033866.1	XM_001662650.2
NR_033876.1	XM_021843700.1
NR_033894.1	XM_001656587.2
NR_033906.2	XM_021849991.1
NR_033938.1	XM_021853477.1
NR_033955.2	XM_001652977.2
NR_033965.1	XM_001654776.2
NR_033995.2	XM_021852282.1
NR_034054.1	XM_021851357.1
NR_034095.1	XM_021856359.1
NR_034105.4	XM_001651361.2
NR_034106.3	XM_021852973.1
NR_034137.1	XR_002498640.1
NR_036433.2	XM_021843797.1
NR_036437.2	XM_021854927.1
NR_036438.1	XM_021848915.1
NR_036485.1	XM_021857021.1
NR_036489.1	XM_021852292.1
NR_036490.1	XM_001655290.2
NR_036496.1	XM_021855998.1
NR_036498.1	XM_021845778.1
NR_036503.1	XM_021840522.1
NR_036569.1	XM_021838840.1
NR_036623.2	XM_021846431.1
NR_036677.1	XM_021846367.1
NR_037141.1	XM_021841555.1
NR_037160.1	XM_021851857.1
NR_037175.1	XM_021849704.1
NR_037179.1	XM_021849908.1
NR_037602.1	XM_021854098.1
NR_037611.1	XM_001654056.2
NR_037641.2	XM_021851416.1
NR_037645.2	XM_021850010.1
NR_037646.1	XR_002500268.1
NR_037703.2	XM_021847479.1
NR_037709.1	XM_001656496.2
NR_037771.2	XM_021842619.1
NR_037801.2	XM_021853172.1
NR_037805.1	XM_021847155.1
NR_037839.1	XR_002500633.1
NR_037863.1	XM_021842876.1

NR_037878.1	XM_021855199.1
NR_037891.1	XM_021843074.1
NR_037916.2	XM_001647977.2
NR_037925.1	XM_021844897.1
NR_037932.1	XM_001653200.2
NR_037946.1	XM_021848991.1
NR_038103.1	XM_021842313.1
NR_038159.2	XM_021845330.1
NR_038204.1	XM_021851802.1
NR_038220.1	XM_021847377.1
NR_038223.1	XM_021848910.1
NR_038228.1	XM_001655415.2
NR_038238.1	XM_021845078.1
NR_038246.1	XM_021838748.1
NR_038319.1	XM_001660460.2
NR_038322.2	XM_021855448.1
NR_038327.2	XM_001647815.3
NR_038392.2	XM_021856112.1
NR_038413.1	XM_021837924.1
NR_038435.1	XM_021840122.1
NR_038439.1	XM_021851597.1
NR_038449.1	XM_021843043.1
NR_038877.1	XM_021837612.1
NR_038916.1	XM_001658356.2
NR_038924.1	XR_002501236.1
NR_038930.1	XM_021838782.1
NR_038962.1	XM_001655911.2
NR_038963.1	XM_021854890.1
NR_038981.1	XM_021842131.1
NR_038996.1	XR_002501729.1
NR_040009.2	XM_021843014.1
NR_040030.1	XM_001654830.2
NR_040040.1	XM_021838114.1
NR_040044.1	XR_002499984.1
NR_040047.1	XM_021843433.1
NR_040049.1	XM_021857691.1
NR_040067.1	XR_002502161.1
NR_040070.1	XM_021852413.1
NR_040073.1	XM_021842708.1
NR_040243.2	XM_001657934.2
NR_040244.1	XM_021848161.1
NR_040718.2	XM_021840456.1
NR_045029.1	XM_021838012.1
NR_045072.2	XM_021849774.1
NR_045114.1	XM_021849547.1
NR_045118.1	XM_001660753.2
NR_045214.1	XM_001661235.2
NR_045216.1	XR_002500006.1
NR_045481.1	XM_001650275.2
NR_045555.2	XM_001647943.2

NR_045582.2	XM_021848826.1
NR_045637.1	XM_021837664.1
NR_045639.2	XM_021846582.1
NR_045663.4	XM_001650834.2
NR_045684.2	XM_001654096.2
NR_045697.1	XM_021846106.1
NR_045770.2	XM_021840477.1
NR_046012.1	XM_021840228.1
NR_046056.2	XR_002500601.1
NR_046082.2	XR_002501751.1
NR_046085.1	XM_021841195.1
NR_046093.2	XM_021844478.1
NR_046099.1	XM_021841293.1
NR_046115.2	XM_021843311.1
NR_046188.5	XM_021841627.1
NR_046223.2	XM_021853486.1
NR_046231.1	XM_001653395.2
NR_046269.1	XM_001655556.2
NR_046351.1	XM_021857063.1
NR_046354.1	XM_021845470.1
NR_046407.1	XM_021846369.1
NR_046411.2	XR_002499107.1
NR_046447.1	XM_001651898.2
NR_046465.2	XM_001651281.2
NR_046536.1	XM_001647557.2
NR_046574.1	XM_021854815.1
NR_046579.1	XM_001654662.2
NR_046630.1	XM_001656645.2
NR_046668.1	XM_021837868.1
NR_046672.1	XM_021844129.1
NR_046681.1	XM_001655738.2
NR_046708.1	XM_001661810.2
NR_046743.1	XM_021842874.1
NR_046748.1	XM_021854529.1
NR_046766.1	XM_021856182.1
NR_046769.1	XM_021840582.1
NR_046848.1	XM_021847826.1
NR_047011.1	XR_002502734.1
NR_047013.1	XM_001655372.2
NR_047493.1	XM_021842383.1
NR_047499.1	XM_021852293.1
NR_047521.1	XM_021851546.1
NR_047542.1	XR_002499665.1
NR_047550.1	XM_021853171.1
NR_047565.3	XM_021851221.1
NR_047577.2	XM_021848136.1
NR_047592.2	XM_021854958.1
NR_047607.1	XM_021840959.1
NR_047618.1	XM_011494872.2
NR_047622.1	XM_021843625.1

NR_047654.2	XM_021852479.1
NR_047658.2	XM_021842063.1
NR_047666.1	XM_001650273.2
NR_047669.2	XM_001657475.2
NR_047689.2	XM_021849966.1
NR_048537.2	XM_001657357.2
NR_048539.2	XM_021841726.1
NR_051954.3	XM_021850260.1
NR_051955.3	XM_001656927.3
NR_051960.1	XM_001652205.3
NR_051983.1	XM_021849796.1
NR_052015.4	XM_021851457.1
NR_072987.1	XM_021845110.1
NR_073020.3	XM_001657372.2
NR_073069.1	XM_021843544.1
NR_073084.1	XM_021842853.1
NR_073108.2	XM_001651063.2
NR_073136.2	XR_002502032.1
NR_073155.1	XM_001663562.2
NR_073156.2	XM_021847592.1
NR_073179.1	XM_001660230.2
NR_073366.2	XM_001661844.2
NR_073418.1	XM_021838938.1
NR_073444.2	XM_001662980.2
NR_073488.2	XM_021854848.1
NR_073504.2	XM_001656771.2
NR_073512.1	XM_021847552.1
NR_073515.3	XM_001651631.2
NR_073516.2	XM_021846366.1
NR_073543.2	XM_021841900.1
NR_073544.2	XM_021851953.1
NR_073553.3	XM_021842357.1
NR_073560.2	XM_001654249.2
NR_073582.2	XM_001653267.2
NR_074074.2	XM_001654234.2
NR_077215.1	XM_021857725.1
NR_077225.1	XM_021848618.1
NR_077236.1	XM_021846374.1
NR_102265.2	XM_021849540.1
NR_102336.1	XM_021845219.1
NR_102347.2	XM_021838023.1
NR_102713.1	XM_001652819.2
NR_102735.1	XM_001650092.2
NR_102747.1	XM_001659423.2
NR_102753.1	XM_021854918.1
NR_102761.1	XM_021855213.1
NR_102762.1	XM_021837436.1
NR_103441.2	XM_021845345.1
NR_103456.1	XR_002498757.1
NR_103460.2	XM_001657182.2

NR_103462.1	XM_001661655.2
NR_103470.1	XM_021840472.1
NR_103501.2	XM_001650043.2
NR_103523.1	XM_021853824.1
NR_103739.2	XM_021847500.1
NR_103757.2	XM_021844670.1
NR_103772.1	XM_021849062.1
NR_103776.1	XM_021839347.1
NR_103782.2	XM_001650005.2
NR_103805.1	XM_021852865.1
NR_103812.1	XM_001663554.2
NR_103828.1	XM_021852155.1
NR_103829.1	XM_001663953.2
NR_103833.1	XM_001660752.2
NR_103867.2	XM_021840036.1
NR_103873.1	XM_021840580.1
NR_104037.2	XM_021843237.1
NR_104089.2	XM_001658753.2
NR_104090.2	XM_001662900.2
NR_104091.1	XM_021855412.1
NR_104104.3	XM_001651232.2
NR_104110.1	XM_021850604.1
NR_104112.1	XM_021849687.1
NR_104116.1	XM_021849978.1
NR_104128.2	XM_021852217.1
NR_104129.1	XR_002499736.1
NR_104130.1	XR_002500106.1
NR_104169.3	XM_001659282.2
NR_104176.1	XM_001660635.2
NR_104195.2	XM_001661236.2
NR_104220.2	XM_021840156.1
NR_104222.2	XM_021851366.1
NR_104234.2	XR_002500930.1
NR_104242.2	XM_021855847.1
NR_104267.2	XM_001656048.2
NR_104442.2	XM_021856571.1
NR_104454.1	XR_002502054.1
NR_104455.2	XR_002500170.1
NR_104456.2	XM_021855022.1
NR_104457.2	XM_021855409.1
NR_104584.2	XM_001651474.3
NR_104585.2	XM_001661374.2
NR_104592.2	XM_021854847.1
NR_104607.1	XM_021837957.1
NR_104612.2	XM_021849063.1
NR_104632.1	XM_021854691.1
NR_104639.2	XM_021843967.1
NR_104675.1	XM_021840177.1
NR_104676.2	XM_001664154.2
NR_105009.1	XR_002502908.1

NR_105016.1	XM_001655632.2
NR_105056.2	XM_021840675.1
NR_108022.1	XM_021843553.1
NR_108058.1	XM_021852485.1
NR_108063.1	XM_021849539.1
NR_108070.1	XM_001659543.2
NR_108094.1	XM_021840581.1
NR_108097.1	XM_021849014.1
NR_109780.1	XM_001653704.2
NR_109805.1	XM_001652271.2
NR_109807.2	XM_021852936.1
NR_109808.2	XM_021839004.1
NR_109826.2	XM_021846414.1
NR_109836.1	XM_021839174.1
NR_109840.2	XM_021857072.1
NR_109846.1	XM_021840805.1
NR_109850.1	XM_021840588.1
NR_109859.1	XM_021855257.1
NR_109865.1	XM_021845906.1
NR_109871.2	XM_001647652.2
NR_109875.1	XM_021845769.1
NR_109881.1	XM_021841789.1
NR_109901.2	XM_021857126.1
NR_109917.1	XM_021843824.1
NR_109933.2	XM_021848052.1
NR_109950.1	XM_021837931.1
NR_109953.1	XM_021841686.1
NR_109978.2	XM_021855013.1
NR_109997.1	XM_021854995.1
NR_110032.1	XM_021854152.1
NR_110033.1	XM_021850199.1
NR_110034.2	XM_021842798.1
NR_110043.1	XM_021844537.1
NR_110052.1	XM_021848793.1
NR_110085.1	XM_021839528.1
NR_110088.1	XM_001664144.2
NR_110120.1	XM_011495023.2
NR_110139.1	XM_001650603.2
NR_110173.2	XM_021853462.1
NR_110183.1	XM_021842849.1
NR_110190.2	XM_021849967.1
NR_110195.1	XM_021840053.1
NR_110201.1	XM_021839252.1
NR_110278.1	XM_001652614.2
NR_110280.3	XM_021838461.1
NR_110303.1	XM_021845296.1
NR_110305.1	XM_001648580.2
NR_110328.3	XM_021840270.1
NR_110433.1	XR_002501990.1
NR_110434.1	XM_021855916.1

NR_110591.1	XM_021856628.1
NR_110606.1	XM_021857538.1
NR_110622.1	XM_021842031.1
NR_110651.1	XM_021856181.1
NR_110654.1	XM_021856433.1
NR_110718.1	XM_021844421.1
NR_110723.1	XM_021840473.1
NR_110729.1	XM_021854153.1
NR_110755.1	XM_021844482.1
NR_110770.2	XM_021844643.1
NR_110796.1	XR_002499808.1
NR_110812.1	XM_021856752.1
NR_110824.1	XM_021854692.1
NR_110852.1	XM_021837381.1
NR_110867.1	XM_021845086.1
NR_110874.1	XM_021840474.1
NR_110880.1	XM_021850489.1
NR_110883.1	XM_001658443.2
NR_110917.1	XM_021845725.1
NR_110945.1	XM_021853267.1
NR_110946.1	XM_021857183.1
NR_110948.1	XM_021857174.1
NR_110951.1	XM_021850548.1
NR_110991.2	XM_021846232.1
NR_110997.1	XM_021843235.1
NR_111001.1	XM_001662198.2
NR_117097.1	XM_021857302.1
NR_120308.1	XR_002500325.1
NR_120311.1	XM_021846748.1
NR_120326.1	XM_001655244.2
NR_120331.1	XM_021840054.1
NR_120343.1	XM_021839170.1
NR_120363.1	XM_021841846.1
NR_120392.1	XR_002499755.1
NR_120470.1	XM_021849068.1
NR_120495.1	XM_021837379.1
NR_120508.1	XM_021845108.1
NR_120513.1	XM_001655633.2
NR_120520.1	XM_001663551.2
NR_120527.1	XM_001663544.2
NR_120531.2	XM_021839935.1
NR_120539.1	XM_021855258.1
NR_120546.1	XM_021852991.1
NR_120547.1	XM_021841697.1
NR_120574.1	XM_021846849.1
NR_120580.1	XM_021849969.1
NR_120599.1	XM_001651950.2
NR_120609.1	XM_021852456.1
NR_120670.1	XM_021845292.1
NR_121187.1	XM_021840192.1

NR_121192.1	XM_001652160.2
NR_121564.1	XM_021843404.1
NR_121568.2	XM_021848374.1
NR_121577.1	XM_001663538.2
NR_121605.1	XM_021837590.1
NR_121612.1	XM_021850202.1
NR_121635.1	XM_021843964.1
NR_121649.1	XM_001658838.2
NR_121654.1	XM_021852668.1
NR_121661.1	XM_021839280.1
NR_121668.1	XM_021845392.1
NR_122112.1	XM_021855544.1
NR_123738.2	XM_021855237.1
NR_125330.2	XM_001661510.2
NR_125363.1	XM_021845156.1
NR_125367.1	XM_001658073.2
NR_125384.1	XM_001661714.2
NR_125426.1	XM_001648398.2
NR_125724.1	XM_021856533.1
NR_125729.1	XM_001660113.2
NR_125732.2	XM_021852455.1
NR_125763.1	XM_021852306.1
NR_125810.1	XM_021857175.1
NR_125821.1	XM_021843741.1
NR_125826.1	XM_021854297.1
NR_125848.1	XM_021857340.1
NR_125849.1	XM_001659911.2
NR_125858.1	XM_001654567.2
NR_125859.1	XM_021840340.1
NR_125860.1	XM_021842925.1
NR_125880.1	XM_021842428.1
NR_125881.1	XM_021837830.1
NR_125921.1	XM_021847503.1
NR_125932.1	XM_021843440.1
NR_125962.1	XM_021838552.1
NR_125970.1	XM_021849060.1
NR_125976.1	XM_021849777.1
NR_125995.1	XM_001651422.2
NR_126020.1	XM_021842907.1
NR_126025.1	XM_021841861.1
NR_126037.2	XM_021840782.1
NR_126054.1	XR_002499913.1
NR_126062.1	XM_021837628.1
NR_126168.1	XM_021855436.1
NR_126331.1	XM_021854957.1
NR_126333.1	XM_021848283.1
NR_126374.1	XM_021846225.1
NR_126406.1	XM_021843140.1
NR_126453.1	XM_021855668.1
NR_126500.1	XM_001657363.2

NR_126523.1	XM_021838421.1
NR_126561.1	XM_021837632.1
NR_130126.2	XM_021853589.1
NR_130130.2	XM_021850595.1
NR_130149.2	XM_021841107.1
NR_130728.1	XM_021841088.1
NR_130761.2	XM_021849028.1
NR_130766.1	XM_021845674.1
NR_130771.1	XM_021855433.1
NR_130772.1	XM_001663302.2
NR_130773.1	XM_001661701.2
NR_130927.2	XM_001651268.2
NR_130940.1	XM_021852453.1
NR_131184.1	XR_002502046.1
NR_131340.2	XM_021841569.1
NR_131753.2	XM_021849545.1
NR_131774.2	XM_001651496.2
NR_132102.1	XM_021841118.1
NR_132273.1	XM_021852450.1
NR_132338.1	XM_021857299.1
NR_132373.1	XM_021838849.1
NR_132380.1	XM_021845062.1
NR_132408.1	XM_021844644.1
NR_132415.1	XR_002499788.1
NR_132647.2	XM_001657915.2
NR_132740.2	XM_021856089.1
NR_132780.1	XM_021857594.1
NR_132970.1	XM_021857702.1
NR_132987.1	XM_021843796.1
NR_133566.1	XR_002502830.1
NR_133636.2	XM_001647913.2
NR_133637.2	XR_002500924.1
NR_133914.1	XR_002502264.1
NR_133940.1	XM_021840465.1
NR_133947.1	XR_002499756.1
NR_134238.1	XM_001658099.2
NR_134254.1	XM_021856066.1
NR_134266.1	XM_021842624.1
NR_134273.1	XM_021843023.1
NR_134275.1	XM_021839068.1
NR_134295.1	XR_002502490.1
NR_134299.2	XM_021841620.1
NR_134305.1	XM_021846172.1
NR_134332.1	XM_021850646.1
NR_134459.1	XM_001658379.2
NR_134464.2	XM_001660215.2
NR_134475.2	XM_021840883.1
NR_134529.2	XM_021844154.1
NR_134563.3	XM_021843743.1
NR_134577.1	XM_001662574.2

NR_134583.1	XM_021838166.1
NR_134598.1	XM_001657919.2
NR_134611.1	XM_021840858.1
NR_134615.1	XM_021854160.1
NR_134632.1	XM_001656434.2
NR_134637.1	XR_002500208.1
NR_134669.1	XM_021838978.1
NR_134872.2	XM_001654840.2
NR_134873.1	XM_021844933.1
NR_134878.1	XM_021848353.1
NR_134881.1	XM_021846192.1
NR_134893.2	XM_021838399.1
NR_134896.2	XM_021854831.1
NR_134924.1	XM_001660507.2
NR_134931.1	XM_021846067.1
NR_134976.3	XM_001650806.2
NR_134989.2	XM_021847047.1
NR_135049.1	XM_021837579.1
NR_135068.2	XM_021846229.1
NR_135076.1	XM_021846171.1
NR_135096.1	XM_021845905.1
NR_135108.1	XM_001659080.2
NR_135127.1	XM_001657986.2
NR_135132.1	XM_001652414.2
NR_135157.2	XM_021843417.1
NR_135166.2	XM_001651335.2
NR_135170.1	XM_021855067.1
NR_135174.1	XM_021847985.1
NR_135176.1	XM_001650166.2
NR_135203.1	XR_002499232.1
NR_135224.1	XM_021843071.1
NR_135238.1	XM_001662279.2
NR_135267.3	XM_021854501.1
NR_135481.1	XM_021847374.1
NR_135486.2	XM_021846041.1
NR_135504.1	XM_001649177.2
NR_135527.1	XM_001663340.2
NR_135532.1	XM_021853310.1
NR_135537.1	XM_021843261.1
NR_135551.1	XM_021847052.1
NR_135557.1	XM_021853884.1
NR_135616.1	XM_021846948.1
NR_135643.1	XR_002499516.1
NR_135653.2	XM_001652763.2
NR_135666.1	XM_021857114.1
NR_135674.1	XM_021857717.1
NR_135682.1	XM_021838427.1
NR_135687.1	XM_021838168.1
NR_135758.2	XM_021847931.1
NR_135764.1	XM_021849799.1

NR_135807.2	XM_021855852.1
NR_135828.1	XM_021843748.1
NR_135847.2	XM_001651427.2
NR_135855.2	XM_001650246.2
NR_136174.2	XR_002501952.1
NR_136178.1	XM_021857719.1
NR_136195.1	XM_021851505.1
NR_136201.1	XM_001660350.2
NR_136230.2	XM_021841672.1
NR_136242.2	XM_021840294.1
NR_136245.1	XM_021846745.1
NR_136253.1	XM_021843794.1
NR_136256.1	XR_002503246.1
NR_136272.1	XM_001655872.3
NR_136276.2	XM_021849963.1
NR_136290.1	XM_021854990.1
NR_136300.1	XM_021852192.1
NR_136301.1	XR_002502101.1
NR_136318.1	XR_002499965.1
NR_136331.3	XM_021856688.1
NR_136403.1	XM_021855919.1
NR_136518.1	XM_021837919.1
NR_136552.1	XM_021850766.1
NR_136565.2	XM_021845726.1
NR_136569.2	XM_021845323.1
NR_136586.2	XR_002499531.1
NR_136635.2	XM_001663091.2
NR_136644.1	XM_001649286.2
NR_136645.2	XM_001649197.2
NR_136700.2	XM_021857330.1
NR_136708.2	XM_021839061.1
NR_136719.2	XM_021844128.1
NR_136721.2	XM_001661898.2
NR_136746.2	XM_021845503.1
NR_136748.1	XM_021839891.1
NR_137185.1	XM_021846330.1
NR_137189.1	XM_001663326.2
NR_137194.1	XM_021845388.1
NR_137281.2	XM_001658958.2
NR_137425.1	XM_021842631.1
NR_137431.1	XM_021850607.1
NR_138035.2	XM_021842982.1
NR_138070.2	XM_021846422.1
NR_138073.2	XM_021853912.1
NR_138076.2	XM_021851801.1
NR_138077.2	XM_021837965.1
NR_138091.1	XM_001660119.2
NR_138116.2	XR_002500293.1
NR_138121.2	XM_001649105.2
NR_138123.2	XM_021853913.1

NR_138130.2	XM_001652027.2
NR_138256.2	XM_011494848.2
NR_138259.1	XM_001654687.2
NR_138426.2	XM_001656014.2
NR_138467.2	XM_021857384.1
NR_138609.2	XM_001651600.2
NR_138612.2	XM_001658187.2
NR_144335.2	XM_001654383.2
NR_144339.2	XM_021843622.1
NR_144380.2	XM_021855430.1
NR_144399.2	XM_021842870.1
NR_144524.2	XM_021849733.1
NR_144530.1	XM_021841568.1
NR_144540.1	XM_021839859.1
NR_144635.2	XM_001653367.2
NR_144759.2	XM_021848790.1
NR_144943.2	XM_021851711.1
NR_145129.1	XM_021849561.1
NR_145441.2	XM_021855402.1
NR_145449.1	XM_021853822.1
NR_145469.1	XM_021855328.1
NR_145502.2	XM_021850999.1
NR_145677.1	XM_021855401.1
NR_145683.2	XR_002500107.1
NR_145684.2	XM_021847487.1
NR_145685.2	XM_001663392.2
NR_145697.1	XM_021844302.1
NR_145704.1	XM_001653015.2
NR_145706.1	XM_011495220.2
NR_145819.1	XM_021846552.1
NR_145823.1	XM_001660882.2
NR_146091.2	XM_021851712.1
NR_146100.2	XM_001660575.2
NR_146109.1	XM_021855426.1
NR_146119.1	XM_001649843.2
NR_146128.2	XM_021842961.1
NR_146134.2	XM_011494916.2
NR_146143.3	XM_021839711.1
NR_146145.2	XM_021848310.1
NR_146167.2	XM_021857671.1
NR_146174.2	XM_021853210.1
NR_146176.2	XM_021844272.1
NR_146223.1	XR_002501800.1
NR_146233.2	XM_021854223.1
NR_146275.1	XM_021844863.1
NR_146279.1	XM_021845917.1
NR_146285.2	XM_001649679.2
NR_146312.1	XM_021844653.1
NR_146323.1	XR_002498996.1
NR_146341.2	XM_021840063.1

NR_146342.2	XM_021852253.1
NR_146384.1	XM_001648269.2
NR_146438.1	XM_001654837.2
NR_146439.1	XM_021839892.1
NR_146442.1	XM_021845282.1
NR_146456.1	XM_021850943.1
NR_146465.2	XR_002498746.1
NR_146476.1	XM_021851556.1
NR_146504.1	XM_021844455.1
NR_146507.1	XR_002500980.1
NR_146512.2	XM_021846676.1
NR_146588.1	XM_001658961.2
NR_146592.1	XM_021840888.1
NR_146598.2	XM_021851363.1
NR_146647.1	XM_021849033.1
NR_146717.1	XM_021849778.1
NR_146720.2	XM_021838327.1
NR_146733.1	XM_001657543.2
NR_146735.1	XM_021850252.1
NR_146736.1	XM_021850257.1
NR_146740.2	XM_021844316.1
NR_146763.2	XM_001658078.2
NR_146769.1	XM_021849429.1
NR_146771.1	XM_021844900.1
NR_146778.2	XM_021855428.1
NR_146866.1	XM_021839786.1
NR_146868.1	XM_021857373.1
NR_146879.2	XM_021843932.1
NR_146896.1	XM_021839710.1
NR_146905.1	XM_021845162.1
NR_146908.1	XM_021847378.1
NR_146957.1	XM_021846677.1
NR_146960.1	XM_021855484.1
NR_146965.2	XM_021847287.1
NR_146980.2	XM_021845053.1
NR_146984.3	XM_021838323.1
NR_147004.1	XM_021851713.1
NR_147021.2	XM_021846679.1
NR_147052.2	XM_021845218.1
NR_147060.2	XM_001653111.2
NR_147061.1	XM_021853316.1
NR_147081.2	XM_021850418.1
NR_147085.2	XM_001663525.2
NR_147095.2	XM_021839856.1
NR_147132.2	XR_002499404.1
NR_147136.2	XM_021846202.1
NR_147170.1	XM_001653553.2
NR_147171.2	XM_001661539.2
NR_147175.1	XM_001652665.2
NR_147186.1	XM_001656168.2

NR_147255.2	XM_021851849.1
NR_147408.1	XM_021849911.1
NR_147452.2	XM_001650224.2
NR_147506.1	XM_021840886.1
NR_147507.1	XM_001659537.2
NR_147791.2	XM_021845325.1
NR_147844.1	XM_021842225.1
NR_147910.2	XM_001662162.2
NR_147924.2	XM_021838344.1
NR_147928.2	XM_021849438.1
NR_147942.2	XM_021848523.1
NR_147986.1	XM_021837689.1
NR_148002.2	XM_001653080.2
NR_148203.1	XM_021857661.1
NR_148241.1	XM_001655945.2
NR_148367.1	XM_001654671.2
NR_148373.1	XM_021846751.1
NR_148384.2	XM_021849218.1
NR_148413.2	XM_021853311.1
NR_148440.2	XM_001658397.2
NR_148447.1	XM_021847933.1
NR_148469.2	XM_021852179.1
NR_148478.2	XM_021838610.1
NR_148508.1	XM_021844806.1
NR_148511.2	XM_021845000.1
NR_148921.2	XM_001652876.3
NR_148977.1	XM_021856829.1
NR_148984.2	XM_021854441.1
NR_148987.2	XM_021847634.1
NR_148995.1	XM_021841532.1
NR_149033.1	XM_021850610.1
NR_149038.1	XM_001649562.2
NR_149039.1	XM_001656886.2
NR_149061.2	XM_001659323.2
NR_149074.1	XR_002500933.1
NR_149086.1	XM_001657661.2
NR_149093.2	XM_001656371.2
NR_149106.1	XM_001658843.2
NR_149109.1	XM_021853946.1
NR_149165.2	XM_021843144.1
NR_151483.1	XM_001655388.2
NR_152570.1	XR_002502226.1
NR_152576.1	XM_001657559.2
NR_152746.1	XM_021843898.1
NR_152799.1	XM_021847818.1
NR_152811.1	XR_002501025.1
NR_152813.1	XM_021848530.1
NR_152870.1	XM_021845037.1
NR_156490.1	XM_001656162.2
NR_156732.1	XM_021843072.1

NR_157086.1	XM_021844274.1
NR_157112.2	XM_021845533.1
NR_157587.1	XM_021847288.1
NR_157804.1	XM_021839989.1
NR_157807.1	XM_001658951.2
NR_157824.1	XM_021846840.1
NR_157826.1	XM_021850498.1
NR_157842.1	XM_021853725.1
NR_158184.1	XM_021843770.1
NR_158193.1	XM_021844016.1
NR_158197.1	XM_021837907.1
NR_158215.1	XM_021854685.1
NR_158567.1	XM_021843636.1
NR_158629.1	XM_021843934.1
NR_158768.1	XR_002502055.1
NR_158770.1	XM_021840149.1
NR_158772.1	XM_021845324.1
NR_158778.1	XM_021848437.1
NR_159362.2	XM_021841300.1
NR_159369.2	XM_021844525.1
NR_159371.1	XM_021847453.1
NR_159376.2	XM_021853137.1
NR_159386.1	XM_021840508.1
NR_159387.1	XM_001650559.2
NR_159403.1	XM_021851855.1
NR_159737.1	XM_021846935.1
NR_159738.1	XM_021846345.1
NR_159806.1	XM_001663167.2
NR_159941.1	XM_001650162.2
NR_159966.1	XM_021852500.1
NR_160296.2	XM_021846495.1
NR_160413.1	XM_021856074.1
NR_160426.1	XR_002500245.1
NR_160551.1	XM_021845344.1
NR_160657.1	XM_021840896.1
NR_160705.1	XM_021857300.1
NR_160708.1	XM_001663119.2
NR_160718.1	XM_021854075.1
NR_160743.1	XM_001657747.2
NR_160745.1	XR_002500815.1
NR_160777.1	XM_011494841.2
NR_160778.1	XM_021841862.1
NR_160783.1	XM_021838467.1
NR_160915.1	XR_002499038.1
NR_160979.1	XR_002499811.1
NR_160980.1	XM_021853109.1
NR_161186.1	XM_021845959.1
NR_161203.1	XM_021853258.1
NR_161233.1	XM_001657351.2
NR_161292.1	XM_001653290.2

NR_161371.1	XM_021849428.1
NR_161435.2	XM_021853950.1
NR_161466.1	XM_021854801.1
NR_161467.1	XM_021841061.1
NR_163149.1	XM_001655108.2
NR_163195.1	XM_021851619.1
NR_163241.1	XM_021838095.1
NR_163266.1	XM_021837438.1
NR_163392.1	XR_002498665.1
NR_163410.1	XM_021855765.1
NR_163466.1	XM_021841283.1
NR_163481.1	XM_021850559.1
NR_163889.1	XR_002500979.1
NR_163917.1	XM_021847796.1
NR_163981.1	XM_021847767.1
NR_163993.1	XM_021847665.1
NR_164009.1	XM_021847504.1
NR_164155.1	XR_002502193.1
NR_164337.1	XM_021843164.1
NR_164338.1	XR_002499922.1
NR_164659.1	XM_001659258.2
NR_164700.2	XM_021839995.1
NR_164706.1	XM_001656401.2
NR_164715.1	XM_001660646.2
NR_164732.1	XM_021839712.1
NR_164747.1	XM_021841813.1
NR_164753.1	XM_021851436.1
NR_164767.1	XM_021837754.1
NR_164774.1	XM_021849817.1
NR_164779.1	XM_021837278.1
NR_164783.1	XM_021857704.1
NR_164787.1	XM_001659861.2
NR_164790.1	XM_021852960.1
NR_164810.1	XM_021840182.1
NR_164822.1	XM_001662731.1
NR_165108.1	XR_002500884.1
NR_165111.1	XR_002500542.1
NR_165115.1	XM_001653675.2
NR_165140.1	XM_021843746.1
NR_165235.1	XM_021844470.1
NR_165295.1	XM_021842851.1
NR_165336.1	XM_001663729.2
NR_165344.1	XM_021845471.1
NR_165367.1	XM_021839249.1
NR_165400.1	XM_001660345.2
NR_165401.1	XM_021844825.1
NR_165409.1	XM_021843769.1
NR_165431.1	XR_002500024.1
NR_165439.1	XM_021840806.1
NR_165630.1	XM_021842879.1

NR_165650.1	XM_001654242.2
NR_166069.1	XM_021838796.1
NR_166196.1	XM_021848452.1
NR_166825.1	XM_021839000.1
NR_166844.1	XM_021851013.1
NR_167706.1	XM_021846430.1
NR_167709.1	XM_021841651.1
NR_167744.1	XM_021853731.1
NR_167745.1	XM_021849066.1
NR_167888.1	XM_021850913.1
NR_167907.1	XM_021845030.1
NR_167910.1	XM_021847870.1
NR_167931.1	XM_001648285.2
NR_167974.1	XM_021856023.1
NR_167991.1	XM_021853243.1
NR_168050.1	XM_001650796.2
NR_168136.1	XM_021839469.1
NR_168365.1	XM_021843432.1
NR_168385.1	XM_021849217.1
NR_168386.1	XM_021838017.1
NR_168437.1	XM_021851617.1
NR_168445.1	XM_001658196.2
NR_168454.1	XM_001661563.2
NR_168520.1	XM_021844472.1
NR_169221.1	XM_021856787.1
NR_169231.1	XM_021841576.1
NR_169511.1	XR_002499182.1
NR_169524.1	XM_001660542.2
NR_169541.1	XM_021856912.1
NR_169542.1	XM_021839841.1
NR_169543.1	XM_021857721.1
NR_169570.1	XM_021845021.1
NR_169578.1	XM_021857563.1
NR_169585.1	XM_021846046.1
NR_169586.1	XM_021857240.1
NR_169640.1	XM_021839813.1
NR_169733.1	XM_021854856.1
NR_169740.1	XM_021838712.1
NR_169752.1	XM_021856811.1
NR_169768.1	XM_021852501.1
NR_169790.1	XM_021846332.1
NR_169835.1	XM_021840348.1
NR_169839.1	XM_021843611.1
NR_169846.1	XM_001661082.2
NR_169859.1	XM_021857433.1
NR_169880.1	XM_021839956.1
NR_170172.1	XM_001651860.2
NR_170230.1	XM_021839451.1
NR_170244.1	XM_021849577.1
NR_170268.1	XM_021854699.1

NR_170274.1	XM_021841924.1
NR_170318.1	XM_021845947.1
NR_170357.1	XM_021842046.1
NR_170364.1	XM_021837874.1
NR_170571.1	XM_021840135.1
NR_170584.1	XM_021844541.1
NR_170613.1	XM_021838649.1
NR_170627.1	XM_021850618.1
NR_170680.1	XM_001660494.2
NR_170691.1	XM_021840772.1
NR_170709.1	XR_002501689.1
NR_170867.1	XM_021839483.1
NR_170886.1	XM_021845399.1
NR_170903.1	XM_021843091.1
NR_170907.1	XM_021843403.1
NR_170928.1	XM_021851367.1
NR_170930.1	XM_021840292.1
NR_170960.1	XM_021848241.1
NR_170966.1	XM_021854074.1
NR_170970.1	XM_001658848.3
NR_170978.1	XM_001658253.2
NR_170982.1	XM_001655641.2
NR_171035.1	XM_001649981.2
NR_171164.1	XM_021851732.1
XM_005245005.2	XM_021840865.1
XM_005245024.2	XM_021839225.1
XM_005245048.3	XM_021845054.1
XM_005245267.4	XR_002501237.1
XM_005245314.2	XM_021848813.1
XM_005245325.4	XM_001647527.2
XM_005245348.4	XM_001650229.2
XM_005245453.1	XM_021854905.1
XM_005245508.3	XM_021844511.1
XM_005245532.4	XM_021849802.1
XM_005246141.4	XM_021846877.1
XM_005246212.3	XM_021838098.1
XM_005246255.2	XM_021854294.1
XM_005246446.3	XM_021844651.1
XM_005246456.1	XM_001662815.3
XM_005246489.4	XM_021840595.1
XM_005246505.2	XR_002502118.1
XM_005246593.2	XM_021847949.1
XM_005246812.2	XM_001654563.2
XM_005247248.5	XM_021853173.1
XM_005247284.3	XM_021842497.1
XM_005247309.2	XM_021850190.1
XM_005247347.4	XM_021847442.1
XM_005247372.4	XM_001654939.2
XM_005247458.5	XM_001654938.2
XM_005247512.1	XM_001654024.2

XM_005247538.3	XM_021840885.1
XM_005247594.5	XM_021844123.1
XM_005247982.3	XM_021848058.1
XM_005248028.5	XM_001654401.2
XM_005248060.1	XM_001657332.2
XM_005248231.3	XM_021843150.1
XM_005248376.4	XM_021856056.1
XM_005248504.4	XM_021845286.1
XM_005248612.3	XR_002501564.1
XM_005248700.3	XM_021841149.1
XM_005248843.4	XM_001656857.2
XM_005248893.3	XM_021843930.1
XM_005248901.3	XM_001663312.2
XM_005248951.4	XM_021843163.1
XM_005248976.1	XM_021842696.1
XM_005248999.2	XM_001655768.2
XM_005249010.2	XM_001652794.2
XM_005249079.2	XM_001659206.2
XM_005249184.5	XR_002499135.1
XM_005249218.1	XM_001654379.2
XM_005249295.1	XM_001649170.2
XM_005249315.3	XM_021838392.1
XM_005249353.4	XM_021854168.1
XM_005249403.3	XM_021840813.1
XM_005249531.1	XM_021842080.1
XM_005249584.3	XM_021857505.1
XM_005249729.1	XM_021838138.1
XM_005249883.5	XM_021853250.1
XM_005249916.1	XM_021844524.1
XM_005249952.4	XM_001650451.2
XM_005250129.4	XM_011494761.2
XM_005250405.2	XM_021847156.1
XM_005250493.1	XM_021846640.1
XM_005250502.2	XM_021846625.1
XM_005250509.4	XM_021849043.1
XM_005250574.3	XM_021839293.1
XM_005250772.3	XM_021840520.1
XM_005250773.3	XM_021846341.1
XM_005250978.3	XM_021857210.1
XM_005251025.5	XM_021854518.1
XM_005251040.4	XM_001659611.2
XM_005251121.2	XM_021853073.1
XM_005251135.4	XM_001656668.2
XM_005251590.1	XM_021839454.1
XM_005251783.3	XM_001655274.2
XM_005251826.2	XM_001657991.2
XM_005251973.4	XM_021851973.1
XM_005251987.4	XM_021841098.1
XM_005252067.4	XM_021838165.1
XM_005252151.1	XM_021851805.1

XM_005252208.2	XM_021843929.1
XM_005252369.3	XM_021849614.1
XM_005252627.3	XM_011494970.2
XM_005252669.3	XM_021855759.1
XM_005252787.2	XM_021841302.1
XM_005252788.2	XM_021842642.1
XM_005252921.3	XM_021845887.1
XM_005253138.5	XM_001655520.3
XM_005253168.3	XM_001657469.2
XM_005253196.3	XM_021843145.1
XM_005253229.2	XR_002502336.1
XM_005253334.3	XM_021856426.1
XM_005253698.4	XM_001657918.2
XM_005253824.3	XM_021847490.1
XM_005253834.4	XR_002499409.1
XM_005253908.4	XM_021852100.1
XM_005253944.4	XM_021848814.1
XM_005254235.3	XM_021848750.1
XM_005254430.5	XM_021852815.1
XM_005254481.3	XM_021841230.1
XM_005254529.4	XM_021838873.1
XM_005254646.2	XR_002501692.1
XM_005254671.2	XM_001648890.2
XM_005254685.4	XM_021856005.1
XM_005254795.5	XM_001647909.2
XM_005254916.3	XM_021854058.1
XM_005254934.4	XM_021854807.1
XM_005255077.1	XM_001657110.2
XM_005255182.3	XM_001657964.2
XM_005255210.2	XM_021846340.1
XM_005255304.4	XM_021849484.1
XM_005255539.3	XM_021854377.1
XM_005255558.2	XM_021845964.1
XM_005255725.5	XM_021857593.1
XM_005255741.4	XM_021841811.1
XM_005255952.5	XM_021852659.1
XM_005256255.2	XM_021852445.1
XM_005256399.5	XM_001655822.2
XM_005256424.2	XM_001660628.2
XM_005256629.1	XM_021838872.1
XM_005256678.5	XM_021841355.1
XM_005256770.1	XM_021851593.1
XM_005256784.4	XR_002499353.1
XM_005256856.3	XM_021856553.1
XM_005256866.5	XM_021844322.1
XM_005256905.2	XM_001652148.2
XM_005257019.1	XM_001661173.2
XM_005257076.3	XM_001654141.2
XM_005257098.3	XM_021856918.1
XM_005257126.4	XM_001649603.2

XM_005257161.3	XR_002501855.1
XM_005257187.4	XM_021843643.1
XM_005257365.4	XR_002499205.1
XM_005257370.4	XM_021846044.1
XM_005257373.4	XM_001656630.2
XM_005257468.5	XR_002499626.1
XM_005257512.2	XM_021852990.1
XM_005257703.1	XM_021840131.1
XM_005257774.4	XM_021850647.1
XM_005257859.4	XM_021841723.1
XM_005258315.5	XM_021852595.1
XM_005258361.3	XM_001662106.2
XM_005258459.4	XR_002502177.1
XM_005258620.2	XM_001653393.2
XM_005258710.5	XM_001647886.2
XM_005258746.3	XM_011494796.2
XM_005258883.2	XR_002500387.1
XM_005259153.3	XM_021840622.1
XM_005259215.4	XM_021849409.1
XM_005259323.1	XM_001648284.2
XM_005259327.3	XM_021848320.1
XM_005259467.1	XM_021845755.1
XM_005259813.4	XM_021848086.1
XM_005259834.1	XR_002502274.1
XM_005259913.2	XM_001658819.2
XM_005259941.4	XM_021852033.1
XM_005259951.4	XM_021838485.1
XM_005260183.2	XM_021854528.1
XM_005260192.2	XM_021854495.1
XM_005260195.4	XM_001652422.2
XM_005260197.4	XM_001663850.2
XM_005260382.4	XM_001648250.2
XM_005260407.4	XM_021840015.1
XM_005260465.3	XM_021844946.1
XM_005260467.4	XM_001654923.2
XM_005260501.5	XM_021847076.1
XM_005260749.4	XM_021841894.1
XM_005260978.4	XM_021839923.1
XM_005260979.2	XM_001651872.2
XM_005260997.5	XM_021856058.1
XM_005261068.3	XM_021851708.1
XM_005261121.3	XM_021846972.1
XM_005261135.3	XM_021841359.1
XM_005261168.4	XM_021855398.1
XM_005261171.3	XM_021847310.1
XM_005261415.3	XM_001653958.2
XM_005261430.2	XR_002500025.1
XM_005261525.4	XM_021857228.1
XM_005261695.1	XM_001653029.2
XM_005261705.4	XM_021850713.1

XM_005261986.4	XM_021847632.1
XM_005262155.4	XM_021857674.1
XM_005262310.3	XM_021842960.1
XM_005262315.3	XM_021857519.1
XM_005262384.4	XR_002500751.1
XM_005262472.2	XM_001656487.3
XM_005262489.5	XR_002502186.1
XM_005262750.4	XM_021857375.1
XM_005262772.3	XM_021843413.1
XM_005262774.4	XM_001655947.2
XM_005262937.4	XM_021852147.1
XM_005263031.4	XM_001656585.2
XM_005263077.4	XM_001653975.2
XM_005263166.4	XM_021844418.1
XM_005263372.3	XM_021844008.1
XM_005263403.3	XM_021840623.1
XM_005263449.5	XM_021843768.1
XM_005263462.4	XM_021852585.1
XM_005263547.3	XM_021854703.1
XM_005263548.3	XM_021842230.1
XM_005263629.3	XM_021851194.1
XM_005263710.2	XM_021847906.1
XM_005263862.4	XM_021837875.1
XM_005263921.4	XM_021852117.1
XM_005264203.3	XM_021850603.1
XM_005264230.4	XM_021856134.1
XM_005264283.2	XM_021840578.1
XM_005264318.3	XM_021854975.1
XM_005264421.3	XM_021840785.1
XM_005264459.2	XM_021847991.1
XM_005264487.2	XM_021855444.1
XM_005264843.4	XM_021847174.1
XM_005264921.5	XM_021852764.1
XM_005264940.4	XM_021845965.1
XM_005264968.1	XM_021841493.1
XM_005265098.4	XR_002501047.1
XM_005265379.3	XM_021846945.1
XM_005265587.5	XM_021853326.1
XM_005265653.4	XM_021854400.1
XM_005265673.4	XM_011495202.2
XM_005265967.2	XM_021847293.1
XM_005266050.4	XR_002499905.1
XM_005266419.1	XM_021839862.1
XM_005266460.2	XM_021853030.1
XM_005266461.3	XM_021850761.1
XM_005266464.3	XR_002499617.1
XM_005266583.5	XM_021838092.1
XM_005266642.3	XM_021839314.1
XM_005266970.1	XM_001657600.2
XM_005266983.4	XM_021850327.1

XM_005266984.4	XM_001652859.2
XM_005267135.3	XM_001658478.3
XM_005267179.4	XM_001656854.2
XM_005267325.5	XR_002499915.1
XM_005267406.5	XM_021837242.1
XM_005267510.1	XM_001653593.2
XM_005267537.4	XM_021841417.1
XM_005267709.3	XM_001662893.2
XM_005267833.5	XM_001663446.2
XM_005267854.1	XM_021848829.1
XM_005267890.5	XM_001652292.2
XM_005267988.3	XM_021839154.1
XM_005267991.3	XM_001655512.2
XM_005268111.3	XM_001659699.2
XM_005268128.1	XM_001652543.3
XM_005268372.4	XM_021839999.1
XM_005268377.4	XM_001652075.2
XM_005268447.4	XM_021837702.1
XM_005268477.1	XR_002500305.1
XM_005268493.2	XM_021840205.1
XM_005268508.4	XM_021842343.1
XM_005268556.2	XM_011495423.2
XM_005268707.4	XM_001657252.2
XM_005268730.3	XM_021847183.1
XM_005268738.3	XR_002502086.1
XM_005268739.4	XM_021841631.1
XM_005268757.4	XM_021852584.1
XM_005268927.2	XM_021838463.1
XM_005268997.2	XM_001650331.2
XM_005269011.3	XM_001649612.2
XM_005269051.3	XM_001647841.2
XM_005269125.2	XM_021850959.1
XM_005269133.2	XM_021844910.1
XM_005269207.1	XM_001655529.2
XM_005269224.5	XM_021856048.1
XM_005269476.4	XM_021847739.1
XM_005269478.4	XM_001660865.2
XM_005269509.3	XM_021837938.1
XM_005269655.3	XM_021837863.1
XM_005269742.1	XM_021846043.1
XM_005269890.1	XM_001661198.2
XM_005270026.4	XM_021855798.1
XM_005270148.2	XM_021845320.1
XM_005270231.3	XM_001663007.2
XM_005270241.4	XM_021840934.1
XM_005270360.2	XM_021837822.1
XM_005270562.3	XM_001658325.2
XM_005270777.2	XM_021853633.1
XM_005270779.4	XM_001653309.2
XM_005271103.4	XM_021850125.1

XM_005271107.2	XM_001653339.2
XM_005271110.2	XM_021841832.1
XM_005271113.5	XM_021857590.1
XM_005271149.4	XR_002501219.1
XM_005271223.3	XM_021845334.1
XM_005271235.3	XR_002500689.1
XM_005271260.5	XM_021841481.1
XM_005271274.4	XM_021839812.1
XM_005271355.3	XM_021856011.1
XM_005271471.3	XM_021839576.1
XM_005271485.3	XM_021840518.1
XM_005271721.5	XM_021842056.1
XM_005271745.4	XR_002501100.1
XM_005271793.4	XM_001657971.2
XM_005271835.4	XR_002498710.1
XM_005271894.3	XM_021851658.1
XM_005272016.4	XM_001661163.2
XM_005272048.4	XM_001662594.2
XM_005272105.4	XM_021845149.1
XM_005272109.5	XM_001663115.2
XM_005272118.4	XM_021848059.1
XM_005272572.4	XM_001658034.2
XM_005273165.4	XM_021841050.1
XM_005273322.4	XM_001658079.2
XM_005273481.3	XM_021844499.1
XM_005273585.4	XM_021850958.1
XM_005273611.4	XM_021839416.1
XM_005273613.4	XM_021849505.1
XM_005273801.4	XM_001650263.2
XM_005273976.2	XM_021852784.1
XM_005274161.1	XR_002502497.1
XM_005274177.3	XM_021852396.1
XM_005274368.2	XR_002499623.1
XM_005274521.4	XR_002499167.1
XM_005274578.2	XM_021843500.1
XM_005274716.3	XM_001656134.2
XM_005274742.2	XM_021847051.1
XM_005274778.3	XM_001658072.2
XM_005276753.5	XM_021844902.1
XM_005276755.5	XM_021841199.1
XM_005276975.5	XM_021848343.1
XM_005278250.5	XR_002500178.1
XM_005278279.2	XR_002500150.1
XM_006710346.3	XM_021842623.1
XM_006710394.4	XM_021839651.1
XM_006710426.3	XM_021843167.1
XM_006710474.3	XM_021839010.1
XM_006710477.3	XM_021838656.1
XM_006710585.3	XM_001661849.2
XM_006710618.3	XM_021855814.1

XM_006710631.3	XM_001664161.2
XM_006710731.1	XM_021853057.1
XM_006710859.1	XM_021852115.1
XM_006710922.1	XM_001661280.2
XM_006710995.2	XM_021839907.1
XM_006711174.2	XM_021839768.1
XM_006711221.3	XR_002502937.1
XM_006711253.2	XM_021853452.1
XM_006711356.3	XM_021857083.1
XM_006711567.4	XM_021850573.1
XM_006711643.3	XM_021838636.1
XM_006711929.3	XM_001657463.2
XM_006711995.3	XM_021848943.1
XM_006712021.3	XM_021839619.1
XM_006712030.4	XM_021844223.1
XM_006712094.3	XM_021857385.1
XM_006712182.3	XM_001654420.2
XM_006712196.3	XM_021847296.1
XM_006712208.3	XM_001651946.2
XM_006712286.3	XM_021843566.1
XM_006712301.2	XR_002500445.1
XM_006712336.3	XM_021857432.1
XM_006712417.1	XM_021840491.1
XM_006712521.4	XM_001658261.2
XM_006712551.1	XM_011494873.2
XM_006712639.3	XM_021843168.1
XM_006712797.3	XM_021849506.1
XM_006712799.3	XR_002499491.1
XM_006712802.1	XM_021846047.1
XM_006712946.3	XM_021847096.1
XM_006713041.1	XM_021854687.1
XM_006713204.3	XM_021853799.1
XM_006713318.3	XM_021851193.1
XM_006713417.3	XM_021850288.1
XM_006713435.3	XM_021845267.1
XM_006713472.1	XM_011495268.2
XM_006713603.2	XM_021853199.1
XM_006713684.3	XM_021848116.1
XM_006713760.4	XM_021843439.1
XM_006713831.4	XM_001661372.2
XM_006713986.3	XM_021854547.1
XM_006714116.4	XM_021839899.1
XM_006714158.1	XM_021841015.1
XM_006714165.3	XM_001656555.2
XM_006714177.3	XM_021840757.1
XM_006714231.2	XM_001655654.2
XM_006714306.4	XM_021857511.1
XM_006714420.3	XM_001656102.2
XM_006714464.3	XM_021857592.1
XM_006714532.3	XM_001649091.2

XM_006714587.4	XM_001656675.2
XM_006714675.4	XM_001651525.2
XM_006714741.2	XM_021841719.1
XM_006714792.2	XM_021838054.1
XM_006714921.3	XM_021841413.1
XM_006714975.1	XR_002500754.1
XM_006715052.3	XM_021850127.1
XM_006715268.2	XM_021840756.1
XM_006715270.1	XM_001660287.2
XM_006715279.2	XM_021851641.1
XM_006715349.4	XM_021840784.1
XM_006715426.3	XM_021844547.1
XM_006715586.3	XM_021851443.1
XM_006715612.3	XM_001652605.2
XM_006715642.2	XR_002499121.1
XM_006715645.3	XM_021854117.1
XM_006715655.2	XM_021843812.1
XM_006715659.1	XM_021843299.1
XM_006715667.3	XM_021852441.1
XM_006715839.3	XM_021843100.1
XM_006716093.3	XM_021856520.1
XM_006716126.3	XM_021854549.1
XM_006716256.4	XM_001653441.2
XM_006716333.3	XM_021848247.1
XM_006716585.1	XM_001652227.2
XM_006716698.3	XM_021843152.1
XM_006716706.2	XM_021847533.1
XM_006716936.2	XM_021842447.1
XM_006716944.4	XM_001651790.2
XM_006717014.2	XM_021851881.1
XM_006717096.4	XM_021842926.1
XM_006717110.3	XM_001657205.2
XM_006717141.3	XM_021847930.1
XM_006717170.4	XM_021845298.1
XM_006717191.1	XM_021845299.1
XM_006717253.1	XM_001657538.2
XM_006717273.4	XM_021840908.1
XM_006717399.3	XM_021849049.1
XM_006717519.4	XM_021843806.1
XM_006717631.4	XM_021851191.1
XM_006717634.3	XM_001663097.2
XM_006717720.4	XM_021839684.1
XM_006717851.3	XM_021850188.1
XM_006717884.4	XM_021844595.1
XM_006717891.4	XR_002499918.1
XM_006717974.3	XM_001654934.2
XM_006718047.2	XM_021840787.1
XM_006718141.4	XM_021843807.1
XM_006718191.3	XM_021851237.1
XM_006718346.2	XM_021853488.1

XM_006718374.2	XM_001650344.2
XM_006718384.2	XM_021843816.1
XM_006718488.4	XR_002501361.1
XM_006718517.2	XM_021843620.1
XM_006718556.4	XM_021841188.1
XM_006718699.3	XM_001652715.2
XM_006718707.3	XM_021843368.1
XM_006718739.2	XM_021854289.1
XM_006718741.2	XM_021849981.1
XM_006718797.3	XM_021842133.1
XM_006718823.2	XM_021843274.1
XM_006718849.4	XM_001648021.2
XM_006718945.3	XM_021848131.1
XM_006719025.4	XM_021846234.1
XM_006719093.1	XM_021838136.1
XM_006719114.3	XM_021855944.1
XM_006719163.4	XM_021844224.1
XM_006719181.2	XM_001659187.2
XM_006719186.4	XM_001664054.2
XM_006719218.1	XR_002501220.1
XM_006719359.1	XM_021847237.1
XM_006719400.4	XM_021837718.1
XM_006719408.4	XM_021853704.1
XM_006719460.4	XM_021848451.1
XM_006719478.2	XM_021840214.1
XM_006719564.2	XM_021851497.1
XM_006719593.3	XM_021856629.1
XM_006719644.2	XM_021841192.1
XM_006719706.2	XM_021854861.1
XM_006719707.4	XM_021855162.1
XM_006719713.4	XR_002499686.1
XM_006719735.1	XM_001662362.2
XM_006719784.3	XR_002502144.1
XM_006719786.3	XM_021851662.1
XM_006719846.3	XM_021852149.1
XM_006719882.1	XM_021850318.1
XM_006719894.3	XM_021837432.1
XM_006719900.2	XM_021845272.1
XM_006719986.2	XM_001649240.2
XM_006719991.4	XM_021850991.1
XM_006720105.3	XM_021843479.1
XM_006720116.4	XM_021845673.1
XM_006720178.1	XM_021842994.1
XM_006720185.3	XM_021844024.1
XM_006720196.2	XM_021843298.1
XM_006720204.1	XM_021842348.1
XM_006720206.4	XM_021843826.1
XM_006720226.3	XM_001649349.2
XM_006720301.3	XM_021856601.1
XM_006720309.1	XM_021839255.1

XM_006720355.3	XM_021852151.1
XM_006720420.1	XM_021840141.1
XM_006720459.2	XR_002499762.1
XM_006720539.3	XM_001659996.2
XM_006720626.3	XM_021851949.1
XM_006720751.3	XM_021845623.1
XM_006720832.2	XM_021837629.1
XM_006720867.3	XM_021844556.1
XM_006720908.4	XM_021840166.1
XM_006720954.4	XM_021838032.1
XM_006720982.3	XM_021849074.1
XM_006721067.4	XM_021853934.1
XM_006721074.2	XM_021847756.1
XM_006721084.1	XR_002501680.1
XM_006721236.4	XM_021857460.1
XM_006721295.3	XM_021846441.1
XM_006721471.3	XM_021840207.1
XM_006721515.2	XM_001649687.2
XM_006721546.3	XM_001661519.2
XM_006721573.3	XM_021856727.1
XM_006721601.4	XM_021846245.1
XM_006721671.4	XM_021846785.1
XM_006721707.3	XM_021854983.1
XM_006721750.4	XM_021840946.1
XM_006721866.3	XM_021848340.1
XM_006721885.3	XM_001662728.2
XM_006721893.3	XM_021849808.1
XM_006721896.3	XM_021838065.1
XM_006721907.3	XR_002498684.1
XM_006721916.3	XM_021850763.1
XM_006721920.2	XM_021854569.1
XM_006721930.3	XM_021857421.1
XM_006722018.3	XR_002498774.1
XM_006722026.2	XM_021855933.1
XM_006722115.3	XM_021838525.1
XM_006722291.4	XM_021847301.1
XM_006722425.3	XM_021843872.1
XM_006722435.3	XM_021842502.1
XM_006722521.2	XM_021846479.1
XM_006722547.3	XM_001654482.2
XM_006722557.2	XM_001652204.2
XM_006722597.4	XM_021848210.1
XM_006722616.1	XM_021838647.1
XM_006722647.3	XM_021847292.1
XM_006722706.3	XM_001657653.2
XM_006722741.3	XM_021843447.1
XM_006722782.4	XM_021841665.1
XM_006722855.4	XM_021839983.1
XM_006722866.2	XM_001656051.2
XM_006722899.4	XM_021844025.1

XM_006722999.2	XM_001654508.2
XM_006723009.3	XM_021843875.1
XM_006723092.4	XM_021840554.1
XM_006723097.4	XM_001662429.2
XM_006723102.4	XR_002498791.1
XM_006723219.3	XM_021842452.1
XM_006723382.3	XM_021840591.1
XM_006723489.1	XM_021851939.1
XM_006723565.3	XM_001657662.2
XM_006723598.1	XM_021848149.1
XM_006723603.2	XM_001648353.2
XM_006723646.3	XM_021851111.1
XM_006723655.2	XM_021849408.1
XM_006723727.3	XM_021840556.1
XM_006723806.3	XM_021839449.1
XM_006723961.4	XM_021842340.1
XM_006723989.2	XM_001654021.2
XM_006724030.3	XM_021855518.1
XM_006724038.3	XM_021838315.1
XM_006724051.3	XM_001652900.2
XM_006724151.2	XM_001655239.2
XM_006724180.2	XR_002499550.1
XM_006724289.4	XM_021848954.1
XM_006724559.1	XM_021848604.1
XM_006724592.4	XM_001648264.2
XM_006724619.1	XM_011495371.2
XM_006724691.2	XM_021853374.1
XM_006724763.1	XR_002501823.1
XM_006724774.3	XM_021857411.1
XM_006724779.2	XM_021857518.1
XM_006724783.2	XM_021849625.1
XM_006724802.4	XM_021853033.1
XM_006724833.2	XM_021842451.1
XM_006724855.3	XM_021857134.1
XM_011509079.3	XM_021851799.1
XM_011509133.2	XM_021855575.1
XM_011509183.2	XM_001649285.2
XM_011509201.2	XM_021856955.1
XM_011509247.1	XM_021844005.1
XM_011509265.3	XM_021839521.1
XM_011509269.2	XM_021854379.1
XM_011509301.3	XM_021847865.1
XM_011509339.3	XM_021843845.1
XM_011509386.2	XM_021856591.1
XM_011509417.2	XM_021855670.1
XM_011509453.2	XM_021850214.1
XM_011509459.2	XR_002499956.1
XM_011509475.2	XR_002500393.1
XM_011509549.1	XM_021854946.1
XM_011509644.3	XR_002502434.1

XM_011509667.2	XM_021842045.1
XM_011509668.2	XM_021844579.1
XM_011509681.1	XM_021845927.1
XM_011509707.2	XM_021837623.1
XM_011509739.2	XM_021848844.1
XM_011509748.2	XM_021846052.1
XM_011509759.3	XM_001661901.2
XM_011509762.3	XM_021846412.1
XM_011509786.1	XM_021849011.1
XM_011509804.1	XM_021839333.1
XM_011509813.2	XM_021855936.1
XM_011509819.1	XR_002500593.1
XM_011509820.2	XR_002501546.1
XM_011509844.2	XM_021837625.1
XM_011509872.2	XM_001652556.2
XM_011509881.2	XR_002500369.1
XM_011509935.1	XM_021839820.1
XM_011510012.1	XM_001652880.3
XM_011510152.2	XM_021850217.1
XM_011510162.2	XM_021856025.1
XM_011510181.2	XM_021839317.1
XM_011510229.3	XM_001651243.2
XM_011510232.2	XR_002501415.1
XM_011510235.2	XM_001654206.2
XM_011510236.1	XR_002499444.1
XM_011510348.1	XM_021845820.1
XM_011510399.2	XM_001657638.2
XM_011510461.3	XM_021849101.1
XM_011510497.2	XM_001658994.2
XM_011510520.1	XM_021850410.1
XM_011510527.3	XM_001664225.2
XM_011510556.2	XM_021855205.1
XM_011510562.2	XM_021853660.1
XM_011510589.1	XM_021847585.1
XM_011510597.3	XM_001648106.2
XM_011510650.3	XR_002502893.1
XM_011510655.3	XM_001659974.2
XM_011510689.3	XM_021856322.1
XM_011510691.2	XM_021842024.1
XM_011510694.2	XM_001659192.2
XM_011510781.3	XM_021857365.1
XM_011510789.2	XM_021839063.1
XM_011510919.1	XM_021857061.1
XM_011510938.1	XM_021849464.1
XM_011511090.3	XM_021837359.1
XM_011511196.3	XM_021839130.1
XM_011511249.3	XM_021849102.1
XM_011511285.2	XM_021840427.1
XM_011511325.3	XM_021846523.1
XM_011511358.2	XR_002501481.1

XM_011511369.3	XM_021839808.1
XM_011511379.2	XM_021851162.1
XM_011511434.3	XM_001652467.2
XM_011511445.2	XM_021857239.1
XM_011511489.2	XM_021857517.1
XM_011511494.3	XM_021843260.1
XM_011511535.3	XM_021843396.1
XM_011511738.3	XM_021839842.1
XM_011511827.2	XM_021845891.1
XM_011511933.1	XM_001654684.2
XM_011511937.1	XM_021850863.1
XM_011511958.3	XM_021846210.1
XM_011511982.1	XR_002498858.1
XM_011511993.2	XM_021843732.1
XM_011511996.2	XM_021854290.1
XM_011512005.2	XM_021856729.1
XM_011512026.3	XM_001658900.2
XM_011512148.2	XM_021855953.1
XM_011512213.2	XM_021854805.1
XM_011512226.2	XM_021849664.1
XM_011512274.1	XM_001662595.2
XM_011512277.2	XM_021837974.1
XM_011512315.1	XM_021849426.1
XM_011512323.2	XR_002499242.1
XM_011512331.2	XM_021846908.1
XM_011512424.2	XM_021843810.1
XM_011512432.2	XM_021848817.1
XM_011512488.2	XM_001655499.2
XM_011512561.2	XM_021853990.1
XM_011512615.3	XM_021843562.1
XM_011512627.3	XM_021857271.1
XM_011512660.2	XR_002501444.1
XM_011512671.2	XM_021856666.1
XM_011512711.2	XM_021839815.1
XM_011512795.3	XR_002500301.1
XM_011512807.2	XM_021856222.1
XM_011512823.2	XM_021845537.1
XM_011512842.2	XM_001660084.2
XM_011512857.2	XR_002499068.1
XM_011512891.2	XM_001656600.2
XM_011512912.2	XM_021846469.1
XM_011512958.3	XM_001648426.2
XM_011513042.3	XM_001663436.2
XM_011513050.2	XM_001661316.2
XM_011513353.3	XM_021845978.1
XM_011513356.3	XM_001658017.2
XM_011513427.2	XM_021839644.1
XM_011513454.2	XM_021846784.1
XM_011513590.2	XM_021838321.1
XM_011513682.3	XM_021853056.1

XM_011513719.2	XM_021839796.1
XM_011513753.2	XM_001651589.2
XM_011513811.2	XM_021853952.1
XM_011513821.3	XM_021843814.1
XM_011513835.2	XM_021844534.1
XM_011513840.3	XM_021843533.1
XM_011513856.3	XM_001649994.2
XM_011513865.2	XM_001652354.2
XM_011513908.2	XM_001651256.2
XM_011513909.2	XM_001660053.3
XM_011513975.2	XR_002501399.1
XM_011513979.2	XM_021854404.1
XM_011514025.2	XM_001660734.2
XM_011514067.1	XM_021851532.1
XM_011514094.2	XM_021857189.1
XM_011514152.2	XM_021838681.1
XM_011514225.1	XM_001661543.2
XM_011514248.3	XM_011495417.2
XM_011514264.1	XM_021853765.1
XM_011514335.2	XM_021845773.1
XM_011514346.3	XM_001658227.2
XM_011514395.2	XM_001648969.2
XM_011514420.2	XM_021856417.1
XM_011514425.1	XM_001651346.2
XM_011514428.1	XM_021847042.1
XM_011514437.3	XM_021855906.1
XM_011514459.2	XM_021855615.1
XM_011514506.2	XM_021843760.1
XM_011514615.2	XM_021841026.1
XM_011514624.2	XM_021854878.1
XM_011514663.1	XM_021857600.1
XM_011514665.1	XM_021841247.1
XM_011514726.2	XM_021852948.1
XM_011514767.2	XM_001657693.2
XM_011514770.1	XM_021839141.1
XM_011514780.1	XM_021843563.1
XM_011514812.2	XM_001651827.2
XM_011514854.2	XM_021842647.1
XM_011514859.1	XM_021842753.1
XM_011514873.2	XM_021855393.1
XM_011514917.2	XM_001663465.2
XM_011514937.2	XM_021854502.1
XM_011514938.2	XM_001649646.2
XM_011514953.3	XM_021842788.1
XM_011515081.2	XM_001650392.2
XM_011515087.1	XM_001648941.2
XM_011515151.3	XM_001654834.2
XM_011515154.2	XM_021852968.1
XM_011515213.2	XM_021850319.1
XM_011515223.2	XM_021847492.1

XM_011515231.2	XM_021842448.1
XM_011515241.1	XM_021853355.1
XM_011515402.3	XM_021845408.1
XM_011515407.2	XM_021843606.1
XM_011515443.2	XM_021842083.1
XM_011515444.2	XM_021847975.1
XM_011515458.2	XM_021851190.1
XM_011515527.3	XM_021850961.1
XM_011515555.1	XM_001649754.2
XM_011515556.1	XM_021850306.1
XM_011515572.1	XM_001651480.2
XM_011515582.3	XM_001650614.2
XM_011515583.2	XM_021850875.1
XM_011515654.2	XM_021845565.1
XM_011515778.1	XM_021845850.1
XM_011515795.2	XM_001654278.2
XM_011515821.2	XM_001660085.2
XM_011515826.1	XM_001656929.2
XM_011515921.1	XM_001657318.2
XM_011515964.2	XM_021845604.1
XM_011515969.1	XM_021841583.1
XM_011515990.2	XM_021839871.1
XM_011515994.1	XM_021845555.1
XM_011516031.1	XM_001657792.2
XM_011516053.2	XR_002498701.1
XM_011516066.3	XM_021847549.1
XM_011516076.2	XM_021856314.1
XM_011516102.2	XM_021843819.1
XM_011516159.3	XM_021846254.1
XM_011516190.2	XM_021837687.1
XM_011516259.2	XR_002498837.1
XM_011516283.1	XM_021850307.1
XM_011516290.2	XM_001655220.2
XM_011516609.2	XR_002498999.1
XM_011516640.2	XM_001661169.2
XM_011516669.3	XM_021845536.1
XM_011516672.2	XM_021849390.1
XM_011516737.1	XM_021838824.1
XM_011516798.1	XM_001661387.2
XM_011516926.2	XM_001662181.2
XM_011516939.3	XM_001653333.2
XM_011516944.2	XM_021840330.1
XM_011517022.2	XM_021853955.1
XM_011517048.2	XM_021843867.1
XM_011517136.2	XM_021845925.1
XM_011517202.2	XM_021838537.1
XM_011517214.1	XM_021847580.1
XM_011517219.1	XM_021842485.1
XM_011517239.2	XM_021853800.1
XM_011517257.2	XM_021851165.1

XM_011517259.2	XM_001660971.2
XM_011517269.1	XM_021848841.1
XM_011517274.2	XM_021855883.1
XM_011517312.2	XM_021856746.1
XM_011517361.2	XR_002500238.1
XM_011517363.3	XM_021845601.1
XM_011517495.2	XR_002501578.1
XM_011517509.3	XM_001656595.2
XM_011517539.3	XM_001662561.2
XM_011517570.2	XM_001662238.2
XM_011517588.3	XM_021851643.1
XM_011517592.3	XM_021841844.1
XM_011517616.2	XM_001648539.2
XM_011517625.2	XM_021846704.1
XM_011517635.1	XM_001656358.2
XM_011517676.2	XR_002502126.1
XM_011517736.3	XM_021839553.1
XM_011517744.1	XM_021838667.1
XM_011517861.2	XM_021846786.1
XM_011517863.3	XM_021840351.1
XM_011517963.1	XM_011495400.2
XM_011518067.1	XM_001660843.2
XM_011518070.2	XM_021850121.1
XM_011518088.2	XM_021847319.1
XM_011518153.1	XM_021852591.1
XM_011518168.2	XM_001663903.2
XM_011518189.3	XM_021844553.1
XM_011518209.3	XM_021848815.1
XM_011518233.2	XM_001647859.2
XM_011518257.2	XM_021847899.1
XM_011518277.2	XM_021849406.1
XM_011518294.3	XM_001655888.2
XM_011518341.3	XM_001654831.2
XM_011518345.3	XM_021851034.1
XM_011518352.1	XM_021838788.1
XM_011518353.1	XM_021853348.1
XM_011518361.2	XM_021853045.1
XM_011518366.3	XM_021852785.1
XM_011518374.2	XM_021851784.1
XM_011518406.2	XR_002500065.1
XM_011518488.2	XM_021841488.1
XM_011518573.3	XM_021846460.1
XM_011518584.1	XM_001663395.2
XM_011518600.1	XM_021854788.1
XM_011518639.1	XM_021854164.1
XM_011518650.3	XM_021848068.1
XM_011518770.2	XM_021842786.1
XM_011518838.2	XM_021843448.1
XM_011518875.2	XM_021854405.1
XM_011518903.3	XR_002501607.1

XM_011518907.2	XM_021856352.1
XM_011518909.2	XM_011495081.2
XM_011518925.1	XM_001655284.2
XM_011518950.2	XM_021856795.1
XM_011519016.1	XM_001663420.2
XM_011519089.3	XM_021852868.1
XM_011519091.3	XM_021847884.1
XM_011519114.2	XM_021852316.1
XM_011519134.3	XM_001659450.2
XM_011519152.3	XM_021850590.1
XM_011519208.2	XR_002499778.1
XM_011519214.2	XM_021837263.1
XM_011519224.1	XM_021856220.1
XM_011519230.2	XM_001659538.2
XM_011519241.2	XR_002502405.1
XM_011519264.2	XM_021847789.1
XM_011519297.1	XM_021850302.1
XM_011519306.2	XM_021841209.1
XM_011519330.2	XM_021840399.1
XM_011519337.2	XM_001656861.2
XM_011519372.2	XM_021837367.1
XM_011519398.2	XM_021850614.1
XM_011519405.2	XM_001663245.2
XM_011519503.1	XM_021848701.1
XM_011519519.3	XM_021845347.1
XM_011519538.2	XR_002503000.1
XM_011519564.2	XR_002501732.1
XM_011519604.2	XM_021847581.1
XM_011519628.1	XM_021852835.1
XM_011519672.1	XM_021849080.1
XM_011519674.1	XM_021841816.1
XM_011519710.2	XM_021849418.1
XM_011519720.2	XM_021851096.1
XM_011519743.1	XM_001660863.2
XM_011519830.3	XR_002501412.1
XM_011519863.1	XM_021854696.1
XM_011519873.3	XM_021846472.1
XM_011519890.1	XM_001659383.3
XM_011519898.3	XM_021848117.1
XM_011519907.2	XM_021850284.1
XM_011519921.2	XM_021840282.1
XM_011519941.2	XM_021848938.1
XM_011520048.1	XM_021848875.1
XM_011520209.3	XM_021843345.1
XM_011520295.2	XM_021856049.1
XM_011520302.3	XM_001656876.2
XM_011520338.1	XM_021839232.1
XM_011520373.3	XM_001656354.2
XM_011520379.2	XM_001654500.3
XM_011520406.2	XM_021839074.1

XM_011520476.2	XM_001657220.2
XM_011520485.2	XM_021854378.1
XM_011520493.2	XM_021853298.1
XM_011520524.1	XM_021856095.1
XM_011520560.2	XR_002501063.1
XM_011520623.3	XM_021840880.1
XM_011520747.2	XM_021837998.1
XM_011520751.3	XM_021848816.1
XM_011520760.2	XR_002499536.1
XM_011520802.2	XM_021857722.1
XM_011520815.3	XM_021841150.1
XM_011520934.3	XM_001656090.3
XM_011520997.3	XM_021841804.1
XM_011521100.2	XM_021839290.1
XM_011521166.2	XM_011494692.2
XM_011521186.2	XM_001649186.2
XM_011521243.3	XM_021847152.1
XM_011521249.2	XM_021855909.1
XM_011521262.2	XM_001647840.2
XM_011521271.2	XM_021847328.1
XM_011521320.1	XM_021849013.1
XM_011521345.3	XM_001656139.2
XM_011521351.2	XM_021846449.1
XM_011521363.2	XM_021855396.1
XM_011521366.2	XM_001653872.2
XM_011521381.2	XM_021846780.1
XM_011521396.2	XM_001658537.2
XM_011521403.2	XM_021847424.1
XM_011521497.2	XM_001651344.3
XM_011521505.2	XM_001650219.2
XM_011521556.2	XM_021838040.1
XM_011521587.2	XM_001652154.2
XM_011521591.2	XM_001648885.2
XM_011521611.3	XM_001661830.2
XM_011521620.3	XM_021845794.1
XM_011521656.3	XM_021846946.1
XM_011521660.3	XM_021855790.1
XM_011521664.2	XM_001658944.2
XM_011521665.2	XM_021846950.1
XM_011521668.2	XM_021846429.1
XM_011521670.2	XM_021850507.1
XM_011521690.2	XM_021855816.1
XM_011521780.3	XM_001661359.2
XM_011521805.2	XM_001661816.2
XM_011521852.1	XM_021845837.1
XM_011521884.1	XM_021841145.1
XM_011521893.1	XM_021841965.1
XM_011521951.3	XM_021842488.1
XM_011521976.3	XM_021843627.1
XM_011522009.2	XM_001662643.2

XM_011522020.1	XM_021839407.1
XM_011522023.1	XM_021840032.1
XM_011522028.1	XM_001664198.2
XM_011522034.2	XM_001651358.2
XM_011522037.2	XM_021848874.1
XM_011522044.2	XM_021840329.1
XM_011522071.1	XM_021852141.1
XM_011522076.2	XM_021857287.1
XM_011522088.1	XM_021845988.1
XM_011522125.3	XM_021846951.1
XM_011522192.2	XM_021847873.1
XM_011522204.3	XR_002502557.1
XM_011522232.2	XM_001650616.2
XM_011522234.3	XM_021848244.1
XM_011522244.1	XR_002500357.1
XM_011522351.2	XM_021846859.1
XM_011522402.1	XM_021854417.1
XM_011522516.3	XM_021838629.1
XM_011522580.2	XM_011494999.2
XM_011522608.2	XR_002503088.1
XM_011522727.3	XM_021847441.1
XM_011522783.2	XM_021850419.1
XM_011522823.2	XM_001649512.2
XM_011522836.2	XM_021837976.1
XM_011522914.2	XM_001661854.2
XM_011522921.2	XM_021843216.1
XM_011523002.2	XM_001658139.2
XM_011523023.2	XM_021842132.1
XM_011523041.2	XM_001648293.2
XM_011523095.2	XM_021847543.1
XM_011523176.2	XM_021840720.1
XM_011523178.2	XM_021837738.1
XM_011523214.2	XM_001662600.2
XM_011523222.2	XM_021857475.1
XM_011523229.1	XM_021854443.1
XM_011523230.2	XM_021846240.1
XM_011523232.2	XM_021849499.1
XM_011523243.1	XM_021838706.1
XM_011523289.1	XM_021846943.1
XM_011523294.2	XR_002498744.1
XM_011523321.1	XM_021843597.1
XM_011523326.1	XM_001658376.2
XM_011523352.1	XM_021842920.1
XM_011523353.1	XM_021854526.1
XM_011523541.2	XM_021840930.1
XM_011523597.2	XM_021846484.1
XM_011523691.2	XM_021851514.1
XM_011523713.2	XM_021845897.1
XM_011523724.1	XM_001648859.2
XM_011523738.2	XM_021838879.1

XM_011523752.3	XM_021857368.1
XM_011523774.2	XM_021849729.1
XM_011523851.2	XM_021852966.1
XM_011523902.3	XR_002501617.1
XM_011523910.2	XM_001652999.2
XM_011523945.2	XM_021846534.1
XM_011523963.1	XM_021849578.1
XM_011523966.1	XM_021844294.1
XM_011523992.3	XM_021853238.1
XM_011524045.2	XM_021848939.1
XM_011524057.2	XM_021849784.1
XM_011524077.3	XM_021851261.1
XM_011524080.2	XM_001651707.2
XM_011524102.3	XM_021844958.1
XM_011524104.2	XM_021853688.1
XM_011524109.3	XM_021857614.1
XM_011524114.3	XM_021846410.1
XM_011524173.1	XM_021856076.1
XM_011524194.1	XM_021854154.1
XM_011524234.1	XM_001661964.2
XM_011524258.1	XM_001662776.2
XM_011524291.1	XM_001649616.2
XM_011524296.2	XM_021846161.1
XM_011524339.2	XM_021841509.1
XM_011524390.2	XM_021846783.1
XM_011524448.2	XM_021839190.1
XM_011524453.1	XM_021853801.1
XM_011524467.2	XM_021844954.1
XM_011524493.1	XM_021842206.1
XM_011524496.2	XM_021852862.1
XM_011524521.2	XR_002499043.1
XM_011524555.2	XM_021837979.1
XM_011524667.2	XM_021842492.1
XM_011524688.3	XM_021844820.1
XM_011524701.1	XM_021841582.1
XM_011524846.3	XM_001660356.2
XM_011524896.2	XM_001652774.2
XM_011524929.2	XM_021843854.1
XM_011524950.2	XM_021853450.1
XM_011524957.2	XM_021850144.1
XM_011525065.2	XM_021845836.1
XM_011525082.3	XM_001663485.2
XM_011525102.3	XR_002502335.1
XM_011525118.2	XM_021842003.1
XM_011525139.2	XM_021847583.1
XM_011525162.2	XM_021844119.1
XM_011525183.2	XM_021848571.1
XM_011525211.3	XM_021853180.1
XM_011525216.1	XM_021844039.1
XM_011525242.2	XM_021841510.1

XM_011525453.2	XM_021842029.1
XM_011525454.3	XM_021841392.1
XM_011525475.3	XM_021844935.1
XM_011525478.2	XM_001652219.2
XM_011525480.2	XM_001659116.2
XM_011525495.2	XM_021848702.1
XM_011525504.3	XM_001661307.2
XM_011525525.1	XM_021853183.1
XM_011525542.1	XM_021850321.1
XM_011525569.2	XM_021857115.1
XM_011525603.2	XM_021853993.1
XM_011525757.1	XM_021841273.1
XM_011525804.2	XM_021848550.1
XM_011525917.3	XM_021843954.1
XM_011525932.1	XM_021848238.1
XM_011525933.2	XM_021838245.1
XM_011526007.2	XM_021854004.1
XM_011526099.2	XR_002499191.1
XM_011526132.2	XM_021848261.1
XM_011526142.1	XM_021846033.1
XM_011526150.2	XM_001662356.2
XM_011526169.3	XM_021839827.1
XM_011526201.2	XM_021837837.1
XM_011526290.2	XM_021841251.1
XM_011526335.2	XM_021853558.1
XM_011526371.2	XM_021844889.1
XM_011526391.1	XM_001650283.2
XM_011526484.2	XM_001649955.2
XM_011526517.2	XM_021842878.1
XM_011526555.3	XM_021843989.1
XM_011526603.2	XM_001658954.2
XM_011526611.2	XM_021842386.1
XM_011526664.2	XM_021838474.1
XM_011526842.1	XM_021838936.1
XM_011526846.1	XM_021846778.1
XM_011526861.3	XM_021841266.1
XM_011526867.1	XM_021853643.1
XM_011526874.2	XM_021840553.1
XM_011526973.2	XM_021854235.1
XM_011526980.3	XM_001662100.2
XM_011526990.2	XM_021848515.1
XM_011527048.3	XM_001656034.2
XM_011527071.1	XM_021855006.1
XM_011527085.2	XM_021838201.1
XM_011527087.2	XM_021838183.1
XM_011527116.1	XM_021848245.1
XM_011527119.1	XM_021853093.1
XM_011527134.1	XM_001660472.2
XM_011527156.2	XR_002500131.1
XM_011527162.2	XM_001660001.2

XM_011527170.1	XM_001654790.2
XM_011527215.1	XR_002498976.1
XM_011527228.3	XM_021851909.1
XM_011527297.2	XM_001659872.2
XM_011527341.2	XM_021837893.1
XM_011527368.2	XM_021846594.1
XM_011527370.2	XM_001653246.2
XM_011527397.2	XM_021846475.1
XM_011527424.3	XM_021843275.1
XM_011527428.1	XM_021839098.1
XM_011527432.3	XM_001657561.2
XM_011527436.1	XM_021844665.1
XM_011527560.2	XM_001654457.2
XM_011527682.2	XM_021849065.1
XM_011527710.3	XM_001660293.2
XM_011527831.2	XM_021840226.1
XM_011527861.1	XM_001650735.2
XM_011527885.3	XM_021850317.1
XM_011527897.3	XM_021838916.1
XM_011527903.3	XM_001654933.2
XM_011527909.3	XM_021845319.1
XM_011527917.1	XR_002500735.1
XM_011527934.1	XM_021847579.1
XM_011527967.2	XM_001650583.2
XM_011527974.2	XM_001652268.2
XM_011527982.3	XM_011495114.2
XM_011528042.2	XM_021840496.1
XM_011528069.2	XM_021848923.1
XM_011528090.2	XM_021848749.1
XM_011528094.1	XM_021854632.1
XM_011528136.1	XM_021837236.1
XM_011528167.2	XM_001650125.2
XM_011528177.2	XM_001655126.2
XM_011528272.2	XR_002499770.1
XM_011528278.2	XM_021850650.1
XM_011528279.2	XM_021846965.1
XM_011528281.2	XM_021853973.1
XM_011528293.2	XM_021853086.1
XM_011528442.2	XM_001658471.2
XM_011528449.3	XM_021841244.1
XM_011528481.3	XM_021839527.1
XM_011528526.2	XM_001647723.2
XM_011528527.2	XM_021848898.1
XM_011528529.3	XM_021839243.1
XM_011528545.1	XM_001654514.2
XM_011528566.2	XR_002499475.1
XM_011528586.2	XM_021853893.1
XM_011528747.2	XM_021840288.1
XM_011528748.2	XM_021853815.1
XM_011528768.3	XM_001660114.2

XM_011528994.2	XM_021857536.1
XM_011528997.3	XM_021838187.1
XM_011529049.1	XM_021841231.1
XM_011529085.2	XM_021841156.1
XM_011529100.2	XM_021842941.1
XM_011529140.2	XM_001648401.2
XM_011529182.2	XM_001658414.2
XM_011529263.2	XM_001650180.2
XM_011529277.2	XM_021845305.1
XM_011529342.2	XM_021850280.1
XM_011529358.3	XM_021849574.1
XM_011529467.1	XM_021844666.1
XM_011529471.1	XM_021848087.1
XM_011529507.3	XM_001659135.2
XM_011529510.2	XM_001663443.2
XM_011529514.3	XM_021855334.1
XM_011529544.2	XM_021847241.1
XM_011529609.2	XM_001652735.2
XM_011529637.2	XM_021840717.1
XM_011529644.1	XM_021841835.1
XM_011529662.2	XM_001657634.2
XM_011529766.2	XM_021842694.1
XM_011529768.2	XR_002502196.1
XM_011529841.1	XM_021838221.1
XM_011529900.2	XM_021841806.1
XM_011529908.2	XM_021855294.1
XM_011529914.2	XM_021849082.1
XM_011529921.3	XR_002500873.1
XM_011529938.2	XM_021857145.1
XM_011529954.2	XM_021838653.1
XM_011529955.1	XM_021851808.1
XM_011529965.2	XM_021837516.1
XM_011530012.1	XM_001658338.2
XM_011530038.2	XM_021854634.1
XM_011530050.2	XM_021841508.1
XM_011530106.1	XM_001650488.2
XM_011530200.2	XM_021848171.1
XM_011530204.1	XM_001650950.2
XM_011530208.2	XM_021843591.1
XM_011530260.3	XM_021851588.1
XM_011530290.2	XM_021842977.1
XM_011530311.1	XM_021837655.1
XM_011530377.2	XM_021855071.1
XM_011530432.2	XR_002502662.1
XM_011530435.2	XM_001657378.2
XM_011530472.2	XM_021840711.1
XM_011530557.2	XM_021849814.1
XM_011530696.1	XM_021845456.1
XM_011530697.1	XM_021857324.1
XM_011530716.2	XM_021854787.1

XM_011530717.2	XM_001658546.2
XM_011530735.2	XM_001652505.2
XM_011530813.1	XM_021837902.1
XM_011530848.3	XM_021837731.1
XM_011530884.1	XM_021852275.1
XM_011530912.2	XR_002499697.1
XM_011530950.2	XM_021841254.1
XM_011530957.3	XM_021857309.1
XM_011530977.1	XM_021850338.1
XM_011530994.1	XM_021839922.1
XM_011531032.1	XR_002499213.1
XM_011531140.2	XM_021848390.1
XM_011531171.1	XM_021857288.1
XM_011531311.2	XM_001649521.2
XM_011531317.3	XM_001658275.2
XM_011531407.3	XM_021845271.1
XM_011531412.3	XM_021845963.1
XM_011531486.2	XM_001654422.2
XM_011531509.3	XR_002501834.1
XM_011531532.2	XM_021843170.1
XM_011531533.2	XM_021848752.1
XM_011531563.2	XM_021855192.1
XM_011531587.3	XM_021853582.1
XM_011531663.3	XM_021851723.1
XM_011531671.2	XM_021847239.1
XM_011531679.3	XM_021857215.1
XM_011531693.2	XM_001652577.2
XM_011531695.3	XM_021839100.1
XM_011531716.3	XM_021852287.1
XM_011531725.1	XR_002501160.1
XM_011531775.1	XM_021839019.1
XM_011531868.3	XM_021856416.1
XM_011531895.2	XM_021847929.1
XM_011531903.2	XM_001655931.2
XM_011531912.2	XM_001658501.2
XM_011532004.1	XM_021838052.1
XM_011532022.2	XM_021843637.1
XM_011532075.1	XM_021841239.1
XM_011532088.2	XR_002499548.1
XM_011532102.1	XM_021850736.1
XM_011532118.3	XM_021857686.1
XM_011532128.2	XM_021846894.1
XM_011532130.1	XM_001663692.2
XM_011532162.2	XM_021854233.1
XM_011532233.3	XM_021846833.1
XM_011532267.2	XM_021850273.1
XM_011532358.2	XR_002503161.1
XM_011532383.2	XR_002503006.1
XM_011532386.2	XM_021853100.1
XM_011532399.2	XR_002499472.1

XM_011532400.2	XM_021857701.1
XM_011532505.2	XM_021844577.1
XM_011532551.2	XM_021846589.1
XM_011532566.2	XM_021854667.1
XM_011532571.2	XM_021852723.1
XM_011532623.2	XM_021855079.1
XM_011532642.2	XM_021843155.1
XM_011532716.3	XM_001658787.2
XM_011532721.2	XM_021846838.1
XM_011532730.3	XM_021844662.1
XM_011532736.2	XR_002500683.1
XM_011532766.1	XM_021843276.1
XM_011532819.2	XM_021845123.1
XM_011532919.3	XM_021857196.1
XM_011532963.3	XM_021846434.1
XM_011533045.2	XM_001650222.2
XM_011533150.3	XM_001654370.2
XM_011533154.2	XM_021848863.1
XM_011533166.3	XR_002501149.1
XM_011533268.1	XM_021842921.1
XM_011533286.2	XM_021851030.1
XM_011533371.1	XM_021845600.1
XM_011533394.3	XM_021850722.1
XM_011533433.2	XR_002499456.1
XM_011533445.2	XR_002500793.1
XM_011533478.2	XM_001664240.2
XM_011533547.3	XM_021851167.1
XM_011533576.2	XM_021856105.1
XM_011533615.2	XM_021848737.1
XM_011533625.3	XM_001657053.3
XM_011533747.3	XM_021838913.1
XM_011533774.1	XM_021855057.1
XM_011533782.2	XM_021855236.1
XM_011533834.1	XM_021839467.1
XM_011533928.1	XR_002502529.1
XM_011533980.1	XM_021854162.1
XM_011533990.3	XM_021847449.1
XM_011533991.2	XM_001661094.2
XM_011534070.1	XM_021848600.1
XM_011534079.1	XM_021856287.1
XM_011534097.2	XM_021851791.1
XM_011534099.2	XM_001649179.2
XM_011534113.2	XM_021852830.1
XM_011534152.2	XM_021845413.1
XM_011534192.2	XM_001651697.2
XM_011534200.3	XM_021843949.1
XM_011534307.2	XM_021857006.1
XM_011534382.2	XM_021851374.1
XM_011534478.3	XM_021839049.1
XM_011534485.1	XM_021845491.1

XM_011534487.1	XM_021848264.1
XM_011534568.2	XM_021850428.1
XM_011534637.2	XM_021840721.1
XM_011534699.2	XM_011494733.2
XM_011534780.2	XM_021852079.1
XM_011534793.2	XM_021854773.1
XM_011534836.3	XM_021853974.1
XM_011534898.2	XM_021846261.1
XM_011534905.1	XM_001658364.2
XM_011534924.3	XM_001661757.2
XM_011534931.1	XM_021839340.1
XM_011534932.2	XM_021848118.1
XM_011534956.2	XM_001648077.2
XM_011535032.3	XM_021848211.1
XM_011535144.2	XM_021856046.1
XM_011535158.2	XM_021852969.1
XM_011535206.1	XR_002499461.1
XM_011535231.2	XR_002499395.1
XM_011535239.3	XM_021840347.1
XM_011535266.2	XM_001652046.2
XM_011535393.3	XM_021852588.1
XM_011535438.2	XM_021847900.1
XM_011535439.2	XM_021849056.1
XM_011535520.1	XM_021856801.1
XM_011535524.2	XM_021852946.1
XM_011535563.2	XR_002502068.1
XM_011535575.3	XM_021839354.1
XM_011535604.3	XM_021847850.1
XM_011535632.2	XM_021842040.1
XM_011535679.3	XM_011495126.2
XM_011535685.3	XM_021853207.1
XM_011535820.2	XM_001651457.2
XM_011535863.1	XM_021842067.1
XM_011535868.2	XM_021846814.1
XM_011535877.3	XM_001654428.2
XM_011535918.3	XM_001653632.3
XM_011535921.2	XM_021854821.1
XM_011535939.2	XM_021846415.1
XM_011535977.2	XM_021854755.1
XM_011536005.3	XR_002500104.1
XM_011536057.3	XM_021841497.1
XM_011536066.1	XM_001658924.2
XM_011536106.2	XM_001654982.2
XM_011536176.2	XM_021847407.1
XM_011536196.3	XM_021852949.1
XM_011536227.2	XM_021843701.1
XM_011536365.2	XM_001650154.2
XM_011536383.2	XM_021855080.1
XM_011536406.2	XM_021846702.1
XM_011536475.2	XM_001653035.2

XM_011536522.3	XM_021847008.1
XM_011536542.3	XM_021842716.1
XM_011536618.2	XM_021840831.1
XM_011536651.3	XM_001656959.2
XM_011536690.3	XM_021850684.1
XM_011536720.3	XM_021837611.1
XM_011536778.2	XM_001657106.2
XM_011536846.2	XM_021851952.1
XM_011536872.1	XM_021846502.1
XM_011536886.2	XM_021841968.1
XM_011536946.3	XM_021844984.1
XM_011536957.1	XM_021849647.1
XM_011536966.2	XM_021839994.1
XM_011537009.1	XM_021839017.1
XM_011537078.2	XM_021838915.1
XM_011537081.2	XM_001656655.3
XM_011537084.2	XM_021851299.1
XM_011537179.2	XM_001654501.3
XM_011537182.2	XM_021842584.1
XM_011537203.3	XM_001650374.2
XM_011537310.1	XR_002499460.1
XM_011537332.2	XM_001659695.2
XM_011537338.1	XM_021849920.1
XM_011537343.1	XM_021851263.1
XM_011537362.2	XM_021854032.1
XM_011537397.1	XM_001651439.2
XM_011537417.2	XM_021841516.1
XM_011537462.2	XM_021847121.1
XM_011537484.1	XM_021853237.1
XM_011537504.2	XM_021847535.1
XM_011537509.2	XM_021852169.1
XM_011537556.2	XM_021842187.1
XM_011537615.1	XM_021846428.1
XM_011537616.1	XM_001662892.2
XM_011537696.2	XM_021852223.1
XM_011537730.3	XM_021838264.1
XM_011537846.2	XM_021841332.1
XM_011537912.2	XM_021852886.1
XM_011537972.3	XM_021844842.1
XM_011537995.2	XM_001660079.2
XM_011538080.2	XM_001658544.2
XM_011538184.2	XM_021852078.1
XM_011538186.3	XM_021857199.1
XM_011538209.2	XM_021844679.1
XM_011538213.2	XM_001650853.2
XM_011538361.3	XM_021854287.1
XM_011538393.2	XR_002503280.1
XM_011538446.3	XM_021853234.1
XM_011538477.2	XM_021841135.1
XM_011538525.3	XM_021846812.1

XM_011538598.2	XR_002498907.1
XM_011538631.2	XM_001660234.2
XM_011538643.3	XM_021850336.1
XM_011538688.2	XM_021847151.1
XM_011538692.2	XM_021855747.1
XM_011538700.2	XM_021841206.1
XM_011538802.1	XM_021837793.1
XM_011538813.2	XM_001660580.2
XM_011538828.3	XM_021857322.1
XM_011538829.3	XM_001660693.3
XM_011538833.2	XM_021856990.1
XM_011538838.2	XM_021841234.1
XM_011538844.2	XM_021847480.1
XM_011538926.1	XM_001651531.2
XM_011538971.2	XM_021856338.1
XM_011538993.3	XM_021838919.1
XM_011539000.2	XM_021845846.1
XM_011539125.2	XM_021845158.1
XM_011539132.2	XM_021841240.1
XM_011539173.3	XR_002501147.1
XM_011539199.3	XM_021847430.1
XM_011539279.1	XM_021854406.1
XM_011539300.2	XM_021840598.1
XM_011539302.2	XM_021839057.1
XM_011539308.2	XM_021842911.1
XM_011539355.2	XM_021839501.1
XM_011539387.2	XM_021848495.1
XM_011539492.3	XM_001654747.2
XM_011539537.2	XM_001655396.2
XM_011539592.3	XR_002502313.1
XM_011539640.1	XM_021856280.1
XM_011539673.2	XM_021843765.1
XM_011539744.3	XM_021851638.1
XM_011539746.3	XM_021847039.1
XM_011539753.2	XM_001662050.2
XM_011539805.1	XM_001649154.2
XM_011539826.3	XM_021854833.1
XM_011539851.3	XM_021853985.1
XM_011539961.2	XM_021847460.1
XM_011539987.2	XM_021840846.1
XM_011540104.2	XM_021848095.1
XM_011540154.2	XM_021842769.1
XM_011540160.2	XM_001654622.2
XM_011540198.2	XM_021851094.1
XM_011540207.2	XM_001662995.2
XM_011540221.3	XM_001652368.2
XM_011540238.2	XM_021851590.1
XM_011540325.3	XM_001656635.2
XM_011540327.2	XM_021838229.1
XM_011540337.1	XM_021853059.1

XM_011540338.1	XR_002500007.1
XM_011540340.3	XM_001653516.2
XM_011540428.1	XM_021855671.1
XM_011540466.3	XM_021844896.1
XM_011540521.3	XM_021849466.1
XM_011540596.2	XM_021847711.1
XM_011540603.2	XM_021853308.1
XM_011540606.2	XM_021856141.1
XM_011540617.3	XM_021840244.1
XM_011540639.3	XM_021845915.1
XM_011540726.3	XM_001660077.2
XM_011540730.1	XM_001649072.2
XM_011540779.3	XM_021855706.1
XM_011540797.2	XR_002501336.1
XM_011540798.1	XM_021843438.1
XM_011540903.2	XM_021841564.1
XM_011540906.3	XR_002498681.1
XM_011540925.2	XM_021853969.1
XM_011540932.2	XM_001648296.2
XM_011540945.2	XM_001661521.2
XM_011540957.1	XM_001650622.2
XM_011540960.1	XM_011495053.2
XM_011540996.2	XM_021856804.1
XM_011541011.2	XM_021841796.1
XM_011541051.2	XM_001649622.2
XM_011541154.2	XM_021852261.1
XM_011541213.1	XR_002499056.1
XM_011541285.1	XM_021843868.1
XM_011541290.2	XM_021855752.1
XM_011541384.2	XM_021852706.1
XM_011541459.2	XR_002501854.1
XM_011541498.1	XM_001654729.2
XM_011541503.2	XR_002503223.1
XM_011541587.3	XM_001658000.2
XM_011541631.3	XM_021846096.1
XM_011541691.2	XM_021840978.1
XM_011541708.3	XM_021843950.1
XM_011541739.2	XM_021848599.1
XM_011541763.1	XM_021842742.1
XM_011541766.2	XM_021855123.1
XM_011541768.1	XM_021842183.1
XM_011541872.3	XM_001647755.2
XM_011541962.2	XR_002500968.1
XM_011541963.2	XM_001657520.2
XM_011541971.2	XM_001663696.2
XM_011541974.2	XM_021839479.1
XM_011542035.3	XM_021848098.1
XM_011542063.2	XM_021846813.1
XM_011542072.1	XM_021838918.1
XM_011542077.2	XM_021846598.1

XM_011542212.2	XM_011494908.2
XM_011542265.3	XM_021841241.1
XM_011542351.1	XR_002500663.1
XM_011542383.2	XM_021837244.1
XM_011542399.2	XM_001652695.2
XM_011542522.2	XM_021854003.1
XM_011542567.2	XM_021846416.1
XM_011542632.3	XM_021846974.1
XM_011542633.2	XM_021857457.1
XM_011542648.2	XM_021837964.1
XM_011542739.2	XM_021846098.1
XM_011542740.2	XM_021853434.1
XM_011542790.3	XM_021840597.1
XM_011542792.1	XM_001648951.2
XM_011542857.2	XM_021849129.1
XM_011542870.2	XM_021840017.1
XM_011542901.2	XM_021840878.1
XM_011542903.3	XR_002501937.1
XM_011542907.1	XM_021840055.1
XM_011542917.2	XM_021837801.1
XM_011543000.2	XM_021854094.1
XM_011543048.1	XM_021849128.1
XM_011543055.2	XM_001657472.2
XM_011543086.2	XM_021840704.1
XM_011543131.3	XM_001656392.2
XM_011543143.2	XM_001653346.2
XM_011543150.1	XM_001658122.2
XM_011543167.3	XM_021844597.1
XM_011543232.2	XM_021847971.1
XM_011543255.3	XM_021850345.1
XM_011543308.3	XM_021854684.1
XM_011543334.2	XM_021854005.1
XM_011543340.2	XM_001648603.2
XM_011543349.3	XM_021846921.1
XM_011543366.2	XM_001654598.2
XM_011543385.3	XM_021842134.1
XM_011543531.3	XM_021842535.1
XM_011543533.2	XM_021837246.1
XM_011543538.2	XM_001654722.2
XM_011543559.2	XR_002500941.1
XM_011543570.2	XM_021842570.1
XM_011543574.3	XM_021840712.1
XM_011543586.2	XM_021852271.1
XM_011543607.2	XM_021837795.1
XM_011543608.2	XM_021848341.1
XM_011543609.2	XM_021839971.1
XM_011543643.2	XM_021855793.1
XM_011543680.2	XM_021856841.1
XM_011543735.3	XM_021851095.1
XM_011543758.2	XM_021842326.1

XM_011543766.1	XM_001662634.2
XM_011543786.2	XM_021840913.1
XM_011543811.2	XM_021853314.1
XM_011543821.2	XM_021838705.1
XM_011543830.3	XM_021848745.1
XM_011543843.3	XM_021841660.1
XM_011543885.2	XR_002498600.1
XM_011544035.2	XM_021855378.1
XM_011544048.2	XR_002502832.1
XM_011544063.2	XM_001658074.2
XM_011544076.1	XR_002501465.1
XM_011544087.2	XM_021837548.1
XM_011544108.3	XM_001658587.2
XM_011544132.2	XM_021847688.1
XM_011544161.3	XR_002500474.1
XM_011544164.2	XM_001649070.2
XM_011544169.2	XM_021850695.1
XM_011544206.3	XM_021839421.1
XM_011544220.2	XM_021839958.1
XM_011544227.1	XM_021842246.1
XM_011544305.2	XM_001650752.3
XM_011544342.3	XM_021848890.1
XM_011544360.2	XM_021851583.1
XM_011544364.3	XM_021852950.1
XM_011544368.3	XM_021847228.1
XM_011544426.2	XM_021848017.1
XM_011544456.2	XM_021838799.1
XM_011544481.2	XM_021853212.1
XM_011544482.2	XM_001662843.2
XM_011544505.2	XM_001656295.2
XM_011544518.2	XM_021856815.1
XM_011544526.2	XM_001653506.2
XM_011544550.2	XM_021854234.1
XM_011544569.1	XM_001656152.2
XM_011544632.2	XM_021838907.1
XM_011544679.3	XM_021841248.1
XM_011544680.3	XM_021855306.1
XM_011544687.1	XM_001659505.2
XM_011544713.2	XM_021855051.1
XM_011544860.3	XM_021849807.1
XM_011544861.1	XM_021843365.1
XM_011544884.2	XM_021846250.1
XM_011544888.2	XM_001648626.2
XM_011544899.1	XM_021840735.1
XM_011544933.3	XM_021843362.1
XM_011544955.3	XM_021853229.1
XM_011545002.2	XM_021843360.1
XM_011545044.2	XR_002502729.1
XM_011545063.3	XM_001655267.2
XM_011545073.1	XM_021847983.1

XM_011545110.1	XM_021843529.1
XM_011545165.3	XM_001653570.2
XM_011545240.2	XM_021837798.1
XM_011545293.2	XM_021842684.1
XM_011545308.3	XM_021847776.1
XM_011545403.2	XM_021841004.1
XM_011545468.2	XM_021857118.1
XM_011545472.3	XM_021843148.1
XM_011545478.2	XM_001649954.2
XM_011545564.3	XM_021838909.1
XM_011545585.2	XM_021838797.1
XM_011545711.2	XM_001652872.3
XM_011545713.1	XM_001657198.2
XM_011545738.1	XM_021838182.1
XM_011545764.1	XM_021854151.1
XM_011545770.1	XM_021843229.1
XM_011545784.3	XM_021842245.1
XM_011545824.3	XM_001660773.2
XM_011545836.3	XM_021838262.1
XM_011545913.2	XM_001655992.2
XM_011546001.3	XM_021848083.1
XM_011546149.2	XR_002502077.1
XM_011546186.1	XM_021855523.1
XM_011547058.2	XM_021847784.1
XM_011547362.3	XM_021853872.1
XM_017000046.1	XM_021850239.1
XM_017000054.1	XM_021841535.1
XM_017000095.2	XM_021850066.1
XM_017000148.2	XM_021846722.1
XM_017000212.2	XM_021849064.1
XM_017000215.2	XM_021845450.1
XM_017000216.1	XM_021853674.1
XM_017000236.2	XM_021837906.1
XM_017000256.2	XM_001663439.2
XM_017000272.1	XM_001658937.2
XM_017000284.1	XM_021850825.1
XM_017000307.1	XM_021838224.1
XM_017000339.1	XM_021838868.1
XM_017000395.1	XM_021845708.1
XM_017000451.1	XM_021848508.1
XM_017000466.1	XM_021847469.1
XM_017000495.1	XM_021837915.1
XM_017000509.2	XM_021846377.1
XM_017000544.2	XM_021846321.1
XM_017000597.1	XM_001649468.2
XM_017000622.1	XM_021852721.1
XM_017000623.2	XM_021848199.1
XM_017000624.2	XM_021841003.1
XM_017000665.1	XM_021850069.1
XM_017000691.1	XR_002498777.1

XM_017000712.1	XM_021843363.1
XM_017000717.1	XM_021848601.1
XM_017000758.1	XM_021845748.1
XM_017000769.2	XM_001654074.2
XM_017000777.1	XM_021852511.1
XM_017000781.1	XM_001657405.2
XM_017000805.2	XM_021841243.1
XM_017000836.1	XM_021839411.1
XM_017000954.2	XM_001657568.2
XM_017000976.1	XM_001651296.3
XM_017000977.1	XM_021857609.1
XM_017000994.2	XM_001648911.2
XM_017001000.2	XM_021851576.1
XM_017001044.1	XR_002499818.1
XM_017001047.1	XM_021850347.1
XM_017001051.1	XM_021855052.1
XM_017001053.1	XM_021851155.1
XM_017001061.2	XM_001653437.2
XM_017001074.1	XM_021852319.1
XM_017001087.2	XM_021850889.1
XM_017001192.2	XM_001662418.2
XM_017001206.1	XM_001662326.2
XM_017001238.1	XM_021843224.1
XM_017001245.2	XM_001651234.2
XM_017001254.1	XM_001661956.2
XM_017001294.1	XM_001663878.2
XM_017001306.2	XM_021853027.1
XM_017001307.2	XM_021841829.1
XM_017001317.1	XM_021848129.1
XM_017001333.1	XM_021855661.1
XM_017001383.1	XM_001660960.2
XM_017001392.2	XM_001662295.2
XM_017001407.1	XM_021853453.1
XM_017001428.1	XM_021851494.1
XM_017001514.1	XM_021849811.1
XM_017001527.1	XM_021854947.1
XM_017001534.1	XM_021840923.1
XM_017001541.2	XM_021839237.1
XM_017001567.1	XM_021849613.1
XM_017001618.1	XM_021855927.1
XM_017001626.1	XM_021840722.1
XM_017001630.1	XM_021842722.1
XM_017001690.1	XM_001655238.2
XM_017001694.1	XM_021850234.1
XM_017001705.1	XM_021842714.1
XM_017001751.1	XM_021850542.1
XM_017001788.1	XM_001654404.2
XM_017001869.1	XM_021842495.1
XM_017001905.1	XM_001655387.2
XM_017001939.1	XM_001654754.2

XM_017002089.2	XM_021849413.1
XM_017002094.2	XM_021838287.1
XM_017002098.2	XM_021837239.1
XM_017002099.2	XM_021847417.1
XM_017002177.1	XM_001661489.2
XM_017002185.1	XM_021857128.1
XM_017002241.1	XM_021846307.1
XM_017002252.2	XM_001651159.2
XM_017002260.2	XR_002502909.1
XM_017002264.2	XM_021844576.1
XM_017002302.1	XM_001655469.2
XM_017002479.1	XM_021842789.1
XM_017002550.2	XM_021842779.1
XM_017002685.2	XM_021852269.1
XM_017002767.1	XM_001648651.2
XM_017002774.1	XM_021839392.1
XM_017002785.2	XM_021840738.1
XM_017002928.2	XM_021848862.1
XM_017002929.1	XR_002500881.1
XM_017002933.2	XM_021840710.1
XM_017002946.2	XM_021847137.1
XM_017002955.1	XM_021849949.1
XM_017002997.1	XM_021839924.1
XM_017003042.2	XM_021847856.1
XM_017003093.1	XM_001650002.2
XM_017003105.2	XM_001656181.2
XM_017003113.1	XM_021837991.1
XM_017003116.1	XM_021845386.1
XM_017003159.2	XM_001657485.2
XM_017003196.2	XM_021838956.1
XM_017003220.1	XM_001658400.2
XM_017003264.2	XR_002499551.1
XM_017003297.1	XM_021846082.1
XM_017003309.1	XM_001655489.2
XM_017003314.2	XM_021843219.1
XM_017003365.1	XM_001651123.2
XM_017003382.2	XM_021854811.1
XM_017003396.1	XR_002499097.1
XM_017003399.2	XM_021847413.1
XM_017003402.1	XM_021839048.1
XM_017003410.1	XM_001654484.2
XM_017003426.2	XM_001647518.2
XM_017003432.2	XM_001650865.2
XM_017003447.1	XM_021838792.1
XM_017003455.2	XM_021851207.1
XM_017003475.2	XM_021839134.1
XM_017003479.1	XM_001663856.2
XM_017003480.2	XM_021853339.1
XM_017003488.2	XM_021839138.1
XM_017003503.1	XM_021837797.1

XM_017003526.1	XM_021842947.1
XM_017003527.1	XM_001652474.2
XM_017003528.1	XM_021856418.1
XM_017003548.1	XM_021856740.1
XM_017003549.2	XM_021852914.1
XM_017003576.1	XM_001661737.2
XM_017003604.1	XM_021854770.1
XM_017003606.1	XM_021854932.1
XM_017003622.1	XM_001650152.2
XM_017003659.1	XM_021842499.1
XM_017003664.1	XM_021850002.1
XM_017003667.1	XM_021838112.1
XM_017003670.1	XM_021846452.1
XM_017003683.2	XM_021856768.1
XM_017003700.1	XM_001654510.2
XM_017003716.1	XM_021856844.1
XM_017003722.1	XM_021841096.1
XM_017003771.1	XM_021855842.1
XM_017003818.1	XM_021855050.1
XM_017003833.2	XM_021855841.1
XM_017003834.1	XM_021847621.1
XM_017003838.1	XM_001648453.2
XM_017003890.1	XM_001649489.2
XM_017003897.1	XM_001661230.2
XM_017003925.1	XM_021852388.1
XM_017003930.1	XR_002501038.1
XM_017003996.1	XM_021856926.1
XM_017004007.1	XM_021837978.1
XM_017004009.1	XM_021848023.1
XM_017004034.1	XM_021857351.1
XM_017004062.1	XM_001664202.2
XM_017004082.2	XM_021847895.1
XM_017004086.2	XM_021846256.1
XM_017004174.1	XR_002501860.1
XM_017004236.1	XM_021848411.1
XM_017004276.1	XR_002501252.1
XM_017004312.2	XR_002498742.1
XM_017004343.2	XM_001655843.2
XM_017004366.1	XM_021838478.1
XM_017004379.2	XM_021839335.1
XM_017004401.2	XM_021843395.1
XM_017004403.1	XM_021850235.1
XM_017004432.2	XM_021856677.1
XM_017004447.1	XM_021849533.1
XM_017004492.2	XM_001657766.2
XM_017004514.2	XM_021844296.1
XM_017004557.1	XM_021844944.1
XM_017004575.2	XM_001663566.2
XM_017004624.2	XM_021847896.1
XM_017004656.1	XM_021846255.1

XM_017004664.1	XM_021848682.1
XM_017004669.1	XM_021852357.1
XM_017004701.1	XR_002499156.1
XM_017004738.1	XM_021842417.1
XM_017004824.1	XM_021855857.1
XM_017004866.1	XM_021852132.1
XM_017004891.1	XM_021839398.1
XM_017004920.2	XM_021857004.1
XM_017004956.2	XR_002501861.1
XM_017004979.2	XM_021853021.1
XM_017004999.1	XM_001654238.2
XM_017005076.2	XM_021842027.1
XM_017005088.1	XM_021851534.1
XM_017005092.2	XM_021846251.1
XM_017005146.1	XM_021847035.1
XM_017005160.2	XM_021847255.1
XM_017005187.2	XM_021839962.1
XM_017005219.2	XM_001655268.2
XM_017005235.1	XM_001651748.2
XM_017005385.1	XM_001659059.2
XM_017005469.2	XM_021857269.1
XM_017005476.1	XM_001659938.2
XM_017005522.1	XR_002501972.1
XM_017005653.1	XM_021847231.1
XM_017005690.1	XM_001655713.2
XM_017005694.2	XM_001650175.2
XM_017005699.1	XM_021847897.1
XM_017005702.1	XM_021839928.1
XM_017005731.1	XM_021856375.1
XM_017005744.2	XM_021843142.1
XM_017005756.1	XM_021842668.1
XM_017005762.2	XM_001661814.2
XM_017005766.2	XM_021855064.1
XM_017005785.1	XM_001652195.2
XM_017005796.1	XM_021842092.1
XM_017005811.2	XM_021850142.1
XM_017005813.1	XM_021843411.1
XM_017005823.2	XM_021838800.1
XM_017005861.1	XR_002502309.1
XM_017005894.1	XM_021847464.1
XM_017005907.2	XM_021838080.1
XM_017005927.1	XM_021851864.1
XM_017005946.2	XM_001654117.2
XM_017005993.1	XM_021838894.1
XM_017006035.1	XM_021847342.1
XM_017006041.2	XM_021855193.1
XM_017006048.1	XM_001659233.2
XM_017006071.2	XM_001650571.2
XM_017006080.2	XM_021853431.1
XM_017006083.2	XM_021849371.1

XM_017006091.1	XM_021857339.1
XM_017006103.1	XM_001656795.2
XM_017006122.1	XM_001661715.3
XM_017006123.1	XM_021840458.1
XM_017006139.1	XM_021850547.1
XM_017006144.1	XR_002500716.1
XM_017006147.1	XM_001650577.2
XM_017006157.1	XM_021840854.1
XM_017006172.2	XM_021852082.1
XM_017006199.1	XM_021841352.1
XM_017006205.1	XM_021851730.1
XM_017006218.1	XM_021853673.1
XM_017006257.1	XM_021855828.1
XM_017006285.1	XM_021840985.1
XM_017006299.1	XM_001652537.2
XM_017006368.1	XR_002502105.1
XM_017006386.1	XM_021847857.1
XM_017006484.2	XM_001659332.2
XM_017006520.1	XM_021846953.1
XM_017006612.2	XM_001664043.2
XM_017006624.1	XM_021848010.1
XM_017006629.1	XM_021842657.1
XM_017006632.1	XM_021838884.1
XM_017006645.2	XM_021837305.1
XM_017006709.1	XM_001657157.2
XM_017006724.1	XM_021845454.1
XM_017006746.2	XM_021851981.1
XM_017006759.2	XM_001659374.2
XM_017006776.1	XM_021855859.1
XM_017006782.1	XM_001659267.2
XM_017006787.2	XM_021853491.1
XM_017006797.2	XM_021856309.1
XM_017006818.2	XM_001656627.2
XM_017006825.2	XM_021852987.1
XM_017006827.2	XM_021855295.1
XM_017006878.2	XM_021850025.1
XM_017006929.2	XM_021847894.1
XM_017006932.2	XM_021849279.1
XM_017006951.2	XM_021840984.1
XM_017006963.2	XM_021847775.1
XM_017006982.1	XM_021845455.1
XM_017007015.1	XM_001661096.2
XM_017007017.1	XM_021855061.1
XM_017007062.1	XM_021848856.1
XM_017007141.1	XR_002501475.1
XM_017007158.2	XM_021854768.1
XM_017007161.2	XM_001652785.2
XM_017007201.1	XM_021846577.1
XM_017007208.1	XM_021855141.1
XM_017007300.1	XM_021840800.1

XM_017007347.1	XM_001651839.2
XM_017007375.1	XM_021844678.1
XM_017007379.2	XM_021841468.1
XM_017007436.1	XM_021838479.1
XM_017007444.1	XM_021844905.1
XM_017007523.2	XM_021849275.1
XM_017007526.2	XM_001659177.2
XM_017007534.1	XM_021851285.1
XM_017007552.2	XR_002502218.1
XM_017007571.1	XM_021837658.1
XM_017007589.1	XM_021855843.1
XM_017007639.2	XM_021844396.1
XM_017007648.2	XM_021849929.1
XM_017007709.2	XM_021854969.1
XM_017007720.1	XR_002500255.1
XM_017007722.1	XM_021853752.1
XM_017007741.2	XM_021856562.1
XM_017007798.1	XM_021855662.1
XM_017007799.1	XM_001649030.2
XM_017007805.2	XM_021850767.1
XM_017007809.1	XM_021848982.1
XM_017007892.2	XM_021851633.1
XM_017007903.1	XM_001658168.2
XM_017007911.1	XM_001664160.2
XM_017007913.2	XM_021854277.1
XM_017007938.1	XM_001660480.2
XM_017007944.1	XM_021850513.1
XM_017007958.1	XM_001661427.2
XM_017007985.1	XM_021842078.1
XM_017007994.1	XM_021851760.1
XM_017008009.1	XM_021838698.1
XM_017008016.2	XM_021840497.1
XM_017008030.2	XM_011494732.2
XM_017008130.1	XM_021850826.1
XM_017008152.2	XM_021847246.1
XM_017008189.1	XM_021849383.1
XM_017008199.1	XM_001651722.2
XM_017008210.2	XM_021842093.1
XM_017008224.1	XM_021849683.1
XM_017008295.2	XR_002500720.1
XM_017008319.1	XM_021845795.1
XM_017008341.1	XM_021847770.1
XM_017008353.1	XR_002500041.1
XM_017008371.1	XM_021842903.1
XM_017008402.1	XM_021849823.1
XM_017008474.1	XM_021842847.1
XM_017008491.1	XM_021839054.1
XM_017008508.1	XM_021848172.1
XM_017008511.2	XM_021853960.1
XM_017008514.2	XM_021839476.1

XM_017008515.2	XM_001660235.2
XM_017008544.2	XM_001659211.2
XM_017008563.1	XM_001663685.2
XM_017008568.1	XM_001653766.2
XM_017008587.1	XM_021856304.1
XM_017008593.2	XM_021851421.1
XM_017008594.2	XM_021843953.1
XM_017008612.2	XM_021846278.1
XM_017008630.1	XM_021854779.1
XM_017008699.1	XM_001654945.2
XM_017008717.2	XM_021854904.1
XM_017008735.1	XM_021840807.1
XM_017008736.1	XM_021852767.1
XM_017008737.1	XM_021841689.1
XM_017008773.2	XM_021847921.1
XM_017008820.2	XM_021852996.1
XM_017008834.1	XM_021849948.1
XM_017008864.2	XM_021856646.1
XM_017008884.1	XM_021838492.1
XM_017008888.1	XM_021848378.1
XM_017008929.2	XM_021852652.1
XM_017008947.2	XM_001649964.2
XM_017008980.2	XM_021855827.1
XM_017008984.2	XM_001658236.2
XM_017008985.2	XM_001655297.2
XM_017009007.1	XM_021851896.1
XM_017009014.1	XM_021854302.1
XM_017009019.2	XM_021848167.1
XM_017009037.2	XR_002500488.1
XM_017009043.2	XM_021840709.1
XM_017009085.1	XM_021842574.1
XM_017009097.2	XM_021842681.1
XM_017009112.2	XM_001658956.2
XM_017009126.2	XM_021857164.1
XM_017009134.1	XM_021854225.1
XM_017009149.2	XR_002499184.1
XM_017009161.2	XM_021849372.1
XM_017009168.1	XR_002500049.1
XM_017009172.1	XM_021844845.1
XM_017009186.1	XM_021841390.1
XM_017009204.1	XM_021837971.1
XM_017009241.2	XM_021847691.1
XM_017009250.2	XM_021842442.1
XM_017009263.1	XM_021840705.1
XM_017009265.1	XR_002499259.1
XM_017009293.2	XM_001651712.2
XM_017009315.2	XM_001649882.3
XM_017009322.2	XM_021841491.1
XM_017009363.1	XM_021848473.1
XM_017009439.2	XM_001658581.2

XM_017009449.1	XM_021846272.1
XM_017009460.1	XM_001655335.2
XM_017009469.1	XM_021845237.1
XM_017009470.2	XM_021846808.1
XM_017009473.1	XM_021857356.1
XM_017009491.1	XM_021857473.1
XM_017009497.2	XM_001651052.2
XM_017009544.2	XM_021849209.1
XM_017009621.2	XM_021848293.1
XM_017009630.1	XM_021847685.1
XM_017009657.1	XM_001659810.2
XM_017009664.1	XM_021847854.1
XM_017009725.2	XM_001661276.2
XM_017009740.1	XM_021847576.1
XM_017009766.1	XM_001652555.2
XM_017009792.2	XM_021842318.1
XM_017009814.1	XM_021854278.1
XM_017009858.2	XR_002502574.1
XM_017009869.1	XM_021838319.1
XM_017009872.1	XM_021853613.1
XM_017009886.2	XM_021841504.1
XM_017010000.1	XM_021838699.1
XM_017010009.1	XM_021849127.1
XM_017010061.1	XR_002499640.1
XM_017010072.2	XM_021856644.1
XM_017010087.2	XM_021845336.1
XM_017010099.2	XM_021846279.1
XM_017010115.2	XM_001657083.2
XM_017010164.2	XM_021841812.1
XM_017010199.2	XM_001663329.2
XM_017010202.1	XR_002501786.1
XM_017010205.2	XR_002499253.1
XM_017010217.2	XM_021848919.1
XM_017010278.2	XM_021857476.1
XM_017010300.2	XM_021849675.1
XM_017010339.1	XR_002499533.1
XM_017010374.2	XM_021853665.1
XM_017010409.1	XM_001660854.2
XM_017010440.2	XR_002499260.1
XM_017010441.2	XM_021843842.1
XM_017010472.1	XR_002499543.1
XM_017010588.1	XM_021848360.1
XM_017010598.2	XM_021855053.1
XM_017010629.2	XM_021850462.1
XM_017010632.2	XM_001649426.2
XM_017010636.2	XM_001650588.2
XM_017010663.2	XM_021855890.1
XM_017010712.1	XR_002499441.1
XM_017010714.2	XM_021843380.1
XM_017010721.1	XM_021842846.1

XM_017010736.2	XR_002502360.1
XM_017010744.2	XM_021845120.1
XM_017010752.1	XM_001653113.2
XM_017010809.2	XM_001662276.2
XM_017010812.1	XM_021840983.1
XM_017010830.1	XM_021840464.1
XM_017010882.1	XM_021837839.1
XM_017010912.2	XM_021841065.1
XM_017010929.2	XM_001656182.2
XM_017010949.2	XM_021837842.1
XM_017010985.1	XM_021849175.1
XM_017011000.1	XM_001659936.2
XM_017011001.1	XM_021839929.1
XM_017011046.1	XM_021839238.1
XM_017011050.1	XM_021853666.1
XM_017011052.1	XM_021857660.1
XM_017011054.1	XM_021848474.1
XM_017011105.2	XM_021857454.1
XM_017011139.2	XM_021857684.1
XM_017011174.2	XM_021855054.1
XM_017011227.1	XM_021855926.1
XM_017011235.2	XM_021846324.1
XM_017011243.2	XM_021851959.1
XM_017011248.2	XM_021841176.1
XM_017011278.2	XM_021850149.1
XM_017011305.2	XM_021855060.1
XM_017011321.1	XM_001661331.2
XM_017011348.1	XM_021841759.1
XM_017011403.1	XM_021840364.1
XM_017011426.2	XM_021855140.1
XM_017011448.2	XM_001654688.2
XM_017011450.2	XM_021852270.1
XM_017011462.1	XM_001662653.2
XM_017011466.2	XM_021837834.1
XM_017011503.1	XM_021841580.1
XM_017011560.2	XM_021843780.1
XM_017011562.2	XM_021840821.1
XM_017011662.2	XM_021841534.1
XM_017011722.1	XM_021837539.1
XM_017011739.1	XM_001655554.2
XM_017011757.1	XR_002502907.1
XM_017011761.2	XM_021853425.1
XM_017011795.1	XM_021840319.1
XM_017011813.1	XM_021838493.1
XM_017011864.1	XM_021848921.1
XM_017011874.1	XM_001657407.2
XM_017011888.1	XM_021839338.1
XM_017011905.2	XM_021844675.1
XM_017011929.2	XM_021846160.1
XM_017011937.1	XR_002501447.1

XM_017011972.1	XM_021846796.1
XM_017011974.1	XM_021853353.1
XM_017012006.2	XM_021847613.1
XM_017012016.2	XM_001658119.2
XM_017012038.2	XR_002503217.1
XM_017012040.2	XM_021854967.1
XM_017012060.2	XM_021850456.1
XM_017012076.1	XM_021840304.1
XM_017012080.1	XM_001658746.2
XM_017012114.1	XM_021837450.1
XM_017012136.2	XR_002502075.1
XM_017012182.1	XM_001652304.2
XM_017012226.1	XM_021842217.1
XM_017012227.1	XM_021842332.1
XM_017012230.1	XR_002500408.1
XM_017012294.1	XM_021855102.1
XM_017012298.2	XM_001648484.2
XM_017012335.2	XM_021849260.1
XM_017012388.1	XM_021857358.1
XM_017012452.1	XM_021845589.1
XM_017012492.2	XM_021852536.1
XM_017012506.2	XM_001655132.2
XM_017012540.1	XM_021847855.1
XM_017012616.2	XM_001656766.2
XM_017012659.1	XM_001662583.2
XM_017012738.1	XR_002502146.1
XM_017012746.1	XM_021843347.1
XM_017012750.2	XM_001654267.2
XM_017012760.2	XM_001648588.3
XM_017012802.1	XM_021838630.1
XM_017012820.1	XM_001661368.2
XM_017012845.2	XR_002502380.1
XM_017012878.2	XM_001655326.2
XM_017012938.1	XM_021844603.1
XM_017012944.1	XM_021851154.1
XM_017012959.1	XR_002500655.1
XM_017012994.2	XM_001659696.2
XM_017012998.2	XM_021853161.1
XM_017013021.2	XM_021838837.1
XM_017013048.1	XM_021852074.1
XM_017013171.1	XM_021846995.1
XM_017013263.1	XM_021847277.1
XM_017013268.2	XM_021841269.1
XM_017013289.1	XM_021857002.1
XM_017013306.1	XM_001652431.3
XM_017013310.2	XM_001653853.2
XM_017013323.1	XM_021849907.1
XM_017013373.1	XM_021849132.1
XM_017013376.2	XR_002498740.1
XM_017013415.2	XM_021837961.1

XM_017013416.1	XM_021850135.1
XM_017013428.2	XM_021850349.1
XM_017013467.2	XM_021854344.1
XM_017013516.1	XM_021847006.1
XM_017013535.1	XM_021856630.1
XM_017013567.2	XM_021843745.1
XM_017013576.1	XM_021854080.1
XM_017013604.2	XM_001658131.2
XM_017013645.1	XM_021844589.1
XM_017013656.2	XM_021845351.1
XM_017013693.1	XM_021852492.1
XM_017013705.2	XR_002502518.1
XM_017013755.1	XM_021846842.1
XM_017013780.1	XM_021855499.1
XM_017013824.2	XM_021854079.1
XM_017013856.2	XM_021847113.1
XM_017013905.1	XM_021856133.1
XM_017013934.1	XM_021843531.1
XM_017013942.1	XM_021852816.1
XM_017013979.2	XM_001657762.2
XM_017013984.1	XM_021841581.1
XM_017013995.2	XM_021852997.1
XM_017014001.2	XM_021838809.1
XM_017014028.2	XM_021837364.1
XM_017014091.1	XM_021857388.1
XM_017014117.2	XM_001651905.2
XM_017014171.1	XM_021838980.1
XM_017014182.1	XM_021843539.1
XM_017014194.1	XR_002502393.1
XM_017014222.1	XM_021849133.1
XM_017014237.2	XM_021845951.1
XM_017014244.2	XM_021856026.1
XM_017014268.1	XM_021846423.1
XM_017014269.2	XM_021844886.1
XM_017014279.1	XM_021839286.1
XM_017014300.1	XM_021851208.1
XM_017014340.1	XM_021856868.1
XM_017014428.1	XM_021837616.1
XM_017014447.1	XM_001650388.2
XM_017014501.2	XM_021838652.1
XM_017014508.1	XR_002499829.1
XM_017014517.2	XM_021853924.1
XM_017014548.2	XR_002502368.1
XM_017014557.1	XM_021856620.1
XM_017014568.1	XM_021839933.1
XM_017014571.1	XR_002503208.1
XM_017014586.1	XM_001650147.2
XM_017014588.1	XM_001651459.2
XM_017014594.2	XM_021853003.1
XM_017014597.2	XM_001650268.2

XM_017014666.2	XM_021847924.1
XM_017014671.1	XM_021852538.1
XM_017014720.1	XM_001662585.2
XM_017014753.2	XM_021839240.1
XM_017014792.1	XM_021848475.1
XM_017014803.1	XM_021841075.1
XM_017014804.1	XM_021841853.1
XM_017014943.2	XM_021840533.1
XM_017014951.2	XM_001657073.2
XM_017014969.2	XM_001661150.2
XM_017015139.2	XM_021853344.1
XM_017015213.1	XM_001657231.2
XM_017015225.1	XM_001650445.2
XM_017015227.1	XM_021844606.1
XM_017015240.2	XM_021842333.1
XM_017015241.2	XM_021851446.1
XM_017015253.1	XM_001660052.2
XM_017015312.2	XM_021843377.1
XM_017015316.1	XM_021853594.1
XM_017015355.2	XM_021841683.1
XM_017015360.2	XM_021844590.1
XM_017015380.1	XM_021840826.1
XM_017015419.1	XM_021839053.1
XM_017015453.1	XM_021846195.1
XM_017015454.1	XM_021837807.1
XM_017015512.1	XM_021849024.1
XM_017015540.1	XM_021847227.1
XM_017015572.1	XM_021849684.1
XM_017015574.2	XM_021845733.1
XM_017015584.2	XR_002500734.1
XM_017015604.1	XM_021849131.1
XM_017015617.1	XR_002501333.1
XM_017015623.1	XM_021848996.1
XM_017015630.1	XR_002498841.1
XM_017015674.1	XM_001658212.2
XM_017015687.2	XM_021845058.1
XM_017015703.2	XM_021838411.1
XM_017015712.1	XM_001660552.3
XM_017015740.2	XM_001649559.2
XM_017015831.2	XM_021837228.1
XM_017015849.1	XM_021847321.1
XM_017015855.1	XM_021847103.1
XM_017015878.1	XM_021845958.1
XM_017015928.1	XR_002499104.1
XM_017015982.1	XM_021841204.1
XM_017016042.1	XM_021848964.1
XM_017016073.1	XM_001662419.2
XM_017016100.1	XM_021840360.1
XM_017016130.1	XM_021851616.1
XM_017016164.1	XM_001649410.2

XM_017016170.1	XM_021842099.1
XM_017016180.1	XM_001663131.2
XM_017016182.1	XM_001652741.2
XM_017016209.2	XR_002498897.1
XM_017016210.2	XM_021854476.1
XM_017016225.2	XM_021855333.1
XM_017016328.2	XM_001662970.2
XM_017016345.2	XM_021853751.1
XM_017016381.2	XM_001662901.2
XM_017016402.1	XM_021849208.1
XM_017016438.2	XM_021848986.1
XM_017016453.1	XM_021856163.1
XM_017016480.1	XM_001661522.2
XM_017016496.2	XM_011495107.2
XM_017016498.1	XM_001660323.2
XM_017016511.1	XM_021854676.1
XM_017016546.2	XR_002500021.1
XM_017016622.1	XM_001651045.2
XM_017016632.2	XM_021849571.1
XM_017016637.1	XM_021852760.1
XM_017016687.1	XM_021840358.1
XM_017016715.2	XM_021857451.1
XM_017016789.2	XM_021852196.1
XM_017016840.1	XM_021845735.1
XM_017016853.2	XM_021841193.1
XM_017016868.1	XM_001663790.2
XM_017016912.2	XM_021839664.1
XM_017016941.1	XM_021838982.1
XM_017016949.2	XR_002501765.1
XM_017016962.1	XM_001662516.2
XM_017016973.1	XR_002499810.1
XM_017017067.1	XM_021848152.1
XM_017017082.2	XM_001661829.2
XM_017017087.1	XM_021847478.1
XM_017017095.1	XM_021848966.1
XM_017017112.2	XM_021840529.1
XM_017017137.1	XM_021845186.1
XM_017017144.2	XM_021838949.1
XM_017017146.2	XM_001653614.2
XM_017017212.1	XM_021839096.1
XM_017017241.2	XM_021845971.1
XM_017017280.2	XM_021837970.1
XM_017017361.1	XM_021853711.1
XM_017017367.1	XM_021848959.1
XM_017017373.2	XM_021853580.1
XM_017017399.1	XM_021847923.1
XM_017017402.1	XM_001657778.2
XM_017017412.1	XM_001651506.2
XM_017017420.1	XM_021842776.1
XM_017017431.1	XM_021838984.1

XM_017017462.2	XM_021852258.1
XM_017017512.1	XM_021843491.1
XM_017017520.1	XM_001649224.2
XM_017017574.1	XM_021846227.1
XM_017017644.1	XM_021854810.1
XM_017017672.1	XM_021850750.1
XM_017017723.1	XM_021846152.1
XM_017017738.2	XM_021854435.1
XM_017017781.1	XR_002499174.1
XM_017017840.1	XM_021838722.1
XM_017017843.2	XM_021856356.1
XM_017017894.1	XM_021840359.1
XM_017017912.1	XM_021838455.1
XM_017017923.1	XM_021849979.1
XM_017017926.1	XR_002501466.1
XM_017017932.1	XM_021840649.1
XM_017017949.2	XM_001648338.2
XM_017018004.1	XM_021849742.1
XM_017018045.1	XM_001656155.2
XM_017018101.2	XM_021843541.1
XM_017018128.1	XM_021845433.1
XM_017018133.1	XM_021854922.1
XM_017018139.1	XM_021845874.1
XM_017018142.1	XM_021840534.1
XM_017018145.1	XM_021853709.1
XM_017018146.1	XM_021838633.1
XM_017018180.1	XM_021852769.1
XM_017018199.1	XM_021839428.1
XM_017018206.1	XM_021855218.1
XM_017018212.1	XM_021845348.1
XM_017018215.1	XM_021855224.1
XM_017018238.1	XM_001649472.2
XM_017018303.1	XM_021856365.1
XM_017018345.1	XM_021849349.1
XM_017018367.1	XM_021847101.1
XM_017018376.2	XM_001657686.2
XM_017018415.2	XM_021855824.1
XM_017018458.1	XM_021846886.1
XM_017018474.2	XR_002499197.1
XM_017018537.1	XM_021853430.1
XM_017018566.1	XM_021842752.1
XM_017018570.1	XR_002502148.1
XM_017018666.2	XM_021844408.1
XM_017018708.1	XM_021840220.1
XM_017018798.2	XM_021851615.1
XM_017018836.1	XM_021857129.1
XM_017018905.1	XM_021845868.1
XM_017018937.1	XM_021838031.1
XM_017018956.1	XM_021850352.1
XM_017018963.2	XM_021850148.1

XM_017018973.1	XM_021851490.1
XM_017018981.2	XM_021857383.1
XM_017019010.1	XM_021837963.1
XM_017019020.1	XM_021842796.1
XM_017019037.2	XM_021840366.1
XM_017019057.1	XR_002499074.1
XM_017019071.1	XM_021856361.1
XM_017019130.2	XM_021848007.1
XM_017019152.2	XM_001656747.2
XM_017019172.1	XM_021841685.1
XM_017019217.1	XM_021852750.1
XM_017019229.2	XM_021845519.1
XM_017019237.1	XM_001655348.2
XM_017019240.2	XM_001657075.2
XM_017019248.1	XM_021848958.1
XM_017019252.2	XM_021845818.1
XM_017019270.1	XM_021853133.1
XM_017019276.1	XM_021848369.1
XM_017019288.2	XM_001657650.2
XM_017019297.1	XM_021846578.1
XM_017019322.1	XM_021841268.1
XM_017019335.1	XM_011494742.2
XM_017019352.1	XM_001652603.2
XM_017019442.2	XM_021840979.1
XM_017019463.2	XM_021839750.1
XM_017019466.2	XM_001660992.2
XM_017019469.1	XM_021849642.1
XM_017019473.2	XM_021837941.1
XM_017019535.1	XM_021839386.1
XM_017019572.1	XM_021843714.1
XM_017019591.1	XM_001659291.2
XM_017019606.1	XM_021840437.1
XM_017019626.2	XM_021851516.1
XM_017019644.1	XM_001655286.2
XM_017019652.2	XM_021849602.1
XM_017019739.2	XM_021857080.1
XM_017019767.1	XM_021843639.1
XM_017019775.2	XR_002501280.1
XM_017019782.1	XM_001656480.2
XM_017019783.2	XM_021855311.1
XM_017019800.1	XM_021839135.1
XM_017019815.1	XM_001653194.2
XM_017019836.1	XM_021840990.1
XM_017019962.2	XM_001661224.3
XM_017019969.2	XM_001662166.2
XM_017019991.2	XM_021848881.1
XM_017020016.2	XM_021840970.1
XM_017020021.1	XM_021855893.1
XM_017020023.1	XM_001660526.2
XM_017020052.2	XM_021857548.1

XM_017020069.2	XM_021847348.1
XM_017020092.2	XM_021841761.1
XM_017020141.1	XM_021856536.1
XM_017020145.1	XM_001654760.2
XM_017020151.1	XM_021841426.1
XM_017020185.2	XM_001659729.2
XM_017020207.1	XM_021837550.1
XM_017020218.1	XM_021845900.1
XM_017020219.1	XM_001651413.2
XM_017020241.2	XM_001656292.2
XM_017020243.1	XM_021841549.1
XM_017020255.2	XM_001652877.3
XM_017020262.1	XM_021837549.1
XM_017020266.1	XM_021837759.1
XM_017020298.1	XM_001655070.2
XM_017020317.2	XM_021846984.1
XM_017020331.1	XM_001661910.2
XM_017020349.1	XM_021840181.1
XM_017020362.2	XM_021854220.1
XM_017020363.2	XM_021846853.1
XM_017020376.1	XM_001654716.2
XM_017020429.1	XM_021838985.1
XM_017020436.2	XM_001661385.2
XM_017020489.1	XR_002499277.1
XM_017020493.1	XM_021852651.1
XM_017020508.1	XM_001658657.2
XM_017020527.1	XR_002503185.1
XM_017020544.1	XM_021849207.1
XM_017020549.1	XM_021840151.1
XM_017020593.2	XR_002500076.1
XM_017020604.1	XM_021847381.1
XM_017020609.1	XM_021847223.1
XM_017020647.1	XM_021851465.1
XM_017020680.2	XR_002502610.1
XM_017020684.1	XM_021841679.1
XM_017020704.2	XM_001656722.2
XM_017020733.2	XM_021838270.1
XM_017020799.2	XM_021839755.1
XM_017020815.1	XM_021851724.1
XM_017020829.2	XM_021838806.1
XM_017020833.1	XM_021854434.1
XM_017020846.1	XM_021850791.1
XM_017020853.2	XM_021852661.1
XM_017020859.2	XM_001648958.2
XM_017020875.1	XM_021854468.1
XM_017020926.1	XM_021840981.1
XM_017020976.1	XM_021854322.1
XM_017020988.1	XM_001652778.2
XM_017020993.1	XM_001663665.2
XM_017021035.2	XM_021855729.1

XM_017021070.1	XM_021842197.1
XM_017021071.1	XM_021838811.1
XM_017021079.1	XM_001658502.2
XM_017021104.2	XM_021839427.1
XM_017021113.2	XM_021855585.1
XM_017021121.2	XM_021853089.1
XM_017021147.1	XM_021849731.1
XM_017021156.2	XM_021842193.1
XM_017021177.1	XM_021842192.1
XM_017021203.1	XM_021847116.1
XM_017021212.1	XM_021851924.1
XM_017021216.2	XM_001657484.2
XM_017021232.1	XM_021857653.1
XM_017021249.2	XM_021838996.1
XM_017021253.2	XM_001656197.2
XM_017021271.1	XR_002501393.1
XM_017021293.1	XM_021849835.1
XM_017021307.2	XM_021851915.1
XM_017021313.1	XM_021846213.1
XM_017021353.1	XM_021852535.1
XM_017021373.1	XM_001650066.2
XM_017021390.2	XM_001659607.2
XM_017021421.2	XM_021837551.1
XM_017021431.1	XM_021850987.1
XM_017021433.1	XR_002500935.1
XM_017021437.2	XM_001657208.2
XM_017021452.1	XM_021843905.1
XM_017021455.1	XM_001651158.2
XM_017021476.1	XM_021857449.1
XM_017021517.1	XM_001655055.2
XM_017021576.1	XM_021842149.1
XM_017021662.1	XM_021851040.1
XM_017021668.2	XM_001659550.2
XM_017021678.2	XM_021846145.1
XM_017021690.2	XR_002501655.1
XM_017021701.1	XM_021849020.1
XM_017021702.1	XM_021855889.1
XM_017021727.2	XM_021857724.1
XM_017021734.1	XM_001651047.2
XM_017021735.1	XM_001659714.2
XM_017021755.2	XM_021848993.1
XM_017021774.1	XM_001654826.2
XM_017021799.2	XM_021837411.1
XM_017021820.2	XM_001658878.2
XM_017021867.2	XM_001663011.2
XM_017021897.2	XM_001657179.2
XM_017021927.1	XM_021840650.1
XM_017021933.1	XM_021838407.1
XM_017021937.2	XM_001660912.2
XM_017021940.2	XM_021851781.1

XM_017022007.1	XM_021838533.1
XM_017022031.2	XM_021837752.1
XM_017022073.1	XM_021840695.1
XM_017022099.1	XM_021848899.1
XM_017022110.1	XR_002501327.1
XM_017022137.1	XM_021840980.1
XM_017022160.2	XM_021837846.1
XM_017022198.2	XM_021838581.1
XM_017022201.1	XM_001663983.2
XM_017022226.1	XM_021853898.1
XM_017022233.1	XM_021842229.1
XM_017022236.2	XM_001650712.2
XM_017022259.1	XM_021852369.1
XM_017022276.1	XM_021841681.1
XM_017022286.1	XM_001662644.3
XM_017022298.1	XM_021838398.1
XM_017022302.1	XM_021850313.1
XM_017022310.2	XM_001651507.2
XM_017022349.1	XM_001660082.2
XM_017022370.1	XM_021849739.1
XM_017022374.2	XM_021852481.1
XM_017022384.2	XM_021838404.1
XM_017022387.2	XM_021843385.1
XM_017022450.1	XM_021850341.1
XM_017022463.2	XM_021838807.1
XM_017022540.1	XM_021853163.1
XM_017022570.1	XM_021839306.1
XM_017022575.2	XM_021840527.1
XM_017022657.1	XR_002501758.1
XM_017022659.1	XM_021845694.1
XM_017022673.2	XM_021852852.1
XM_017022685.1	XR_002500264.1
XM_017022712.2	XM_021840239.1
XM_017022761.1	XM_021854433.1
XM_017022778.1	XM_001661157.2
XM_017022815.2	XM_021847224.1
XM_017022822.2	XM_021853074.1
XM_017022824.2	XR_002499731.1
XM_017022876.1	XM_001652468.2
XM_017022912.1	XR_002502727.1
XM_017022921.2	XM_021844770.1
XM_017022925.1	XM_001656833.2
XM_017022928.1	XM_021851838.1
XM_017022960.2	XM_001661928.2
XM_017022968.1	XR_002500324.1
XM_017023019.1	XM_001656505.2
XM_017023077.1	XM_021841762.1
XM_017023098.1	XR_002502079.1
XM_017023099.1	XM_001656716.3
XM_017023190.2	XR_002500862.1

XM_017023193.2	XM_021857399.1
XM_017023266.2	XM_021838737.1
XM_017023277.1	XM_021848106.1
XM_017023281.1	XM_021839849.1
XM_017023297.2	XM_021837894.1
XM_017023303.2	XM_021844578.1
XM_017023350.1	XM_021837823.1
XM_017023383.2	XM_021856986.1
XM_017023403.1	XM_001656309.2
XM_017023424.2	XM_021847879.1
XM_017023477.1	XM_021848892.1
XM_017023527.1	XM_021853398.1
XM_017023532.1	XM_021857361.1
XM_017023550.1	XM_021839756.1
XM_017023554.1	XM_021840997.1
XM_017023625.1	XM_001648434.2
XM_017023634.2	XM_021855381.1
XM_017023707.2	XM_021857270.1
XM_017023715.1	XM_021848466.1
XM_017023723.2	XM_001661478.2
XM_017023751.1	XM_021857679.1
XM_017023757.1	XM_021842539.1
XM_017023770.1	XM_021840140.1
XM_017023786.2	XM_001664232.2
XM_017023808.1	XM_001657293.2
XM_017023853.2	XR_002498607.1
XM_017023859.2	XM_021845993.1
XM_017023966.2	XM_021837622.1
XM_017023974.1	XM_021850458.1
XM_017023991.1	XM_021845429.1
XM_017024006.1	XM_021838047.1
XM_017024027.1	XM_021853189.1
XM_017024038.2	XM_001649726.2
XM_017024040.2	XM_021857681.1
XM_017024057.1	XM_021837523.1
XM_017024075.2	XM_021838635.1
XM_017024079.1	XM_021855548.1
XM_017024102.1	XM_021850226.1
XM_017024122.1	XM_021853124.1
XM_017024149.2	XM_001652347.2
XM_017024200.1	XM_021838803.1
XM_017024209.1	XM_001660791.2
XM_017024228.1	XM_021841078.1
XM_017024273.1	XM_021852051.1
XM_017024277.1	XM_021850447.1
XM_017024289.2	XM_001663742.2
XM_017024297.1	XR_002503284.1
XM_017024325.1	XM_021839114.1
XM_017024338.1	XM_021841847.1
XM_017024371.1	XM_001658024.2

XM_017024409.1	XM_001657276.2
XM_017024434.1	XM_021839115.1
XM_017024468.2	XR_002498857.1
XM_017024484.1	XR_002498868.1
XM_017024543.2	XR_002502657.1
XM_017024574.1	XM_021840988.1
XM_017024618.1	XR_002501529.1
XM_017024641.2	XM_021855586.1
XM_017024642.1	XM_021846004.1
XM_017024730.2	XM_021856368.1
XM_017024731.2	XM_021855379.1
XM_017024736.1	XM_021855138.1
XM_017024740.1	XM_021857525.1
XM_017024764.2	XM_021838222.1
XM_017024777.1	XM_001663386.2
XM_017024808.1	XM_001662851.2
XM_017024825.1	XM_001653316.2
XM_017024841.1	XM_021848686.1
XM_017024844.2	XM_021849743.1
XM_017024845.2	XM_021849073.1
XM_017024846.2	XM_001657465.2
XM_017024921.2	XM_001652306.2
XM_017024955.1	XM_001647878.2
XM_017024989.1	XM_001652231.2
XM_017025036.2	XM_011494870.2
XM_017025061.1	XM_001650481.2
XM_017025095.1	XM_021855923.1
XM_017025096.1	XM_021854906.1
XM_017025113.1	XM_021842175.1
XM_017025132.2	XM_001660253.2
XM_017025146.1	XM_021850665.1
XM_017025159.2	XM_021851995.1
XM_017025166.2	XM_021854895.1
XM_017025170.1	XM_021841538.1
XM_017025231.1	XM_021848236.1
XM_017025233.1	XM_021842012.1
XM_017025305.1	XM_001656366.2
XM_017025350.1	XM_021851564.1
XM_017025374.2	XM_001651438.2
XM_017025377.1	XM_021838134.1
XM_017025397.2	XM_001659743.2
XM_017025409.2	XM_001656502.2
XM_017025441.1	XM_001663051.2
XM_017025451.1	XM_021842572.1
XM_017025453.1	XM_021849108.1
XM_017025454.2	XM_021838781.1
XM_017025514.2	XM_021850476.1
XM_017025530.1	XM_021851843.1
XM_017025561.1	XM_021844018.1
XM_017025574.1	XM_001650801.2

XM_017025599.2	XM_001652367.2
XM_017025663.2	XM_021849304.1
XM_017025669.2	XM_021837615.1
XM_017025684.1	XM_021838790.1
XM_017025716.1	XM_001661129.2
XM_017025728.2	XR_002503147.1
XM_017025787.1	XM_021855918.1
XM_017025803.2	XM_021845979.1
XM_017025808.2	XM_021844840.1
XM_017025894.1	XM_021853164.1
XM_017025927.2	XR_002500500.1
XM_017025967.1	XR_002501319.1
XM_017025993.1	XM_021847790.1
XM_017026007.1	XM_021851839.1
XM_017026035.2	XM_001658610.2
XM_017026086.1	XM_021853598.1
XM_017026099.1	XM_001648855.2
XM_017026150.1	XM_021855182.1
XM_017026170.1	XM_021845435.1
XM_017026172.2	XM_021851644.1
XM_017026191.1	XR_002500798.1
XM_017026200.1	XM_021843980.1
XM_017026202.2	XM_021849837.1
XM_017026203.2	XR_002501965.1
XM_017026210.1	XM_001663809.2
XM_017026213.1	XM_021847221.1
XM_017026216.1	XM_021852853.1
XM_017026243.2	XM_021853672.1
XM_017026246.1	XM_021843536.1
XM_017026262.2	XM_021852540.1
XM_017026269.1	XM_001657109.2
XM_017026324.1	XM_021853129.1
XM_017026354.1	XM_021842074.1
XM_017026411.2	XM_021853791.1
XM_017026445.1	XM_021850744.1
XM_017026456.1	XM_001655545.2
XM_017026470.2	XM_001659214.2
XM_017026523.1	XM_021845547.1
XM_017026530.2	XM_001650645.2
XM_017026541.1	XM_021856031.1
XM_017026551.1	XM_021844796.1
XM_017026558.1	XR_002499315.1
XM_017026573.1	XM_001650118.2
XM_017026601.1	XM_021848363.1
XM_017026643.1	XM_021843384.1
XM_017026711.1	XM_021846215.1
XM_017026726.1	XM_021849430.1
XM_017026746.1	XM_021855373.1
XM_017026752.1	XM_001658980.2
XM_017026771.1	XR_002498578.1

XM_017026798.2	XM_021838758.1
XM_017026855.1	XM_021842803.1
XM_017026857.2	XM_021849636.1
XM_017026928.1	XM_021838282.1
XM_017026942.1	XM_021857355.1
XM_017026983.1	XM_001663411.2
XM_017027020.1	XR_002500082.1
XM_017027040.1	XM_021852818.1
XM_017027058.1	XM_001660394.2
XM_017027074.1	XM_001653536.2
XM_017027077.1	XM_021837259.1
XM_017027094.1	XM_001658778.2
XM_017027096.1	XM_021849652.1
XM_017027113.2	XM_021857286.1
XM_017027139.1	XM_001660279.2
XM_017027153.1	XM_021848855.1
XM_017027204.2	XR_002498725.1
XM_017027220.2	XM_021839906.1
XM_017027231.1	XM_021838905.1
XM_017027258.1	XM_001654414.2
XM_017027297.2	XM_021853036.1
XM_017027305.1	XM_021852488.1
XM_017027312.1	XM_021837423.1
XM_017027316.1	XM_021843545.1
XM_017027327.1	XM_021837413.1
XM_017027334.1	XM_021842797.1
XM_017027358.1	XM_021851639.1
XM_017027362.1	XM_001652110.2
XM_017027374.2	XR_002498995.1
XM_017027411.2	XM_011494914.2
XM_017027428.1	XR_002502337.1
XM_017027430.1	XM_021841131.1
XM_017027451.1	XM_001649703.2
XM_017027453.1	XM_021851201.1
XM_017027461.1	XM_021840287.1
XM_017027464.1	XM_001648178.2
XM_017027481.1	XM_001656842.2
XM_017027522.2	XM_021853701.1
XM_017027529.1	XM_021851914.1
XM_017027532.1	XM_021841500.1
XM_017027554.1	XM_021848730.1
XM_017027565.1	XM_001659344.2
XM_017027592.2	XM_001649222.2
XM_017027600.2	XM_021849437.1
XM_017027602.1	XM_021841744.1
XM_017027628.1	XM_001662619.2
XM_017027646.1	XM_021841351.1
XM_017027647.2	XM_021847936.1
XM_017027667.1	XM_021847193.1
XM_017027678.1	XM_021856905.1

XM_017027692.2	XM_021839140.1
XM_017027736.2	XM_021845526.1
XM_017027744.2	XM_021843543.1
XM_017027754.2	XR_002501068.1
XM_017027784.2	XM_001658307.2
XM_017027803.2	XM_021854062.1
XM_017027827.2	XM_021841881.1
XM_017027894.2	XM_021847069.1
XM_017027915.2	XM_021847071.1
XM_017027951.2	XM_001657604.2
XM_017027969.2	XM_021842421.1
XM_017027980.2	XM_021838372.1
XM_017027982.1	XM_001654041.2
XM_017027998.1	XM_021845442.1
XM_017027999.1	XM_021839566.1
XM_017028064.1	XM_001660072.2
XM_017028096.1	XM_021845576.1
XM_017028101.1	XM_021849404.1
XM_017028109.1	XM_021853702.1
XM_017028155.1	XM_021843661.1
XM_017028160.1	XM_021838777.1
XM_017028258.1	XM_021838269.1
XM_017028271.1	XM_021850486.1
XM_017028344.1	XM_001652585.2
XM_017028362.2	XM_021852714.1
XM_017028415.2	XM_021838090.1
XM_017028440.2	XM_001661396.2
XM_017028451.2	XR_002499058.1
XM_017028459.2	XM_021841353.1
XM_017028474.1	XM_021838989.1
XM_017028485.2	XM_021852489.1
XM_017028510.1	XM_021839244.1
XM_017028546.1	XM_021851513.1
XM_017028556.1	XM_021852046.1
XM_017028560.1	XM_021854839.1
XM_017028580.1	XM_021852899.1
XM_017028612.1	XM_001653774.2
XM_017028637.1	XM_001661887.2
XM_017028643.2	XM_021856380.1
XM_017028667.2	XM_021843540.1
XM_017028698.1	XR_002502468.1
XM_017028747.2	XM_021854413.1
XM_017028769.2	XM_021855171.1
XM_017028774.1	XM_001658057.2
XM_017028788.1	XM_021837241.1
XM_017028809.2	XM_021849516.1
XM_017028814.1	XM_021838006.1
XM_017028825.1	XM_001657295.2
XM_017028836.1	XM_001653378.2
XM_017028839.1	XM_021856150.1

XM_017028896.2	XM_021849757.1
XM_017028920.1	XM_021855353.1
XM_017028955.2	XM_021852062.1
XM_017028957.2	XM_021852493.1
XM_017028971.2	XM_001656760.2
XM_017028975.2	XM_021841952.1
XM_017028976.1	XM_021837613.1
XM_017029015.1	XM_021853986.1
XM_017029035.2	XM_021854412.1
XM_017029053.1	XM_021853623.1
XM_017029099.2	XM_021851330.1
XM_017029119.1	XM_001661501.2
XM_017029130.1	XR_002502325.1
XM_017029221.1	XM_021837408.1
XM_017029228.1	XM_001662327.2
XM_017029364.1	XM_001656284.2
XM_017029391.1	XM_021848507.1
XM_017029416.1	XM_021846331.1
XM_017029418.1	XR_002502667.1
XM_017029450.2	XM_021855911.1
XM_017029464.2	XM_001647574.2
XM_017029469.1	XM_021842552.1
XM_017029479.1	XM_021837440.1
XM_017029491.2	XM_021839514.1
XM_017029513.1	XM_001651723.2
XM_017029523.1	XM_021853122.1
XM_017029531.1	XM_021847734.1
XM_017029571.1	XM_001650885.2
XM_017029575.1	XM_021848460.1
XM_017029578.1	XM_001659416.2
XM_017029585.2	XM_021857391.1
XM_017029588.2	XR_002501395.1
XM_017029590.2	XM_001653009.2
XM_017029602.1	XM_001652157.2
XM_017029619.2	XM_021847867.1
XM_017029631.1	XM_021847251.1
XM_017029647.2	XM_001650794.2
XM_017029662.1	XM_021848724.1
XM_017029675.1	XM_021844771.1
XM_017029679.1	XM_001661840.3
XM_017029681.1	XM_021850666.1
XM_017029689.2	XM_021846124.1
XM_017029708.1	XM_021837370.1
XM_017029726.2	XM_021849433.1
XM_017029740.1	XR_002500035.1
XM_017029762.1	XM_021840762.1
XM_017029770.1	XM_021853080.1
XM_017029783.2	XM_021838369.1
XM_017029817.1	XM_021849926.1
XM_017029821.1	XM_021855363.1

XM_017029872.1	XR_002498688.1
XM_017029873.1	XM_011494794.2
XM_017029890.2	XR_002499010.1
XM_017029893.2	XR_002502479.1
XM_017029911.1	XM_021838991.1
XM_017029916.1	XM_021841141.1
XM_017029920.1	XR_002501830.1
XM_017029968.1	XM_001656672.2
XM_017030103.1	XM_001657162.2
XM_017030116.1	XM_001655281.2
XM_017030122.1	XM_021849273.1
XM_017030301.2	XM_021841263.1
XM_024446078.1	XR_002498626.1
XM_024446080.1	XM_021848071.1
XM_024446127.1	XM_021854070.1
XM_024446137.1	XR_002502916.1
XM_024446138.1	XM_021849252.1
XM_024446148.1	XM_021847959.1
XM_024446149.1	XM_021848221.1
XM_024446155.1	XR_002498848.1
XM_024446163.1	XM_001656286.2
XM_024446181.1	XM_021849283.1
XM_024446216.1	XM_021845747.1
XM_024446243.1	XM_021837606.1
XM_024446265.1	XM_021847067.1
XM_024446333.1	XM_021850271.1
XM_024446354.1	XM_021838954.1
XM_024446367.1	XM_021850187.1
XM_024446378.1	XM_001660173.2
XM_024446385.1	XM_021839031.1
XM_024446424.1	XM_001651536.2
XM_024446428.1	XM_021844799.1
XM_024446430.1	XR_002499374.1
XM_024446431.1	XM_021852971.1
XM_024446468.1	XM_021839145.1
XM_024446524.1	XM_021853275.1
XM_024446529.1	XM_021855385.1
XM_024446533.1	XM_001652374.2
XM_024446618.1	XM_021853626.1
XM_024446636.1	XM_021839241.1
XM_024446655.1	XM_021838046.1
XM_024446664.1	XM_021856985.1
XM_024446671.1	XM_021841395.1
XM_024446692.1	XM_021850977.1
XM_024446709.1	XM_001650312.2
XM_024446726.1	XM_021851845.1
XM_024446748.1	XM_021843583.1
XM_024446783.1	XM_001653977.2
XM_024446786.1	XM_021853928.1
XM_024446817.1	XR_002499833.1

XM_024446820.1	XM_021852464.1
XM_024446838.1	XM_021840309.1
XM_024446876.1	XM_021850831.1
XM_024446881.1	XM_021841769.1
XM_024446888.1	XM_021849137.1
XM_024446896.1	XM_021845744.1
XM_024446963.1	XM_021848409.1
XM_024446969.1	XM_021842736.1
XM_024446980.1	XM_001655096.2
XM_024446986.1	XM_021849670.1
XM_024447053.1	XM_021843713.1
XM_024447054.1	XR_002500671.1
XM_024447055.1	XM_001654743.2
XM_024447057.1	XM_021849140.1
XM_024447086.1	XM_001654657.2
XM_024447113.1	XM_001655050.2
XM_024447120.1	XM_021854783.1
XM_024447136.1	XM_021840975.1
XM_024447140.1	XM_021846695.1
XM_024447141.1	XM_021856832.1
XM_024447228.1	XM_021849203.1
XM_024447266.1	XM_021853376.1
XM_024447270.1	XM_021848516.1
XM_024447272.1	XM_021857471.1
XM_024447295.1	XM_021849353.1
XM_024447308.1	XM_021848721.1
XM_024447311.1	XM_001656546.2
XM_024447315.1	XM_021837783.1
XM_024447354.1	XR_002501320.1
XM_024447367.1	XM_001655604.2
XM_024447377.1	XR_002500127.1
XM_024447380.1	XM_001648896.2
XM_024447384.1	XM_021843880.1
XM_024447434.1	XM_021853784.1
XM_024447506.1	XM_021842697.1
XM_024447538.1	XR_002499477.1
XM_024447549.1	XM_021839156.1
XM_024447573.1	XM_021852898.1
XM_024447630.1	XM_021856353.1
XM_024447632.1	XM_021854396.1
XM_024447681.1	XM_001654227.2
XM_024447699.1	XM_021856839.1
XM_024447735.1	XM_001656267.2
XM_024447736.1	XM_001658837.2
XM_024447763.1	XM_021840105.1
XM_024447765.1	XM_021852042.1
XM_024447767.1	XM_021837792.1
XM_024447784.1	XM_021842674.1
XM_024447830.1	XM_021850479.1
XM_024447865.1	XM_001662010.2

XM_024447867.1	XM_001660419.2
XM_024447868.1	XM_021852733.1
XM_024447902.1	XM_021843902.1
XM_024447967.1	XR_002501547.1
XM_024447981.1	XM_001653543.2
XM_024447986.1	XM_021842526.1
XM_024448026.1	XM_021849195.1
XM_024448038.1	XM_021844607.1
XM_024448081.1	XM_021837646.1
XM_024448083.1	XM_021853320.1
XM_024448086.1	XM_021845419.1
XM_024448100.1	XM_021855356.1
XM_024448120.1	XR_002502800.1
XM_024448143.1	XM_021853342.1
XM_024448156.1	XM_021853351.1
XM_024448170.1	XM_021847034.1
XM_024448223.1	XM_021856373.1
XM_024448241.1	XM_021837899.1
XM_024448243.1	XR_002501879.1
XM_024448257.1	XR_002499227.1
XM_024448284.1	XM_021842128.1
XM_024448286.1	XR_002501099.1
XM_024448287.1	XR_002500701.1
XM_024448303.1	XM_021851447.1
XM_024448319.1	XM_021842038.1
XM_024448321.1	XM_021846243.1
XM_024448322.1	XM_001653256.2
XM_024448341.1	XM_021854373.1
XM_024448342.1	XM_001660112.2
XM_024448358.1	XR_002501276.1
XM_024448381.1	XM_001658127.2
XM_024448385.1	XM_021842980.1
XM_024448408.1	XM_021840841.1
XM_024448426.1	XM_001650482.2
XM_024448492.1	XM_021855482.1
XM_024448494.1	XM_001650817.2
XM_024448501.1	XM_021856907.1
XM_024448510.1	XM_021848725.1
XM_024448536.1	XM_021853321.1
XM_024448572.1	XM_001664087.2
XM_024448689.1	XM_021837811.1
XM_024448694.1	XM_021850983.1
XM_024448739.1	XM_001663784.2
XM_024448740.1	XM_001658162.2
XM_024448741.1	XM_001660267.2
XM_024448767.1	XM_021856972.1
XM_024448772.1	XM_001659220.2
XM_024448776.1	XM_021840627.1
XM_024448806.1	XR_002499695.1
XM_024448832.1	XR_002499488.1

XM_024448861.1	XR_002498850.1
XM_024448869.1	XM_021852897.1
XM_024448885.1	XM_021857394.1
XM_024448954.1	XM_021849580.1
XM_024449008.1	XM_021845590.1
XM_024449013.1	XM_001647845.2
XM_024449022.1	XM_021841276.1
XM_024449024.1	XM_021855376.1
XM_024449047.1	XM_001651917.2
XM_024449052.1	XM_021856854.1
XM_024449059.1	XM_021844856.1
XM_024449073.1	XM_021851422.1
XM_024449085.1	XM_021852418.1
XM_024449096.1	XR_002502819.1
XM_024449112.1	XM_021841885.1
XM_024449130.1	XM_021837588.1
XM_024449134.1	XR_002503018.1
XM_024449152.1	XM_021837393.1
XM_024449190.1	XM_021851286.1
XM_024449213.1	XR_002499598.1
XM_024449229.1	XM_021843733.1
XM_024449252.1	XM_021855939.1
XM_024449270.1	XM_021841502.1
XM_024449275.1	XM_021839951.1
XM_024449278.1	XM_001648394.2
XM_024449290.1	XM_021849010.1
XM_024449300.1	XM_021847217.1
XM_024449312.1	XM_021853596.1
XM_024449326.1	XM_021850072.1
XM_024449373.1	XM_021842566.1
XM_024449390.1	XM_021851210.1
XM_024449420.1	XM_021847092.1
XM_024449425.1	XM_001657186.2
XM_024449470.1	XM_021856845.1
XM_024449477.1	XM_001648705.2
XM_024449495.1	XR_002499105.1
XM_024449520.1	XM_021840043.1
XM_024449532.1	XM_001647895.2
XM_024449600.1	XM_001653340.2
XM_024449618.1	XM_021842126.1
XM_024449636.1	XR_002502550.1
XM_024449646.1	XM_021849669.1
XM_024449649.1	XM_021840635.1
XM_024449659.1	XM_021837416.1
XM_024449699.1	XM_021848753.1
XM_024449704.1	XM_021854254.1
XM_024449717.1	XM_001653734.2
XM_024449721.1	XM_021847347.1
XM_024449739.1	XM_001650987.2
XM_024449744.1	XM_021838290.1

XM_024449748.1	XM_021837787.1
XM_024449781.1	XM_021845984.1
XM_024449813.1	XM_021846388.1
XM_024449823.1	XM_021837270.1
XM_024449889.1	XM_021842747.1
XM_024449896.1	XM_001653317.2
XM_024449912.1	XM_021850902.1
XM_024449930.1	XM_021838307.1
XM_024449946.1	XM_021856765.1
XM_024449957.1	XM_001661906.2
XM_024449959.1	XM_001660295.2
XM_024449977.1	XM_001662877.2
XM_024449992.1	XM_021838133.1
XM_024449995.1	XR_002500452.1
XM_024450008.1	XM_021843284.1
XM_024450013.1	XM_021851009.1
XM_024450055.1	XM_021837455.1
XM_024450070.1	XM_021853920.1
XM_024450075.1	XM_021848893.1
XM_024450112.1	XM_021843153.1
XM_024450134.1	XM_021857360.1
XM_024450147.1	XM_021855955.1
XM_024450151.1	XR_002499165.1
XM_024450153.1	XM_021840736.1
XM_024450213.1	XM_021856230.1
XM_024450219.1	XM_021844280.1
XM_024450233.1	XM_021854479.1
XM_024450271.1	XM_001663233.2
XM_024450280.1	XM_021855711.1
XM_024450289.1	XM_001657096.2
XM_024450306.1	XM_021838125.1
XM_024450308.1	XR_002499677.1
XM_024450328.1	XM_021840768.1
XM_024450356.1	XM_021849752.1
XM_024450374.1	XM_021842110.1
XM_024450409.1	XM_001661825.2
XM_024450414.1	XR_002499164.1
XM_024450440.1	XM_001656246.2
XM_024450441.1	XM_021838312.1
XM_024450455.1	XM_021852482.1
XM_024450463.1	XM_021854465.1
XM_024450468.1	XM_021837768.1
XM_024450471.1	XM_021849944.1
XM_024450476.1	XM_021854242.1
XM_024450477.1	XM_021840763.1
XM_024450512.1	XM_021851844.1
XM_024450553.1	XR_002500390.1
XM_024450568.1	XM_021851694.1
XM_024450586.1	XR_002499166.1
XM_024450595.1	XM_021837737.1

XM_024450624.1	XM_021844561.1
XM_024450671.1	XM_021856139.1
XM_024450698.1	XM_021846608.1
XM_024450710.1	XM_021853167.1
XM_024450719.1	XM_001653123.2
XM_024450722.1	XM_021840617.1
XM_024450729.1	XM_021840843.1
XM_024450757.1	XM_021838381.1
XM_024450798.1	XM_021849271.1
XM_024450817.1	XM_021857569.1
XM_024450818.1	XM_001648579.2
XM_024450823.1	XM_021841775.1
XM_024450875.1	XM_001661418.2
XM_024450885.1	XM_001660740.2
XM_024450890.1	XM_001653195.2
XM_024450930.1	XM_001660109.2
XM_024450995.1	XM_021840299.1
XM_024451026.1	XM_021847138.1
XM_024451045.1	XM_001662382.2
XM_024451050.1	XM_021837763.1
XM_024451061.1	XM_001658906.2
XM_024451070.1	XM_001659516.2
XM_024451076.1	XM_001652395.2
XM_024451114.1	XM_001651025.2
XM_024451116.1	XM_001662411.2
XM_024451160.1	XM_021837556.1
XM_024451249.1	XM_021846253.1
XM_024451268.1	XM_021847617.1
XM_024451275.1	XM_021840072.1
XM_024451281.1	XM_021853771.1
XM_024451308.1	XM_021837587.1
XM_024451331.1	XM_021855973.1
XM_024451338.1	XM_021840702.1
XM_024451380.1	XM_021841208.1
XM_024451397.1	XM_021853319.1
XM_024451399.1	XM_021845732.1
XM_024451405.1	XM_021856249.1
XM_024451450.1	XM_021838559.1
XM_024451453.1	XM_021854314.1
XM_024451462.1	XR_002499291.1
XM_024451473.1	XM_021849841.1
XM_024451487.1	XM_021843180.1
XM_024451499.1	XR_002498895.1
XM_024451527.1	XM_021839568.1
XM_024451531.1	XM_001661775.2
XM_024451533.1	XM_021854892.1
XM_024451534.1	XM_001656763.2
XM_024451558.1	XM_001657877.3
XM_024451575.1	XM_021854066.1
XM_024451600.1	XM_001651551.2

XM_024451622.1	XM_001652052.2
XM_024451625.1	XM_001649718.2
XM_024451629.1	XM_021838210.1
XM_024451647.1	XR_002499722.1
XM_024451726.1	XM_001661707.2
XM_024451732.1	XM_021849196.1
XM_024451734.1	XM_001647968.2
XM_024451764.1	XM_021847307.1
XM_024451770.1	XM_021842981.1
XM_024451774.1	XM_021840596.1
XM_024451779.1	XM_021846114.1
XM_024451806.1	XM_021852462.1
XM_024451824.1	XM_001653747.3
XM_024451827.1	XM_021849248.1
XM_024451909.1	XM_001654308.2
XM_024451916.1	XR_002501914.1
XM_024451922.1	XM_021846798.1
XM_024451956.1	XM_001658084.2
XM_024451961.1	XM_001658246.2
XM_024451974.1	XM_021842028.1
XM_024451994.1	XM_021850137.1
XM_024452005.1	XM_021837385.1
XM_024452032.1	XR_002501925.1
XM_024452037.1	XM_021849537.1
XM_024452062.1	XM_021837643.1
XM_024452067.1	XM_001652466.2
XM_024452081.1	XM_021856756.1
XM_024452091.1	XM_021847063.1
XM_024452131.1	XM_021850993.1
XM_024452132.1	XM_021848024.1
XM_024452133.1	XM_021837789.1
XM_024452180.1	XM_021837417.1
XM_024452231.1	XM_021842176.1
XM_024452232.1	XM_021856570.1
XM_024452268.1	XM_021855940.1
XM_024452292.1	XM_021839139.1
XM_024452296.1	XM_021854131.1
XM_024452311.1	XM_021848727.1
XM_024452349.1	XM_001654166.2
XM_024452370.1	XM_001648857.2
XM_024452372.1	XM_021842172.1
XM_024452376.1	XM_001659500.2
XM_024452391.1	XM_021842143.1
XM_024452439.1	XM_021848715.1
XM_024452448.1	XM_021837458.1
XM_024452465.1	XM_021853638.1
XM_024452470.1	XM_021855956.1
XM_024452483.1	XM_021841211.1
XM_024452494.1	XR_002502616.1
XM_024452531.1	XR_002500458.1

XM_024452578.1	XR_002503109.1
XM_024452662.1	XM_001648856.2
XM_024452683.1	XM_021849194.1
XM_024452703.1	XR_002499223.1
XM_024452713.1	XM_021848714.1
XM_024452715.1	XM_021842322.1
XM_024452723.1	XM_021848944.1
XM_024452733.1	XM_021840648.1
XM_024452780.1	XM_001648844.2
XM_024452796.1	XM_021849923.1
XM_024452806.1	XM_021854449.1
XM_024452807.1	XM_021853849.1
XM_024452824.1	XM_021844642.1
XM_024452832.1	XM_021837645.1
XM_024452866.1	XM_021849707.1
XM_024452898.1	XR_002501910.1
XM_024452926.1	XM_001656108.2
XM_024452942.1	XM_021852421.1
XM_024452965.1	XR_002502002.1
XM_024453032.1	XM_021840677.1
XM_024453050.1	XM_021838294.1
XM_024453052.1	XM_021853323.1
XM_024453056.1	XM_001660756.2
XM_024453080.1	XM_021839118.1
XM_024453089.1	XR_002499681.1
XM_024453106.1	XM_021847065.1
XM_024453109.1	XR_002499308.1
XM_024453160.1	XM_021848177.1
XM_024453184.1	XM_001647616.2
XM_024453193.1	XM_021857708.1
XM_024453196.1	XR_002499561.1
XM_024453213.1	XM_021854036.1
XM_024453257.1	XR_002501350.1
XM_024453265.1	XM_021854371.1
XM_024453303.1	XM_001650479.2
XM_024453308.1	XR_002500451.1
XM_024453316.1	XM_001649602.3
XM_024453319.1	XM_021851020.1
XM_024453342.1	XM_021851344.1
XM_024453365.1	XM_021853442.1
XM_024453380.1	XM_021850445.1
XM_024453396.1	XM_021855961.1
XM_024453429.1	XM_001654759.2
XM_024453437.1	XM_001661200.3
XM_024453438.1	XM_021837494.1
XM_024453445.1	XR_002502167.1
XM_024453451.1	XM_021839165.1
XM_024453460.1	XM_021851850.1
XM_024453471.1	XM_021841020.1
XM_024453490.1	XM_021848712.1

XM_024453491.1	XR_002501575.1
XM_024453532.1	XM_021849965.1
XM_024453556.1	XM_021856698.1
XM_024453558.1	XM_021843133.1
XM_024453563.1	XR_002502917.1
XM_024453571.1	XM_021841023.1
XM_024453628.1	XM_021841271.1
XM_024453654.1	XM_021846081.1
XM_024453656.1	XM_021848592.1
XM_024453659.1	XM_021847887.1
XM_024453667.1	XM_021843532.1
XM_024453672.1	XM_001648324.2
XM_024453676.1	XM_021838279.1
XM_024453682.1	XM_021842158.1
XM_024453694.1	XM_021852793.1
XM_024453708.1	XM_001662393.2
XM_024453737.1	XM_021845126.1
XM_024453747.1	XM_021847888.1
XM_024453767.1	XM_001654157.2
XM_024453775.1	XR_002501346.1
XM_024453783.1	XM_021851524.1
XM_024453793.1	XM_021842215.1
XM_024453796.1	XM_001658334.2
XM_024453838.1	XM_001661778.2
XM_024453839.1	XR_002501457.1
XM_024453908.1	XM_001649171.2
XM_024453911.1	XR_002502836.1
XM_024453945.1	XM_021856939.1
XM_024454028.1	XM_001655122.2
XM_024454036.1	XM_021846803.1
XM_024454069.1	XM_021852730.1
XM_024454118.1	XM_021849205.1
XM_024454142.1	XM_021855528.1
XM_024454166.1	XM_021853820.1
XM_024454167.1	XM_021855174.1
XM_024454170.1	XM_021843065.1
XM_024454186.1	XM_001647924.3
XM_024454206.1	XM_001662645.2
XM_024454207.1	XM_021855027.1
XM_024454208.1	XM_021851281.1
XM_024454218.1	XM_021855966.1
XM_024454227.1	XM_001653950.2
XM_024454246.1	XM_021838482.1
XM_024454251.1	XM_001651793.2
XM_024454267.1	XM_001659328.3
XM_024454269.1	XM_021842483.1
XM_024454277.1	XM_021845426.1
XM_024454319.1	XM_021855607.1
XM_024454328.1	XM_021855203.1
XM_024454368.1	XM_021857136.1

XM_024454390.1	XM_021838480.1
XM_037916392.1	XM_021840699.1
XR_001736906.2	XM_001655205.2
XR_001736922.2	XM_021842008.1
XR_001736973.2	XM_001653836.2
XR_001736998.1	XM_021857023.1
XR_001737015.1	XM_021841134.1
XR_001737023.2	XM_021844867.1
XR_001737032.2	XM_021854580.1
XR_001737079.2	XM_021851518.1
XR_001737080.1	XM_021846689.1
XR_001737092.1	XM_021844000.1
XR_001737094.2	XM_021843130.1
XR_001737098.1	XM_021837479.1
XR_001737115.1	XM_001658740.2
XR_001737133.1	XM_021850446.1
XR_001737134.2	XM_001652485.2
XR_001737137.2	XM_021848075.1
XR_001737144.1	XM_001654205.2
XR_001737165.2	XM_001651767.2
XR_001737223.2	XR_002500885.1
XR_001737237.1	XR_002499031.1
XR_001737277.1	XM_021842698.1
XR_001737310.2	XM_021838617.1
XR_001737317.2	XM_021854361.1
XR_001737323.1	XM_021843734.1
XR_001737330.1	XM_001655383.3
XR_001737357.2	XM_021845232.1
XR_001737361.2	XM_001653284.2
XR_001737461.1	XM_001663957.2
XR_001737462.1	XM_001659455.2
XR_001737482.2	XM_021853091.1
XR_001737485.2	XM_021850471.1
XR_001737511.1	XM_021850390.1
XR_001737517.2	XM_021855701.1
XR_001737544.1	XM_021837659.1
XR_001737546.1	XM_021839874.1
XR_001737579.2	XM_021841210.1
XR_001737582.2	XM_021841360.1
XR_001737611.1	XM_001656178.2
XR_001737624.1	XR_002502786.1
XR_001737631.1	XM_021837412.1
XR_001737632.2	XM_001654758.2
XR_001737668.1	XM_001655067.2
XR_001737683.1	XM_021841021.1
XR_001737705.1	XM_021847126.1
XR_001737710.1	XM_021848462.1
XR_001737725.2	XM_021854614.1
XR_001737732.2	XM_021855858.1
XR_001737771.1	XM_021855605.1

XR_001737773.1	XR_002500950.1
XR_001737798.1	XM_021841883.1
XR_001737813.1	XR_002501693.1
XR_001737822.1	XM_001652777.2
XR_001737842.1	XM_001662307.2
XR_001737876.1	XM_021837247.1
XR_001737880.1	XM_021845248.1
XR_001737887.1	XM_001661517.2
XR_001737901.1	XM_001653028.2
XR_001737940.1	XM_021841539.1
XR_001737962.1	XM_021840697.1
XR_001737965.1	XM_021849204.1
XR_001738037.2	XM_001656297.2
XR_001738063.1	XM_021856496.1
XR_001738081.1	XM_021838830.1
XR_001738098.1	XM_021856177.1
XR_001738100.1	XR_002499210.1
XR_001738128.1	XM_021846408.1
XR_001738151.2	XR_002502791.1
XR_001738163.1	XM_021855177.1
XR_001738193.1	XR_002500912.1
XR_001738199.1	XM_021856284.1
XR_001738207.1	XM_021840000.1
XR_001738270.1	XM_001653590.2
XR_001738277.2	XM_001650564.2
XR_001738296.1	XM_021837591.1
XR_001738356.1	XM_021844789.1
XR_001738364.1	XM_021844927.1
XR_001738366.1	XM_021847177.1
XR_001738395.1	XR_002500810.1
XR_001738411.1	XM_001658487.2
XR_001738415.1	XM_021846113.1
XR_001738468.1	XM_021849202.1
XR_001738472.1	XM_021848630.1
XR_001738477.1	XM_001662406.2
XR_001738488.1	XM_021840041.1
XR_001738517.1	XR_002500811.1
XR_001738553.1	XR_002501418.1
XR_001738626.2	XM_021840274.1
XR_001738633.1	XM_021857578.1
XR_001738636.2	XM_021842440.1
XR_001738648.1	XM_001659361.2
XR_001738679.1	XM_021848104.1
XR_001738688.2	XM_021847840.1
XR_001738697.1	XM_021846115.1
XR_001738723.1	XR_002502213.1
XR_001738738.1	XR_002502755.1
XR_001738748.1	XM_001648793.2
XR_001738764.1	XM_021840001.1
XR_001738822.1	XM_021841887.1

XR_001738850.1	XM_001660691.2
XR_001738864.2	XR_002500996.1
XR_001738871.1	XR_002498727.1
XR_001738899.2	XM_001653587.2
XR_001738927.1	XR_002502843.1
XR_001738961.1	XM_021854581.1
XR_001738970.1	XM_001651415.2
XR_001738979.1	XM_001650213.2
XR_001738989.2	XM_001651263.2
XR_001739020.1	XR_002502109.1
XR_001739021.1	XM_021852746.1
XR_001739055.2	XM_021847024.1
XR_001739066.1	XM_021848103.1
XR_001739068.1	XM_021843511.1
XR_001739090.1	XM_001650802.2
XR_001739093.1	XM_021838088.1
XR_001739109.1	XM_021849708.1
XR_001739117.1	XM_001654416.2
XR_001739134.1	XM_001661841.3
XR_001739169.1	XM_001652830.2
XR_001739177.1	XM_001650117.2
XR_001739197.1	XR_002500710.1
XR_001739201.1	XM_021842701.1
XR_001739250.1	XM_021842011.1
XR_001739266.2	XM_021852534.1
XR_001739285.1	XM_021840935.1
XR_001739298.1	XM_021852803.1
XR_001739304.2	XM_001649480.2
XR_001739354.1	XM_001658178.2
XR_001739359.1	XM_001651081.2
XR_001739377.1	XM_021852110.1
XR_001739414.2	XM_021837675.1
XR_001739418.2	XM_001660153.2
XR_001739422.1	XM_021848760.1
XR_001739445.1	XM_021853322.1
XR_001739496.1	XM_011494668.2
XR_001739519.1	XM_021850575.1
XR_001739530.1	XM_021849090.1
XR_001739556.1	XM_001663923.2
XR_001739566.1	XM_021855611.1
XR_001739576.1	XM_021850709.1
XR_001739636.1	XM_001650557.2
XR_001739660.1	XM_021844965.1
XR_001739664.1	XM_001656257.2
XR_001739695.2	XM_001662001.2
XR_001739716.1	XM_001655482.2
XR_001739755.1	XR_002501934.1
XR_001739778.1	XM_021855990.1
XR_001739815.1	XM_021857313.1
XR_001739823.1	XM_021852111.1

XR_001739846.1	XM_021848968.1
XR_001739852.2	XM_001655054.2
XR_001739861.1	XM_021846732.1
XR_001739873.1	XM_021851024.1
XR_001739889.1	XM_021844696.1
XR_001739906.1	XM_021840074.1
XR_001739923.1	XM_021856270.1
XR_001739979.1	XM_021845424.1
XR_001739996.2	XM_001658557.2
XR_001740001.1	XM_021837465.1
XR_001740047.1	XM_021855360.1
XR_001740091.1	XM_001652331.2
XR_001740131.2	XM_021852741.1
XR_001740138.1	XM_021846101.1
XR_001740145.2	XR_002503113.1
XR_001740149.2	XR_002499890.1
XR_001740164.1	XM_021840250.1
XR_001740175.1	XM_021849888.1
XR_001740199.2	XM_021853324.1
XR_001740200.2	XM_001657415.2
XR_001740209.2	XM_021857327.1
XR_001740219.2	XM_001655620.2
XR_001740232.1	XM_001649022.2
XR_001740250.1	XM_001656353.2
XR_001740253.2	XM_001658614.2
XR_001740257.1	XM_021841541.1
XR_001740301.2	XM_021848465.1
XR_001740353.2	XM_021850425.1
XR_001740396.1	XM_001664252.2
XR_001740412.1	XM_001656773.2
XR_001740415.1	XR_002501003.1
XR_001740436.2	XM_021852494.1
XR_001740438.1	XM_021857026.1
XR_001740449.1	XM_011495358.2
XR_001740465.1	XM_011495055.2
XR_001740467.1	XM_021838668.1
XR_001740471.1	XM_021840381.1
XR_001740490.2	XM_021846461.1
XR_001740500.1	XM_021837597.1
XR_001740524.1	XM_021852207.1
XR_001740547.1	XM_021837510.1
XR_001740554.1	XR_002499424.1
XR_001740575.1	XM_021845528.1
XR_001740599.1	XM_021846397.1
XR_001740600.1	XM_001657105.2
XR_001740605.2	XR_002502480.1
XR_001740609.1	XM_001658500.2
XR_001740621.1	XM_021853869.1
XR_001740622.1	XM_021850632.1
XR_001740634.1	XM_021847129.1

XR_001740740.1	XM_001656940.2
XR_001740741.1	XR_002499532.1
XR_001740748.2	XM_021838787.1
XR_001740760.2	XM_021838646.1
XR_001740761.1	XM_001655133.2
XR_001740807.1	XM_021848205.1
XR_001740819.1	XM_001654940.2
XR_001740824.1	XM_021848164.1
XR_001740850.2	XM_021855718.1
XR_001740939.1	XR_002502987.1
XR_001740980.1	XM_021849885.1
XR_001741071.1	XM_021843742.1
XR_001741091.2	XR_002500706.1
XR_001741131.2	XM_021837251.1
XR_001741136.1	XM_021838301.1
XR_001741146.2	XM_021851672.1
XR_001741185.1	XM_021854459.1
XR_001741218.1	XM_001649227.2
XR_001741272.2	XM_021839865.1
XR_001741277.1	XM_021856697.1
XR_001741285.2	XM_001657281.2
XR_001741289.1	XM_021855997.1
XR_001741311.2	XM_021844157.1
XR_001741374.1	XR_002499137.1
XR_001741421.1	XM_021846066.1
XR_001741425.2	XM_001656915.2
XR_001741432.1	XR_002501046.1
XR_001741437.1	XM_001651884.2
XR_001741438.1	XM_021846462.1
XR_001741444.2	XM_021848988.1
XR_001741503.1	XM_021851203.1
XR_001741530.1	XM_021849971.1
XR_001741616.1	XM_021841272.1
XR_001741634.2	XM_021851734.1
XR_001741656.1	XM_021839158.1
XR_001741689.1	XR_002499889.1
XR_001741693.2	XM_021855596.1
XR_001741694.1	XM_021847603.1
XR_001741697.1	XR_002498761.1
XR_001741704.1	XM_021846612.1
XR_001741729.1	XM_021856130.1
XR_001741733.1	XM_021849529.1
XR_001741735.1	XM_001654328.2
XR_001741738.1	XM_021839167.1
XR_001741745.2	XM_021854599.1
XR_001741796.1	XM_021855421.1
XR_001741816.1	XM_021840407.1
XR_001741823.2	XM_021845880.1
XR_001741861.1	XM_021852681.1
XR_001741884.2	XR_002499103.1

XR_001741885.1	XM_001651085.2
XR_001741907.2	XR_002502578.1
XR_001741914.1	XM_021849485.1
XR_001741920.1	XR_002501595.1
XR_001741929.1	XM_021855937.1
XR_001741933.1	XM_021850981.1
XR_001741943.2	XM_021849337.1
XR_001741949.1	XM_001657587.2
XR_001741972.2	XM_021856321.1
XR_001742013.2	XM_021853939.1
XR_001742017.2	XR_002499876.1
XR_001742018.1	XM_021840700.1
XR_001742021.1	XM_001657816.2
XR_001742039.1	XM_021856609.1
XR_001742067.2	XM_001652590.2
XR_001742172.1	XM_021847881.1
XR_001742177.1	XM_001654863.2
XR_001742212.1	XM_021855131.1
XR_001742346.1	XM_021843080.1
XR_001742390.2	XM_001655921.2
XR_001742407.1	XM_021857045.1
XR_001742420.2	XM_001648242.2
XR_001742426.1	XM_001648865.2
XR_001742431.1	XM_021846085.1
XR_001742448.1	XM_021846313.1
XR_001742489.1	XM_001649280.2
XR_001742523.1	XR_002501067.1
XR_001742562.1	XM_021838665.1
XR_001742570.1	XM_001653617.2
XR_001742578.1	XM_021856796.1
XR_001742597.1	XM_001663732.2
XR_001742607.1	XM_021854593.1
XR_001742616.1	XM_001655406.2
XR_001742674.1	XR_002503157.1
XR_001742688.1	XM_021853889.1
XR_001742709.1	XM_001653067.2
XR_001742725.2	XM_021840395.1
XR_001742804.1	XM_021847133.1
XR_001742807.1	XM_001653995.2
XR_001742811.2	XM_021838515.1
XR_001742822.1	XM_001649928.2
XR_001742867.1	XR_002500115.1
XR_001742868.1	XR_002498720.1
XR_001742905.1	XM_001650986.2
XR_001742913.1	XM_021846230.1
XR_001742931.1	XM_021853000.1
XR_001742941.1	XM_021853001.1
XR_001742945.1	XR_002499130.1
XR_001742960.1	XM_001664089.2
XR_001742987.1	XM_021847073.1

XR_001742990.1	XM_021845657.1
XR_001743034.1	XM_021838074.1
XR_001743048.1	XM_021854245.1
XR_001743180.2	XM_021856071.1
XR_001743200.1	XM_021844926.1
XR_001743223.2	XM_021841827.1
XR_001743233.2	XM_021849840.1
XR_001743243.1	XM_021847594.1
XR_001743262.2	XM_021844915.1
XR_001743302.1	XM_021844618.1
XR_001743308.2	XM_021855145.1
XR_001743453.1	XM_021854841.1
XR_001743487.2	XR_002498632.1
XR_001743504.1	XR_002499274.1
XR_001743623.2	XM_021839295.1
XR_001743626.2	XM_001658271.2
XR_001743696.2	XR_002503120.1
XR_001743702.1	XM_001653720.2
XR_001743720.1	XM_021854442.1
XR_001743723.1	XM_021849804.1
XR_001743726.2	XM_021857631.1
XR_001743790.1	XR_002498824.1
XR_001743815.1	XM_021846459.1
XR_001743903.1	XM_021839103.1
XR_001743911.2	XM_001657699.2
XR_001743912.2	XM_021841911.1
XR_001743913.1	XR_002498584.1
XR_001743918.1	XM_001663070.2
XR_001743956.1	XM_021844916.1
XR_001743981.1	XM_021845422.1
XR_001743990.1	XR_002502919.1
XR_001744027.1	XM_001658879.2
XR_001744091.1	XM_021852559.1
XR_001744099.1	XM_001653305.2
XR_001744157.1	XM_021849288.1
XR_001744160.1	XR_002501101.1
XR_001744190.1	XM_001648078.3
XR_001744216.2	XM_021854551.1
XR_001744257.1	XM_021846918.1
XR_001744333.1	XM_021844920.1
XR_001744360.2	XM_021851517.1
XR_001744374.1	XM_021854489.1
XR_001744391.1	XM_001663754.2
XR_001744393.1	XM_021841876.1
XR_001744397.2	XM_021838692.1
XR_001744408.1	XM_021838815.1
XR_001744418.1	XR_002500460.1
XR_001744431.1	XM_021837453.1
XR_001744446.1	XM_001659468.2
XR_001744482.1	XM_001653619.2

XR_001744514.1	XM_021854639.1
XR_001744601.2	XR_002499676.1
XR_001744694.1	XM_021842749.1
XR_001744731.2	XM_021856619.1
XR_001744750.1	XM_001661607.2
XR_001744811.1	XM_021852634.1
XR_001744812.1	XM_021837524.1
XR_001744823.1	XM_021853776.1
XR_001744840.1	XM_021839166.1
XR_001744887.2	XR_002501263.1
XR_001744889.2	XM_001664080.2
XR_001744898.2	XM_021846456.1
XR_001744903.2	XM_021838502.1
XR_001744938.1	XM_001653057.2
XR_001744943.2	XM_021851883.1
XR_001745010.1	XM_021842262.1
XR_001745019.1	XM_021851882.1
XR_001745068.1	XM_021837403.1
XR_001745070.2	XM_021854842.1
XR_001745074.1	XM_021847598.1
XR_001745083.2	XM_021855242.1
XR_001745090.1	XM_021843594.1
XR_001745096.1	XR_002499891.1
XR_001745183.1	XM_021838664.1
XR_001745186.1	XM_021840514.1
XR_001745257.1	XR_002499102.1
XR_001745273.1	XR_002502033.1
XR_001745332.1	XM_021837681.1
XR_001745352.1	XM_021849522.1
XR_001745364.1	XM_001651043.2
XR_001745380.1	XM_001663668.3
XR_001745384.1	XM_021847644.1
XR_001745422.1	XM_021847345.1
XR_001745496.1	XR_002502808.1
XR_001745506.2	XM_021838820.1
XR_001745508.2	XM_001661622.2
XR_001745533.1	XM_021838349.1
XR_001745554.2	XM_021854281.1
XR_001745582.1	XM_001657219.2
XR_001745593.1	XM_021849605.1
XR_001745611.2	XM_021856608.1
XR_001745613.2	XM_001662007.2
XR_001745629.2	XM_001657181.2
XR_001745651.2	XM_001654198.2
XR_001745657.1	XM_001657837.2
XR_001745684.1	XM_021838434.1
XR_001745705.1	XM_021856968.1
XR_001745709.1	XM_021849878.1
XR_001745769.1	XM_021849706.1
XR_001745779.1	XR_002501251.1

XR_001745885.1	XM_021857099.1
XR_001745914.1	XM_001653056.2
XR_001745965.1	XM_021837715.1
XR_001745973.1	XR_002500503.1
XR_001746017.1	XM_021845167.1
XR_001746060.1	XM_021852720.1
XR_001746092.2	XM_021842007.1
XR_001746128.1	XM_001658518.2
XR_001746129.1	XM_021853426.1
XR_001746150.2	XM_021844029.1
XR_001746162.2	XM_001649859.2
XR_001746166.2	XM_021856879.1
XR_001746272.1	XM_021846237.1
XR_001746310.2	XM_021855610.1
XR_001746329.2	XM_021838957.1
XR_001746345.1	XM_021852871.1
XR_001746417.2	XM_021838549.1
XR_001746431.1	XR_002502560.1
XR_001746459.1	XM_021844963.1
XR_001746478.2	XM_021840631.1
XR_001746499.1	XM_021837641.1
XR_001746540.2	XR_002502292.1
XR_001746542.2	XM_021851322.1
XR_001746556.2	XM_021849735.1
XR_001746565.1	XM_021855343.1
XR_001746574.1	XR_002502432.1
XR_001746577.1	XR_002501324.1
XR_001746613.2	XM_021838081.1
XR_001746656.1	XR_002502639.1
XR_001746667.1	XM_021840307.1
XR_001746681.1	XR_002502677.1
XR_001746684.2	XM_001653729.2
XR_001746690.1	XM_021844922.1
XR_001746718.1	XM_021838538.1
XR_001746727.1	XM_021837761.1
XR_001746756.1	XM_021840633.1
XR_001746760.1	XM_001661354.2
XR_001746839.1	XM_001659222.2
XR_001746844.1	XM_021857618.1
XR_001746887.1	XM_001651280.3
XR_001746907.1	XM_021846685.1
XR_001746914.1	XM_001655423.2
XR_001746931.1	XM_001653857.2
XR_001746932.1	XM_021848901.1
XR_001746934.1	XM_021847601.1
XR_001746963.1	XM_001661839.2
XR_001746986.1	XM_021857574.1
XR_001747015.1	XM_021853384.1
XR_001747016.1	XM_001657776.2
XR_001747096.1	XM_021845554.1

XR_001747123.2	XM_021838289.1
XR_001747173.1	XM_021845709.1
XR_001747197.2	XM_021845660.1
XR_001747218.1	XM_021855405.1
XR_001747221.1	XM_001648407.2
XR_001747223.1	XM_021839321.1
XR_001747227.1	XM_021845359.1
XR_001747229.1	XM_021837722.1
XR_001747250.1	XM_021839749.1
XR_001747275.1	XM_021838359.1
XR_001747280.1	XR_002499186.1
XR_001747282.1	XM_021845653.1
XR_001747293.1	XM_021841843.1
XR_001747319.2	XR_002502051.1
XR_001747335.1	XR_002501966.1
XR_001747343.1	XM_021846524.1
XR_001747348.1	XM_021840571.1
XR_001747359.1	XM_021849520.1
XR_001747385.2	XM_021839723.1
XR_001747416.1	XM_021840559.1
XR_001747428.1	XM_021843073.1
XR_001747431.1	XM_021849594.1
XR_001747434.1	XM_021849853.1
XR_001747479.1	XM_021837569.1
XR_001747484.2	XM_001648240.2
XR_001747488.2	XM_021839169.1
XR_001747494.1	XM_021843135.1
XR_001747560.2	XM_021840394.1
XR_001747561.1	XM_021837568.1
XR_001747568.1	XM_021855126.1
XR_001747602.1	XM_021840173.1
XR_001747603.1	XM_021844593.1
XR_001747616.1	XM_021851670.1
XR_001747657.1	XM_021855322.1
XR_001747753.1	XR_002502697.1
XR_001747852.1	XR_002501339.1
XR_001747896.1	XM_021847616.1
XR_001747911.2	XR_002498716.1
XR_001747931.1	XM_001653685.2
XR_001747949.2	XR_002499313.1
XR_001747953.2	XM_001650619.2
XR_001748011.2	XM_001649738.2
XR_001748053.2	XM_021842750.1
XR_001748059.1	XR_002499249.1
XR_001748066.1	XM_021851395.1
XR_001748072.1	XR_002498753.1
XR_001748092.1	XM_021855530.1
XR_001748099.2	XM_021846404.1
XR_001748108.2	XM_021848074.1
XR_001748163.1	XM_001648605.2

XR_001748164.1	XM_021855792.1
XR_001748167.1	XM_021837560.1
XR_001748204.2	XM_021842703.1
XR_001748227.1	XM_001651988.2
XR_001748242.1	XM_021840608.1
XR_001748254.1	XM_001652746.2
XR_001748279.1	XM_001652244.2
XR_001748294.1	XM_001651062.2
XR_001748325.1	XM_001659690.2
XR_001748327.1	XM_021838496.1
XR_001748341.1	XM_001648918.2
XR_001748349.1	XR_002502658.1
XR_001748376.1	XR_002502265.1
XR_001748382.1	XM_001662090.2
XR_001748384.1	XM_021842275.1
XR_001748400.1	XM_021841130.1
XR_001748439.1	XM_021842857.1
XR_001748456.1	XM_021837249.1
XR_001748469.1	XM_021841374.1
XR_001748486.1	XM_001661748.2
XR_001748580.2	XM_021849017.1
XR_001748606.2	XM_001649842.2
XR_001748628.1	XR_002499639.1
XR_001748691.1	XM_001649915.2
XR_001748700.2	XM_021845656.1
XR_001748726.2	XM_001658911.2
XR_001748728.1	XM_021851043.1
XR_001748820.1	XR_002498862.1
XR_001748839.1	XR_002502642.1
XR_001748843.1	XR_002498776.1
XR_001748866.2	XM_021841869.1
XR_001748897.2	XM_021848850.1
XR_001748898.2	XM_021842261.1
XR_001748905.2	XM_001656746.2
XR_001748933.2	XM_001649460.2
XR_001748946.1	XM_021857712.1
XR_001749033.1	XR_002500673.1
XR_001749039.1	XM_001661822.2
XR_001749071.1	XM_021854894.1
XR_001749095.1	XM_021847111.1
XR_001749102.1	XM_001655763.2
XR_001749108.1	XR_002502710.1
XR_001749114.1	XM_021837599.1
XR_001749127.2	XM_021840678.1
XR_001749131.1	XR_002498707.1
XR_001749146.1	XM_001655511.2
XR_001749251.1	XM_001658844.2
XR_001749260.1	XM_021837603.1
XR_001749305.1	XM_001656499.2
XR_001749346.2	XM_021851866.1

XR_001749378.1	XR_002499743.1
XR_001749394.1	XR_002501924.1
XR_001749430.1	XM_021840007.1
XR_001749585.2	XM_021846000.1
XR_001749614.2	XM_021845987.1
XR_001749668.2	XM_021839018.1
XR_001749717.2	XM_021838497.1
XR_001749854.1	XM_021843961.1
XR_001749883.1	XM_001650170.2
XR_001749924.1	XM_001655193.2
XR_001749952.1	XM_021840172.1
XR_001749996.1	XM_001653421.2
XR_001750001.1	XM_021852801.1
XR_001750008.1	XM_021851982.1
XR_001750018.1	XM_021844249.1
XR_001750024.1	XM_001663866.2
XR_001750181.2	XM_001656713.2
XR_001750279.1	XM_001650151.2
XR_001750360.1	XM_021843171.1
XR_001750400.1	XM_001657313.2
XR_001750415.1	XM_021845812.1
XR_001750478.2	XM_001658363.2
XR_001750482.1	XM_001664001.2
XR_001750510.2	XM_021852496.1
XR_001750605.1	XM_001657576.3
XR_001750621.1	XM_021838220.1
XR_001750626.1	XM_021846476.1
XR_001750673.1	XM_021852280.1
XR_001750696.1	XM_021841871.1
XR_001750733.1	XM_001657695.2
XR_001750736.1	XM_021852160.1
XR_001750758.1	XM_021851039.1
XR_001750759.1	XM_021853423.1
XR_001750777.1	XR_002499229.1
XR_001750780.1	XM_001652399.2
XR_001750788.2	XM_001648473.2
XR_001750796.1	XM_001649528.2
XR_001750818.2	XR_002499690.1
XR_001750836.2	XM_021839698.1
XR_001750866.1	XM_001661405.2
XR_001750871.2	XR_002501568.1
XR_001750891.1	XM_001656024.2
XR_001750945.1	XM_021838077.1
XR_001750963.1	XM_021849482.1
XR_001751016.1	XM_001657056.2
XR_001751081.1	XM_021848317.1
XR_001751098.2	XM_001651845.2
XR_001751142.1	XR_002499095.1
XR_001751181.1	XM_001660686.2
XR_001751221.2	XM_021837276.1

XR_001751298.2	XR_002501921.1
XR_001751311.1	XM_001653987.2
XR_001751318.1	XM_021837248.1
XR_001751319.2	XM_001650112.2
XR_001751333.2	XR_002503240.1
XR_001751379.2	XM_021855323.1
XR_001751381.1	XM_021849859.1
XR_001751400.1	XM_001658617.2
XR_001751523.1	XM_021843061.1
XR_001751554.2	XM_001662359.2
XR_001751587.1	XR_002499691.1
XR_001751594.1	XM_021855124.1
XR_001751607.1	XM_021856020.1
XR_001751613.2	XM_021841377.1
XR_001751636.1	XM_021840044.1
XR_001751683.1	XR_002502528.1
XR_001751694.1	XM_001652146.2
XR_001751696.1	XM_021847813.1
XR_001751763.2	XM_021839867.1
XR_001751767.2	XM_021845457.1
XR_001751795.1	XM_001663283.2
XR_001751806.1	XM_021849470.1
XR_001751818.2	XM_021853048.1
XR_001751827.2	XM_021851548.1
XR_001751838.1	XM_021848836.1
XR_001751881.2	XM_021851929.1
XR_001751886.1	XM_021841216.1
XR_001751895.1	XM_001654936.2
XR_001751907.2	XR_002501125.1
XR_001751939.1	XM_021837333.1
XR_001751942.1	XR_002502170.1
XR_001751948.1	XM_021845055.1
XR_001751996.1	XM_021838670.1
XR_001752064.1	XM_021838293.1
XR_001752066.1	XM_021845458.1
XR_001752082.1	XR_002501154.1
XR_001752085.2	XR_002502925.1
XR_001752103.1	XM_021841923.1
XR_001752119.2	XM_021838941.1
XR_001752179.1	XM_021845950.1
XR_001752213.1	XM_001653993.2
XR_001752214.1	XM_021840775.1
XR_001752219.1	XM_021843129.1
XR_001752224.1	XM_001655478.2
XR_001752225.1	XM_021839218.1
XR_001752227.1	XM_021855150.1
XR_001752232.1	XM_001652057.2
XR_001752236.1	XM_021849546.1
XR_001752238.1	XM_021841441.1
XR_001752250.1	XM_001662421.2

XR_001752271.2	XM_001653580.2
XR_001752336.1	XM_001663378.2
XR_001752359.1	XM_021840077.1
XR_001752394.2	XM_021851232.1
XR_001752486.2	XM_021845936.1
XR_001752487.1	XM_001661586.2
XR_001752495.1	XM_021854594.1
XR_001752509.2	XM_021856345.1
XR_001752529.1	XM_021853301.1
XR_001752545.1	XM_001653777.2
XR_001752558.1	XM_021857639.1
XR_001752594.1	XM_021838276.1
XR_001752604.2	XM_021839618.1
XR_001752626.2	XM_021849457.1
XR_001752661.2	XM_021854173.1
XR_001752664.1	XM_021853276.1
XR_001752677.2	XM_021846450.1
XR_001752679.1	XM_001652704.2
XR_001752687.2	XM_001654156.2
XR_001752688.2	XR_002502805.1
XR_001752722.2	XM_001659083.3
XR_001752740.1	XM_021838292.1
XR_001752749.1	XM_021838204.1
XR_001752814.2	XM_021854587.1
XR_001752868.1	XM_001651800.2
XR_001752870.1	XM_021845879.1
XR_001752882.2	XR_002502104.1
XR_001752918.2	XM_021851044.1
XR_001752972.2	XM_021841080.1
XR_001752980.2	XM_021842479.1
XR_001752996.1	XM_021857634.1
XR_001753013.2	XM_021844658.1
XR_001753025.1	XM_021847285.1
XR_001753096.1	XM_021854641.1
XR_001753115.2	XM_021854907.1
XR_001753127.1	XM_001657779.2
XR_001753138.1	XM_001648520.2
XR_001753166.1	XM_021846830.1
XR_001753189.1	XM_021851252.1
XR_001753260.2	XM_001660368.2
XR_001753324.1	XM_021856864.1
XR_001753345.1	XM_021841446.1
XR_001753371.1	XR_002500262.1
XR_001753374.1	XM_021838688.1
XR_001753375.1	XM_001658390.2
XR_001753379.2	XM_021848076.1
XR_001753382.1	XM_001659049.2
XR_001753404.1	XM_001663952.2
XR_001753412.1	XM_021853419.1
XR_001753427.1	XR_002502289.1

XR_001753435.1	XR_002501325.1
XR_001753442.1	XR_002499754.1
XR_001753467.1	XM_021850024.1
XR_001753495.1	XM_001664123.2
XR_001753503.1	XM_021849712.1
XR_001753542.1	XM_001658796.2
XR_001753549.1	XM_001653334.2
XR_001753552.1	XM_021856419.1
XR_001753553.1	XM_021851248.1
XR_001753567.2	XR_002501806.1
XR_001753614.2	XM_021842799.1
XR_001753642.1	XM_021854813.1
XR_001753661.1	XM_001652895.2
XR_001753662.1	XM_021839296.1
XR_001753663.2	XM_021843955.1
XR_001753665.2	XM_001654611.2
XR_001753675.1	XM_021856709.1
XR_001753702.1	XR_002500636.1
XR_001753751.1	XM_021838191.1
XR_001753840.1	XM_021839122.1
XR_001753866.1	XM_001650584.2
XR_001753920.1	XM_021848860.1
XR_001753961.1	XM_021854589.1
XR_001754017.1	XR_002500298.1
XR_001754024.1	XM_021846688.1
XR_001754058.1	XR_002501352.1
XR_001754062.1	XM_001647564.2
XR_001754077.1	XM_021855348.1
XR_001754085.1	XM_001652775.2
XR_001754187.1	XM_021855340.1
XR_001754252.1	XM_001659833.2
XR_001754294.1	XR_002501145.1
XR_001754324.1	XR_002502552.1
XR_001754379.1	XM_021840008.1
XR_001754412.1	XM_001656887.2
XR_001754443.2	XM_021838704.1
XR_001754491.1	XR_002500724.1
XR_001754493.1	XM_001648259.2
XR_001754526.1	XR_002501258.1
XR_001754532.1	XM_001653646.2
XR_001754556.2	XM_021850663.1
XR_001754560.1	XM_021852341.1
XR_001754596.1	XM_001649218.2
XR_001754618.1	XM_001654203.2
XR_001754623.1	XM_001652058.2
XR_001754634.2	XM_001663791.2
XR_001754691.1	XM_021838035.1
XR_001754738.1	XM_001660118.2
XR_001754902.2	XM_021856282.1
XR_001754920.2	XM_001662816.2

XR_001754963.2	XM_021851250.1
XR_001754973.1	XM_021851389.1
XR_001754982.1	XM_021840312.1
XR_001754983.1	XM_001652166.2
XR_001754994.1	XM_001648785.2
XR_001754995.1	XM_021854272.1
XR_001755040.1	XR_002501072.1
XR_001755082.2	XM_001655379.2
XR_001755190.1	XM_021839846.1
XR_001755232.1	XM_021844962.1
XR_001755233.1	XM_021841010.1
XR_001755416.2	XM_021854591.1
XR_001755441.1	XM_001658930.2
XR_001755448.1	XM_021846841.1
XR_001755452.1	XM_021843422.1
XR_001755457.1	XR_002501890.1
XR_001755490.1	XM_021853766.1
XR_001755513.1	XM_021838314.1
XR_001755552.2	XM_021847431.1
XR_001755623.1	XM_021843184.1
XR_001755679.1	XM_001656397.2
XR_001755707.2	XM_001647970.2
XR_001755714.1	XR_002499765.1
XR_001755718.2	XM_021852356.1
XR_001755743.1	XM_001662860.2
XR_001755765.1	XM_021848391.1
XR_001755783.1	XR_002500543.1
XR_001755784.1	XM_021837716.1
XR_001755790.1	XM_001654927.2
XR_001755798.1	XM_001649552.2
XR_001755858.1	XM_021851921.1
XR_001755861.2	XM_001663674.2
XR_001755870.1	XM_021855274.1
XR_001755898.1	XM_021849685.1
XR_001755913.1	XM_021847057.1
XR_001755922.1	XR_002503165.1
XR_001755939.1	XM_021856389.1
XR_001755967.1	XR_002501045.1
XR_001755969.1	XM_021843066.1
XR_001756037.1	XM_021851689.1
XR_001756056.1	XM_021841603.1
XR_001756065.1	XR_002500691.1
XR_001756077.1	XM_021855871.1
XR_001756087.2	XM_021852641.1
XR_001756115.1	XR_002499688.1
XR_001756128.1	XR_002499519.1
XR_001756135.1	XM_021854586.1
XR_001756158.2	XM_001653835.2
XR_001756170.1	XM_021847058.1
XR_001756188.1	XM_001662525.2

XR_001756224.1	XM_021848079.1
XR_001756227.1	XM_021851393.1
XR_001756308.1	XM_021842082.1
XR_001756322.1	XM_021851919.1
XR_001756339.1	XR_002502509.1
XR_001756347.1	XM_021838076.1
XR_001756355.1	XM_021853278.1
XR_001756371.1	XM_001662111.2
XR_001756382.1	XM_001650810.2
XR_001756402.1	XR_002500995.1
XR_001756421.2	XM_001656052.2
XR_001756422.2	XR_002499272.1
XR_001756455.1	XM_021849262.1
XR_001756475.1	XR_002498786.1
XR_001756486.1	XR_002501643.1
XR_001756499.1	XM_021842654.1
XR_001756508.1	XM_001650795.2
XR_001756514.1	XM_001660548.2
XR_001756529.2	XR_002502082.1
XR_001756547.2	XR_002498947.1
XR_001756560.1	XM_021841995.1
XR_001756600.2	XM_001648849.2
XR_001756614.1	XM_001654721.2
XR_001756617.1	XM_021845553.1
XR_001756628.1	XM_021852467.1
XR_001756639.1	XM_021845529.1
XR_001756649.1	XR_002498734.1
XR_001756671.1	XM_021841957.1
XR_001756770.1	XM_021844237.1
XR_001756782.2	XM_021853280.1
XR_001756882.1	XM_021856319.1
XR_001756883.1	XM_021843998.1
XR_001756888.2	XM_021840033.1
XR_001756895.1	XM_001660603.2
XR_001756897.1	XM_021846821.1
XR_001756940.1	XM_021855349.1
XR_001756948.2	XM_001652105.2
XR_002956186.1	XM_001663505.2
XR_002956193.1	XR_002501540.1
XR_002956238.1	XM_021853277.1
XR_002956299.1	XR_002499282.1
XR_002956316.1	XR_002501127.1
XR_002956323.1	XM_021850438.1
XR_002956327.1	XM_021855551.1
XR_002956329.1	XM_021853846.1
XR_002956330.1	XM_001648602.2
XR_002956338.1	XM_021840030.1
XR_002956359.1	XM_001648550.2
XR_002956360.1	XM_001662491.2
XR_002956377.1	XM_021838588.1

XR_002956385.1	XM_001653222.2
XR_002956402.1	XM_001651521.2
XR_002956419.1	XR_002499040.1
XR_002956438.1	XM_021857575.1
XR_002956472.1	XR_002499584.1
XR_002956484.1	XR_002502962.1
XR_002956516.1	XR_002500919.1
XR_002956522.1	XM_001655733.2
XR_002956528.1	XM_001660347.2
XR_002956556.1	XM_021839219.1
XR_002956570.1	XR_002500808.1
XR_002956581.1	XM_021838465.1
XR_002956587.1	XM_021852166.1
XR_002956616.1	XM_021849421.1
XR_002956645.1	XM_001651177.2
XR_002956648.1	XM_021856055.1
XR_002956664.1	XR_002501390.1
XR_002956710.1	XR_002499721.1
XR_002956719.1	XM_001650281.2
XR_002956749.1	XM_021850844.1
XR_002956759.1	XM_021839533.1
XR_002956775.1	XM_001654282.2
XR_002956808.1	XM_021846614.1
XR_002956817.1	XM_011494789.2
XR_002956845.1	XM_011495041.2
XR_002956869.1	XM_021855920.1
XR_002956908.1	XR_002500725.1
XR_002956924.1	XM_011494996.2
XR_002956954.1	XM_021837422.1
XR_002956983.1	XM_001657428.2
XR_002957021.1	XM_001651388.2
XR_002957043.1	XR_002499127.1
XR_002957106.1	XM_021846903.1
XR_002957116.1	XM_021841771.1
XR_002957174.1	XM_021838487.1
XR_002957188.1	XM_021849419.1
XR_002957190.1	XM_001654857.2
XR_002957204.1	XM_021857409.1
XR_002957228.1	XR_002503176.1
XR_002957229.1	XR_002501522.1
XR_002957230.1	XM_021846901.1
XR_002957272.1	XM_021846717.1
XR_002957281.1	XM_001659513.2
XR_002957294.1	XM_001653636.3
XR_002957308.1	XM_021856981.1
XR_002957310.1	XM_001652993.3
XR_002957337.1	XM_001652133.2
XR_002957340.1	XM_021856567.1
XR_002957350.1	XM_021851083.1
XR_002957360.1	XR_002498782.1

XR_002957363.1	XM_021857404.1
XR_002957384.1	XM_021842016.1
XR_002957422.1	XM_021845530.1
XR_002957474.1	XR_002501179.1
XR_002957476.1	XM_001658393.2
XR_002957480.1	XM_021856913.1
XR_002957486.1	XM_021840615.1
XR_002957489.1	XM_021850821.1
XR_002957490.1	XM_021850910.1
XR_002957496.1	XM_021846843.1
XR_002957501.1	XM_021845871.1
XR_002957533.1	XM_021839132.1
XR_002957537.1	XM_001651270.2
XR_002957577.1	XM_001655472.2
XR_002957578.1	XM_021856874.1
XR_002957582.1	XM_021847729.1
XR_002957618.1	XM_001648783.2
XR_002957624.1	XM_001661996.2
XR_002957634.1	XM_021848080.1
XR_002957679.1	XM_001655909.2
XR_002957691.1	XM_021841396.1
XR_002957706.1	XM_001664212.2
XR_002957746.1	XM_021856088.1
XR_002957752.1	XM_001662964.2
XR_002957809.1	XM_021854695.1
XR_002957853.1	XM_021841956.1
XR_002957873.1	XR_002500147.1
XR_002957881.1	XM_001660006.2
XR_002957885.1	XR_002501177.1
XR_002957897.1	XM_001657585.2
XR_002957901.1	XR_002500213.1
XR_002957911.1	XM_021841972.1
XR_002957945.1	XM_021841158.1
XR_002957961.1	XM_001652048.2
XR_002958000.1	XM_021857408.1
XR_002958039.1	XM_001654870.2
XR_002958047.1	XM_021848057.1
XR_002958065.1	XM_001654599.2
XR_002958071.1	XM_021841716.1
XR_002958079.1	XR_002499096.1
XR_002958082.1	XM_021847021.1
XR_002958088.1	XM_021846120.1
XR_002958091.1	XR_002499316.1
XR_002958096.1	XM_001663071.2
XR_002958104.1	XM_021856370.1
XR_002958114.1	XM_001657642.2
XR_002958116.1	XM_001659905.2
XR_002958175.1	XM_021857434.1
XR_002958176.1	XM_001651862.2
XR_002958182.1	XM_001647791.2

XR_002958204.1	XR_002498981.1
XR_002958206.1	XM_021838020.1
XR_002958207.1	XM_021854873.1
XR_002958235.1	XM_021845404.1
XR_002958244.1	XM_021844503.1
XR_002958250.1	XM_021845263.1
XR_002958252.1	XM_021841393.1
XR_002958257.1	XM_021855163.1
XR_002958266.1	XM_001653633.3
XR_002958282.1	XM_001656339.2
XR_002958330.1	XM_021839649.1
XR_002958342.1	XM_021857253.1
XR_002958369.1	XR_002501651.1
XR_002958373.1	XR_002501828.1
XR_002958387.1	XM_001653915.2
XR_002958388.1	XM_021850511.1
XR_002958393.1	XM_021839787.1
XR_002958414.1	XM_001653989.2
XR_002958418.1	XM_001653884.2
XR_002958423.1	XM_021851690.1
XR_002958460.1	XM_001651993.2
XR_002958481.1	XM_001660592.2
XR_002958482.1	XM_021855083.1
XR_002958489.1	XM_021841936.1
XR_002958504.1	XR_002501364.1
XR_002958524.1	XM_021849150.1
XR_002958557.1	XM_001653765.2
XR_002958565.1	XM_021852894.1
XR_002958575.1	XM_021848198.1
XR_002958601.1	XM_021840088.1
XR_002958616.1	XM_021846015.1
XR_002958625.1	XM_001649482.2
XR_002958639.1	XM_021837328.1
XR_002958666.1	XM_021853132.1
XR_002958698.1	XM_001656531.2
XR_002958716.1	XM_021846315.1
XR_002958720.1	XM_021846139.1
XR_002958749.1	XM_021838278.1
XR_002958760.1	XR_002499504.1
XR_002958774.1	XM_021849051.1
XR_002958806.1	XR_002502818.1
XR_002958821.1	XM_021841994.1
XR_002958830.1	XM_021843222.1
XR_002958865.1	XM_021852638.1
XR_002958872.1	XR_002500583.1
XR_002958884.1	XR_002503206.1
XR_002958894.1	XM_021846138.1
XR_002958898.1	XM_021848338.1
XR_002958922.1	XM_001661763.2
XR_002958941.1	XM_021851733.1

XR_002958976.1	XM_021841442.1
XR_002959024.1	XM_021845139.1
XR_002959052.1	XM_001653086.2
XR_002959055.1	XM_001649092.2
XR_002959065.1	XM_021847023.1
XR_002959099.1	XM_021854539.1
XR_002959104.1	XR_002501946.1
XR_002959124.1	XM_021853958.1
XR_002959134.1	XM_021852163.1
XR_002959142.1	XR_002499365.1
XR_002959146.1	XM_021856195.1
XR_002959170.1	XM_021853957.1
XR_002959184.1	XM_001651272.2
XR_002959197.1	XM_021847053.1
XR_002959213.1	XR_002501959.1
XR_002959292.1	XM_001661123.2
XR_002959317.1	XR_002500529.1
XR_002959329.1	XM_021852691.1
XR_002959342.1	XM_021852640.1
XR_002959350.1	XR_002502973.1
XR_002959355.1	XR_002502141.1
XR_002959363.1	XM_001656806.2
XR_002959364.1	XM_021840773.1
XR_002959368.1	XM_001648257.2
XR_002959392.1	XM_021854156.1
XR_002959403.1	XR_002501298.1
XR_002959407.1	XM_001663995.2
XR_002959421.1	XM_021847901.1
XR_002959424.1	XM_001655167.2
XR_002959425.1	XM_001659854.2
XR_002959439.1	XM_021840512.1
XR_002959446.1	XM_021845834.1
XR_002959451.1	XM_001661610.2
XR_002959471.1	XM_001662036.3
XR_002959489.1	XR_002498839.1
XR_002959503.1	XM_021851379.1
XR_002959510.1	XR_002501961.1
XR_002959518.1	XM_021844635.1
XR_002959528.1	XM_001649611.2
XR_002959568.1	XM_021850032.1
XR_002959592.1	XM_021850289.1
XR_002959605.1	XM_021850033.1
XR_002959606.1	XM_021839790.1
XR_002959616.1	XM_021857620.1
XR_002959656.1	XM_021841800.1
XR_002959782.1	XM_021842000.1
XR_002959813.1	XM_001660247.2
XR_002959816.1	XM_021855345.1
XR_002959821.1	XM_021849362.1
XR_002959824.1	XR_002501880.1

XR_004643303.1	XM_001657420.2
XR_004837503.1	XM_021841740.1
XR_004837528.1	XM_001649340.2
XR_004837530.1	XR_002501382.1
XR_004837531.1	XM_001657174.2
XR_005228010.1	XM_001649821.2
XR_005228025.1	XM_021839459.1
XR_158942.3	XM_001649696.2
XR_171071.2	XM_001649289.2
XR_241302.2	XM_021838370.1
XR_241325.1	XR_002498770.1
XR_241866.3	XM_001656916.2
XR_241885.2	XM_021842251.1
XR_242213.1	XR_002499527.1
XR_242682.4	XM_021857621.1
XR_242777.3	XR_002501186.1
XR_242877.4	XM_021844228.1
XR_242895.4	XM_001656164.2
XR_243258.3	XM_021849809.1
XR_243305.3	XM_001657961.3
XR_243433.3	XM_021852694.1
XR_243875.3	XR_002499011.1
XR_244323.3	XM_021837349.1
XR_244356.3	XM_021839271.1
XR_244468.4	XM_021851916.1
XR_244887.5	XM_021854601.1
XR_244972.3	XM_021851189.1
XR_245020.3	XM_021838382.1
XR_245100.1	XM_001660426.2
XR_245182.3	XM_001661618.2
XR_245208.3	XM_021841932.1
XR_245442.5	XM_001655564.2
XR_245604.3	XM_001652020.2
XR_245695.2	XM_001660270.2
XR_245708.4	XM_021840311.1
XR_245788.2	XM_001653769.2
XR_246111.5	XM_011494982.2
XR_246197.2	XM_001662377.2
XR_246305.4	XM_001663852.2
XR_426708.3	XM_021841567.1
XR_426737.3	XM_021847160.1
XR_426846.4	XM_021842683.1
XR_426950.2	XM_001655743.2
XR_427017.3	XR_002499292.1
XR_427085.3	XM_001663394.2
XR_427149.4	XM_021842006.1
XR_427503.4	XR_002499604.1
XR_427775.3	XM_021837284.1
XR_427805.2	XM_001662581.2
XR_427811.3	XM_021837767.1

XR_427883.4	XM_021842692.1
XR_427919.3	XM_021852695.1
XR_428154.3	XM_021851375.1
XR_428319.2	XM_021854530.1
XR_428359.3	XR_002501471.1
XR_428520.3	XR_002501174.1
XR_428583.3	XM_021852693.1
XR_428650.1	XM_021850340.1
XR_428922.3	XM_001652562.2
XR_428971.3	XM_001650364.2
XR_428993.3	XM_021852692.1
XR_429016.3	XR_002500197.1
XR_429135.2	XM_021840167.1
XR_429192.4	XM_001654226.2
XR_429333.3	XM_021839123.1
XR_429360.2	XR_002499401.1
XR_429740.2	XM_021837348.1
XR_429875.3	XR_002499327.1
XR_429913.4	XM_021856824.1
XR_430031.3	XM_001654740.2
XR_430124.4	XM_021857119.1
XR_430202.4	XM_001663079.2
XR_430301.3	XM_021854447.1
XR_430308.3	XR_002501367.1
XR_430821.3	XM_001661894.2
XR_922018.2	XM_001653349.2
XR_922067.2	XR_002499689.1
XR_922140.2	XM_021855030.1
XR_922148.2	XM_001655168.2
XR_922160.2	XM_021839345.1
XR_922213.1	XM_021843206.1
XR_922299.3	XM_021857057.1
XR_922306.2	XM_021853150.1
XR_922317.2	XM_001662129.2
XR_922341.2	XM_021855869.1
XR_922454.2	XM_021843516.1
XR_922459.2	XM_001660098.2
XR_922472.1	XM_021841159.1
XR_922508.2	XM_021840861.1
XR_922530.3	XM_021856867.1
XR_922599.1	XM_001657368.2
XR_922623.2	XR_002500355.1
XR_922667.1	XM_021845165.1
XR_922705.3	XM_001654193.2
XR_922815.2	XR_002500044.1
XR_922879.2	XR_002503081.1
XR_923022.3	XM_021852688.1
XR_923082.1	XM_021850176.1
XR_923085.2	XM_001649937.2
XR_923090.2	XM_021848155.1

XR_923105.2	XR_002500533.1
XR_923108.2	XM_001648019.2
XR_923125.1	XM_021852972.1
XR_923137.2	XM_021851726.1
XR_923142.2	XM_001653472.2
XR_923144.2	XM_021848156.1
XR_923175.2	XM_021841610.1
XR_923250.2	XM_021854603.1
XR_923288.1	XM_001648287.2
XR_923296.1	XM_021856875.1
XR_923388.1	XM_021857638.1
XR_923505.2	XM_021845163.1
XR_923509.2	XM_001647914.2
XR_923527.1	XM_021854540.1
XR_923591.3	XM_001656992.2
XR_923624.2	XM_021849185.1
XR_923747.2	XM_001654361.2
XR_923757.2	XM_001660868.2
XR_923855.2	XM_001659205.2
XR_923914.2	XM_021852635.1
XR_923916.2	XM_021852035.1
XR_923917.2	XM_021838779.1
XR_923954.1	XR_002501359.1
XR_924046.2	XM_001654190.2
XR_924073.3	XR_002499726.1
XR_924097.2	XM_021846121.1
XR_924162.3	XM_021854605.1
XR_924191.3	XM_001656340.2
XR_924256.2	XR_002499641.1
XR_924302.2	XM_021851749.1
XR_924434.1	XM_001655610.2
XR_924555.2	XM_021839346.1
XR_924563.2	XM_001659726.2
XR_924578.2	XM_001662220.2
XR_924807.2	XM_001652992.3
XR_924819.2	XM_021837281.1
XR_925034.2	XM_021840899.1
XR_925064.2	XM_021853802.1
XR_925198.1	XM_021838248.1
XR_925203.2	XM_021839281.1
XR_925257.3	XM_021840941.1
XR_925305.2	XM_021842653.1
XR_925402.2	XM_021846451.1
XR_925515.2	XM_021848837.1
XR_925530.2	XM_001658159.2
XR_925579.2	XR_002499030.1
XR_925644.2	XM_021842178.1
XR_925714.1	XM_021847258.1
XR_925762.2	XM_021855389.1
XR_925792.2	XR_002499877.1

XR_925956.3	XR_002499955.1
XR_926044.1	XR_002502781.1
XR_926099.2	XR_002499187.1
XR_926141.2	XM_021840483.1
XR_926166.2	XM_001661339.2
XR_926267.2	XR_002501224.1
XR_926402.2	XM_021847950.1
XR_926458.2	XM_021852687.1
XR_926469.2	XM_021845851.1
XR_926618.1	XM_021849180.1
XR_926620.2	XM_021847161.1
XR_926710.1	XM_011494657.2
XR_926747.2	XR_002499053.1
XR_926753.2	XM_001664022.2
XR_926757.2	XM_021847306.1
XR_926762.2	XM_001656288.2
XR_926780.2	XM_001651391.2
XR_926787.2	XR_002500332.1
XR_926797.2	XM_021851335.1
XR_926868.1	XR_002501001.1
XR_926894.2	XM_021847935.1
XR_926895.2	XM_021849373.1
XR_926908.2	XR_002501960.1
XR_926955.2	XM_001660047.2
XR_926956.2	XR_002501317.1
XR_927051.2	XR_002503168.1
XR_927111.2	XM_001651276.2
XR_927113.2	XM_001659158.2
XR_927123.2	XM_001653861.2
XR_927134.2	XM_021844808.1
XR_927174.2	XM_021848204.1
XR_927176.3	XM_021847257.1
XR_927187.2	XM_021852838.1
XR_927199.2	XM_021844629.1
XR_927421.1	XM_021848498.1
XR_927518.1	XM_021856872.1
XR_927559.2	XM_001661968.2
XR_927576.1	XR_002499832.1
XR_927649.2	XM_001663324.3
XR_927652.1	XR_002499977.1
XR_927698.2	XM_021846361.1
XR_927729.2	XM_021854604.1
XR_927826.3	XM_021842685.1
XR_927864.2	XM_021857028.1
XR_927885.2	XM_001661855.3
XR_927895.2	XM_001653515.2
XR_927952.3	XM_001655804.2
XR_927953.3	XR_002500019.1
XR_928005.2	XM_021852612.1
XR_928029.2	XM_021844207.1

XR_928033.1	XR_002499394.1
XR_928047.2	XM_021857400.1
XR_928059.2	XM_021841458.1
XR_928066.1	XM_021842687.1
XR_928171.2	XM_001649862.2
XR_928242.2	XM_021838753.1
XR_928260.1	XM_021843514.1
XR_928309.1	XM_021849178.1
XR_928312.3	XR_002503104.1
XR_928340.2	XR_002501594.1
XR_928399.2	XR_002502702.1
XR_928407.3	XM_021853153.1
XR_928448.3	XM_001655922.2
XR_928452.3	XM_001655381.2
XR_928461.2	XM_001661509.2
XR_928491.2	XM_021853010.1
XR_928650.2	XM_001648333.2
XR_928665.3	XM_001656591.2
XR_928727.1	XM_001649855.2
XR_928855.3	XM_011495222.2
XR_928863.2	XM_001657117.2
XR_928868.2	XM_001649044.2
XR_928896.2	XM_021854492.1
XR_928899.2	XM_021843686.1
XR_928910.2	XM_021839646.1
XR_928913.2	XM_021845640.1
XR_928934.2	XM_021849179.1
XR_928959.1	XM_021852255.1
XR_928962.2	XM_001656888.2
XR_928965.2	XR_002501761.1
XR_928967.1	XM_021852686.1
XR_928972.3	XM_001657191.2
XR_928993.2	XR_002502624.1
XR_929013.2	XM_021850637.1
XR_929027.2	XM_021857213.1
XR_929040.2	XM_001654336.2
XR_929113.2	XM_021846123.1
XR_929119.2	XR_002501891.1
XR_929159.2	XR_002502776.1
XR_929407.2	XM_001661708.2
XR_929462.2	XM_021852841.1
XR_929464.2	XR_002502168.1
XR_929529.2	XM_021852170.1
XR_929580.3	XR_002502936.1
XR_929605.1	XR_002498614.1
XR_929627.2	XM_021841375.1
XR_929731.3	XR_002501144.1
XR_929748.2	XM_001648676.3
XR_929767.1	XM_001655083.2
XR_929939.1	XM_021844960.1

XR_929945.1	XM_001652114.2
XR_929976.2	XM_021843513.1
XR_929983.2	XR_002502354.1
XR_929985.2	XR_002500905.1
XR_930039.2	XM_001662959.2
XR_930050.2	XR_002502234.1
XR_930068.2	XM_021853155.1
XR_930102.2	XM_021845630.1
XR_930133.2	XR_002499724.1
XR_930187.2	XR_002502766.1
XR_930195.1	XM_021855161.1
XR_930304.2	XM_001661927.2
XR_930345.1	XM_001655664.2
XR_930391.2	XM_001655753.2
XR_930393.2	XM_021837437.1
XR_930428.2	XR_002499388.1
XR_930469.2	XR_002499733.1
XR_930472.1	XR_002501583.1
XR_930475.2	XM_021851719.1
XR_930476.2	XM_021856865.1
XR_930488.1	XM_021837505.1
XR_930538.2	XM_021857629.1
XR_930542.2	XM_021852613.1
XR_930560.2	XR_002499009.1
XR_930564.2	XM_021857630.1
XR_930583.3	XR_002500951.1
XR_930643.1	XM_021854879.1
XR_930729.2	XM_001657170.2
XR_930755.3	XR_002501446.1
XR_930765.2	XR_002500525.1
XR_930828.2	XM_001662770.3
XR_930832.2	XM_021857628.1
XR_930903.3	XR_002502548.1
XR_930914.2	XM_021851983.1
XR_931009.2	XM_001649211.2
XR_931022.2	XM_021845241.1
XR_931118.2	XM_001658428.3
XR_931165.2	XR_002501669.1
XR_931178.2	XM_001656360.2
XR_931214.2	XM_021845187.1
XR_931241.2	XR_002498942.1
XR_931262.1	XR_002500304.1
XR_931351.2	XR_002500766.1
XR_931422.2	XR_002501077.1
XR_931448.2	XM_021842637.1
XR_931522.2	XR_002503201.1
XR_931541.2	XM_001651794.2
XR_931557.3	XM_001654085.2
XR_931568.2	XM_021840943.1
XR_931573.2	XR_002498637.1

XR_931621.2	XM_001649048.2
XR_931700.1	XM_021855595.1
XR_931734.2	XR_002502627.1
XR_931736.2	XR_002502863.1
XR_931777.1	XM_021839414.1
XR_931877.2	XM_021845939.1
XR_931938.2	XM_021838317.1
XR_931945.2	XM_001654730.2
XR_931993.2	XR_002502814.1
XR_932048.2	XR_002502825.1
XR_932069.3	XR_002501532.1
XR_932171.2	XR_002499432.1
XR_932236.2	XM_001663924.2
XR_932250.2	XM_001662063.2
XR_932255.3	XM_011495298.2
XR_932399.2	XR_002499008.1
XR_932404.3	XR_002502346.1
XR_932409.2	XM_001659710.2
XR_932424.2	XM_021838776.1
XR_932478.1	XM_021852227.1
XR_932493.2	XR_002500858.1
XR_932518.2	XM_021839952.1
XR_932542.2	XR_002503057.1
XR_932590.1	XR_002500333.1
XR_932615.3	XM_021852172.1
XR_932644.2	XM_021853850.1
XR_932669.2	XR_002500207.1
XR_932838.3	XR_002501993.1
XR_932928.3	XR_002502204.1
XR_933085.2	XR_002499110.1
XR_933149.3	XR_002500004.1
XR_933341.3	XM_001651106.2
XR_933352.2	XM_001657076.2
XR_933433.3	XM_001650350.2
XR_933519.2	XM_001651505.2
XR_933596.2	XR_002499967.1
XR_933619.3	XR_002503150.1
XR_933623.2	XR_002503083.1
XR_933711.2	XM_001656141.2
XR_933729.3	XM_001649043.2
XR_933754.2	XR_002500260.1
XR_933786.2	XM_021853868.1
XR_934174.2	XR_002500436.1
XR_934178.2	XM_001652520.2
XR_934252.2	XR_002501159.1
XR_934258.2	XM_021839816.1
XR_934312.3	XM_001653027.2
XR_934326.2	XM_021842689.1
XR_934369.1	XM_001660285.2
XR_934476.3	XR_002500686.1

XR_934502.1	XM_021849451.1
XR_934535.1	XM_001660437.2
XR_934637.3	XR_002500623.1
XR_934687.2	XM_021840900.1
XR_934751.2	XM_021856689.1
XR_934753.2	XM_021846306.1
XR_934771.2	XR_002499749.1
XR_934811.2	XM_021854556.1
XR_934874.3	XM_021856228.1
XR_934976.2	XM_021854838.1
XR_935089.2	XM_001657773.2
XR_935157.2	XM_021839551.1
XR_935171.2	XM_021853851.1
XR_935195.1	XM_001647696.2
XR_935200.1	XM_001658854.2
XR_935202.1	XR_002503250.1
XR_935325.1	XM_021842320.1
XR_935327.2	XM_001653431.2
XR_935378.3	XM_001650951.2
XR_935432.2	XR_002498876.1
XR_935456.2	XM_001649950.2
XR_935559.2	XR_002499670.1
XR_935599.2	XR_002499634.1
XR_935690.3	XR_002502201.1
XR_935712.2	XM_001657878.2
XR_935741.2	XM_021842956.1
XR_935812.3	XR_002502878.1
XR_935823.1	XM_021857241.1
XR_935866.2	XM_001661702.2
XR_935896.2	XR_002499573.1
XR_935942.2	XR_002500743.1
XR_935966.2	XM_021857131.1
XR_936006.2	XM_021839658.1
XR_936015.2	XM_001662431.2
XR_936052.2	XR_002499387.1
XR_936074.2	XR_002500483.1
XR_936091.2	XR_002503213.1
XR_936111.2	XM_021857127.1
XR_936137.3	XR_002501873.1
XR_936169.2	XM_001649678.2
XR_936174.1	XR_002500368.1
XR_936272.2	XR_002498705.1
XR_936283.2	XR_002499964.1
XR_936472.1	XM_021852365.1
XR_936554.2	XM_001649308.2
XR_936626.2	XM_021846055.1
XR_936728.2	XM_001655942.2
XR_936739.3	XM_001657238.3
XR_936750.3	XR_002501658.1
XR_936764.2	XR_002502625.1

XR_936847.2	XR_002503152.1
XR_936848.2	XR_002498948.1
XR_936896.2	XR_002499884.1
XR_936898.2	XM_021838437.1
XR_936911.2	XR_002499711.1
XR_936916.2	XM_021844413.1
XR_936962.1	XR_002502385.1
XR_936963.1	XR_002501391.1
XR_936968.2	XM_021837443.1
XR_936984.2	XR_002503069.1
XR_937057.1	XR_002500908.1
XR_937251.2	XM_001660286.2
XR_937263.2	XM_001661470.2
XR_937267.2	XR_002499798.1
XR_937268.2	XR_002501435.1
XR_937394.2	XR_002502688.1
XR_937396.2	XR_002501566.1
XR_937438.2	XR_002503075.1
XR_937606.3	XM_001660459.2
XR_937637.2	XR_002502947.1
XR_937688.3	XM_001654154.2
XR_937739.1	XR_002502467.1
XR_937748.3	XR_002502295.1
XR_937786.3	XR_002501456.1
XR_937811.2	XR_002501377.1
XR_937819.1	XM_001650457.2
XR_937827.3	XM_001659009.2
XR_937856.1	XR_002500907.1
XR_937863.2	XR_002502879.1
XR_937952.2	XR_002502472.1
XR_938055.2	XR_002501734.1
XR_938058.2	XR_002502572.1
XR_938072.2	XR_002502427.1
XR_938076.1	XR_002498621.1
XR_938086.2	XM_011494699.2
XR_938102.2	XR_002500352.1
XR_938131.2	XR_002500054.1
XR_938139.2	XR_002500703.1
XR_938171.2	XM_001655456.2
XR_938212.2	XM_001652391.2
XR_938226.2	XM_021845976.1
XR_938283.2	XR_002498721.1
XR_938289.2	XM_021855248.1
XR_938433.1	XR_002502773.1
XR_938507.2	XR_002499490.1
XR_938516.1	XR_002501119.1
XR_938534.1	XR_002498795.1
XR_938603.3	XR_002501178.1
XR_938641.2	XM_021838628.1
XR_938677.3	

XR_938679.1
XR_938701.3
XR_938753.2
XR_938771.1
XR_938780.3
XR_938796.3
XR_938854.1
XR_938919.2
XR_938920.2
XR_938931.2
XR_938935.2
XR_938957.3
XR_938977.2
XR_939091.2
XR_939185.2
XR_939242.2
XR_939498.2
XR_939522.2
XR_939620.2
XR_939722.2
XR_939785.2
XR_939834.1
XR_939844.2
XR_939854.2
XR_939873.2
XR_939886.2
XR_939974.2
XR_940002.3
XR_940019.2
XR_940067.2
XR_940083.1
XR_940093.1
XR_940111.2
XR_940183.3
XR_940187.3
XR_940205.2
XR_940228.2
XR_940235.2
XR_940260.1
XR_940281.2
XR_940317.2
XR_940392.3
XR_940455.2
XR_940541.3
XR_940586.1
XR_940609.2
XR_940700.3
XR_940733.1
XR_940782.3
XR_940846.2

XR_940857.2
XR_940859.1
XR_940897.3
XR_940970.2
XR_940974.2
XR_940990.2
XR_940997.2
XR_941024.2
XR_941077.1
XR_941191.2
XR_941198.2
XR_941226.2
XR_941265.2
XR_941266.2
XR_941302.2
XR_941314.2
XR_941352.2
XR_941358.2
XR_941368.2
XR_941401.2
XR_941422.3
XR_941546.1
XR_941752.1
XR_941758.1
XR_941779.3
XR_941849.2
XR_941947.1
XR_941954.2
XR_941972.2
XR_942014.1
XR_942092.2
XR_942116.2
XR_942124.2
XR_942150.1
XR_942152.1
XR_942158.3
XR_942305.3
XR_942460.2
XR_942666.1
XR_942676.2
XR_942693.3
XR_942699.3
XR_942738.3
XR_942765.3
XR_942866.2
XR_942881.2
XR_942901.2
XR_942987.2
XR_943084.3
XR_943085.3

XR_943136.2
XR_943176.1
XR_943270.2
XR_943413.3
XR_943577.1
XR_943586.2
XR_943610.2
XR_943636.2
XR_943640.2
XR_943701.2
XR_943705.2
XR_943724.2
XR_943736.2
XR_943771.2
XR_943864.3
XR_943908.2
XR_943932.2
XR_943953.2
XR_943955.3
XR_943977.2
XR_944008.2
XR_944013.3
XR_944089.2
XR_944155.2
XR_944157.1
XR_944172.1
XR_944174.2
XR_944187.3
XR_944190.2
XR_944191.2
XR_944321.3
XR_944329.3
XR_944386.2
XR_944428.3
XR_944432.2
XR_944515.2
XR_944537.2
XR_944581.2
XR_944864.2
XR_944868.2
XR_944901.2
XR_944904.2
XR_944974.2
XR_945015.2
XR_945054.2
XR_945057.2
XR_945081.2
XR_945188.1
XR_945238.2
XR_945280.2

XR_945284.3
XR_945329.2
XR_945356.1
XR_945365.3
XR_945389.2
XR_945416.1
XR_945429.2
XR_945449.2
XR_945453.2
XR_945520.1
XR_945568.2
XR_945949.2
XR_945957.2
XR_945991.2
XR_946003.2
XR_946152.2
XR_946198.2
XR_946246.2
XR_946309.2
XR_946513.3
XR_946521.3
XR_946523.1
XR_946576.2
XR_946636.2
XR_946642.2
XR_946665.1
XR_946674.2
XR_946682.2
XR_946721.2
XR_946764.1
XR_946802.3
XR_946869.2
XR_946892.2
XR_946952.2
XR_947005.2
XR_947088.2
XR_947141.2
XR_947147.2
XR_947174.2
XR_947255.2
XR_947273.2
XR_947278.2
XR_947280.2
XR_947289.1
XR_947488.2
XR_947518.2
XR_947529.2
XR_947603.2
XR_947608.2
XR_947636.1

XR_947682.2
XR_947727.1
XR_947807.1
XR_947834.2
XR_947851.2
XR_947940.2
XR_948008.2
XR_948039.1
XR_948057.2
XR_948092.3
XR_948352.2
XR_948410.2
XR_948435.2
XR_948528.2
XR_948533.2
XR_948540.2
XR_948581.2
XR_948623.2
XR_948664.2
XR_948692.2
XR_948899.3
XR_948944.3
XR_948956.2
XR_948958.3
XR_948965.1
XR_949045.1
XR_949163.3
XR_949201.3
XR_949269.2
XR_949286.2
XR_949402.3
XR_949563.2
XR_949584.2
XR_949692.2
XR_949693.2
XR_949694.3
XR_949705.1
XR_949706.2
XR_949933.3
XR_949950.2
XR_950008.3
XR_950143.2
XR_950215.2
XR_950235.2
XR_950265.2
XR_950334.1
XR_950580.1
XR_950632.1
XR_950638.2
XR_950767.3

XR_950818.3
XR_950871.2
XR_950891.2
XR_950945.3
XR_951108.2
XR_951291.2
XR_951312.2
XR_951398.2
XR_951426.3
XR_951438.2
XR_951439.2
XR_951443.2
XR_951449.2
XR_951681.2
XR_951862.2
XR_951872.2
XR_951914.2
XR_951920.2
XR_951932.3
XR_951996.2
XR_952154.1
XR_952182.3
XR_952219.2
XR_952247.2
XR_952538.2
XR_952545.2
XR_952686.2
XR_952697.2
XR_952711.2
XR_952881.3
XR_952979.1
XR_953178.3
XR_953253.2
