# Supplementary material for: The dinucleotide composition of the Zika virus genome is shaped by conflicting evolutionary pressures in mammalian hosts and mosquito vectors
Source: PLoS Biol. 2021 Apr 19;19(4):e3001201. doi: 10.1371/journal.pbio.3001201 (PMC8084339; doi:10.1371/journal.pbio.3001201)
Supplement: S2 Text — Nucleotide sequences of the selected region within Zika virus for all mutant and WT sequences in FASTA format. (PDF) [file pbio.3001201.s007.pdf]

```
>Wildtype
ACCGCCTGGGGGTGGGGGGAGGCTGGGGCCCTGATCACAGCCGCAACTTCCACTTTGTGGGAAGGCTCTCCGA
ACAAGTACTGGAACTCCTCTACAGCCACTTCACTGTGTAACATTTTTAGGGGAAGTTACTTGGCTGGAGCTTC
TCTAATCTACACAGTAACAAGAAACGCTGGCTTGGTCAAGAGACGTGGGGGTGGAACAGGAGAGACCCTGGGA
GAGAAATGGAAGGCCCGCTTGAACCAGATGTCGGCCCTGGAGTTCTACTCCTACAAAAAGTCAGGCATCACCG
AGGTGTGCAGAGAAGAGGCCCGCCGCGCCCTCAAGGACGGTGTGGCAACGGGAGGCCATGCTGTGTCCCGAGG
AAGTGCAAAGCTGAGATGGTTGGTGGAGCGGGGATACCTGCAGCCCTATGGAAAGGTCATTGATCTTGGATGT
GGCAGAGGGGGCTGGAGTTACTACGCCGCCACCATCCGCAAAGTTCAAGAAGTGAAAGGATACACAAAAGGAG
GCCCTGGTCATGAAGAACCCGTGTTGGTGCAAAGCTATGGGTGGAACATAGTCCGTCTTAAGAGTGGGGTGGA
CGTCTTTCATATGGCGGCTGAGCCGTGTGACACGCTGCTGTGTGACATAGGTGAGTCATCATCTAGTCCTGAA
GTGGAAGAAGCACGGACGCTCAGAGTCCTCTCCATGGTGGGGGATTGGCTTGAAAAAAGACCAGGAGCCTTTT
GTATAAAAGTGTTGTGCCCATACACCAGCACTATGATGGAAACCCTGGAGCGACTGCAGCGTAGGTATGGGGG
AGGACTGGTCAGAGTGCCACTCTCCCGCAACTCTACACATGAGATGTACTGGGTCTCTGGAGCGAAAAGCAAC
ACCATAAAAAGTGTGTCCACCACGAGCCAGCTCCTCTTGGGGCGCATGGACGGGCCTAGAAGGCCAGTGAAAT
ATGAGGAGGATGTGAATCTCGGCTCTGGCACGCGGGCTGTGGTAAGCTGCGCTGAAGCTCCCAACATGAAGAT
CATTGGTAACCGCATTGAAAGGATCCGCAGTGAGCACGCGGAAACGTGGTTCTTTGACGAGAACCACCCATAT
AGGACATGGGCTTACCATGGAAGCTATGAGGCCCCCACACAAGGGTCAGCGTCCTCTCTAATAAACGGGGTTG
TCAGGCTCCTGTCAAAACCCTGGGATGTGGTGACTGGAGTCACAGGAATAGCCATGACCGACACCACACCGTA
TGGTCAGCAAAGAGTTTTCAAGGAAAAAGTGGACACTAGGGTGCCAGACCCCCAAGAAGGCACTCGTCAGGTT
ATGAGCATGGTCTCTTCCTGGTTGTGGAAAGAGCTAGGCAAACACAAACGGCCACGAGTCTGTACCAAAGAAG
AGTTCATCAACAAGGTTCGTAGCAATGCAGCATTAGGGGCAATATTTGAAGAGGAAAAAGAGTGGAAGACTGC
AGTGGAAGCTGTGAACGATCCAAGGTTCTGGGCTCTAGTGGACAAGGAAAGAGAGCACCACCTGAGAGGAGAG
TGCCAGAGTTGTGTGTATAACATGATGGGAAAAAGAGAAAAGAAACAAGGGGAATTTGGAAAGGCCAAGGGCA
GCCGCGCCATCTGGTATATGTGGCTAGGGGCTAGATTTCTAGAGTTCG

>SCR
ACCGCATGGGGATGGGGAGAGGCAGGGGCACTGATCACCGCCGCAACATCGACATTGTGGGAAGGCTCCCCAA
ACAAGTACTGGAACTCCTCCACCGCCACCTCCCTGTGTAACATCTTCAGAGGCAGCTACTTAGCTGGCGCATC
TCTGATCTACACAGTGACCAGGAACGCCGGTTTGGTTAAGAGGCGAGGGGGTGGAACTGGTGAGACTCTAGGA
GAGAAGTGGAAGGCTCGATTGAACCAGATGTCGGCCCTGGAGTTTTACTCGTACAAAAAGTCAGGTATCACAG
AGGTTTGCAGAGAAGAGGCGCGTCGCGCCCTCAAGGACGGAGTAGCAACGGGTGGCCATGCAGTCTCGCGAGG
TAGTGCAAAACTGAGATGGTTGGTGGAGCGGGGATATCTGCAGCCGTATGGCAAGGTAATTGATCTTGGGTGC
GGCAGAGGGGGTTGGAGCTATTACGCTGCCACCATCCGCAAGGTGCAGGAAGTGAAGGGTTACACGAAAGGGG
GCCCGGGGCATGAAGAACCTGTCTTAGTGCAAAGTTATGGATGGAACATAGTCCGTCTGAAGAGTGGGGTTGA
TGTGTTTCATATGGCTGCTGAACCCTGTGACACACTGCTCTGTGACATTGGAGAGTCGTCCTCCAGCCCAGAA
GTAGAGGAAGCCCGCACCCTCAGAGTGCTTTCCATGGTGGGGGACTGGCTAGAGAAAAGACCAGGAGCATTCT
GTATCAAAGTGTTGTGCCCTTACACCAGCACTATGATGGAGACACTGGAGCGGCTTCAGCGGAGATATGGAGG
GGGTCTGGTCAGAGTGCCTCTCTCCCGGAACTCAACTCATGAGATGTACTGGGTGTCAGGTGCCAAGAGCAAC
ACCATAAAGAGTGTCTCAACGACCAGTCAGCTCCTCTTGGGACGAATGGATGGACCAAGGAGGCCAGTGAAGT
ACGAGGAAGATGTAAACCTGGGCTCAGGCACCCGTGCTGTTGTAAGCTGCGCTGAAGCCCCCAACATGAAGAT
CATTGGCAACCGCATTGAAAGGATTCGCAGTGAGCACGCAGAAACTTGGTTCTTCGACGAGAATCACCCCTAT
AGAACATGGGCTTACCATGGAAGCTATGAGGCCCCCACACAAGGCTCAGCCTCATCTCTGATCAACGGAGTTG
TCAGACTCCTGTCAAAGCCTTGGGATGTGGTGACGGGAGTCACAGGAATTGCCATGACTGACACCACCCCCTA
TGGACAGCAGAGGGTGTTCAAGGAAAAAGTGGACACAAGGGTCCCGGACCCCCAAGAGGGAACACGCCAAGTC
ATGAGTATGGTGTCTTCTTGGTTGTGGAAAGAGCTTGGCAAGCACAAACGGCCTCGAGTTTGTACCAAAGAAG
AGTTCATTAACAAGGTGCGAAGCAATGCAGCATTAGGGGCAATTTTTGAAGAGGAAAAAGAGTGGAAAACGGC
AGTGGAAGCTGTAAACGATCCAAGATTCTGGGCGCTGGTGGACAAGGAAAGAGAGCATCATCTGAGAGGAGAG
TGCCAGAGTTGTGTTTATAACATGATGGGCAAAAGGGAGAAAAAGCAAGGAGAATTTGGAAAGGCCAAGGGCA
GCCGAGCCATCTGGTATATGTGGCTAGGGGCAAGATTCCTAGAGTTCG

>UpA_max          TpA^Target_ratio=2.500^
ACCGCCTGGGGGTGGGGGGAGGCTGGGGCCCTAATAACAGCCGCTACTTCTACTTTATGGGAAGGCTCTCCGA
ATAAGTACTGGAACTCCTCTACAGCTACTTCACTATGTAATATATTTAGGGGTAGTTACTTAGCTGGAGCTTC
TCTAATATATACAGTAACTAGAAACGCTGGCTTAGTTAAGAGACGTGGGGGTGGTACAGGAGAGACCCTAGGA
GAGAAATGGAAGGCCCGCTTAAACCAGATGTCGGCCCTAGAGTTCTACTCCTATAAAAAGTCAGGTATTACCG
AGGTATGTAGAGAAGAGGCCCGCCGCGCCCTTAAGGACGGTGTAGCTACGGGAGGCCATGCTGTATCCCGAGG
TAGTGCTAAGCTTAGATGGTTAGTAGAGCGGGGATACCTACAGCCCTATGGTAAGGTTATAGATCTAGGATGT
GGTAGAGGGGGCTGGAGTTACTACGCCGCTACTATACGTAAAGTACAAGAAGTTAAAGGATATACTAAAGGAG
```

GCCCTGGTCATGAAGAACCCGTATTAGTACAAAGCTATGGGTGGAATATAGTACGTCTTAAGAGTGGGGTAGA
CGTATTTCATATGGCGGCTGAGCCGTGTGATACGCTACTATGTGATATAGGTGAGTCATCATCTAGTCCTGAA
GTAGAAGAAGCACGTACGCTTAGAGTACTATCTATGGTAGGGGATTGGCTAGAAAAAAGACCAGGAGCCTTTT
GTATAAAAGTATTATGCCCATATACTAGTACTATGATGGAAACCCTAGAGCGACTACAGCGTAGGTATGGGGG
AGGACTAGTAAGAGTACCACTATCCCGTAACTCTACACATGAGATGTACTGGGTATCTGGAGCGAAAAGTAAT
ACTATAAAAAGTGTATCTACTACGAGCCAGCTACTATTAGGGCGTATGGACGGGCCTAGAAGGCCAGTTAAAT
ATGAGGAGGATGTAAATCTCGGCTCTGGTACGCGGGCTGTAGTAAGCTGCGCTGAAGCTCCTAATATGAAGAT
AATAGGTAACCGTATAGAAAGGATACGTAGTGAGCACGCGGAAACGTGGTTCTTTGACGAGAACCACCCATAT
AGGACATGGGCTTACCATGGTAGCTATGAGGCCCCTACACAAGGGTCAGCGTCCTCTCTAATAAACGGGGTAG
TAAGGCTACTATCTAAACCCTGGGATGTAGTTACTGGAGTAACAGGTATAGCTATGACCGATACTACACCGTA
TGGTCAGCAAAGAGTATTTAAGGAAAAAGTAGATACTAGGGTACCAGACCCCCAAGAAGGTACTCGTCAGGTT
ATGAGTATGGTATCTTCCTGGTTATGGAAAGAGCTAGGTAAACATAAACGGCCACGAGTATGTACTAAAGAAG
AGTTTATTAATAAGGTACGTAGTAATGCAGCATTAGGGGCTATATTTGAAGAGGAAAAAGAGTGGAAGACTGC
AGTAGAAGCTGTAAACGATCCTAGGTTCTGGGCTCTAGTAGATAAGGAAAGAGAGCACCACCTTAGAGGAGAG
TGCCAGAGTTGTGTATATAATATGATGGGTAAAAGAGAAAAGAAACAAGGGGAATTTGGTAAGGCTAAGGGTA
GCCGCGCTATATGGTATATGTGGCTAGGGGCTAGATTTCTAGAGTTCG

>CpG_max hi CpG^Target_ratio=2.500^
ACCGCCTGGGGGTGGGGCGAGGCCGGCGCGCTGATCACCGCCGCGACGTCGACGTTGTGGGAAGGCTCGCCGA
ACAAGTACTGGAACTCGTCTACCGCGACGTCGCTGTGTAACATTTTTAGGGGAAGTTACTTGGCCGGCGCGTC
GCTAATCTACACGGTAACGCGAAACGCCGGCTTGGTCAAGCGACGCGGCGGCGGAACCGGCGAGACGCTCGGC
GAGAAATGGAAGGCGCGCTTGAACCAGATGTCGGCGCTCGAGTTCTACTCGTACAAAAAGTCGGGCATCACCG
AGGTGTGCCGCGAAGAGGCGCGCCGCGCGCTCAAGGACGGCGTCGCGACGGGCGGCCACGCGGTGTCGCGCGG
AAGCGCGAAGCTGCGATGGTTGGTCGAGCGCGGATACCTGCAGCCGTACGGAAAGGTCATCGATCTCGGATGC
GGCCGCGGCGGCTGGAGTTACTACGCCGCGACGATCCGCAAAGTTCAAGAAGTGAAAGGATACACGAAAGGCG
GCCCCGGTCACGAAGAACCCGTGTTGGTGCAAAGCTACGGGTGGAACATAGTCCGTCTTAAGAGCGGCGTCGA
CGTCTTTCATATGGCGGCGGAGCCGTGCGACACGCTGCTGTGCGACATAGGCGAGTCGTCGTCTAGTCCGGAA
GTCGAAGAAGCGCGGACGCTCCGCGTCCTCTCGATGGTCGGCGATTGGCTCGAAAAACGACCGGGCGCGTTTT
GTATAAAAGTGTTGTGCCCGTACACGAGCACTATGATGGAAACGCTCGAGCGCCTGCAGCGTAGGTACGGCGG
CGGACTCGTCCGCGTGCCGCTCTCGCGCAACTCTACGCACGAGATGTACTGGGTCTCCGGCGCGAAAAGCAAC
ACGATAAAAAGCGTGTCGACGACGAGCCAGCTCCTCTTGGGGCGCATGGACGGGCCTAGACGGCCGGTGAAAT
ACGAGGAGGACGTGAATCTCGGCTCCGGCACGCGCGCCGTCGTAAGCTGCGCCGAAGCGCCGAACATGAAGAT
CATCGGTAACCGCATCGAACGAATCCGCAGCGAGCACGCGGAAACGTGGTTCTTCGACGAGAACCACCCGTAT
AGGACGTGGGCGTACCACGGAAGCTACGAGGCGCCGACGCAAGGGTCCGCGTCGTCGCTAATAAACGGCGTCG
TCCGGCTCCTGTCGAAACCGTGGGACGTCGTGACCGGCGTCACCGGAATAGCGATGACCGACACGACGCCGTA
CGGTCAGCAACGCGTTTTCAAGGAAAAAGTCGACACTAGGGTGCCGGACCCGCAAGAAGGCACGCGTCAGGTT
ATGAGCATGGTCTCGTCGTGGTTGTGGAAAGAGCTAGGCAAACACAAACGGCCGCGCGTCTGTACGAAAGAAG
AGTTCATCAACAAGGTTCGTAGCAACGCGGCGTTAGGCGCGATATTCGAGGAGGAAAAAGAGTGGAAGACCGC
CGTCGAAGCCGTGAACGATCCGCGGTTCTGGGCGCTAGTCGACAAGGAACGCGAGCACCACCTGCGCGGCGAG
TGCCAGAGTTGCGTGTATAACATGATGGGAAAACGCGAAAAGAAACAAGGCGAATTCGGAAAGGCGAAGGGCA
GCCGCGCGATCTGGTATATGTGGCTAGGCGCTAGATTTCTAGAGTTCG

>CpG_1.0 CpG^Target_ratio=1.000^
ACCGCCTGGGGCTGGGGTGAGGCTGGAGCACTCATCACCGCCGCAACGTCGACGTTGTGGGAAGGCTCGCCGA
ACAAGTACTGGAACTCGTCTACGGCCACGTCACTGTGTAACATTTTTAGGGGAAGTTACTTGGCGGGAGCTTC
GCTAATCTACACGGTAACACGAAACGCGGGCTTGGTCAAGAGAAGGGGCGGTGGAACAGGAGAGACTCTGGGT
GAGAAATGGAAGGCGAGATTGAACCAGATGTCCGCGCTCGAGTTCTACTCCTACAAAAAGTCCGGCATCACGG
AGGTGTGCAGAGAAGAGGCGCGTCGCGCTCTAAAGGACGGTGTCGCAACTGGAGGCCATGCTGTGTCGCGCGG
AAGTGCAAAGCTGAGATGGTTGGTTGAGCGAGGATACCTGCAGCCGTATGGAAAGGTCATTGATCTTGGATGT
GGCAGAGGTGGCTGGAGTTATTATGCCGCAACGATCCGCAAAGTTCAAGAAGTGAAAGGATACACAAAAGGAG
GACCGGGTCATGAAGAACCAGTTTTGGTGCAAAGCTACGGGTGGAACATAGTACGTCTTAAGAGTGGGGTAGA
CGTCTTTCATATGGCTGCCGAGCCGTGTGACACCCTCCTTTGCGACATCGGTGAGTCGTCATCTAGTCCTGAA
GTCGAAGAAGCACGGACTCTCCGCGTCCTCTCGATGGTGGGGGATTGGCTTGAAAAAAGACCGGGCGCCTTCT
GTATAAAAGTGTTGTGCCCATACACCAGCACTATGATGGAAACTCTCGAGAGACTGCAGCGTAGGTATGGCGG
CGGACTCGTCAGAGTGCCACTCTCTCGAAACTCTACACATGAGATGTACTGGGTGTCTGGAGCGAAAAGCAAC
ACGATAAAAAGTGTTTCCACGACAAGCCAGCTCCTATTGGGGAGGATGGATGGTCCTAGAAGGCCAGTGAAAT
ATGAGGAGGATGTAAATCTCGGCTCTGGGACTCGAGCCGTGGTAAGCTGCGCTGAAGCGCCGAACATGAAGAT
CATTGGTAACCGCATTGAACGAATCCGCAGCGAGCACGCTGAAACATGGTTCTTTGACGAGAACCACCCCTAT

AGGACATGGGCGTACCATGGAAGCTATGAGGCGCCGACACAAGGTTCTGCGTCCTCTCTAATAAACGGGGTCG
TCCGACTCCTGTCAAAACCCTGGGATGTGGTGACGGGGGTCACGGGAATAGCGATGACGGACACCACACCATA
CGGTCAGCAACGAGTTTTCAAGGAAAAAGTCGACACTCGCGTGCCAGACCCGCAAGAAGGCACTCGTCAGGTT
ATGAGCATGGTCTCGTCGTGGTTGTGGAAAGAACTAGGCAAACACAAAAGACCACGAGTCTGTACGAAAGAAG
AGTTCATCAACAAGGTTAGAAGCAACGCCGCATTAGGCGCAATATTTGAAGAGGAAAAAGAGTGGAAGACTGC
CGTGGAAGCTGTGAACGATCCACGGTTCTGGGCTCTCGTCGACAAGGAAAGAGAGCATCACCTCAGAGGAGAG
TGCCAGAGTTGTGTGTATAACATGATGGGAAAAAGAGAAAAGAAACAAGGCGAATTTGGAAAGGCCAAGGGCA
GCCGCGCGATCTGGTATATGTGGCTCGGCGCGCGATTTCTAGAGTTCG
